# Microwave-assisted synthesis of tubulin assembly inhibitors as anticancer agents by aryl ring reversal and conjunctive approach†

**DOI:** 10.1039/d5md00406c

**Published:** 2025-07-02

**Authors:** Domiziana Masci, Michela Puxeddu, Claudia Colla, Antonio Coluccia, Martina Santelli, Pietro Sciò, Elena Mariotto, Giampietro Viola, Ernest Hamel, Rosa Lerose, Carmela Mazzoccoli, Romano Silvestri, Giuseppe La Regina

**Affiliations:** a Department of Basic Biotechnological Sciences, Intensivological and Perioperative Clinics, Catholic University of the Sacred Heart Largo Francesco Vito 1 00168 Rome Italy; b Policlinico Universitario A. Gemelli Foundation-IRCCS 00168 Rome Italy; c Laboratory affiliated with the Institute Pasteur Italy – Cenci Bolognetti Foundation, Department of Drug Chemistry and Technologies, Sapienza University of Rome Piazzale Aldo Moro 5 00185 Rome Italy romano.silvestri@uniroma1.it; d Department of Woman's and Child's Health, University of Padua, Hemato-oncology Lab Via Giustiniani 3 35128 Padua Italy; e Istituto di Ricerca Pediatrica Città della Speranza – IRP Corso Stati Uniti 4-35127 Padua Italy; f Molecular Pharmacology Branch, Developmental Therapeutics Program, Division of Cancer Treatment and Diagnosis, Frederick National Laboratory for Cancer Research, National Cancer Institute, National Institutes of Health Frederick Maryland 21702 USA; g Hospital Pharmacy, Centro di Riferimento Oncologico della Basilicata (IRCCS-CROB) 85028 Rionero in Vulture Italy

## Abstract

Microwave-assisted synthesis of new pyrrole and indole derivatives as tubulin assembly inhibitors was performed with remarkably improved yields and short reaction times. In designing the new inhibitors, aryl ring reversal and conjunctive approach notions were applied. (4-(4-Methoxyphenyl)-1-(pyridin-2-yl)-1*H*-pyrrol-3-yl)(3,4,5-trimethoxyphenyl) methanone (4) inhibited [^3^H] colchicine binding by 78% and MCF-7 breast cancer cell growth with an IC_50_ of 9.6 nM. Compound 4 also inhibited the growth of HCT116, BX-PC3 and Jurkat cancer cells with IC_50_ values of 18, 17 and 41 nM, respectively, and altered the morphology of treated spheroids in both the BX-PC3 and HCT116 cell lines.

## Abstract 2

Microwave-assisted synthesis of new pyrrole and indole derivatives as tubulin assembly inhibitors was performed with remarkably improved yields and short reaction times.

## Graphical abstract

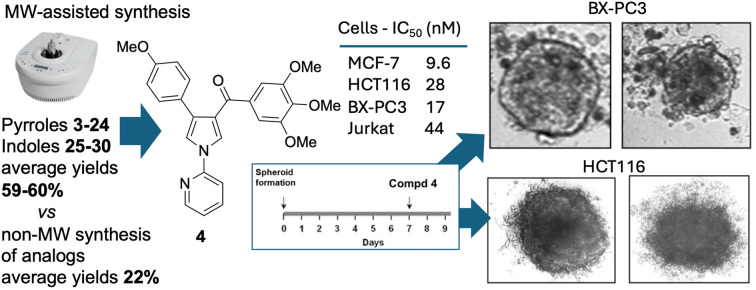

## 1. Introduction

Chemotherapy is the first-line treatment for patients who received poor or no response from cancer surgery or irradiation. Microtubules play a key role in pivotal cellular processes, including spindle formation, intracellular transport and maintenance of cellular shape. Due to the prominent role played by microtubules during mitosis, interfering with microtubule/tubulin dynamics rapidly became a major strategy in the search for new anti-cancer agents.^1^ Tubulin polymerization inhibitors (colchicinoids, vinca alkaloids) and tubulin stabilizers (taxanes, epothilones) effectively inhibit cell growth with induction of apoptosis. However, most anti-tubulin agents show an unfavorable therapeutic index due to their toxic effects on normal cells, in addition to their toxicity for cancer cells.^2^

Compared to taxanes and vinca alkaloids, colchicine site compounds offer therapeutic advantages, since they are less vulnerable to overexpression of the ABC-transporters responsible for multidrug resistance (MDR), can have better aqueous solubility to facilitate oral administration and show vascular disrupting activity.^3^ Several colchicine site anti-tubulin agents have shown promising anticancer effects in clinical trials.^4^

We applied a microwave (MW)-assisted procedure to synthesize a series of pyrrole (3–24) and indole (25–30) colchicine site derivatives, with the dual objectives of enhancing overall reaction efficiency and aligning with the principles of green chemistry, including optimized energy usage, minimization of waste generation and the use of safer solvents. MW irradiation indeed represents a more sustainable alternative to conventional heating, primarily due to its ability to bypass the limitations of thermal conductivity. By enabling direct coupling of electromagnetic energy with polar molecules or solvents, it facilitates rapid internal heating, thereby significantly reducing reaction times while enhancing yields. This methodology is particularly attractive in medicinal chemistry, where time-efficient and scalable synthetic strategies are essential for accelerating the drug discovery and development process.

## 2. Results and discussion

### 2.1. Rational design

**Chart 1:**
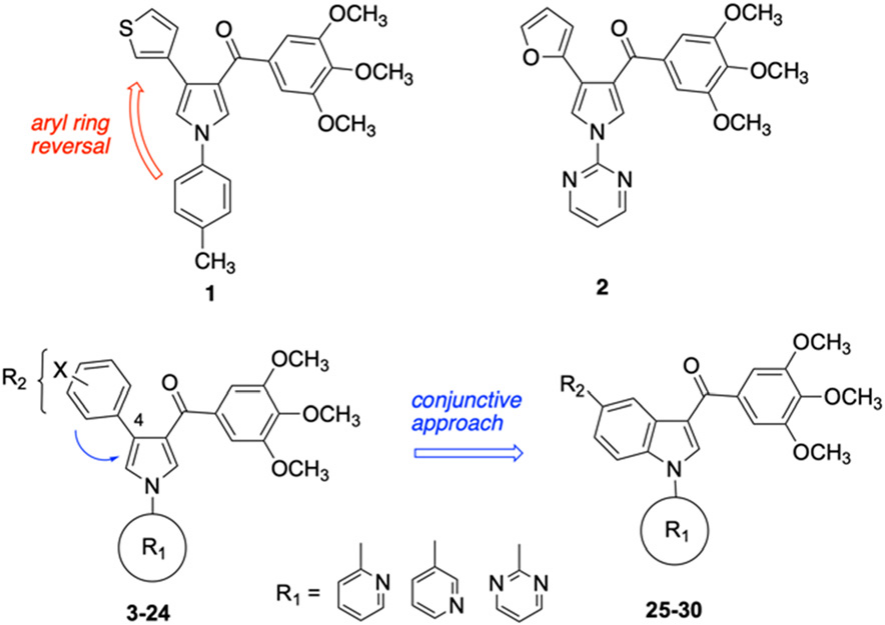
General structures: reference compounds 1 and 2, new pyrrole 3–24 and indole 25–30 derivatives. See Tables 1 and 2 for *R*_1_ and *R*_2_ values.

In this work, we shifted the 4-methylphenyl ring at position 1 of the pyrrole compound 1 (ref. 5) to position 4 (aryl ring reversal) (3–24) and fused this aryl with the pyrrole to form an indole ring (conjunctive approach) (25–30) while keeping at position 1 a heterocyclic ring, pyridine or pyrimidine, as in compound 2 (ref. 6) (Chart 1). Neither approach affects the global complexity of the parent compounds and are expected to yield close analogs maintaining or improving the activity of the original active principle.^7^ We found a new 4-aryl-1-heterocyclyl pyrrole derivative, 4, whose mechanism is the inhibition of tubulin assembly, and exhibited strong inhibition of MCF-7 breast cancer cell growth and of [^3^H]colchicine binding to tubulin.

Compound 4 also effectively inhibited the growth of HCT116 (colorectal), BX-PC3 (pancreatic) and Jurkat (T-cell acute lymphoblastic leukemia) cancer cells, and altered the morphology of treated spheroids in both the BX-PC3 and HCT116 cell lines.

### 2.2. Chemical synthesis

#### 2.2.1. Synthesis of 3–24

**Scheme 1:**
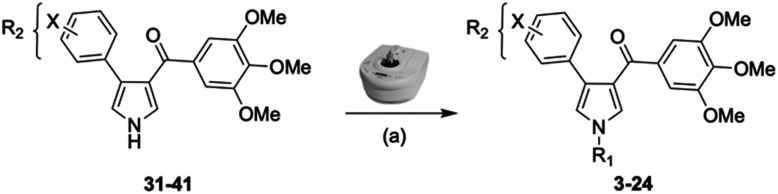
Reagents and conditions: (a) appropriate halogenated-pyridine or pyrimidine, CuI, Cs_2_CO_3_, 1,10-phenanthroline, dry 1,4-dioxane, MW irradiation, (closed vessel, 150 W, 180 °C, 40 min), yield: 22–89%. *R*_1_: see Table 1. X: 31, 4-methyl; 32, 4-methoxy; 33, 4-trifluoromethyl; 34: 4-chloro; 35: 4-*N*,*N*-dimethylamino; 36: 3,5-dimethoxy; 37: 3,4,5-trimethoxy; 38: 4-fluoro; 39: 4-nitro; 40: *R*_2_ = (*E*)-4-styryl: 4-(piperidin-1-yl).

In our continuous effort to reduce the environmental impacts of our synthetic studies by use of MW-assisted synthesis,^8,9^ we focused first on the synthesis of pyrrole compounds 3–24 through the protocol outlined in Scheme 1. Building upon the well-documented beneficial effect of MW irradiation on Cu(i)-catalyzed Ullmann-type coupling reactions,^10^ we employed this methodology to functionalize with different halogenated heterocycles the *N*-1-position of pyrrole intermediates 31–41 previously synthesized *via* a reported van Leusen cyclization reaction.^11^ Using the appropriate halogenated pyridine or pyrimidine, the coupling reaction was carried out in the presence of cesium carbonate, 1,10-phenanthroline in dry 1,4-dioxane under MW irradiation conditions (150 W, 180 °C for 40 min) resulting in significantly improved reaction profiles. The desired products 3–24 were obtained in significantly higher yields, up to 89%, with short reaction times and notable reduction in solvent consumption during both the reaction phase and the purification steps.

#### 2.2.2. Synthesis of 25–30

**Scheme 2:**
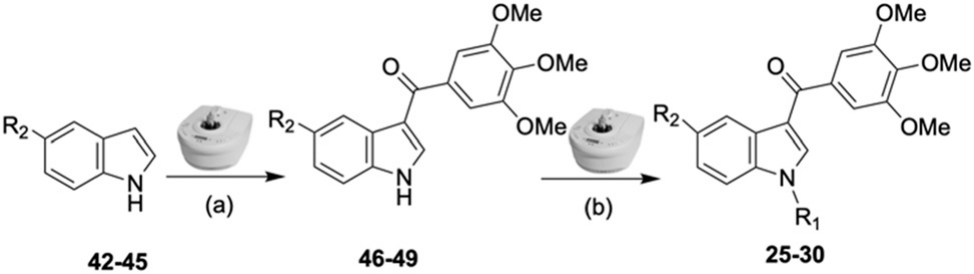
Reagents and conditions: (a) 3,4,5-trimethoxybenzoyl chloride, AlCl_3_, 1,2-dichloroethane, MW irradiation (closed vessel, 150 W, 110 °C, 4 min), yield: 41–96%; (b) 2-bromopyridine, 3-bromopyridine or 2-bromopyrimidine, CuI, Cs_2_CO_3_, 1,10-phenanthroline, dry 1,4-dioxane, MW irradiation (closed vessel, 150 W, 180 °C, 40 min), yield: 29–81%. *R*_1_: see Table 2. *R*_2_: 42, 46: Cl; 43, 47: F; 44, 48: CN; 45, 49: OMe.

For the synthesis of indole compounds 25–30, the MW-assisted protocol was systematically applied to each step of the synthetic route, as depicted in Scheme 2, allowing the entire sequence to be conducted in a more sustainable and resource-efficient manner. Indole intermediates 46–49 were synthesized from 5-substituted-1*H*-indoles 42–45 which underwent regioselective Friedel–Crafts acylation at the C3 position using 3,4,5-trimethoxybenzoyl chloride in the presence of anhydrous aluminum chloride under MW conditions. This step yielded the corresponding acyl-intermediates 46–49 with excellent regioselectivity and efficiency (yield up to 96%). The latter intermediates were subsequently subjected to a Cu(i)-catalyzed Ullmann-type coupling reaction with either 2-bromopyridine, 3-bromopyridine, or 2-bromopyrimidine, again utilizing MW irradiation conditions, to obtain the desired compounds 25–30.

Comparing the synthesis of 3–30 with our previous protocols,^12^ the MW-assisted synthesis improved significantly average yields and reduced dramatically reaction times in both the pyrrole and indole series. For example, our previously reported procedure for the synthesis of analogs of (1-phenyl-4-(aryl)-1*H*-pyrrol-3-yl)(3,4,5-trimethoxy-phenyl)methanone averaged a 22% yield, while by the procedures described here the average yield increased to 60% for compounds 3–24 and 59% for compounds 25–30 (Chart S1, ESI†). The adopted MW heating throughout the entire synthetic sequence resulted in considerable improvements in reaction rates, yields, and solvent economy compared to classic protocols, accelerating the overall workflow and, thereby, facilitating faster access to novel heterocyclic compounds of interest in medicinal chemistry.

## 3. Biology

### 3.1. Tubulin polymerization and colchicine binding

Most pyrrole derivatives inhibited tubulin assembly with IC_50_'s ranging from 0.19 (4) to 3.4 μM (13), while compounds 12 and 17 did not inhibit assembly even at 20 μM (Table 1). The indole derivatives inhibited assembly in a range from 0.38 (30) to 1.5 μM (25, 26) (Table 2). In this assay, combretastatin A-4 (CA4) yielded IC_50_'s of 0.37–0.69 μM. Compounds 4, 7 and 30 inhibited the binding of [^3^H]colchicine to tubulin by 78, 72 and 71%, respectively, with tubulin, [^3^H]colchicine and inhibitor at 1, 5 and 5 μM, respectively. Compound 4 at 1 μM inhibited the binding of [^3^H]colchicine by 76%. Inhibition of [^3^H]colchicine binding by reference CA4 was 98% at 5 μM and 78% at 1 μM.

**Table 1:** Inhibition of tubulin polymerization, growth of MCF-7 human breast carcinoma cells and [^3^H]colchcine binding to tubulin by compounds 3–24^a^

Cpd	*R*_1_	*R*_2_	Tubulin^b^ IC_50_ ± SD (μM)	MCF-7^c^ IC_50_ ± SD (nM)	Colch. binding^d^ (% ± SD)
3	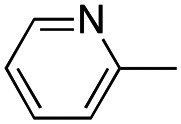	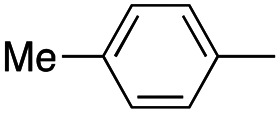	0.50 ± 0.1	1500 ± 200	59 ± 0.9
4	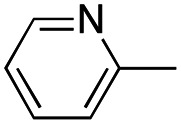	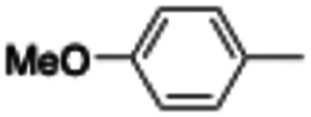	0.19 ± 0.006	9.6 ± 0.8	78 ± 1
5	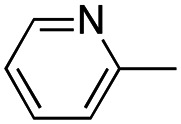	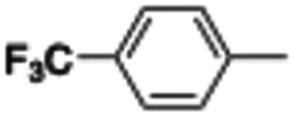	2.0 ± 0.1	652 ± 12.6	3.2 ± 5
6	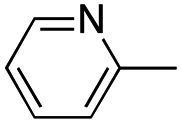	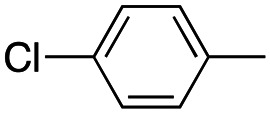	0.52 ± 0.005	340 ± 60	66 ± 3
7	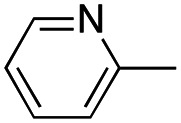	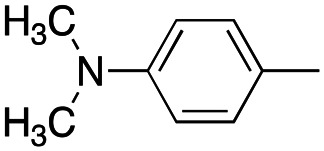	0.43 ± 0.04	120 ± 40	72 ± 0.2
8	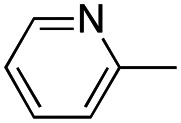	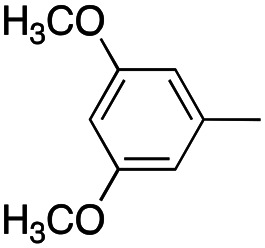	1.6 ± 0.2	2200 ± 400	21 ± 5
9	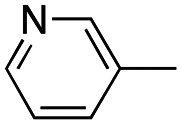	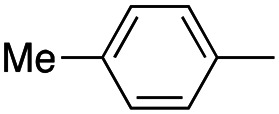	1.9 ± 0.2	670 ± 200	20 ± 5
10	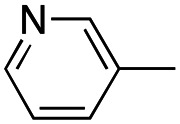	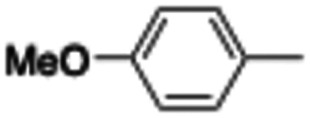	0.84 ± 0.02	758 ± 9.3	29 ± 0.8
11	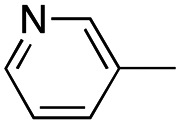	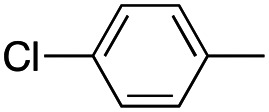	1.5 ± 0.2	1500 ± 400	29 ± 4
12	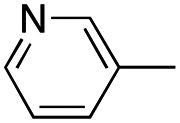	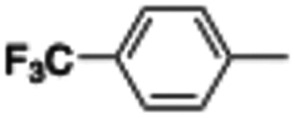	>20	3900 ± 70	nd
13	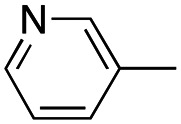	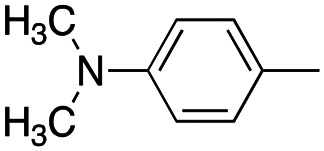	3.4 ± 0.3	1000 ± 100	9.6 ± 4
14	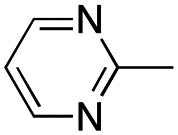	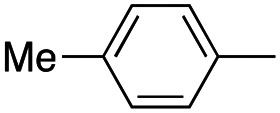	1.3 ± 0.04	170 ± 50	54 ± 3
15	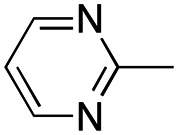	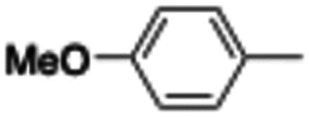	1.1 ± 0.1	95 ± 20	65 ± 5
16	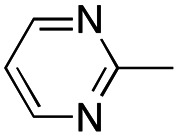	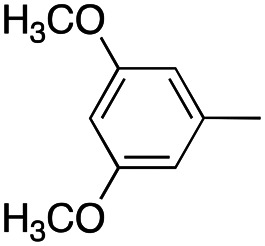	1.1 ± 0.06	780 ± 40	25 ± 5
17	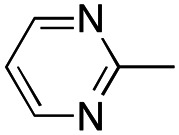	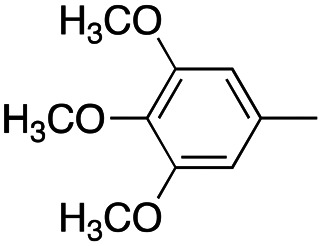	>20	>5000	nd
18	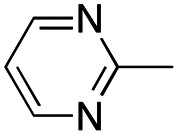	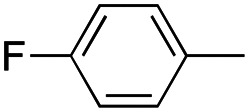	2.4 ± 0.08	150 ± 7	70 ± 1
19	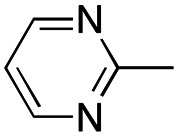	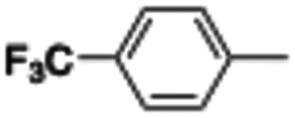	2.8 ± 0.1	530 ± 00	18 ± 4
20	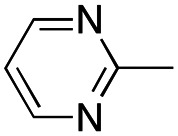	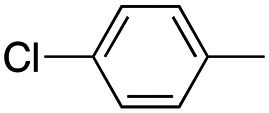	0.76 ± 0.1	68 ± 10	69 ± 4
21	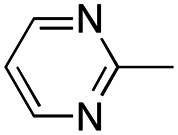	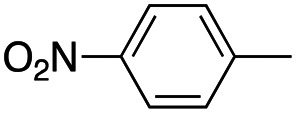	1.3 ± 0.2	450 ± 100	34 ± 1
22	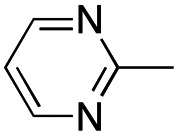	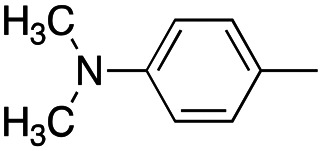	0.68 ± 0.1	10 ± 0	80 ± 0.2
23	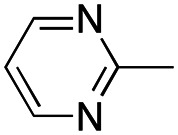	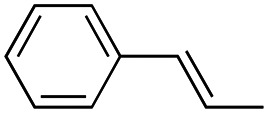	0.77 ± 0.03	270 ± 10	65 ± 5
24	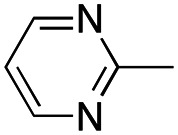	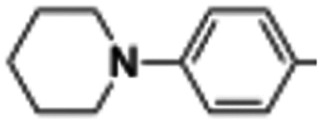	0.63 ± 0.04	112 ± 7.7	35 ± 2

a Experiments were performed in duplicate or triplicate.

b Inhibition of tubulin polymerization: tubulin was at 10 μM. CA4 yielded IC_50_'s of 0.37–0.69 μM in this assay.

c MCF-7 cell growth inhibition.

d Inhibition of [^3^H]colchicine binding: tubulin, [^3^H]colchicine, inhibitor were at 1, 5 and 5 μM. CA4 yielded 98% inhibition at 5 μM.

**Table 2:** Inhibition of tubulin polymerization, growth of MCF-7 human breast carcinoma cells and [^3^H]colchcine binding to tubulin by compounds 25–30^a^

Cpd	*R*_1_	*R*_2_	Tubulin^b^ IC_50_ ± SD (μM)	MCF-7^c^ IC_50_ ± SD (nM)	Colch. binding^d^ (% ± SD)
25	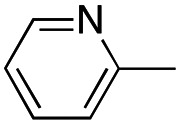	Cl	1.5 ± 0.3	41.7 ± 2.6	36 ± 4
26	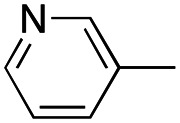	Cl	1.5 ± 0.3	306 ± 13.4	23 ± 4
27	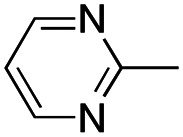	Cl	1.0 ± 0.09	77 ± 10	34 ± 5
28	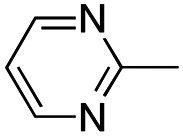	F	0.59 ± 0.1	73 ± 4	45 ± 0.8
29	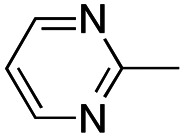	CN	1.3 ± 0.07	230 ± 7	42 ± 5
30	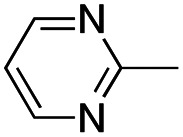	OCH_3_	0.38 ± 0.04	220 ± 60	71 ± 5

a Experiments were performed in duplicate or triplicate.

b Inhibition of tubulin polymerization; tubulin was at 10 μM. CA4 yielded IC_50_'s of 0.37–0.69 μM in this assay.

c MCF-7 cell growth inhibition.

d Inhibition of [^3^H]colchicine binding: tubulin, [^3^H]colchicine, inhibitor were at 1, 5 and 5 μM. CA4 yielded 98% inhibition at 5 μM.

### 3.2. Cancer cell growth

Breast (BC), colorectal and pancreatic cancers are among the 5 deadliest cancers.^13^ BC is the most common cancer diagnosed in women, affecting 1 in 8 women often after menopause, in the U.S.^14,15^ Five-year survival among women with stage II, III or IV BC are 93, 72 and 22%, respectively. Surgery, radiation, chemotherapy and immunotherapy are used to treat BC, depending on tumor stage and type.^16^

#### MCF-7 breast cancer cells

We introduced electron-withdrawing, electron-donating, and sterically bulky groups at position 4 of the 4-phenyl substituent on the pyrrole ring, and pyridine-2-yl, pyridine-3-yl and pyrimidin-2-yl rings at position 1 of the pyrrole (compounds 3–24, Table 1). We did not synthesize 1-(pyridin-4-yl)pyrrole derivatives since these compounds were previously found to be weak inhibitors of the MCF-7 cancer cells.^6^ As inhibitors of MCF-7 cancer cell growth, pyridine-2-yl (3–8) and pyrimidin-2-yl derivatives (14–24) were generally superior to the pyridine-3-yl derivatives (9–13). In both the pyridine-2-yl and pyrimidin-2-yl series, 4-chloro- (6) and (20), and 4-dimethylaminophenyl (7) and (22) were valuable substituents for inhibition of MCF-7 cell growth. Among pyrrole compounds, 4, with IC_50_ of 9.6 nM, and 22, with IC_50_ of 10 nM were the strongest inhibitors of the MCF-7 cell growth (Table 1).

Having in mind the excellent inhibition of tubulin assembly exhibited by indole derivatives,^17^ we joined the 4-aryl ring with the pyrrole nucleus to form an indole nucleus and maintained the same pyridyne-2-yl, pyridine-3-yl and pyrimidyn-2-yl heterocycles at position 1 (compounds 25–30, Table 2). As inhibitors of MCF-7 cancer cell growth, both 5-chloroindoles 25 and 27, bearing the pyrimidyn-2-yl and pyrimidyn-2-yl group at position 1, were effective inhibitors, with IC_50_'s of 42 and 77 nM, respectively. Compound 27 was almost equipotent with the 5-fluoroindole analog 28.

**Chart 2:**
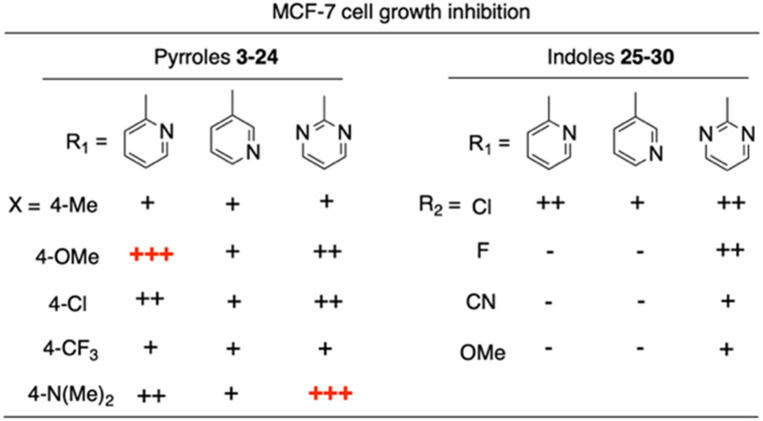
SAR summary of MCF-7 cell growth inhibition: +, weak; ++ strong; +++ strongest; −, no data. See Charts 1 and 2 for structural formulas 3–24 and 25–30.

A SAR summary of the MCF-7 cancer cell growth inhibitions by 3–30 is depicted in Chart 2. The inhibition of MCF-7 cancer cell growth (IC_50_ values) correlated with [^3^H]colchicine binding inhibition (% values) (Fig. S1, ESI†).

#### Colorectal, pancreatic and T lymphocyte cancer cells

Compound 4, the most potent of the compounds against the MCF-7 cells, was examined as inhibitor of HCT116 (colorectal cancer), BX-PC3 (pancreatic) and Jurkat (T lymphocyte) cancer cell growth. The risk of developing colorectal cancer (CRC) depends on genetic factors, adenomatous polyposis and Lynch syndrome (hereditary nonpolyposis CRC),^18,19^ but most CRCs are sporadic, most likely being related to age and individual lifestyle.^20,21^ Stages I to III CRC and early stage non-metastatic CRC are treated with chemotherapy and/or radiotherapy, whereas patients with metastatic CRC (stage IV) are usually treated with systemic chemotherapy.^22^ Chronic pancreatitis (long-lasting inflammation of the pancreas) may develop into pancreatic cancer (PC). The extremely low prognosis is attributable to its high aggressiveness, invasiveness, late diagnosis, and lack of effective therapies. Early diagnosis of this cancer is very difficult since the pancreas is located deep inside the body. Thus, when symptoms develop, the cancer has usually already spread. Surgical resection is the only current option for a cure, but only 20% of PC is surgically resectable at the time of diagnosis.^23^ Just 6% of people diagnosed with PC are alive 5 years later. T-cell acute lymphoblastic leukemia (T-ALL) represents 15% of pediatric and 25% of adult newly diagnosed lymphoblastic leukemia and is characterized by infiltration of bone marrow by immature T-cell lymphoblasts. The five-year survival in children is superior to 80%, but less than 50% in adults and even less in adults over the age of 50.^24^ Compound 4 induced a dose-dependent inhibition of cell viability of HCT116, BX-PC3 and Jurkat T at low nanomolar concentrations, with IC_50_ values of 18, 17 and 41 nM, respectively (Table 3, Fig. S2 and S3, ESI†). Thus, compound 4 shows the potential to be used for the treatment of deadly breast, colorectal and pancreatic cancers and for the treatment of highly aggressive T-ALL.

**Table 3:** Inhibition of HCT116, BX-PC3 and Jurkat T carcinoma cells by compound 4^a^*^,^*^b^

Cpd	HCT116	BX-PC3	Jurkat
	IC_50_^c^ (nM)	IC_50_^d^ (nM)	IC_50_^e^ (nM)
4	28	17	44

a Experiments were performed in duplicate or triplicate.

b Standard deviations were in the range of ±5% of the reported IC_50_ values.

c Inhibition of growth of HCT116 colon cancer cells. Incubation time was 48 h.

d Inhibition of growth of BX-PC3 pancreatic cancer cells. Incubation time was 48 h.

e Inhibition of growth of Jurkat T lymphocyte cancer cells. Incubation time was 96 h.

#### Effect of 4 on 3D cultures

**Fig. 1:**
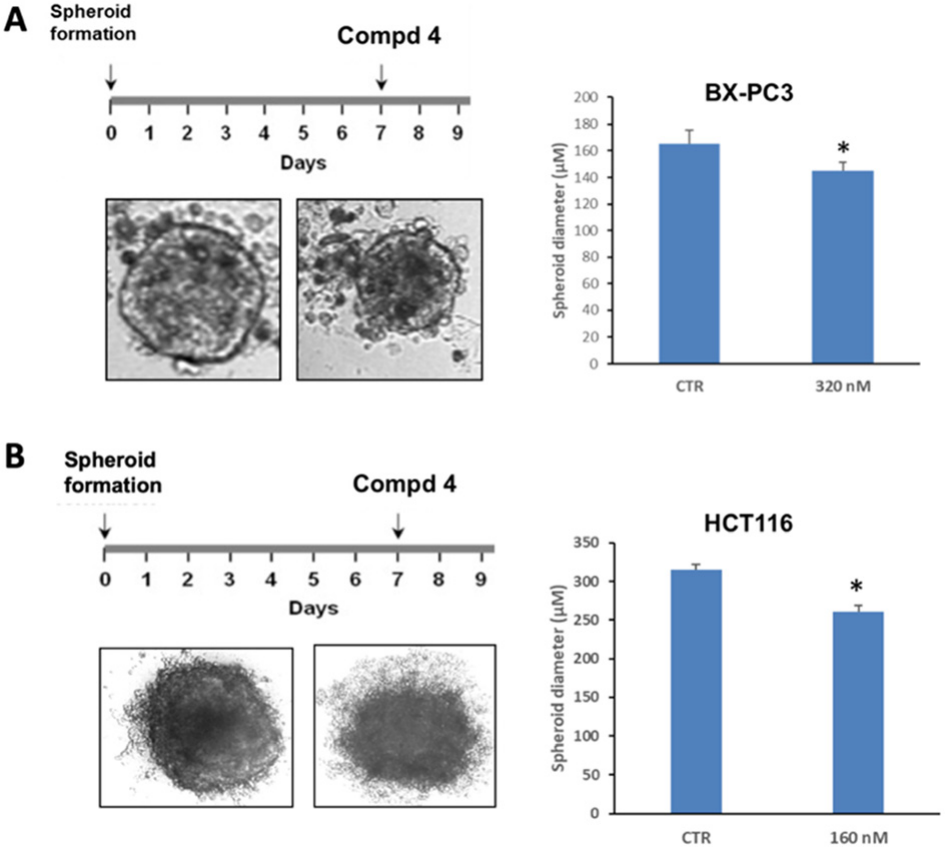
Effect of 4 on 3D culture from BX-PC3 and HCT116 cells. (A) Representative images of 3D culture obtained from BX-PC3 seeded into ultra-low attachment plates, cultured for 7 days to obtain spheroids and treated with 320 nM 4 for 48 h. Spheroid diameter measurement was obtained with Zen software in BX-PC3 cells. (B) Representative images of 3D culture obtained from HCT116 cells seeded into ultra-low attachment plates, cultured for 7 days to obtain spheroids and treated with 160 nM 4 for 48 h. Spheroid diameter measurement at 160 nM was obtained with Zen software. Bars are averaged normalized values ± SD of three independent experiments: (*) *p* < 0.05.

The antitumor properties of compound 4 were further validated on 3D cultures derived from BX-PC3 and HCT116 cells. Tumor-derived spheroids are floating spheres that serve as surrogate systems to evaluate cancer stem cell (CSC) related characteristics of solid tumors *in vitro*. Because CSCs are associated with tumorigenicity or chemoresistance, the activity in tumor-derived spheroids may usefully characterize the antitumor profile.^25^Fig. 1 shows micrographs of the spheroids obtained after 7 days in appropriate culture medium and then treated with 320 nM and 160 nM 4 for 48 h in BX-PC3 and HCT116 cell lines, respectively. In the BX-PC3 cell line, compound 4 at 320 nM caused significant reduction of spheroid diameter; in the HCT116 cell line, a significant reduction of spheroid diameter was observed at 160 nM. Based on the results obtained, we can say that at the highest doses of 4 in both cell lines the morphology of treated spheroids was altered, with progressively less defined shapes and significantly reduced diameter after a 48 h treatment.

## 4. Computational studies

**Fig. 2:**
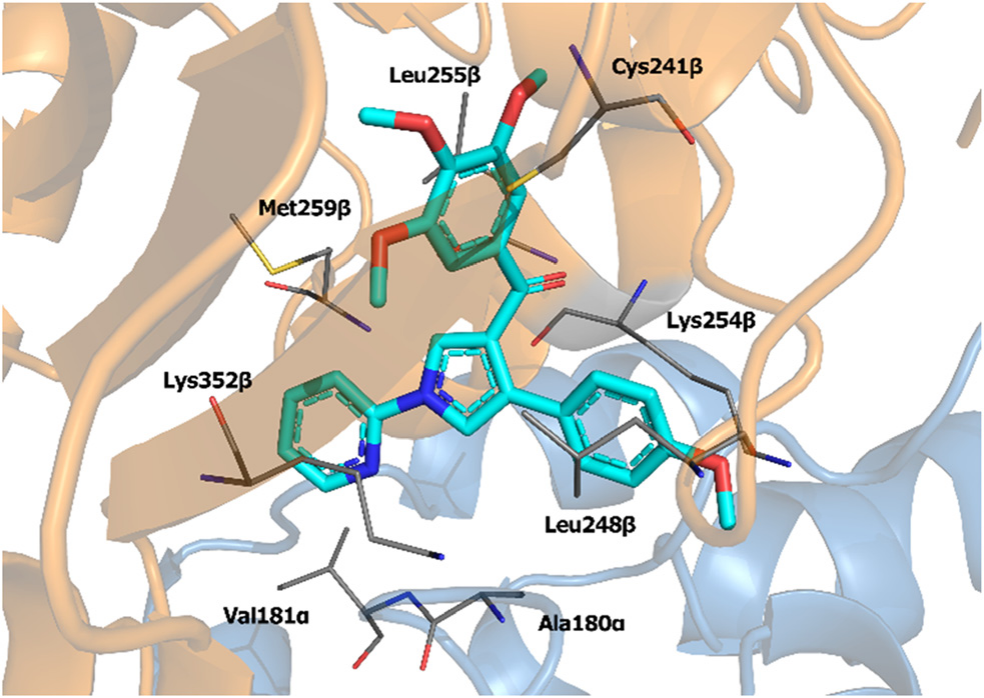
Proposed binding mode for compound 4. Protein is depicted as a cartoon, orange for β- and light blue for α-subunits. Residue involved in interactions are depicted as grey lines.

The binding mode of compound 4 was investigated through molecular docking experiments. The compound was docked in the colchicine site based on its strong inhibition of [^3^H]colchicine binding to tubulin. Analysis of the docking results revealed key pharmacophoric interactions: (i) the trimethoxyphenyl moiety engaged in hydrophobic contacts with Leu255β; (ii) a polar interaction was observed between the side chain of Cys241β and the closer trimethoxy group; (iii) the pyrimidin-2-yl ring at the pyrrole nitrogen formed hydrophobic interactions with the side chains of Met259β, Lys352β, Ala180α and Val181α; (iv) the 4-methoxyphenyl at position 4 of the pyrrole extended toward the solvent-exposed region of the pocket forming hydrophobic contacts with the Lys254β side chain (Fig. 2).

Notably, the docking studies also suggested alternative binding poses in which the pyrrole ring was rotated about 180 degrees. In this binding conformation, the *R*_1_ substituent faced the solvent-exposed area, while the *R*_2_ substituent pointed toward the bottom of the pocket. However, this latter binding mode received lower scores in MM-GBSA calculations, indicating a less favorable interaction profile.

To further investigate the proposed binding mode, a 200 ns molecular dynamics simulation was performed. Trajectory analysis revealed that the described interactions remained stable throughout the simulation (Fig. S4, ESI†). Additionally, a new hydrogen bond was observed between the ketone group and the side chain of Asp251β, with a formation frequency of 80% (Fig. S5, ESI†).

To assess drug-like behavior of compound 4, common ADMET descriptors were computed. The studied derivative (Table S2†) complies with both Lipinski's and Veber's rules, suggesting it is likely to be bioavailable by oral administration and with reduced likelihood of toxicity.^26^

## 5. Experimental

### 5.1. Chemistry

#### 5.1.1. General information

All reagents and solvents were handled according to the material safety data sheet of the supplier and used as purchased without further purification. Anhydrous organic solvents were purchased from Merck and stored under an inert atmosphere according to standard procedures. Evaporation of solvents was carried out on a Büchi Rotavapor R-210 equipped with a Büchi V-850 vacuum controller and a Büchi V-700 vacuum pump. Column chromatography was performed on columns packed with silica gel from Macherey-Nagel (70–230 mesh). Silica gel thin-layer chromatography (TLC) cards from Macherey-Nagel (silica gel-precoated aluminum cards with a fluorescent indicator visualizable at 254 nm) were used for TLC. Developed plates were visualized with a Spectroline ENF 260C/FE UV apparatus. Melting points (mp) were determined on a Stuart Scientific SMP1 apparatus and are uncorrected. Infrared (IR) spectra were recorded on a PerkinElmer Spectrum 100 FT-IR spectrophotometer equipped with a universal attenuated total reflectance accessory, and IR data were acquired and processed by PerkinElmer Spectrum 10.03.00.0069 software. Band position and absorption ranges are given in cm^−1^. Proton nuclear magnetic resonance (^1^H NMR) spectra were recorded with a Bruker Avance (400 MHz) spectrometer in the indicated solvent. Carbon-13 nuclear magnetic resonance (^13^C NMR) spectra were recorded with a Bruker Avance (100 MHz) spectrometer in the indicated solvent. The corresponding ^1^H and ^13^C NMR fid files were processed by MestreLab Research SL MestreReNova 6.2.1-769 software. Chemical shifts of ^1^H and ^13^C NMR are expressed in *δ* units (ppm) from tetramethylsilane.

Compound purity was checked by high performance liquid chromatography (HPLC). Purity of tested compounds was found to be >95%. The HPLC system used (Thermo Fisher Scientific Inc. Dionex UltiMate 3000) consisted of an SR-3000 solvent rack, an LPG-3400SD quaternary analytical pump, a TCC-3000SD column compartment, a DAD-3000 diode array detector, and an analytical manual injection valve with a 20 μL loop. Samples were dissolved in acetonitrile (1 mg mL^−1^). HPLC analysis was performed by using a Thermo Fisher Scientific Inc. Acclaim 120 C18 column (5 μm, 4.6 mm × 250 mm), at 25 ± 1 °C with an appropriate solvent gradient (acetonitrile/water), flow rate of 1.0 mL min^−1^, and signal detector at 206, 230, 254, and 365 nm were used. Chromatographic data were acquired and processed by Thermo Fisher Scientific Inc. Chromeleon 6.80 SR15 Build 4656 software.

#### 5.1.2. Procedure A. Synthesis of pyrroles 3–24 and indoles 25–30

##### (1-(Pyridin-2-yl)-4-(4-tolyl)-1*H*-pyrrol-3-yl)(3,4,5-trimethoxyphenyl)methanone (3)

To a MW reaction vial equipped with a magnetic stirring bar, a mixture of (4-(4-tolyl)-1*H*-pyrrol-3-yl)(3,4,5-trimethoxyphenyl)methanone (31) (500.0 mg, 1.423 mmol), 2-bromopyridine (0.176 mL, 1.845 mmol), copper(i) iodide (136.0 mg, 0.712 mmol), cesium carbonate (696.0 mg, 2.135 mmol), and 1,10-phenanthroline (25.0 mg, 0.142 mmol) in 1,4-dioxane (9 mL) was added. The vial was placed in the MW reactor in closed-vessel mode, and heated for 40 min (150 W, internal temperature 180 °C, ramp time of 5 min, *P*_max_ 200 psi). Upon completion, the reaction mixture was cooled to room temperature, diluted with water and extracted with ethyl acetate (2 × 15 mL). The combined organic layers were washed with brine (2 × 15 mL), dried over anhydrous sodium sulfate, filtered and concentrated *in vacuo* to give a crude product. The residue was purified by flash chromatography (silica gel, eluting with cyclohexane : ethyl acetate = 7 : 3) to furnish the desired compound 3 (475.0 mg, 1.108 mmol; 78%), mp 148–150 °C (from ethanol). ^1^H NMR (400 MHz, DMSO-*d*_6_) *δ* 8.50 (d, *J* = 4.9 Hz, 1H), 8.18 (d, *J* = 2.3 Hz, 1H), 8.00–7.95 (m, 3H), 7.35 (dt, *J* = 6.4, 3.4 Hz, 1H), 7.29 (d, *J* = 7.7 Hz, 2H), 7.12 (s, 2H), 7.10 (d, *J* = 7.4 Hz, 2H), 3.77 (s, 6H), 3.73 (s, 3H), 2.28 (s, 3H) ppm. ^13^C NMR (101 MHz, DMSO-*d*_6_) *δ* 189.3, 152.5, 149.6, 148.7, 141.1, 139.6, 135.5, 133.9, 131.2, 128.6, 128.1, 127.7, 125.2, 123.3, 121.9, 118.2, 112.3, 106.9, 60.1, 55.9, 20.7 ppm. IR: ν 1443, 1580, 1636 and 2939 cm^−1^.

##### (4-(4-Methoxyphenyl)-1-(pyridin-2-yl)-1*H*-pyrrol-3-yl)(3,4,5-trimethoxyphenyl)methanone (4)

Was synthesized according to general procedure A, starting from (4-(4-methoxyphenyl)-1*H*-pyrrol-3-yl)(3,4,5-trimethoxyphenyl)methanone (32) (300.0 mg, 0.817 mmol) and 2-bromopyridine (0.101 mL, 1.062 mmol). Yield: 74%. Mp 129–130 °C (from ethanol). ^1^H NMR (400 MHz, DMSO-*d*_6_) *δ* 8.50–8.48 (m, 1H), 8.17 (d, *J* = 2.4 Hz, 1H), 8.01–7.96 (m, 2H), 7.95 (d, *J* = 2.3 Hz, 1H), 7.37–7.32 (m, 3H), 7.12 (s, 2H), 6.87 (d, *J* = 8.7 Hz, 2H), 3.78 (s, 6H), 3.73 (s, 3H), 3.74 (s, 3H) ppm. ^13^C NMR (101 MHz, DMSO-*d*_6_) *δ* 189.3, 158.0, 152.5, 149.6, 148.7, 141.1, 139.6, 134.0, 129.4, 127.5, 126.5, 125.2, 123.2, 121.9, 117.9, 113.4, 112.3, 106.9, 60.1, 55.9, 55.1 ppm. IR: *ν* 1441, 1581, 1645, 2936 and 3129 cm^−1^.

##### (1-(Pyridin-2-yl)-4-(4-(trifluoromethyl)phenyl)-1*H*-pyrrol-3-yl)(3,4,5-trimethoxyphenyl)methanone (5)

Was synthesized according to general procedure A, starting from (4-(4-(trifluoromethyl)phenyl)-1*H*-pyrrol-3-yl)(3,4,5-trimethoxyphenyl)methanone (33) (405.4 mg, 0.987 mmol) and 2-bromopyridine (0.122 mL, 1.283 mmol). Yield: 22%. Mp 166–168 °C (from ethanol). ^1^H NMR (400 MHz, DMSO-*d*_6_) *δ* 8.52 (dt, *J* = 4.8, 1.4 Hz, 1H), 8.26 (d, *J* = 2.3 Hz, 1H), 8.16 (d, *J* = 2.3 Hz, 1H), 8.04–7.99 (m, 2H), 7.65 (q, *J* = 8.4 Hz, 4H), 7.42–7.36 (m, 1H), 7.15 (s, 2H), 3.79 (s, 6H), 3.74 (s, 3H) ppm. ^13^C NMR (101 MHz, DMSO-*d*_6_) *δ* 189.0, 152.5, 149.4, 148.7, 141.2, 139.7, 138.4, 133.8, 128.8, 126.8 (q, ^2^*J*_C–F_ = 30.7 Hz), 125.2 (q, ^1^*J*_C–F_ = 272.3 Hz), 124.8 (q, ^3^*J*_C–F_ = 3.5 Hz), 123.1, 122.2, 119.7, 112.5, 106.9, 60.1, 56.0 ppm. IR: *ν* 1324, 1500, 1580, 1645, 2948 and 3138 cm^−1^.

##### (4-(4-Chlorophenyl)-1-(pyridin-2-yl)-1*H*-pyrrol-3-yl)(3,4,5-trimethoxyphenyl)methanone (6)

Was synthesized according to general procedure A, starting from (4-(4-chlorophenyl)-1*H*-pyrrol-3-yl)(3,4,5-trimethoxyphenyl)methanone (34) (400.0 mg, 1.076 mmol) and 2-bromopyridine (0.133 mL, 1.399 mmol). Yield: 77%. Mp 158–160 °C (from ethanol). ^1^H NMR (400 MHz, DMSO-*d*_6_) *δ* 8.50 (dd, *J* = 4.8, 1.5 Hz, 1H), 8.22 (d, *J* = 2.3 Hz, 1H), 8.06 (d, *J* = 2.3 Hz, 1H), 8.02–7.96 (m, 2H), 7.43 (d, *J* = 8.3 Hz, 2H), 7.38–7.35 (m, 1H), 7.35 (d, *J* = 8.6 Hz, 2H), 7.12 (s, 2H), 3.79 (s, 6H), 3.74 (s, 3H) ppm. ^13^C NMR (101 MHz, DMSO-*d*_6_) *δ* 189.1, 152.5, 149.5, 148.7, 141.1, 139.7, 133.9, 133.0, 131.0, 130.0, 127.9, 126.4, 125.7, 123.1, 122.1, 118.9, 112.4, 106.9, 60.1, 56.0 ppm. IR: *ν* 771, 1580, 1643, 2944 and 3135 cm^−1^.

##### (4-(4-(Dimethylamino)phenyl)-1-(pyridin-2-yl)-1*H*-pyrrol-3-yl)(3,4,5-trimethoxyphenyl)methanone (7)

Was synthesized according to general procedure A, starting from (4-(4-(dimethylamino)phenyl)-1*H*-pyrrol-3-yl)(3,4,5-trimethoxyphenyl)methanone (35) (500.0 mg, 1.423 mmol) and 2-bromopyridine (0.175 mL, 1.845 mmol). Yield: 43%. Mp 155–157 °C (from ethanol). ^1^H NMR (400 MHz, DMSO-*d*_6_) *δ* 8.49–8.46 (m, 1H), 8.14 (d, *J* = 2.4 Hz, 1H), 8.00–7.93 (m, 2H), 7.88 (d, *J* = 2.4 Hz, 1H), 7.34 (t, *J* = 5.9 Hz, 1H), 7.24 (d, *J* = 8.8 Hz, 2H), 7.12 (s, 2H), 6.65 (d, *J* = 8.8 Hz, 2H), 3.77 (s, 6H), 3.73 (s, 3H), 2.88 (s, 6H) ppm. ^13^C NMR (101 MHz, DMSO-*d*_6_) *δ* 189.4, 152.4, 149.7, 149.2, 148.7, 141.0, 139.5, 134.0, 128.9, 128.0, 124.9, 123.2, 121.9, 121.7, 117.1, 112.2, 112.0, 106.9, 60.1, 55.9, 40.2 ppm. IR: *ν* 1630, 1734, 2933, 2997 and 3750 cm^−1^.

##### (4-(3,5-Dimethoxyphenyl)-1-(pyridin-2-yl)-1*H*-pyrrol-3-yl)(3,4,5-trimethoxyphenyl)methanone (8)

Was synthesized according to general procedure A, starting from (4-(3,5-dimethoxyphenyl)-1*H*-pyrrol-3-yl)(3,4,5-trimethoxyphenyl)methanone (36) (194.0 mg, 0.492 mmol) and 2-bromopyridine (0.060 mL, 0.639 mmol). Yield: 57%. Mp 146–148 °C (from ethanol). ^1^H NMR (400 MHz, DMSO-*d*_6_) *δ* 8.51–8.49 (m, 1H), 8.20 (d, *J* = 2.4 Hz, 1H), 8.05 (d, *J* = 2.4 Hz, 1H), 8.02–7.96 (m, 2H), 7.36 (t, *J* = 5.8 Hz, 1H), 7.08 (s, 2H), 6.53 (d, *J* = 2.3 Hz, 2H), 6.35 (d, *J* = 2.4 Hz, 1H), 3.75 (s, 6H), 3.71 (s, 3H), 3.68 (s, 6H) ppm. ^13^C NMR (101 MHz, DMSO-*d*_6_) *δ* 189.5, 160.1, 152.5, 149.6, 148.7, 141.1, 139.7, 136.0, 134.0, 127.6, 125.0, 123.7, 122.1, 118.6, 112.4, 106.9, 106.4, 98.7, 60.1, 55.9, 55.1 ppm. IR: *ν* 1441, 1592, 1645, 2835, 2943 and 3135 cm^−1^.

##### (1-(Pyridin-3-yl)-4-(4-tolyl)-1*H*-pyrrol-3-yl)(3,4,5-trimethoxyphenyl)methanone (9)

Was synthesized according to general procedure A, starting from (4-(4-tolyl)-1*H*-pyrrol-3-yl)(3,4,5-trimethoxyphenyl)methanone (31) (287.1 mg, 0.817 mmol) and 3-bromopyridine (0.101 mL, 1.062 mmol). Yield: 60%. Mp 175–177 °C (from ethanol). ^1^H NMR (400 MHz, DMSO-*d*_6_) *δ* 9.07 (d, *J* = 3.3 Hz, 1H), 8.55 (d, *J* = 5.2 Hz, 1H), 8.23 (d, *J* = 8.5 Hz, 1H), 8.06–8.04 (m, 1H), 7.85–7.80 (m, 1H), 7.56–7.53 (m, 1H), 7.28 (d, *J* = 7.7 Hz, 2H), 7.13 (s, 2H), 7.10 (d, *J* = 7.8 Hz, 2H), 3.83–3.76 (m, 6H), 3.73 (s, 3H), 2.28 (s, 3H) ppm. ^13^C NMR (101 MHz, DMSO-*d*_6_) *δ* 189.1, 152.5, 147.4, 141.5, 141.1, 135.4, 135.3, 133.9, 131.2, 128.5, 128.2, 127.9, 127.5, 126.4, 124.3, 123.3, 119.8, 107.0, 60.1, 55.9, 20.7 ppm. IR: *ν* 1440, 1498, 1582, 1636, 2934 and 3124 cm^−1^.

##### (4-(4-Methoxyphenyl)-1-(pyridin-3-yl)-1*H*-pyrrol-3-yl)(3,4,5-trimethoxyphenyl)methanone (10)

Was synthesized according to general procedure A, starting from (4-(4-methoxyphenyl)-1*H*-pyrrol-3-yl)(3,4,5-trimethoxyphenyl)methanone (32) (300.0 mg, 0.817 mmol) and 3-bromopyridine (0.101 mL, 1.062 mmol). Yield: 62%. Mp 146–148 °C (from ethanol). ^1^H NMR (400 MHz, DMSO-*d*_6_) *δ* 9.07 (d, *J* = 2.8 Hz, 1H), 8.54 (dd, *J* = 4.7, 1.4 Hz, 1H), 8.22 (ddd, *J* = 8.3, 2.8, 1.4 Hz, 1H), 8.04 (d, *J* = 2.4 Hz, 1H), 7.77 (d, *J* = 2.4 Hz, 1H), 7.54 (dd, *J* = 8.3, 4.7 Hz, 1H), 7.33 (d, *J* = 8.7 Hz, 2H), 7.13 (s, 2H), 6.87 (d, *J* = 8.8 Hz, 2H), 3.79 (s, 6H), 3.74 (s, 3H), 3.73 (s, 3H) ppm. ^13^C NMR (101 MHz, DMSO-*d*_6_) *δ* 189.1, 158.0, 152.5, 147.4, 141.5, 141.1, 135.3, 133.9, 129.4, 127.7, 127.5, 126.5, 126.4, 124.3, 123.2, 119.5, 113.4, 107.0, 60.1, 55.9, 55.1 ppm. IR: *ν* 1500, 1579, 1637, 2947 and 3093 cm^−1^.

##### (4-(4-Chlorophenyl)-1-(pyridin-3-yl)-1*H*-pyrrol-3-yl)(3,4,5-trimethoxyphenyl)methanone (11)

Was synthesized according to general procedure A, starting from (4-(4-chlorophenyl)-1*H*-pyrrol-3-yl)(3,4,5-trimethoxyphenyl)methanone (34) (400.0 mg, 1.076 mmol) and 3-bromopyridine (0.135 mL, 1.399 mmol). Yield: 61%. Mp 164–166 °C (from ethanol). ^1^H NMR (400 MHz, DMSO-*d*_6_) *δ* 9.06 (d, *J* = 2.8 Hz, 1H), 8.55 (dd, *J* = 4.7, 1.3 Hz, 1H), 8.22 (ddd, *J* = 8.4, 2.8, 1.4 Hz, 1H), 8.09 (d, *J* = 2.4 Hz, 1H), 7.89 (d, *J* = 2.4 Hz, 1H), 7.55 (dd, *J* = 8.4, 4.7 Hz, 1H), 7.42 (d, *J* = 8.5 Hz, 2H), 7.35 (d, *J* = 8.5 Hz, 2H), 7.14 (s, 2H), 3.80 (s, 6H), 3.74 (s, 3H) ppm. ^13^C NMR (101 MHz, DMSO-*d*_6_) *δ* 189.0, 152.6, 147.6, 141.6, 141.2, 135.2, 133.9, 133.1, 131.0, 130.0, 127.9, 127.7, 127.0, 126.7, 124.4, 123.1, 120.6, 107.0, 60.2, 56.0 ppm. IR: *ν* 787, 1583, 1635 and 2938 cm^−1^.

##### (1-(Pyridin-3-yl)-4-(4-(trifluoromethyl)phenyl)-1*H*-pyrrol-3-yl)(3,4,5-trimethoxyphenyl)methanone (12)

Was synthesized according to general procedure A, starting from (4-(4-(trifluoromethyl)phenyl)-1*H*-pyrrol-3-yl)(3,4,5-trimethoxyphenyl)methanone (33) (400.0 mg, 0.987 mmol) and 3-bromopyridine (0.123 mL, 1.283 mmol). Yield: 47%. Mp 172–174 °C (from ethanol). ^1^H NMR (400 MHz, DMSO-*d*_6_) *δ* 9.09 (d, *J* = 2.7 Hz, 1H), 8.57 (d, *J* = 4.4 Hz, 1H), 8.26–8.23 (m, 1H), 8.14 (d, *J* = 2.4 Hz, 1H), 7.99 (d, *J* = 2.4 Hz, 1H), 7.64 (dd, *J* = 13.4, 8.4 Hz, 4H), 7.57 (dd, *J* = 8.3, 4.7 Hz, 1H), 7.17 (s, 2H), 3.81 (s, 6H), 3.74 (s, 3H) ppm. ^13^C NMR (101 MHz, DMSO-*d*_6_) *δ* 188.8, 152.6, 147.7, 141.7, 141.2, 138.5, 135.2, 133.7, 128.8, 127.8, 127.3, 126.6 (q, ^2^*J*_C–F_ = 31.7 Hz), 126.4, 125.2 (q, ^1^*J*_C–F_ = 272.3 Hz), 124.8 (q, ^3^*J*_C–F_ = 3.7 Hz), 124.3, 123.1, 121.4, 107.0, 60.1, 56.0 ppm. IR: *ν* 1165, 1582, 1631, 1735, 2938 and 3128 cm^−1^.

##### (4-(4-(Dimethylamino)phenyl)-1-(pyridin-3-yl)-1*H*-pyrrol-3-yl)(3,4,5-trimethoxyphenyl)methanone (13)

Was synthesized according to general procedure A, starting from (4-(4-(dimethylamino)phenyl)-1*H*-pyrrol-3-yl)(3,4,5-trimethoxyphenyl)methanone (35) (500.0 mg, 1.314 mmol) and 3-bromopyridine (0.164 mL, 1.708 mmol). Yield: 43%. Mp 173–175 °C (from ethanol). ^1^H NMR (400 MHz, DMSO-*d*_6_) *δ* 9.06 (d, *J* = 2.8 Hz, 1H), 8.53 (dd, *J* = 4.7, 1.4 Hz, 1H), 8.21 (ddd, *J* = 8.4, 2.9, 1.4 Hz, 1H), 8.00 (d, *J* = 2.4 Hz, 1H), 7.70 (d, *J* = 2.4 Hz, 1H), 7.53 (dd, *J* = 8.3, 4.7 Hz, 1H), 7.23 (d, *J* = 8.7 Hz, 2H), 7.14 (s, 2H), 6.65 (d, *J* = 8.8 Hz, 2H), 3.78 (s, 6H), 3.73 (s, 3H), 2.87 (s, 6H) ppm. ^13^C NMR (101 MHz, DMSO-*d*_6_) *δ* 189.2, 152.5, 149.1, 147.3, 141.4, 141.0, 135.4, 134.0, 128.9, 128.3, 127.4, 126.1, 124.3, 123.2, 122.0, 118.7, 112.0, 107.0, 60.1, 55.9, 40.2 ppm. IR: *ν* 1645, 1734, 2834, 2935, 3005 and 3128 cm^−1^.

##### (1-(Pyrimidin-2-yl)-4-(4-tolyl)-1*H*-pyrrol-3-yl)(3,4,5-trimethoxyphenyl)methanone (14)

Was synthesized according to general procedure A, starting from (4-(4-tolyl)-1*H*-pyrrol-3-yl)(3,4,5-trimethoxyphenyl)methanone (31) (337.0 mg, 0.959 mmol) and 2-bromopyrimidine (198.2 mg, 1.247 mmol). Yield: 48%. Mp 161–163 °C (from ethanol). ^1^H NMR (400 MHz, DMSO-*d*_6_) *δ* 8.86 (d, *J* = 4.9 Hz, 2H), 8.12 (d, *J* = 2.3 Hz, 1H), 7.94 (d, *J* = 2.3 Hz, 1H), 7.47 (t, *J* = 4.9 Hz, 1H), 7.28 (d, *J* = 8.1 Hz, 2H), 7.11–7.09 (m, 4H), 3.76 (s, 6H), 3.73 (s, 3H), 2.28 (s, 3H) ppm. ^13^C NMR (101 MHz, DMSO-*d*_6_) *δ* 189.3, 159.4, 154.5, 152.5, 141.2, 135.8, 133.8, 130.7, 128.7, 128.1, 128.0, 125.4, 124.0, 119.3, 117.9, 106.9, 60.2, 56.0, 20.7 ppm. IR: *ν* 1497, 1571, 1636, 1724 and 2938 cm^−1^.

##### (4-(4-Methoxyphenyl)-1-(pyrimidin-2-yl)-1*H*-pyrrol-3-yl)(3,4,5-trimethoxyphenyl)methanone (15)

Was synthesized according to general procedure A, starting from (4-(4-methoxyphenyl)-1*H*-pyrrol-3-yl)(3,4,5-trimethoxyphenyl)methanone (32) (265.0 mg, 0.721 mmol) and 2-bromopyrimidine (149.1 mg, 0.937 mmol). Yield: 89%. Mp 70–72 °C (from ethanol). ^1^H NMR (400 MHz, DMSO-*d*_6_) *δ* 8.85 (d, *J* = 4.9 Hz, 2H), 8.12 (d, *J* = 2.3 Hz, 1H), 7.91 (d, *J* = 2.3 Hz, 1H), 7.46 (t, *J* = 4.9 Hz, 1H), 7.33 (d, *J* = 8.7 Hz, 2H), 7.11 (s, 2H), 6.87 (d, *J* = 8.7 Hz, 2H), 3.76 (s, 6H), 3.74 (s, 3H), 3.74 (s, 3H) ppm. ^13^C NMR (101 MHz, DMSO-*d*_6_) *δ* 189.3, 159.4, 158.2, 154.5, 152.5, 141.2, 133.8, 129.4, 127.9, 126.0, 125.4, 124.0, 119.3, 117.6, 113.5, 106.9, 60.2, 56.0, 55.1 ppm. IR: *ν* 1505, 1566, 1654, 2842, 2971 and 3007 cm^−1^.

##### (4-(3,5-Dimethoxyphenyl)-1-(pyrimidin-2-yl)-1*H*-pyrrol-3-yl)(3,4,5-trimethoxyphenyl)methanone (16)

Was synthesized according to general procedure A, starting from (4-(3,5-dimethoxyphenyl)-1*H*-pyrrol-3-yl)(3,4,5-trimethoxyphenyl)methanone (36) (500.0 mg, 1.258 mmol) and 2-bromopyrimidine (259.94 mg, 1.635 mmol). Yield: 70%. Mp 136–138 °C (from ethanol). ^1^H NMR (400 MHz, DMSO-*d*_6_) *δ* 8.87 (d, *J* = 4.8 Hz, 2H), 8.15 (d, *J* = 2.1 Hz, 1H), 8.03 (d, *J* = 2.3 Hz, 1H), 7.48 (t, *J* = 5.0 Hz, 1H), 7.07 (s, 2H), 6.52 (d, *J* = 2.3 Hz, 2H), 6.35 (t, *J* = 2.4 Hz, 1H), 3.74 (s, 6H), 3.72 (s, 3H), 3.68 (s, 6H) ppm. ^13^C NMR (101 MHz, DMSO-*d*_6_) *δ* 189.4, 160.1, 159.4, 154.5, 152.4, 141.2, 135.5, 133.8, 128.0, 125.1, 124.4, 119.4, 118.3, 106.9, 106.3, 99.0, 60.1, 55.9, 55.1 ppm. IR: *ν* 1498, 1571, 1642, 1719, 2829, 2934, 2998 and 3127 cm^−1^.

##### (1-(Pyrimidin-2-yl)-4-(3,4,5-trimethoxyphenyl)-1*H*-pyrrol-3-yl)(3,4,5-trimethoxyphenyl)methanone (17)

Was synthesized according to general procedure A, starting from (3,4,5-trimethoxyphenyl)(4-(3,4,5-trimethoxyphenyl)-1*H*-pyrrol-3-yl)methanone (37) (400.0 mg, 0.936 mmol) and 2-bromopyrimidine (193.50 mg, 1.217 mmol). Yield: 56%. Mp 75–77 °C (from ethanol). ^1^H NMR (400 MHz, DMSO-*d*_6_) *δ* 8.88 (d, *J* = 4.8 Hz, 2H), 8.19 (d, *J* = 2.4 Hz, 1H), 8.06 (d, *J* = 2.4 Hz, 1H), 7.49 (t, *J* = 4.8 Hz, 1H), 7.02 (s, 2H), 6.63 (s, 2H), 3.72 (s, 6H), 3.69 (s, 3H), 3.68 (s, 6H), 3.62 (s, 3H) ppm. ^13^C NMR (101 MHz, DMSO-*d*_6_) *δ* 189.6, 159.4, 154.5, 152.44, 152.38, 141.1, 136.4, 133.8, 129.3, 128.1, 125.3, 124.5, 119.5, 118.1, 106.9, 106.0, 60.1, 60.0, 55.9, 55.7 ppm. IR: *ν* 1499, 1583, 1633, 1724 and 2938 cm^−1^.

##### (4-(4-Fluorophenyl)-1-(pyrimidin-2-yl)-1*H*-pyrrol-3-yl)(3,4,5-trimethoxyphenyl)methanone (18)

Was synthesized according to general procedure A, starting from (4-(4-fluorophenyl)-1*H*-pyrrol-3-yl)(3,4,5-trimethoxyphenyl)methanone (38) (482.0 mg, 1.356 mmol) and 2-bromopyrimidine (280.30 mg, 1.763 mmol). Yield: 72%. Mp 176–177 °C (from ethanol). ^1^H NMR (400 MHz, DMSO-*d*_6_) *δ* 8.86 (d, *J* = 4.9 Hz, 1H), 8.15–8.14 (m, 1H), 7.98–7.97 (m, 1H), 7.49–7.42 (m, 3H), 7.15–7.12 (m, 2H), 7.11 (s, 2H), 3.77 (s, 6H), 3.74 (s, 3H) ppm. ^13^C NMR (101 MHz, DMSO-*d*_6_) *δ* 189.2, 160.0 (d, ^1^*J*_C–F_ = 244.2 Hz), 159.4, 154.5, 152.5, 141.2, 133.8, 130.2 (d, ^3^*J*_C–F_ = 8.5 Hz), 130.1 (d, ^4^*J*_C–F_ = 3.5 Hz), 127.1, 125.7, 123.9, 119.5, 118.4, 114.8 (d, ^2^*J*_C–F_ = 21.3 Hz), 106.9, 60.2, 56.0 ppm. IR: *ν* 1328, 1572, 1642, 2837 and 2944 cm^−1^.

##### (1-(Pyrimidin-2-yl)-4-(4-(trifluoromethyl)phenyl)-1*H*-pyrrol-3-yl)(3,4,5-trimethoxyphenyl)methanone (19)

Was synthesized according to general procedure A, starting from (4-(4-(trifluoromethyl)phenyl)-1*H*-pyrrol-3-yl)(3,4,5-trimethoxyphenyl)methanone (33) (300.0 mg, 0.740 mmol) and 2-bromopyrimidine (152.96 mg, 0.962 mmol). Yield: 62%. Mp 147–151 °C (from ethanol). ^1^H NMR (400 MHz, DMSO-*d*_6_) *δ* 8.88 (d, *J* = 4.8 Hz, 2H), 8.19 (d, *J* = 2.2 Hz, 1H), 8.12 (d, *J* = 2.2 Hz, 1H), 7.64 (dd, *J* = 8.5, 3.9 Hz, 4H), 7.50 (t, *J* = 4.8 Hz, 1H), 7.14 (s, 2H), 3.78 (s, 6H), 3.74 (s, 3H) ppm. ^13^C NMR (101 MHz, DMSO-*d*_6_) *δ* 189.0, 159.4, 154.4, 152.6, 141.4, 138.0, 133.7, 128.9, 126.7, 126.6 (q, ^2^*J*_C–F_ = 32.1 Hz), 126.1, 125.5 (q, ^1^*J*_C–F_ = 272.3 Hz), 124.9 (q, ^3^*J*_C–F_ = 3.5 Hz), 123.8, 119.6, 119.4, 106.9, 60.1, 56.2 ppm. IR: *ν* 1323, 1440, 1499, 1575, 1645, 1724, 2838, 2941 and 3135 cm^−1^.

##### (4-(4-Chlorophenyl)-1-(pyrimidin-2-yl)-1*H*-pyrrol-3-yl)(3,4,5-trimethoxyphenyl)methanone (20)

Was synthesized according to general procedure A, starting from (4-(4-chlorophenyl)-1*H*-pyrrol-3-yl)(3,4,5-trimethoxyphenyl)methanone (34) (300.0 mg, 0.807 mmol) and 2-bromopyrimidine (167 mg, 1.049 mmol). Yield: 72%. Mp 156–159 °C (from ethanol). ^1^H NMR (400 MHz, DMSO-*d*_6_) *δ* 8.86 (d, *J* = 4.8 Hz, 2H), 8.15 (d, *J* = 2.3 Hz, 1H), 8.02 (d, *J* = 2.3 Hz, 1H), 7.48 (t, *J* = 4.9 Hz, 1H), 7.43 (d, *J* = 8.4 Hz, 2H), 7.35 (d, *J* = 8.4 Hz, 2H), 7.11 (s, 2H), 3.77 (s, 6H), 3.74 (s, 3H) ppm. ^13^C NMR (101 MHz, DMSO-*d*_6_) *δ* 189.1, 159.4, 154.4, 152.5, 141.3, 133.8, 132.6, 131.3, 130.0, 128.0, 126.9, 125.9, 123.8, 119.5, 118.7, 106.9, 60.2, 56.0 ppm. IR: *ν* 780, 1570, 1644, 2835, 2997 and 3155 cm^−1^.

##### (4-(4-Nitrophenyl)-1-(pyrimidin-2-yl)-1*H*-pyrrol-3-yl)(3,4,5-trimethoxyphenyl)methanone (21)

Was synthesized according to general procedure A, starting from (4-(4-nitrophenyl)-1*H*-pyrrol-3-yl)(3,4,5-trimethoxyphenyl)methanone (39) (250.0 mg, 0.654 mmol) and 2-bromopyrimidine (135.1 mg, 0.850 mmol). Yield: 63%. Mp 186–188 °C (from ethanol). ^1^H NMR (400 MHz, DMSO-*d*_6_) *δ* 8.89 (d, *J* = 4.8 Hz, 2H), 8.22–8.19 (m, 2H), 8.17 (d, *J* = 8.8 Hz, 2H), 7.70 (d, *J* = 8.8 Hz, 2H), 7.52 (t, *J* = 4.9 Hz, 1H), 7.16 (s, 2H), 3.79 (s, 6H), 3.75 (s, 3H) ppm. ^13^C NMR (101 MHz, DMSO-*d*_6_) *δ* 188.9, 159.5, 154.3, 152.6, 145.8, 141.4, 140.8, 133.6, 129.1, 126.5, 126.1, 123.8, 123.3, 120.3, 119.8, 106.9, 60.2, 56.1 ppm. IR: *ν* 1438, 1573, 1638, 2835, 2933, 3158 and 3491 cm^−1^.

##### (4-(4-(Dimethylamino)phenyl)-1-(pyrimidin-2-yl)-1*H*-pyrrol-3-yl)(3,4,5-trimethoxyphenyl)methanone (22)

Was synthesized according to general procedure A, starting from (4-(4-(dimethylamino)phenyl)-1*H*-pyrrol-3-yl)(3,4,5-trimethoxyphenyl)methanone (35) (300.0 mg, 0.789 mmol) and 2-bromopyrimidine (163.1 mg, 1.026 mmol). Yield: 60%. Mp 179–181 °C (from ethanol). ^1^H NMR (400 MHz, DMSO-*d*_6_) *δ* 8.87–8.82 (m, 2H), 8.09 (d, *J* = 2.4 Hz, 1H), 7.85 (d, *J* = 2.4 Hz, 1H), 7.44 (d, *J* = 5.1 Hz, 1H), 7.23 (d, *J* = 8.3 Hz, 2H), 7.11 (s, 2H), 6.65 (d, *J* = 8.3 Hz, 2H), 3.76 (s, 6H), 3.73 (s, 3H), 2.88 (s, 6H) ppm. ^13^C NMR (101 MHz, DMSO-*d*_6_) *δ* 189.5, 159.3, 154.6, 152.5, 149.3, 141.2, 133.8, 128.8, 128.5, 125.2, 124.0, 121.4, 119.1, 116.8, 112.0, 106.9, 60.1, 56.0, 40.1 ppm. IR: *ν* 1635, 1723, 2836, 2938 and 3128 cm^−1^.

##### (*E*)-(1-(pyrimidin-2-yl)-4-styryl-1*H*-pyrrol-3-yl)(3,4,5-trimethoxyphenyl)methanone (23)

Synthesized according to general procedure A, starting from (*E*)-(4-styryl-1*H*-pyrrol-3-yl)(3,4,5-trimethoxyphenyl)methanone (40) (558.0 mg, 1.535 mmol) and 2-bromopyrimidine (318 mg, 2.0 mmol). Yield: 59%. Mp 173–175 °C (from ethanol). ^1^H NMR (400 MHz, DMSO-*d*_6_) *δ* 8.85 (d, *J* = 4.9 Hz, 2H), 8.25 (d, *J* = 2.2 Hz, 1H), 8.12 (d, *J* = 2.2 Hz, 1H), 7.51–7.45 (m, 4H), 7.36 (t, *J* = 7.6 Hz, 2H), 7.26–7.19 (m, 2H), 7.14 (s, 2H), 3.82 (s, 6H), 3.77 (s, 3H) ppm. ^13^C NMR (101 MHz, DMSO-*d*_6_) *δ* 189.8, 159.4, 154.4, 152.6, 141.0, 137.5, 134.7, 128.7, 128.5, 127.3, 126.4, 126.0, 125.6, 123.4, 120.4, 119.5, 117.0, 106.5, 60.2, 56.1 ppm. IR: *ν* 1571, 1632, 2832, 2932 and 2997 cm^−1^.

##### (4-(4-(Piperidin-1-yl)phenyl)-1-(pyrimidin-2-yl)-1*H*-pyrrol-3-yl)(3,4,5-trimethoxyphenyl)methanone (24)

Was synthesized according to general procedure A, starting from (4-(4-(piperidin-1-yl)phenyl)-1*H*-pyrrol-3-yl)(3,4,5-trimethoxyphenyl)methanone (41) (90.0 mg, 0.214 mmol) and 2-bromopyrimidine (44.0 mg, 0.278 mmol). Yield: 70%. Mp 127–130 °C (from ethanol). ^1^H NMR (400 MHz, CDCl_3_) *δ* 8.66 (d, *J* = 4.8 Hz, 2H), 8.24 (d, *J* = 2.3 Hz, 1H), 7.89 (d, *J* = 2.3 Hz, 1H), 7.32 (d, *J* = 8.7 Hz, 2H), 7.18 (s, 2H), 7.15 (t, *J* = 4.8 Hz, 1H), 6.85 (d, *J* = 8.8 Hz, 2H), 3.90 (s, 3H), 3.83 (s, 6H), 3.14 (t, *J* = 5.4 Hz, 4H), 1.71–1.67 (m, 4H), 1.59–1.54 (m, 2H) ppm. ^13^C NMR (101 MHz, CDCl_3_) *δ* 190.7, 158.7, 155.7, 152.8, 151.2, 141.8, 134.3, 129.23, 129.20, 125.9, 125.0, 124.6, 118.3, 117.7, 116.2, 107.5, 61.0, 56.3, 50.6, 25.9, 24.4 ppm. IR: *ν* 1437, 1567, 1637, 2806, 2931 and 3125 cm^−1^.

##### (5-Chloro-1-(pyridin-2-yl)-1*H*-indol-3-yl)(3,4,5-trimethoxyphenyl)methanone (25)

Was synthesized according to general procedure A, starting from (5-chloro-1*H*-indol-3-yl)(3,4,5-trimethoxyphenyl)methanone (46) (300.0 mg, 0.867 mmol) and 2-bromopyridine (0.107 mL, 1.127 mmol). Yield: 79%. Mp 166–168 °C (from ethanol). ^1^H NMR (400 MHz, DMSO-*d*_6_) *δ* 8.68 (s, 1H), 8.64 (dd, *J* = 4.8, 1.9 Hz, 1H), 8.41 (d, *J* = 8.9 Hz, 1H), 8.31 (d, *J* = 2.3 Hz, 1H), 8.06 (td, *J* = 7.8, 1.9 Hz, 1H), 7.94 (d, *J* = 8.2 Hz, 1H), 7.47–7.41 (m, 2H), 7.21 (s, 2H), 3.87 (s, 6H), 3.78 (s, 3H) ppm. ^13^C NMR (101 MHz, DMSO-*d*_6_) *δ* 189.0, 152.8, 150.9, 148.6, 140.8, 139.7, 136.4, 134.6, 134.1, 129.2, 127.9, 124.4, 122.4, 120.8, 116.3, 116.1, 116.0, 106.4, 60.1, 56.1 ppm. IR: *ν* 765, 1582, 1638, 2835, 2934 and 3071 cm^−1^.

##### (5-Chloro-1-(pyridin-3-yl)-1*H*-indol-3-yl)(3,4,5-trimethoxyphenyl)methanone (26)

Was ynthesized according to general procedure A, starting from (5-chloro-1*H*-indol-3-yl)(3,4,5-trimethoxyphenyl)methanone (46) (300.0 mg, 0.867 mmol) and 3-bromopyridine (0.107 mL, 1.127 mmol). Yield: 40%. Mp 96–98 °C (from ethanol). ^1^H NMR (400 MHz, CDCl_3_) *δ* 8.82 (d, *J* = 2.6 Hz, 1H), 8.73 (dd, *J* = 4.9, 1.5 Hz, 1H), 8.42 (d, *J* = 2.0 Hz, 1H), 7.86 (ddd, *J* = 8.2, 2.7, 1.5 Hz, 1H), 7.82 (s, 1H), 7.55 (dd, *J* = 8.2, 4.8 Hz, 1H), 7.37 (d, *J* = 8.8 Hz, 1H), 7.31 (dd, *J* = 8.8, 2.1 Hz, 1H), 7.12 (s, 2H), 3.92 (s, 3H), 3.90 (s, 6H) ppm. ^13^C NMR (101 MHz, CDCl_3_) *δ* 189.7, 153.2, 149.6, 146.1, 141.6, 136.1, 135.5, 135.2, 134.9, 132.3, 129.6, 128.8, 125.2, 124.6, 122.7, 117.7, 111.5, 106.5, 61.1, 56.5 ppm. IR: *ν* 765, 1581, 1638, 2835, 2937 and 3671 cm^−1^.

##### (5-Chloro-1-(pyrimidin-2-yl)-1*H*-indol-3-yl)(3,4,5-trimethoxyphenyl)methanone (27)

Was synthesized according to general procedure A, starting from (5-chloro-1*H*-indol-3-yl)(3,4,5-trimethoxyphenyl)methanone (46) (300.0 mg, 0.867 mmol) and 2-bromopyrimidine (179.0 mg, 1.127 mmol). Yield: 55%. Mp 214–217 °C (from ethanol). ^1^H NMR (400 MHz, CDCl_3_) *δ* 8.75 (s, 1H), 8.68 (d, *J* = 5.1 Hz, 3H), 8.33 (d, *J* = 2.2 Hz, 1H), 7.31 (dd, *J* = 9.0, 2.2 Hz, 1H), 7.14 (t, *J* = 4.8 Hz, 1H), 7.11 (s, 2H), 3.89 (s, 3H), 3.85 (s, 6H) ppm. ^13^C NMR (101 MHz, CDCl_3_) *δ* 190.1, 158.6, 157.1, 153.2, 141.7, 135.0, 134.7, 134.4, 130.4, 130.0, 125.5, 122.1, 120.6, 118.2, 118.1, 117.5, 106.7, 61.1, 56.5 ppm. IR: *ν* 797, 1575, 1637 and 2967 cm^−1^.

##### (5-Fluoro-1-(pyrimidin-2-yl)-1*H*-indol-3-yl)(3,4,5-trimethoxyphenyl)methanone (28)

Was synthesized according to general procedure A, starting from (5-fluoro-1*H*-indol-3-yl)(3,4,5-trimethoxyphenyl)methanone (47) (350.0 mg, 1.063 mmol) and 2-bromopyrimidine (211.1 mg, 1.382 mmol). Yield: 39%. Mp 205–207 °C (from ethanol). ^1^H NMR (400 MHz, CDCl_3_) *δ* 8.87 (s, 1H), 8.82 (dd, *J* = 9.2, 4.7 Hz, 1H), 8.77 (d, *J* = 4.8 Hz, 2H), 8.11 (dd, *J* = 9.2, 2.7 Hz, 1H), 7.23–7.20 (m, 3H), 7.16 (dd, *J* = 9.0, 2.7 Hz, 1H), 3.98 (s, 3H), 3.95 (s, 6H) ppm. ^13^C NMR (101 MHz, CDCl_3_) *δ* 190.2, 158.5, 157.1 (d, ^1^*J*_C–F_ = 256.9 Hz), 141.6, 135.0 (d, *J*_C–F_ = 9.3 Hz), 132.5, 130.2 (d, *J*_C–F_ = 10.9 Hz), 118.6 (d, *J*_C–F_ = 4.3 Hz), 117.9, 117.5 (d, *J*_C–F_ = 9.1 Hz), 113.2 (d, *J*_C–F_ = 25.0 Hz), 108.1 (d, *J*_C–F_ = 24.8 Hz), 106.7, 61.1, 56.5 ppm. IR: *ν* 1328, 1452, 1570, 1641, 1729, 2839 and 2938 cm^−1^.

##### 1-(Pyrimidin-2-yl)-3-(3,4,5-trimethoxybenzoyl)-1*H*-indole-5-carbonitrile (29)

Was synthesized according to general procedure A, starting from 3-(3,4,5-trimethoxybenzoyl)-1*H*-indole-5-carbonitrile (48) (260.0 mg, 0.773 mmol) and 2-bromopyrimidine (159.8 mg, 1.005 mmol). Yield: 29%. Mp >245 °C (from ethanol). ^1^H NMR (400 MHz, CDCl_3_) *δ* 8.99 (d, *J* = 8.8 Hz, 1H), 8.94 (s, 1H), 8.83–8.79 (m, 3H), 7.70 (dd, *J* = 8.7, 1.8 Hz, 1H), 7.28 (t, *J* = 4.8 Hz, 1H), 7.20 (s, 2H), 3.97 (s, 3H), 3.93 (s, 6H) ppm. ^13^C NMR (101 MHz, CDCl_3_) *δ* 189.8, 158.7, 156.9, 153.3, 141.9, 137.7, 135.3, 134.6, 129.3, 128.3, 127.6, 119.8, 118.6, 118.5, 117.4, 107.5, 106.7, 61.1, 56.5 ppm. IR: *ν* 1423, 1574, 1645, 2225 and 2949 cm^−1^.

##### (5-Methoxy-1-(pyrimidin-2-yl)-1*H*-indol-3-yl)(3,4,5-trimethoxyphenyl)methanone (30)

Was synthesized according to general procedure A, starting from (5-methoxy-1*H*-indol-3-yl)(3,4,5-trimethoxyphenyl)methanone (49) (349.0 mg, 1.022 mmol) and 2-bromopyrimidine (211.3 mg, 1.329 mmol). Yield: 81%. Mp 187–189 °C (from ethanol). ^1^H NMR (400 MHz, CDCl_3_) *δ* 8.79 (s, 1H), 8.73–8.71 (m, 3H), 7.95 (d, *J* = 2.7 Hz, 1H), 7.20 (s, 2H), 7.16 (t, *J* = 4.8 Hz, 1H), 7.06 (dd, *J* = 9.2, 2.7 Hz, 1H), 3.96 (s, 3H), 3.94 (s, 3H), 3.92 (s, 6H) ppm. ^13^C NMR (101 MHz, CDCl_3_) *δ* 190.7, 158.5, 157.3, 157.1, 153.1, 141.4, 135.4, 134.3, 130.8, 130.2, 118.5, 117.7, 117.2, 115.0, 106.7, 104.0, 61.1, 56.4, 55.8 ppm. IR: *ν* 1579, 1644, 2970 and 3675 cm^−1^.

#### 5.1.3. Procedure B. Synthesis of pyrrole intermediates 31–41

##### (4-(4-Tolyl)-1*H*-pyrrol-3-yl)(3,4,5-trimethoxyphenyl)methanone (31)

Was synthesized according to previous literature.^12^ The spectroscopic data were consistent with those reported therein.

##### (4-(4-Methoxyphenyl)-1*H*-pyrrol-3-yl)(3,4,5-trimethoxyphenyl)methanone (32)

Was synthesized according to previous literature.^26^ The spectroscopic data were consistent with those reported therein.

##### (4-(4-(Trifluoromethyl)phenyl)-1*H*-pyrrol-3-yl)(3,4,5-trimethoxyphenyl)methanone (33)

To a suspension of sodium hydride (0.154 g, 3.54 mmol; 60% in mineral oil) in anhydrous diethyl ether (13 mL) at 0 °C a solution of (*E*)-3-(4-(trifluoromethyl)phenyl)-1-(3,4,5-trimethoxyphenyl)prop-2-en-1-one (1.08 g, 2.950 mmol) and 4-toluenesulfonylmethyl isocyanide (TosMIC) (0.633 g, 3.245 mmol) in anhydrous dimethyl sulfoxide/diethyl ether 1 : 2 (24 mL) was added dropwise under and argon stream. The reaction mixture was allowed to stir at room temperature for around 5 h. The reaction mixture was diluted with water and extracted with ethyl acetate (2 × 15 mL). The combined organic layers were washed with brine (2 × 15 mL), dried over anhydrous sodium sulfate, filtered and concentrated *in vacuo* to yield a crude product that was purified by flash chromatography (silica gel, cyclohexane : ethyl acetate = 7 : 3, then 1 : 1 as elution system) to furnish the desired compound 33 (1.195 g, 2.948 mmol; 100%), mp 216–218 °C (from ethanol). ^1^H NMR (400 MHz, DMSO-*d*_6_) *δ* 11.77 (br. s, 1H), 7.60 (d, *J* = 8.3 Hz, 2H), 7.54 (d, *J* = 8.2 Hz, 2H), 7.45 (d, *J* = 2.0 Hz, 1H), 7.23 (d, *J* = 2.0 Hz, 1H), 7.06 (s, 2H), 3.78 (s, 6H), 3.72 (s, 3H) ppm. ^13^C NMR (101 MHz, DMSO-*d*_6_) *δ* 189.1, 152.4, 140.7, 139.7, 134.7, 130.2, 128.8, 128.1, 126.0 (q, ^2^*J* = 31.9 Hz), 125.9 (q, ^1^*J* = 272.6 Hz), 124.6 (q, ^4^*J* = 3.8 Hz), 124.1, 120.6 (d, ^3^*J* = 7.1 Hz), 106.7, 60.1, 55.9 ppm. IR: *ν* 1161, 1572, 1608 and 3205 cm^−1^.

##### (4-(4-Chlorophenyl)-1*H*-pyrrol-3-yl)(3,4,5-trimethoxyphenyl)methanone (34)

Was synthesized according to previous literature.^27^ The spectroscopic data were consistent with those reported therein.

##### (4-(4-(Dimethylamino)phenyl)-1*H*-pyrrol-3-yl)(3,4,5-trimethoxyphenyl)methanone (35)

Was synthesized according to general procedure B starting from (*E*)-3-(4-(dimethylamino)phenyl)-1-(3,4,5-trimethoxyphenyl)prop-2-en-1-one (1.0 g, 2.929 mmol). Yield: 100%. Mp 195–198 °C (from ethanol). ^1^H NMR (400 MHz, DMSO-*d*_6_) *δ* 11.47 (br. s, 1H), 7.30 (d, *J* = 2.8 Hz, 1H), 7.17 (d, *J* = 8.4 Hz, 2H), 7.03 (s, 2H), 6.93 (d, *J* = 2.5 Hz, 1H), 6.62 (d, *J* = 8.4 Hz, 2H), 3.76 (s, 6H), 3.71 (s, 3H), 2.85 (s, 6H) ppm. ^13^C NMR (101 MHz, DMSO-*d*_6_) *δ* 189.3, 152.3, 148.7, 140.4, 135.1, 129.0, 127.1, 125.7, 123.5, 120.3, 118.1, 112.1, 106.7, 60.1, 55.8, 40.4 ppm. IR: *ν* 1575, 1613, 2925 and 3261 cm^−1^.

##### (4-(3,5-Dimethoxyphenyl)-1*H*-pyrrol-3-yl)(3,4,5-trimethoxyphenyl)methanone (36)

Was synthesized according to general procedure B starting from (*E*)-3-(3,5-dimethoxyphenyl)-1-(3,4,5-trimethoxyphenyl)prop-2-en-1-one (0.645 mg, 1.799 mmol). Yield: 100%. Mp 146–148 °C (from ethanol). ^1^H NMR (400 MHz, DMSO-*d*_6_) *δ* 11.65 (br. s, 1H), 7.38 (t, *J* = 2.7 Hz, 1H), 7.13 (t, *J* = 2.4 Hz, 1H), 7.00 (s, 2H), 6.46 (t, *J* = 1.5 Hz, 2H), 6.29 (t, *J* = 2.4 Hz, 1H), 3.75 (s, 6H), 3.70 (s, 3H), 3.66 (s, 6H) ppm. ^13^C NMR (101 MHz, DMSO-*d*_6_) *δ* 189.5, 159.9, 152.3, 140.5, 137.2, 134.9, 127.1, 125.3, 120.9, 119.4, 106.7, 106.5, 97.9, 60.0, 55.8, 55.0 ppm. IR: *ν* 1497, 1575, 1599, 1617 and 3303 cm^−1^.

##### (3,4,5-Trimethoxyphenyl)(4-(3,4,5-trimethoxyphenyl)-1*H*-pyrrol-3-yl)methanone (37)

Was synthesized according to general procedure B starting from (*E*)-1,3-bis(3,4,5-trimethoxyphenyl)prop-2-en-1-one (0.969 mg, 2.495 mmol). Yield: 100%. Mp 142–144 °C (from ethanol). ^1^H NMR (400 MHz, DMSO-*d*_6_) *δ* 11.66 (br. s, 1H), 7.41 (t, *J* = 2.5 Hz, 1H), 7.12 (t, *J* = 2.3 Hz, 1H), 6.95 (s, 2H), 6.55 (s, 2H), 3.71 (s, 6H), 3.67 (s, 3H), 3.66 (s, 6H), 3.60 (s, 3H) ppm. ^13^C NMR (101 MHz, DMSO-*d*_6_) *δ* 189.8, 152.3, 140.5, 135.8, 135.0, 131.1, 130.3, 127.2, 125.5, 121.0, 119.2, 106.8, 106.2, 60.1, 60.0, 55.8, 55.7 ppm. IR: *ν* 1581, 1605, 2938 and 3271 cm^−1^.

##### (4-(4-Fluorophenyl)-1*H*-pyrrol-3-yl)(3,4,5-trimethoxyphenyl)methanone (38)

Was synthesized according to previous literature.^12^ The spectroscopic data were consistent with those reported therein.

##### (4-(4-Nitrophenyl)-1*H*-pyrrol-3-yl)(3,4,5-trimethoxyphenyl)methanone (39)

Was synthesized according to previous literature.^12^ The spectroscopic data were consistent with those reported therein.

##### (*E*)-(4-styryl-1*H*-pyrrol-3-yl)(3,4,5-trimethoxyphenyl)methanone (40)

Was synthesized according to previous literature.^26^ The spectroscopic data were consistent with those reported therein.

##### (4-(4-(Piperidin-1-yl)phenyl)-1*H*-pyrrol-3-yl)(3,4,5-trimethoxyphenyl)methanone (41)

Was synthesized according to general procedure B starting from (*E*)-3-(4-(piperidin-1-yl)phenyl)-1-(3,4,5-trimethoxyphenyl)prop-2-en-1-one (0.900 mg, 2.359 mmol). Yield: 63%. Mp 207–209 °C (from ethanol). ^1^H NMR (400 MHz, CDCl_3_) *δ* 8.95 (br. s, 1H), 7.22 (d, *J* = 8.8 Hz, 3H), 7.09 (s, 2H), 6.82 (d, *J* = 7.1 Hz, 3H), 3.87 (s, 3H), 3.79 (s, 6H), 3.09 (t, *J* = 5.5 Hz, 4H), 1.70–1.63 (m, 4H), 1.59–1.49 (m, 2H) ppm. ^13^C NMR (101 MHz, CDCl_3_) *δ* 191.1, 152.7, 150.9, 141.4, 134.9, 129.3, 126.8, 126.5, 125.7, 121.9, 117.9, 116.4, 107.4, 61.0, 56.3, 50.8, 25.9, 24.4 ppm. IR: *ν* 1577, 1613, 2934 and 3252 cm^−1^.

#### 5.1.4. Procedure C. Synthesis of indole intermediates 46–49 (ref. 17)

##### (5-Chloro-1*H*-indol-3-yl)(3,4,5-trimethoxyphenyl)methanone (46)

To a MW reaction vial equipped with a magnetic stirring bar, a solution of 5-chloro-1*H*-indole (42) (300.0 mg; 1.979 mmol), anhydrous aluminum chloride (343.0 mg; 2.573 mmol) and 3,4,5-trimethoxybenzoyl chloride (593.0 mg, 2.573 mmol) in 1,2-dichloroethane (8 mL) was added. The vial was sealed and placed in the MW reactor in closed-vessel mode, and heated for 4 min (150 W, internal temp 110 °C, ramp time of 1 min, *P*_max_ 150 psi). Upon completion, the reaction mixture was cooled to room temperature, quenched with ice water and extracted with ethyl acetate (2 × 15 mL). The combined organic layers were washed with a saturated solution of sodium hydrogen carbonate (1 × 15 mL), brine (2 × 15 mL), dried over anhydrous sodium sulfate, filtered and concentrated *in vacuo* to a small volume. The residue was filtered through a pad of silica gel and evaporated under reduced pressure to give the desired compound 46 (658.0 mg, 1.903 mmol; 96%), Mp 242–245 °C (from ethanol). ^1^H NMR (400 MHz, DMSO-*d*_6_) *δ* 12.20 (br. s, disappeared after treatment with D_2_O, 1H), 8.24 (d, *J* = 2.2 Hz, 1H), 8.20 (s, 1H), 7.54 (d, *J* = 8.6 Hz, 1H), 7.28 (dd, *J* = 8.6, 2.2 Hz, 1H), 7.10 (s, 2H) ppm. ^13^C NMR (101 MHz, DMSO-*d*_6_) *δ* 188.8, 152.7, 140.2, 137.0, 135.3, 135.3, 127.6, 126.6, 123.2, 120.6, 114.4, 113.9, 106.0, 60.1, 56.0 ppm. IR: *ν* 1645, 1711 and 3259 cm^−1^.

##### (5-Fluoro-1*H*-indol-3-yl)(3,4,5-trimethoxyphenyl)methanone (47)

Was synthesized as 46 starting from 5-fluoro-1*H*-indole (43) (300.0 mg; 2.219 mmol). Yield: 92%. Mp 179–181 °C (from ethanol). ^1^H NMR (400 MHz, DMSO-*d*_6_) *δ* 10.88 (br. s, disappeared after treatment with D_2_O, 1H), 6.93 (s, 1H), 6.67 (dd, *J* = 10.0, 2.6 Hz, 1H), 6.27 (dd, *J* = 8.8, 4.6 Hz, 1H), 5.89–5.85 (m, 1H), 5.84 (s, 2H), 2.60 (s, 6H), 2.50 (s, 3H) ppm. ^13^C NMR (101 MHz, DMSO-*d*_6_) *δ* 188.8, 160.9, 159.2 (d, ^1^*J*_C–F_ = 234.3 Hz), 152.7, 140.1, 137.1, 135.5, 133.3, 127.1 (d, *J*_C–F_ = 10.7 Hz), 114.9 (d, *J*_C–F_ = 4.6 Hz), 113.5 (d, *J*_C–F_ = 9.9 Hz), 111.3 (d, *J*_C–F_ = 25.6 Hz), 106.3 (d, *J*_C–F_ = 24.4 Hz), 106.0, 60.1, 56.0 ppm. IR: *ν* 1454, 1574, 1633 and 3229 cm^−1^.

##### 3-(3,4,5-Trimethoxybenzoyl)-1*H*-indole-5-carbonitrile (48)

Was synthesized as 46 starting from 1*H*-indole-5-carbonitrile (44) (300.0 mg; 2.110 mmol). Yield: 41%. Mp 247–250 °C (from ethanol). ^1^H NMR (400 MHz, DMSO-*d*_6_) *δ* 12.52 (br. s, disappeared after treatment with D_2_O, 1H), 8.60 (d, *J* = 1.6 Hz, 1H), 8.35 (s, 1H), 7.70 (d, *J* = 8.5 Hz, 1H), 7.64 (dd, *J* = 8.5, 1.6 Hz, 1H), 7.12 (s, 2H), 3.87 (s, 6H), 3.77 (s, 3H) ppm. ^13^C NMR (101 MHz, DMSO-*d*_6_) *δ* 188.8, 152.7, 140.4, 138.6, 137.8, 134.9, 126.5, 126.3, 126.0, 120.2, 115.0, 113.7, 106.1, 104.1, 60.1, 56.0 ppm. IR: *ν* 1573, 1607, 2228 and 3304 cm^−1^.

##### (5-Methoxy-1*H*-indol-3-yl)(3,4,5-trimethoxyphenyl)methanone (49)

Was synthesized as 46 starting from 5-methoxy-1*H*-indole (45) (300.0 mg; 2.039 mmol). Yield: 57%. Mp 200–202 °C (from ethanol). ^1^H NMR (400 MHz, DMSO-*d*_6_) *δ* 11.92 (br. s, disappeared after treatment with D_2_O, 1H), 8.03 (s, 1H), 7.78 (d, *J* = 2.6 Hz, 1H), 7.41 (d, *J* = 8.8 Hz, 1H), 7.07 (s, 2H), 6.89 (dd, *J* = 8.7, 2.5 Hz, 1H), 3.85 (s, 6H), 3.80 (s, 3H), 3.76 (s, 3H) ppm. ^13^C NMR (101 MHz, DMSO-*d*_6_) *δ* 188.9, 155.5, 152.6, 139.9, 135.9, 135.8, 131.6, 127.3, 114.7, 112.9, 105.9, 103.3, 60.1, 56.0, 55.3 ppm. IR: *ν* 1574, 1633 and 3220 cm^−1^.

### 5.2. Computational studies

All molecular modeling studies were performed on a SuperMicro, Intel Xeon Silver machine 64 core running Ubuntu 20LTS equipped with GPU NVIDIA A30. The tubulin structure was downloaded from the PDB (http://www.rcsb.org/), PDB code: 8WD0.^28^ Ligand structures were prepared with Maestro.^29^ The proteins were prepared by the Protein Preparation Wizard of Maestro.^30^ The docking simulations were performed using PLANTS.^31^ MMGBSA computations were carried out by the Maestro suite, using the OPLS4 forcefield. Molecular dynamics was carried out with Desmond by Maestro^32,33^ suite and repeated three times. Images shown in the manuscript were prepared with Pymol.^34^

### 5.3. Biology

#### 5.3.1. Tubulin assembly and colchicine binding assays

The assembly reaction mixtures contained 0.8 M monosodium glutamate (pH 6.6 with HCl in a 2 M stock solution), 10 μM tubulin, 4% (v/v) DMSO, and varying concentrations of drug. Following a 15 min preincubation at 30 °C, samples were chilled on ice, GTP to 0.4 mM was added, and turbidity development was followed at 350 nm in a temperature-controlled recording spectrophotometer for 20 min at 30 °C. The extent of reaction was measured. Full experimental details were previously described.^35^

#### 5.3.2. [^3^H]colchicine binding assay

The reaction mixtures contained 1.0 μM tubulin, 5.0 μM [^3^H]colchicine, and 5.0 or 1.0 μM inhibitor, as indicated, and were incubated for 10 min at 37 °C. Complete details were described previously.^36^

#### 5.3.3. Cell lines

MCF-7 breast carcinoma cells were obtained from the National Cancer Institute drug screening laboratory. HCT116 cell line was grown in Dulbecco's modified Eagle's medium (DMEM). BX-PC3 cells were grown in RPMI-1640 medium. Both media were supplemented with 10% fetal bovine serum (FBS), 100 U mL^−1^ penicillin, 100 mg mL^−1^ streptomycin and 1% of l-glutamine. Cell lines were cultured at 37 °C in 5% CO_2_ and 95% air in a humidified incubator. T-ALL (Jurkat) cells were grown in RPMI-1640 medium. Both media were supplemented with 115 units per mL of penicillin G, 115 μg mL^−1^ of streptomycin, and 10% fetal bovine serum (FBS, all purchased from Invitrogen, Milan, Italy). Cancer cells were seeded in tissue treated, flat bottom, 384-well plates (Corning, Corning, NY, USA) in 24 μL of complete medium per well, using a Microlab STAR 96-CORE liquid handling system (Hamilton Bonaduz, Bonaduz, Switzerland).

##### Cell viability assays

The methodology for the evaluation of the growth of human MCF-7 breast carcinoma cells previously described, except that cells were grown for 96 h for IC_50_ determinations.^37^ Cell viability of HCT116 and BX-PC3 cells was determined using the 3-(4,5-dimethylthiazol-2-yl)-5-(3-carboxymethoxyphenyl)-2-(4-sulfophenyl)-2*H*-tetrazolium (MTS) colorimetric assay.^38^ The cells were seeded into 96-well plates to a density of 5000 cells for well. After 24 h of growth, the compound was added at 5–320 nM concentrations. After 48 h of growth, the MTS reagent was added to each well and absorbance at 590 nm was measured by a microplate reader (Victor, PerkinElmer). The results were expressed as % relative to control cells (100%) and IC_50_ values were calculated using GraphPad Prism statistics software by nonlinear regression analysis.

For T-ALL, drug stock solutions were prepared for each compound by dissolving them at 10 mM in DMSO. To ensure a final concentration of DMSO < 0.5%, an intermediate drug dilution in PBS was performed as previously described.^39^ Each compound was tested in a 6-points 2-fold dose–response curve, and each dose was tested in duplicate within each plate, in three independent experiments. At the experimental endpoint (96 h), 3 μL of resazurin stock solution (10×) was added to each well for a final concentration of 44 μM, and the plates were incubated at 37 °C for an additional 3 h. The fluorescence signal was measured at 590 nm using a Spark 10 M spectrophotometer (Tecan Group Ltd., Mannedorf, Switzerland). Only plates with *Z*′ > 0.5 were further analyzed for the drug screening. Raw data were normalized according to the following equation: cell viability (%) = (*x* − POS)/(NEG − POS) × 100, where *x* is the relative fluorescence units (RFU) collected from each single well, NEG is the mean of intraplate negative controls (DMSO, corresponding to 100% cell viability), and POS is the mean of intraplate positive controls (benzethonium chloride 75 μM, equal to 0% cell viability). Normalized data were then processed with the GRmetrics package^40^ using *R* 3.6.3 and Rstudio version for GI_50_ calculation, defined as the compound concentration required to inhibit cell proliferation by 50% compared to DMSO.

##### Spheroids

3D culturing of BX-PC3 and HCT116 cells were performed seeding 500 cells/well into ultra-low attachment 96-well round-bottomed plates and cultured in supplemented RPMI and DMEM, respectively, at 37 °C under 0.5% CO_2_ atmosphere for 7 days, until spheroids were formed. To evaluate the effect of compound 4 on spheroid growth, a range of doses (10–320 nM) was added to the cell suspension after spheroid formation, and the culture was maintained for 48 h. Spheroid diameters wwere measured using the ZEISS ZEN imaging software from the pictures obtained with an inverted optical microscope (Axio Vert A1, Zeiss, Oberkochen, Germany).

##### Statistical analysis

For data analysis GraphPrism 5 software was used as previously described.^6^

## 6. Conclusions

We synthesized new pyrrole (3–25) and indole (26–30) derivatives as tubulin assembly inhibitors by using a MW-assisted approach allowing fast reactions and improved yields. For the drug design of the new inhibitors, we applied notions of *aryl ring reversal* and *conjunctive approach*.^7^ We moved the 4-methylphenyl ring from position 1 to position 4 of pyrrole 1 to give compounds 3–24 or fused this aryl group with the pyrrole to give the indole compounds 26–30. The adopted MW-based synthetic procedures resulted in considerable improvements in reaction rates and yields. The average yields for the pyrrole and indole series were 60 and 59%, respectively. These yields were clearly superior to the previously reported procedure for the synthesis of analogs of (1-phenyl-4-(aryl)-1*H*-pyrrol-3-yl)(3,4,5-trimethoxyphenyl)methanone. The previous procedure reached average yields of 22% (Chart S1, ESI†).^12^ Compound (4-(4-methoxyphenyl)-1-(pyridin-2-yl)-1*H*-pyrrol-3-yl)(3,4,5 trimethoxyphenyl)methanone (4) inhibited tubulin assembly and [^3^H]colchicine binding by 78%. Compound 4 demonstrated strong activity against the growth of the MCF-7 cancer cells with an IC_50_ of 9.6 nM. Beside this cancer cell line, compound 4 inhibited effectively the growth of HCT116 (colorectal), BX-PC3 (pancreatic) and Jurkat (T-cell acute lymphoblastic leukemia) cancer cells with IC_50_ values of 18, 17 and 41 nM, respectively. Compound 4 at the 320 and 160 nM altered the morphology of treated spheroids in both BX-PC3 and HCT116 cell lines after 48 h treatments, respectively, and caused progressively less defined shapes and significant reductions of diameter. These results encourage further development of this novel small molecule as an anticancer agent.

## Author contributions

DM, synthesis, investigation, writing and editing; MP, synthesis, formal analysis, investigation; CC, synthesis; AC, PS, molecular modelling; MS synthesis, spectral analysis; GP, EM, MCF-7 and Jurkat cells; EH, tubulin, [^3^H]colchicine and MCF-7 cells and editing; CM, RL, HCT116 and BX-PC3 cells, and spheroids; RS, conceptualization, resources, investigation, writing – original; GLR, supervision, investigation, project administration, review and editing.

## Conflicts of interest

The authors declare that they have no known competing financial interests or personal relationships that could have appeared to influence the work reported in this paper.

## Supplementary Material

MD-016-D5MD00406C-s001

## Data Availability

Data will be made available on request.
