# Supplementary material for: Microwave-assisted synthesis of tubulin assembly inhibitors as anticancer agents by aryl ring reversal and conjunctive approach
Source: RSC Med Chem. 2025 Jul 2;16(10):4845–58. doi: 10.1039/d5md00406c (PMC12306498; doi:10.1039/d5md00406c)
Supplement: MD-016-D5MD00406C-s001 [file MD-016-D5MD00406C-s001.pdf]

Supplementary Information (SI) for RSC Medicinal Chemistry.
This journal is © The Royal Society of Chemistry 2025

Supplementary Information

Microwave-assisted synthesis of tubulin assembly inhibitors as anticancer agents by aryl ring reversal and conjunctive approach

Domiziana Masci,[a,b] Michela Puxeddu,[c] Claudia Colla,[c] Antonio Coluccia,[c] Martina Santelli,[a] Pietro Sciò,[c] Elena Mariotto,[d,e] Giampietro Viola,[d,e] Ernest Hamel,[f] Rosa Lerose,[g] Carmela Mazzoccoli,[g] Romano Silvestri,[c,*] and Giuseppe La Regina[c]

[a] Department of Basic Biotechnological Sciences, Intensivological and Perioperative Clinics, Catholic University of the Sacred Heart, Largo Francesco Vito 1, 00168 Rome, Italy

[b] Policlinico Universitario A. Gemelli Foundation-IRCCS, 00168 Rome, Italy

[c] Laboratory affiliated with the Institute Pasteur Italy – Cenci Bolognetti Foundation, Department of Drug Chemistry and Technologies, Sapienza University of Rome, Piazzale Aldo Moro 5, 00185 Rome, Italy

[d] Department of Woman's and Child's Health, University of Padua, Hemato-oncology Lab, Via Giustiniani 3, 35128 Padua, Italy

[e] Istituto di Ricerca Pediatrica Città della Speranza - IRP, Corso Stati Uniti, 4-35127 Padua, Italy

[f] Molecular Pharmacology Branch, Developmental Therapeutics Program, Division of Cancer Treatment and Diagnosis, Frederick National Laboratory for Cancer Research, National Cancer Institute, National Institutes of Health, Frederick, Maryland 21702, United States

[g] Hospital Pharmacy, Centro di Riferimento Oncologico della Basilicata (IRCCS-CROB), 85028 Rionero in Vulture, Italy

Corresponding author: romano.silvestri@uniroma1.it

Contents of Supporting Information

Chart 1S. Comparing MW-assisted synthesis of **3-30** with our previous protocols (Ref.1S).
Figure S1. Correlation between the IC_{50} values of MCF-7 cancer cell growth inhibition and % values of [^{3}H]colchicine binding inhibition (Table 1 and Table 2)
Figure S2. Inhibition of HCT116 cell growth by compound **4**.
Figure S3. Inhibition of BX-PC3 cell growth by compound **4**.
Figure S4. RMSD Plot.
Figure S5. New hydrogen bond between the ketone group of compound **4** and Asp251β side chain, observed with 80% formation frequency during MD simulation.
Table S1. Docking score and interactions of compound **4**.
Table S2. ADME profile of compound **4**.
^{1}H and ^{13}C NMR spectra of compounds 3-30.
IR spectra of compounds 3-30.
HPLC chromatograms of compounds 4, 14, 15, 18, 20, 22 and 24.

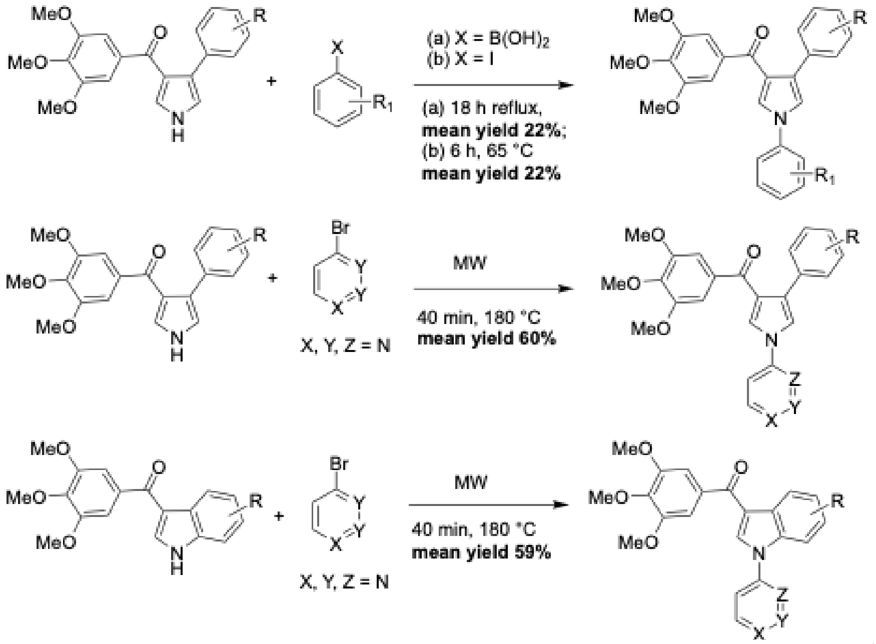

Chart 1S. Comparing MW-assisted synthesis of **3-30** with our previous protocols.[S1]

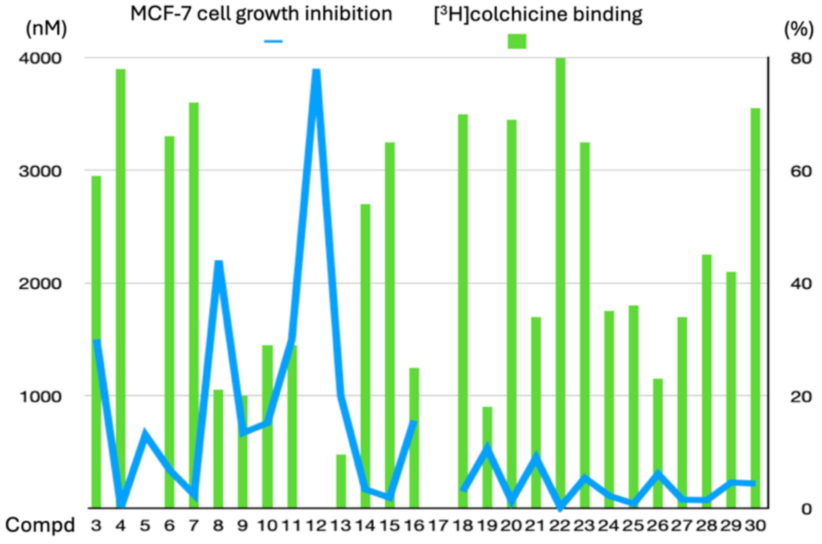

Figure S1. Correlation between the IC_{50} values of MCF-7 cancer cell growth inhibition and % values of [^{3}H]colchicine binding inhibition (Table 1 and Table 2).

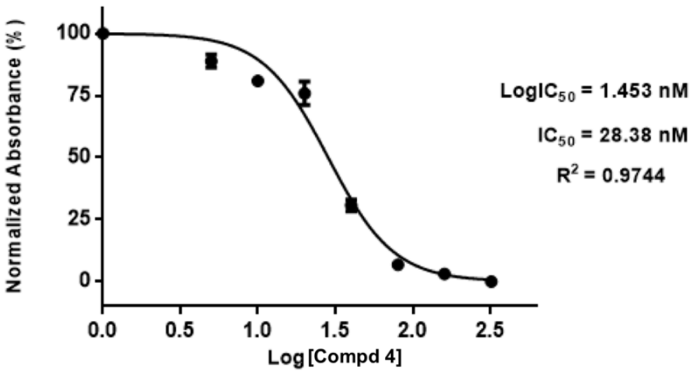

Figure S2. Inhibition of HCT116 cell growth by compound **4**.

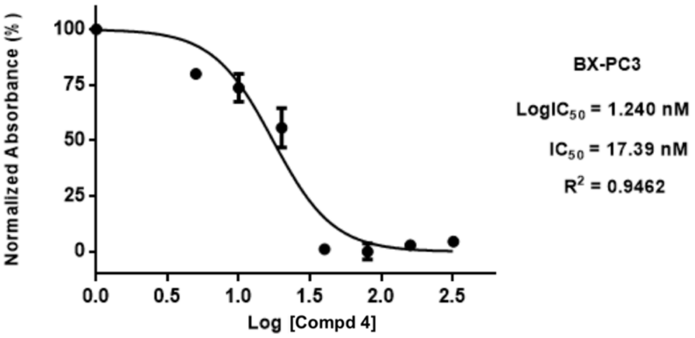

Figure S3. Inhibition of BX-PC3 cell growth by compound **4**.

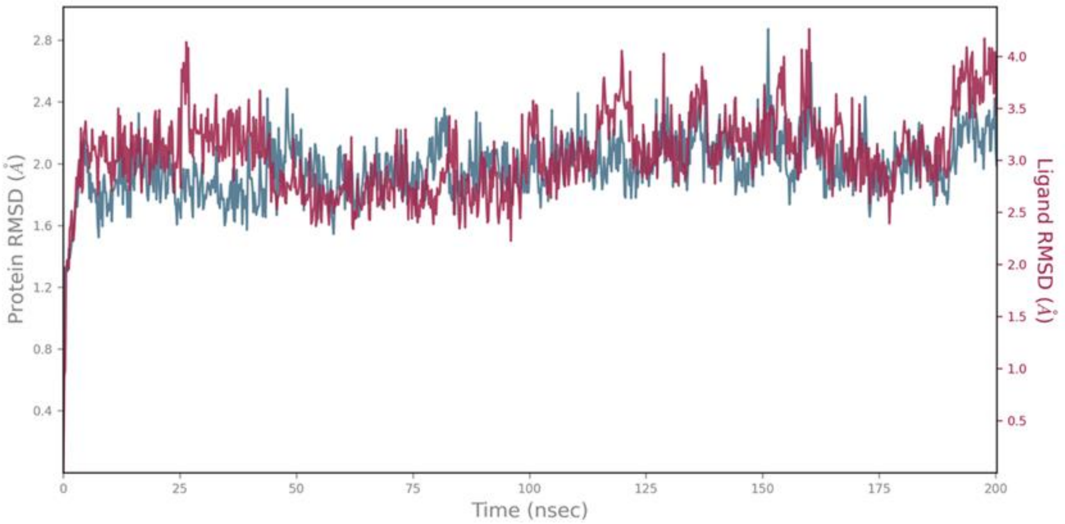

Figure S4. RMSD Plot.

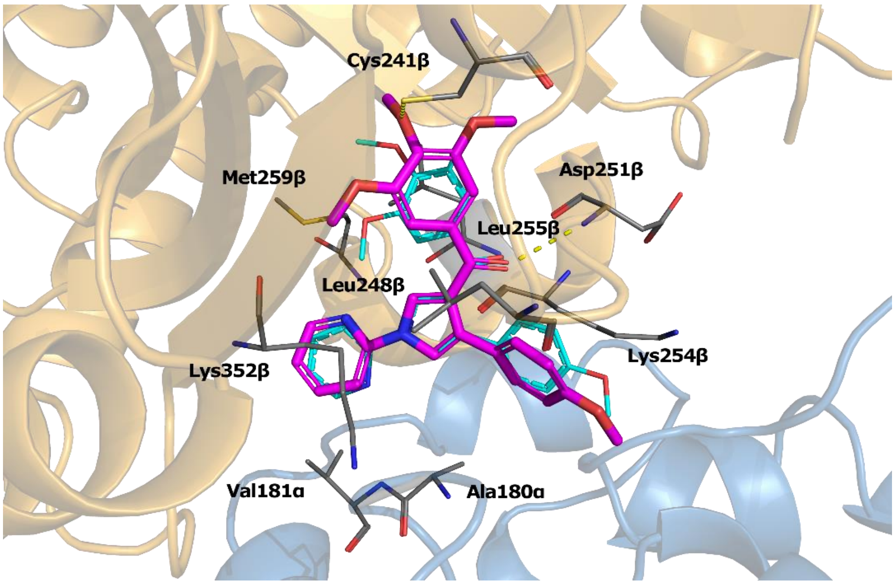

Figure S5. New hydrogen bond between the ketone group of compound **4** and Asp251β side chain, observed with 80% formation frequency during MD simulation.

Table S1. Docking score and interactions of compound **4**.

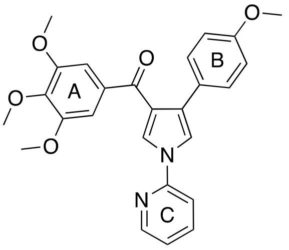

Cpd	Docking score[a]	Type of Interactions: H-bond	Type of Interactions: Hydrophobic[b]	Type of Interactions: Polar
**4**	-7.65	Ring A *p*OCH_3 Cys241β 3.1 Å	Ring A Leu254β 4.1 Å<br>Ring B Leu248β 4.1 Å<br>Ring B Lys252β 3.7 Å<br>Ring C Met259β 3.8 Å<br>Ring C Lys352β 3.7 Å<br>Ring C Ala180α 4.4 Å<br>Ring C Val181α 4.7 Å	Ring B OCH_3 Lys254β 4.2 Å

[a]Docking score is in Kcal/mol; [b] Distance are computed between the ring centroid and closest residue atom.

Table S2. ADME parameter for compound **4**.

Cpd	MW[a]	H don[b]	H Acc[c]	logP[d]	logS[e]	tPSA[f]	L r[g]	V r[h]
**4**	444.48	0	6	4.44	-5.38	71.81	0	0

Physicochemical properties predicted by QikProp:[S2,3] *[a]* Molecular Weight; *[b]* Number of H-bond acceptors; *[c]* Number of H-bond donors; *[d]* Octanol-water partition coefficient predictor by XLOGP3 method;[S4] [e] Logarithm of compounds water solubility by ESOL method;[S5] [f] Topological polar surface area; *[g]* L r: Lipinsky Rule deviation (log P <5, H-bond donors ≤5, H-bond acceptors ≤10, and a molecular weight <500);[S6] [h] V r: Veber rule deviation (rotable bonds ≤ 10, tPSA ≤ 140).[S7]

References of SI

(S1) G. La Regina, R. Bai, A. Coluccia, V. Famiglini, S. Passacantilli, V. Naccarato, G. Ortar, C. Mazzoccoli, V. Ruggieri, F. Agriesti, C. Piccoli, T Tataranni, M. Nalli, A. Brancale, S. Vultaggio, C. Mercurio, M. Varasi, C. Saponaro, S. Sergio, M. Maffia, A. M. L. Coluccia, E. Hamel, R. Silvestri. 3-Aroyl-1,4-diarylpyrroles inhibit chronic myeloid leukemia cell growth through an interaction with tubulin. *ACS Med. Chem. Lett*. 2017, **8**, 521-526. DOI: 10.1021/acsmedchemlett.7b00022.

(S2) E. M. Duffy, W. L. Jorgensen. Prediction of Properties from Simulations: Free Energies of Solvation in Hexadecane, Octanol, and Water. *J. Am. Chem. Soc*. 2000, **122**, 2878-2888. DOI: 10.1021/ja993663t.

(S3) Schrödinger Release 2018: QikProp, Schrödinger, LLC, New York, NY, 2018.

(S4) T. Cheng, Y. Zhao, X. Li, F. Lin, Y. Xu, X. Zhang, Y. Li, R. Wang, L. Lai. Computation of octanol-water partition coefficients by guiding an additive model with knowledge. *J Chem Inf Model*. 2007, **47**, 2140-2148. DOI: 10.1021/ci700257y.

(S5) J. S. Delaney. ESOL: Estimating Aqueous Solubility Directly from Molecular Structure. *J. Chem. Inf. Comput. Sci*. 2004, **44**, 1000-1005. DOI: 10.1021/ci034243x.

(S6) C. A. Lipinski, F. Lombardo, C. A. Dominy, P. J. Feeney. Experimental and computational approaches to estimate solubility and permeability in drug discovery and development settings. *Adv. Drug Delivery Rev*. 2001, **46**, 3-26. DOI: 10.1016/s0169-409x(00)00129-0.

(S7) D. F. Veber, S. R. Johnson, H. Y. Cheng, B. R. Smith, K. W. Ward, K. D. Kopple. Molecular properties that influence the oral bioavailability of drug candidates. *J Med Chem*. 2002, **6**, 2615-23. DOI: 10.1021/jm020017n.

^{1}H and ^{13}C NMR Spectra of Pyrroles **3-24** and Indoles **25-30**

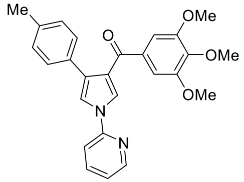
Me
O
OMe
OMe
OMe
N
N

3

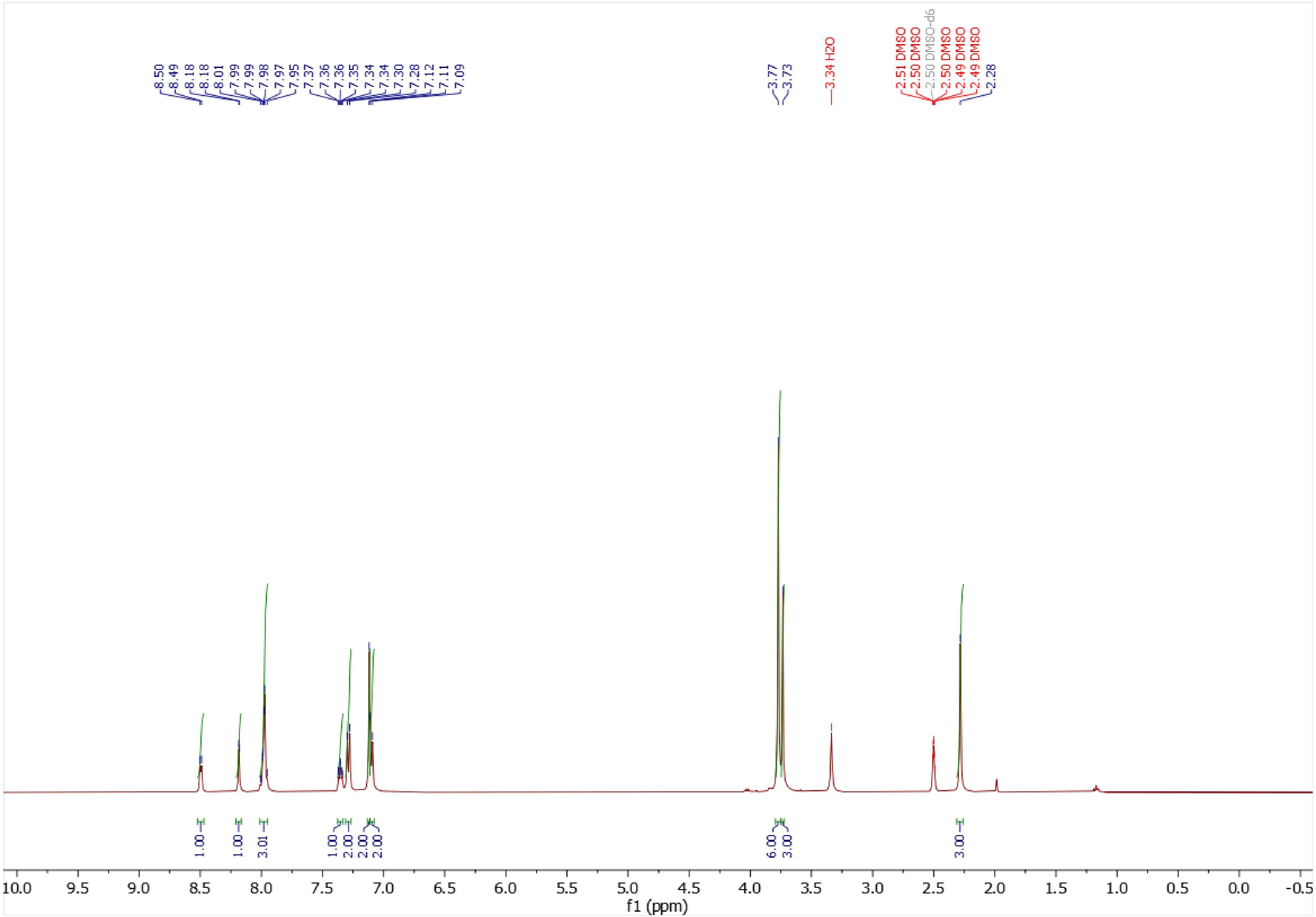
8.50
8.49
8.18
8.18
8.01
7.99
7.98
7.97
7.95
7.37
7.36
7.36
7.35
7.34
7.34
7.30
7.28
7.12
7.11
7.09
3.77
3.73
3.34 H2O
2.51 DMSO
2.50 DMSO
2.50 DMSO-d6
2.50 DMSO
2.49 DMSO
2.49 DMSO
2.28
1.00
1.00
3.01
1.00
2.00
2.00
2.00
6.00
3.00
3.00
10.0
9.5
9.0
8.5
8.0
7.5
7.0
6.5
6.0
5.5
5.0
4.5
4.0
3.5
3.0
2.5
2.0
1.5
1.0
0.5
0.0
-0.5
f1 (ppm)

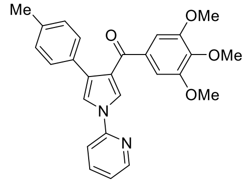
Me
O
OMe
OMe
OMe
N
N

3

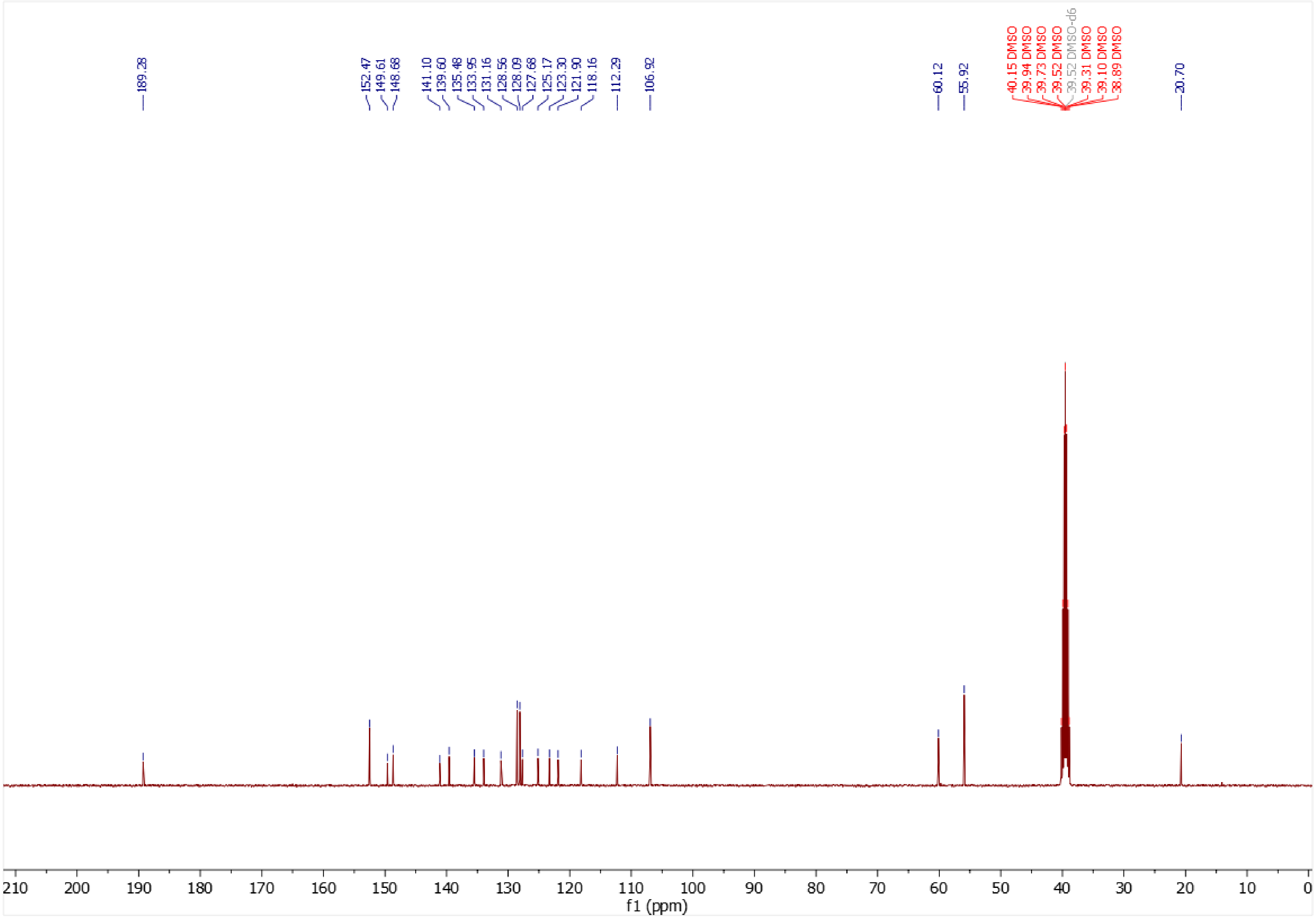
189.28
152.47
149.61
148.68
141.10
139.60
135.48
133.95
131.16
128.56
128.09
127.68
125.17
123.30
121.90
118.16
112.29
106.92
60.12
55.92
40.15 DMSO
39.94 DMSO
39.73 DMSO
39.52 DMSO
39.52 DMSO-d6
39.31 DMSO
39.10 DMSO
38.89 DMSO
20.70
210 200 190 180 170 160 150 140 130 120 110 100 90 80 70 60 50 40 30 20 10 0
f1 (ppm)

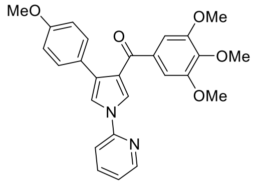
MeO
O
OMe
OMe
OMe
N
N

4

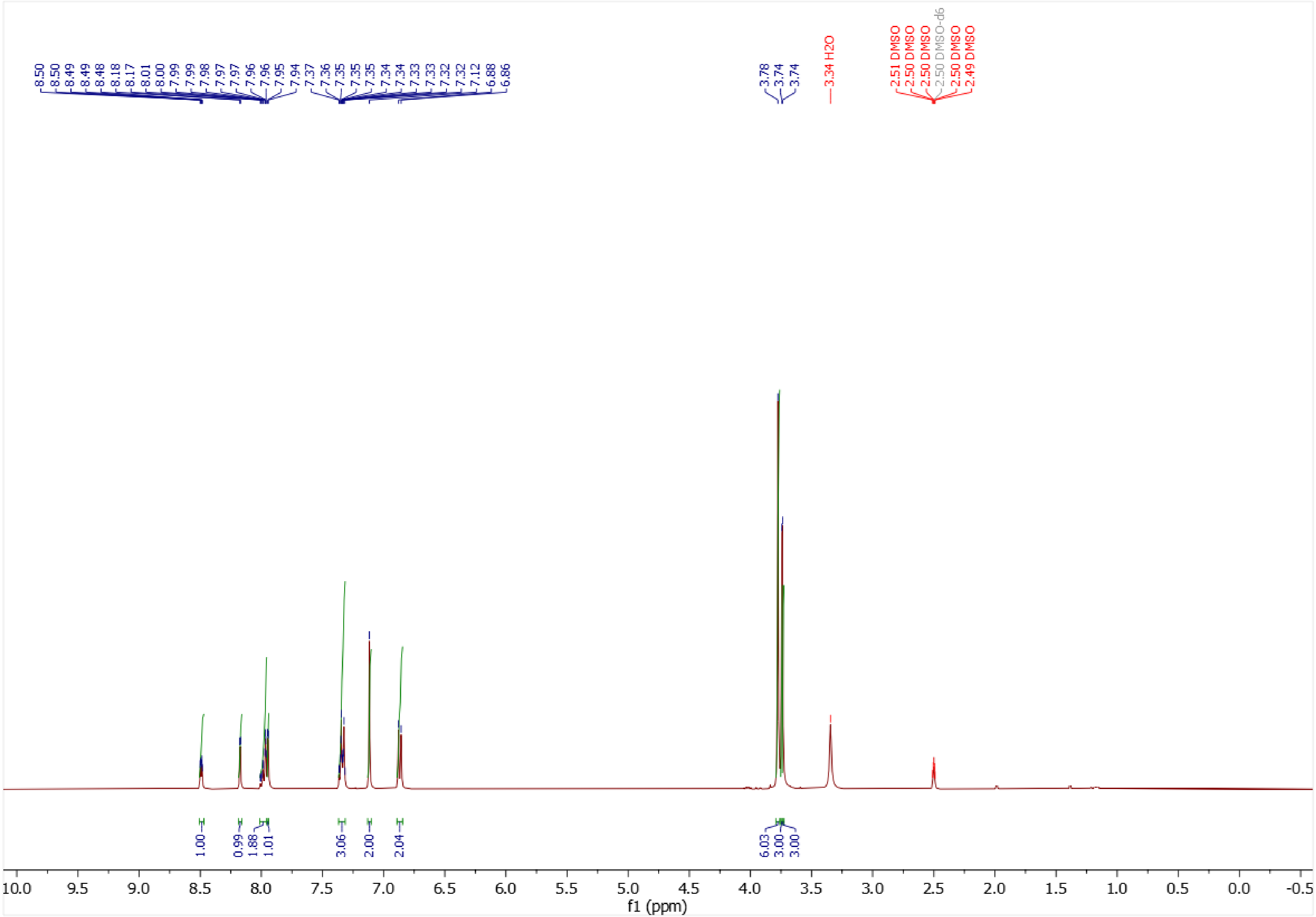
8.50
8.50
8.49
8.49
8.48
8.18
8.17
8.01
8.00
7.99
7.99
7.98
7.97
7.97
7.96
7.96
7.95
7.94
7.37
7.36
7.35
7.35
7.35
7.34
7.34
7.33
7.32
7.32
7.12
6.88
6.86
3.78
3.74
3.74
3.34 H2O
2.51 DMSO
2.50 DMSO
2.50 DMSO
2.50 DMSO-d6
2.50 DMSO
2.49 DMSO
1.00
0.99
1.88
1.01
3.06
2.00
2.04
6.03
3.00
3.00
10.0 9.5 9.0 8.5 8.0 7.5 7.0 6.5 6.0 5.5 5.0 4.5 4.0 3.5 3.0 2.5 2.0 1.5 1.0 0.5 0.0 -0.5
f1 (ppm)

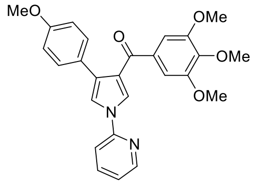
MeO
O
OMe
OMe
OMe
N
N

4

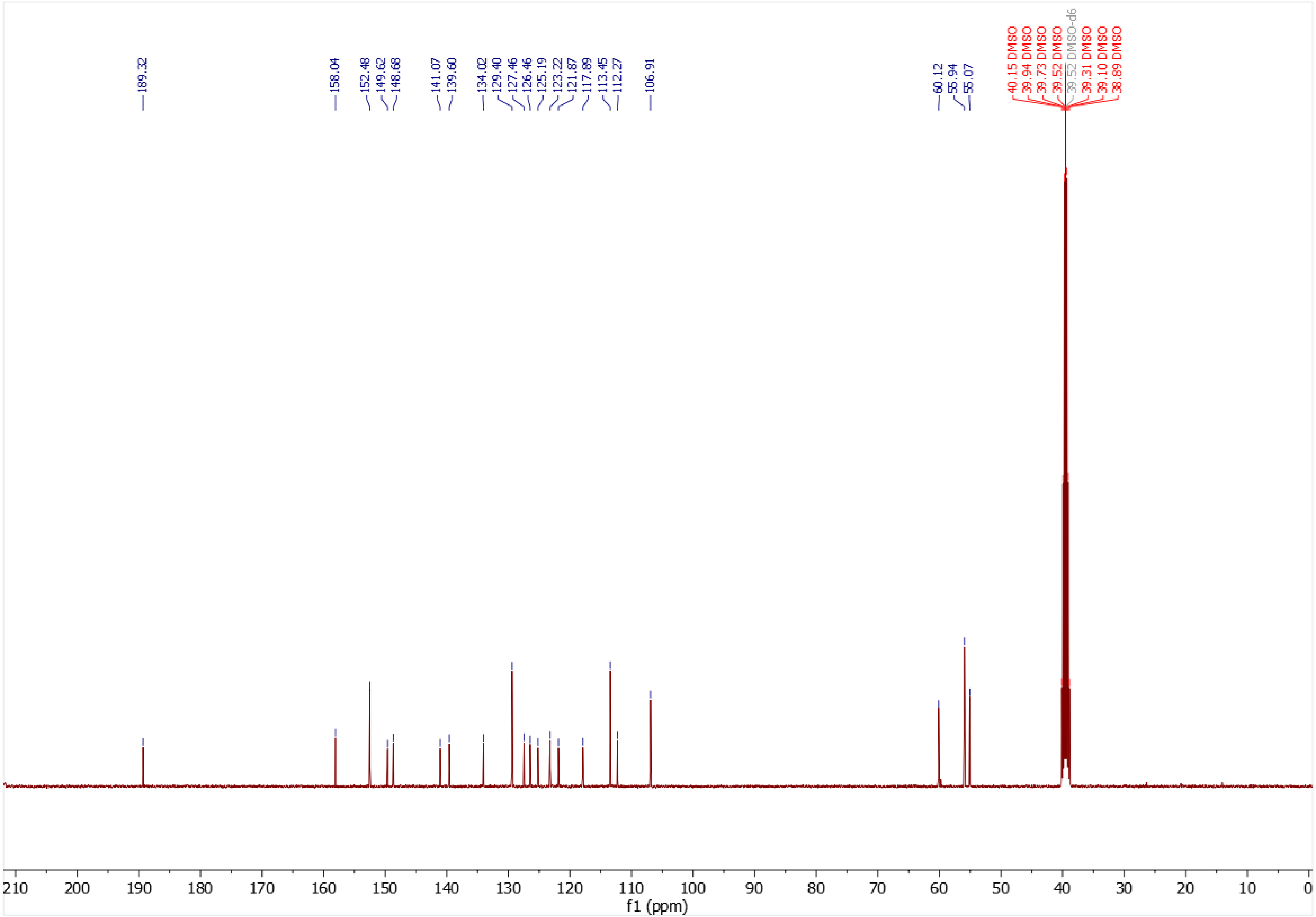
189.32
158.04
152.48
149.62
148.68
141.07
139.60
134.02
129.40
127.46
126.46
125.19
123.22
121.87
117.89
113.45
112.27
106.91
60.12
55.94
55.07
40.15 DMSO
39.94 DMSO
39.73 DMSO
39.52 DMSO
39.52 DMSO-d6
39.31 DMSO
39.10 DMSO
38.89 DMSO
210
200
190
180
170
160
150
140
130
120
110
100
90
80
70
60
50
40
30
20
10
0
f1 (ppm)

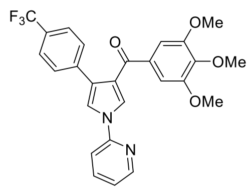
F3C
OMe
O
OMe
OMe
N
N

5

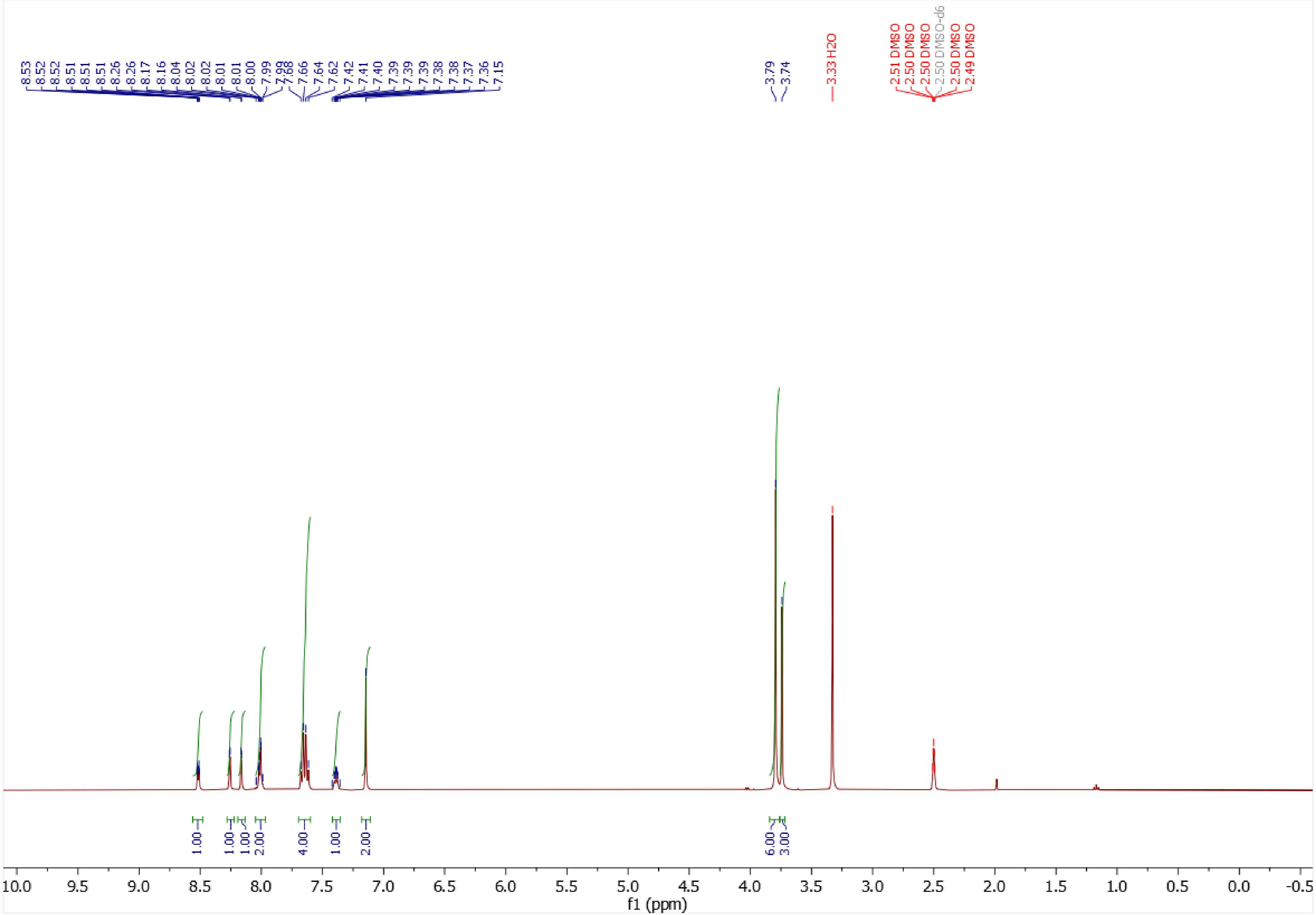
8.53
8.52
8.52
8.51
8.51
8.26
8.26
8.17
8.16
8.04
8.02
8.02
8.01
8.01
8.00
7.99
7.98
7.66
7.64
7.62
7.42
7.41
7.40
7.39
7.39
7.39
7.38
7.37
7.36
7.15
3.79
3.74
3.33 H2O
2.51 DMSO
2.50 DMSO
2.50 DMSO
2.50 DMSO-d6
2.50 DMSO
2.49 DMSO
1.00
1.00
1.00
2.00
4.00
1.00
2.00
6.00
3.00
10.0
9.5
9.0
8.5
8.0
7.5
7.0
6.5
6.0
5.5
5.0
4.5
4.0
3.5
3.0
2.5
2.0
1.5
1.0
0.5
0.0
-0.5
f1 (ppm)

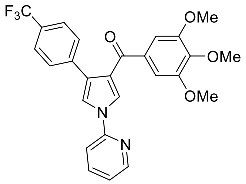
F3C
OMe
O
OMe
OMe
N
N

5

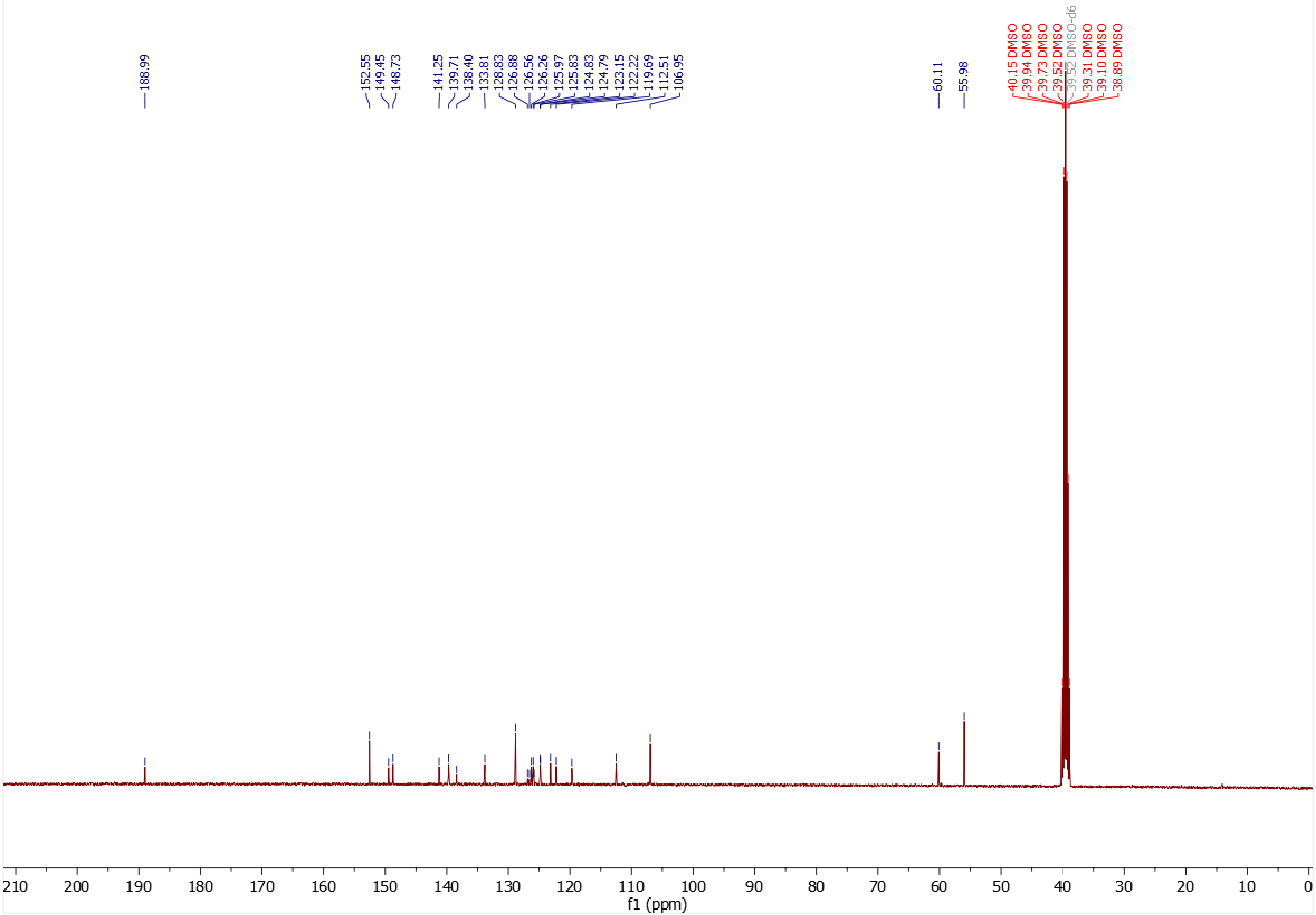
188.99
152.55
149.45
148.73
141.25
139.71
138.40
133.81
128.83
126.88
126.56
126.26
125.97
125.83
124.83
124.79
123.15
122.22
119.69
112.51
106.95
60.11
55.98
40.15 DMSO
39.94 DMSO
39.73 DMSO
39.52 DMSO-d6
39.31 DMSO
39.10 DMSO
38.89 DMSO
210 200 190 180 170 160 150 140 130 120 110 100 90 80 70 60 50 40 30 20 10 0
f1 (ppm)

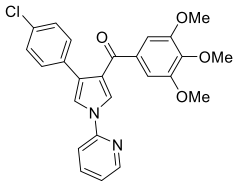

6

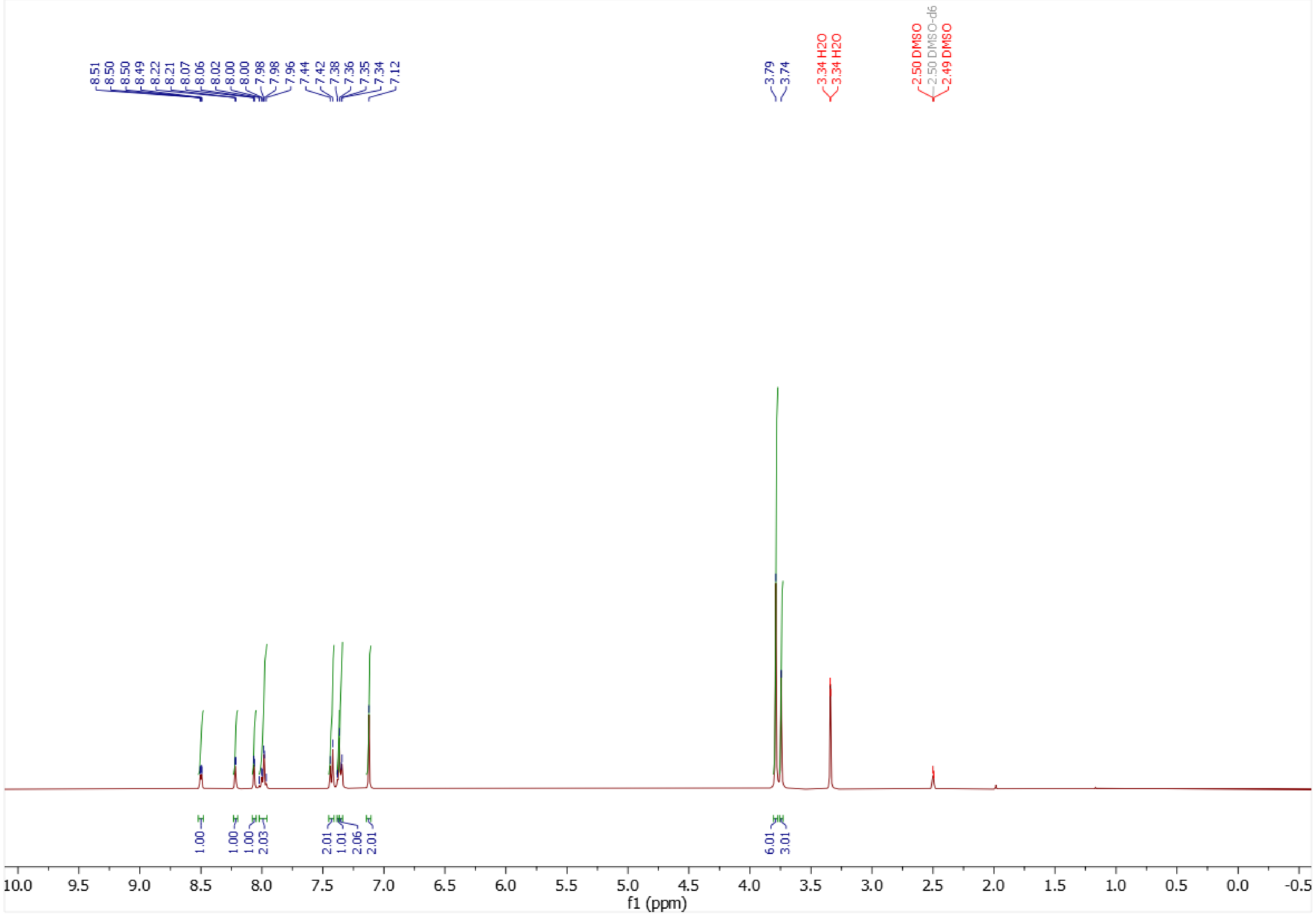

8.51
8.50
8.50
8.49
8.22
8.21
8.07
8.06
8.02
8.00
8.00
7.98
7.98
7.96
7.44
7.42
7.38
7.36
7.35
7.34
7.12
3.79
3.74
3.34 H2O
3.34 H2O
2.50 DMSO
2.50 DMSO-d6
2.49 DMSO
1.00
1.00
1.00
2.03
2.01
1.01
2.06
2.01
6.01
3.01
f1 (ppm)

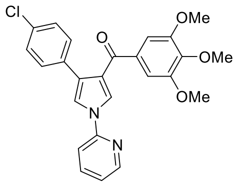
Cl
O
OMe
OMe
OMe
N
N

6

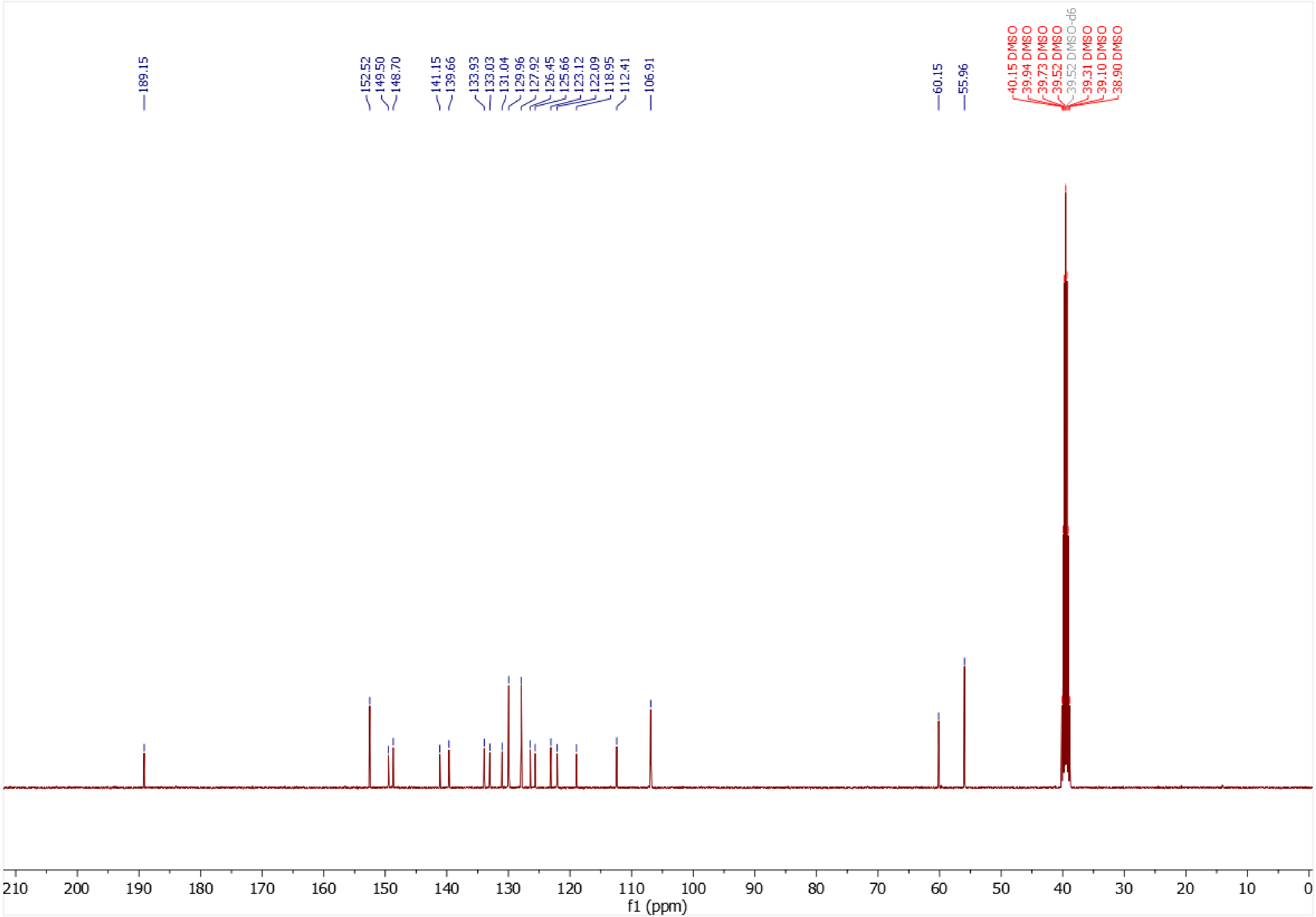
189.15
152.52
149.50
148.70
141.15
139.66
133.93
133.03
131.04
129.96
127.92
126.45
125.66
123.12
122.09
118.95
112.41
106.91
60.15
55.96
40.15 DMSO
39.94 DMSO
39.73 DMSO
39.52 DMSO
39.52 DMSO-d6
39.31 DMSO
39.10 DMSO
38.90 DMSO
210 200 190 180 170 160 150 140 130 120 110 100 90 80 70 60 50 40 30 20 10 0
f1 (ppm)

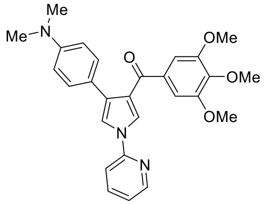
Me
Me–N
O
OMe
OMe
OMe
N
N

7

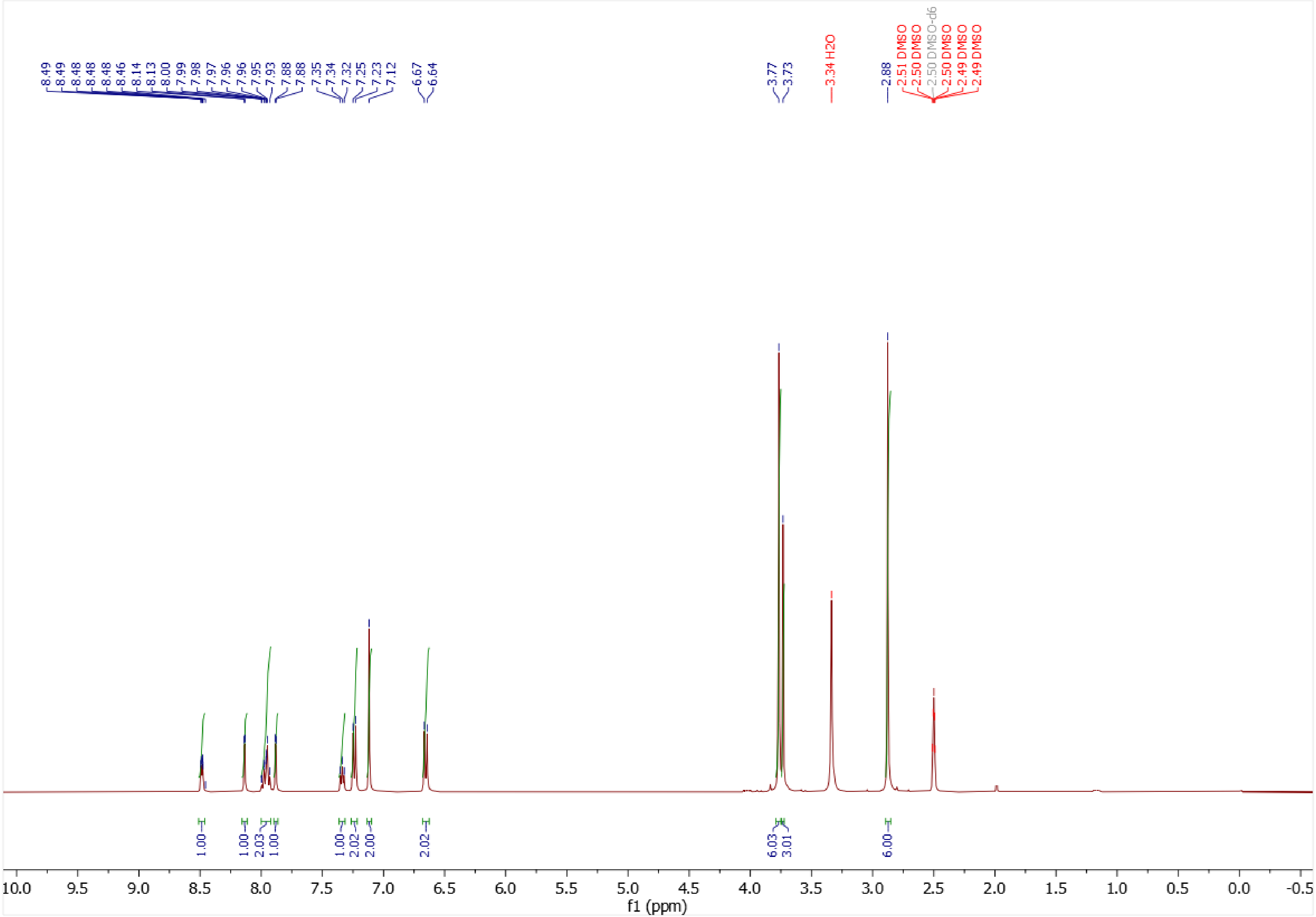
8.49
8.49
8.48
8.48
8.48
8.46
8.14
8.13
8.00
7.99
7.98
7.97
7.96
7.95
7.93
7.88
7.88
7.35
7.34
7.32
7.25
7.23
7.12
6.67
6.64
3.77
3.73
3.34 H2O
2.88
2.51 DMSO
2.50 DMSO
2.50 DMSO-d6
2.50 DMSO
2.49 DMSO
2.49 DMSO
1.00
1.00
2.03
1.00
1.00
2.02
2.00
2.02
6.03
3.01
6.00
10.0
9.5
9.0
8.5
8.0
7.5
7.0
6.5
6.0
5.5
5.0
4.5
4.0
3.5
3.0
2.5
2.0
1.5
1.0
0.5
0.0
-0.5
f1 (ppm)

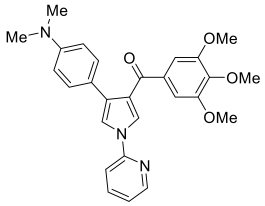
Me
Me
N
O
OMe
OMe
OMe
N
N

7

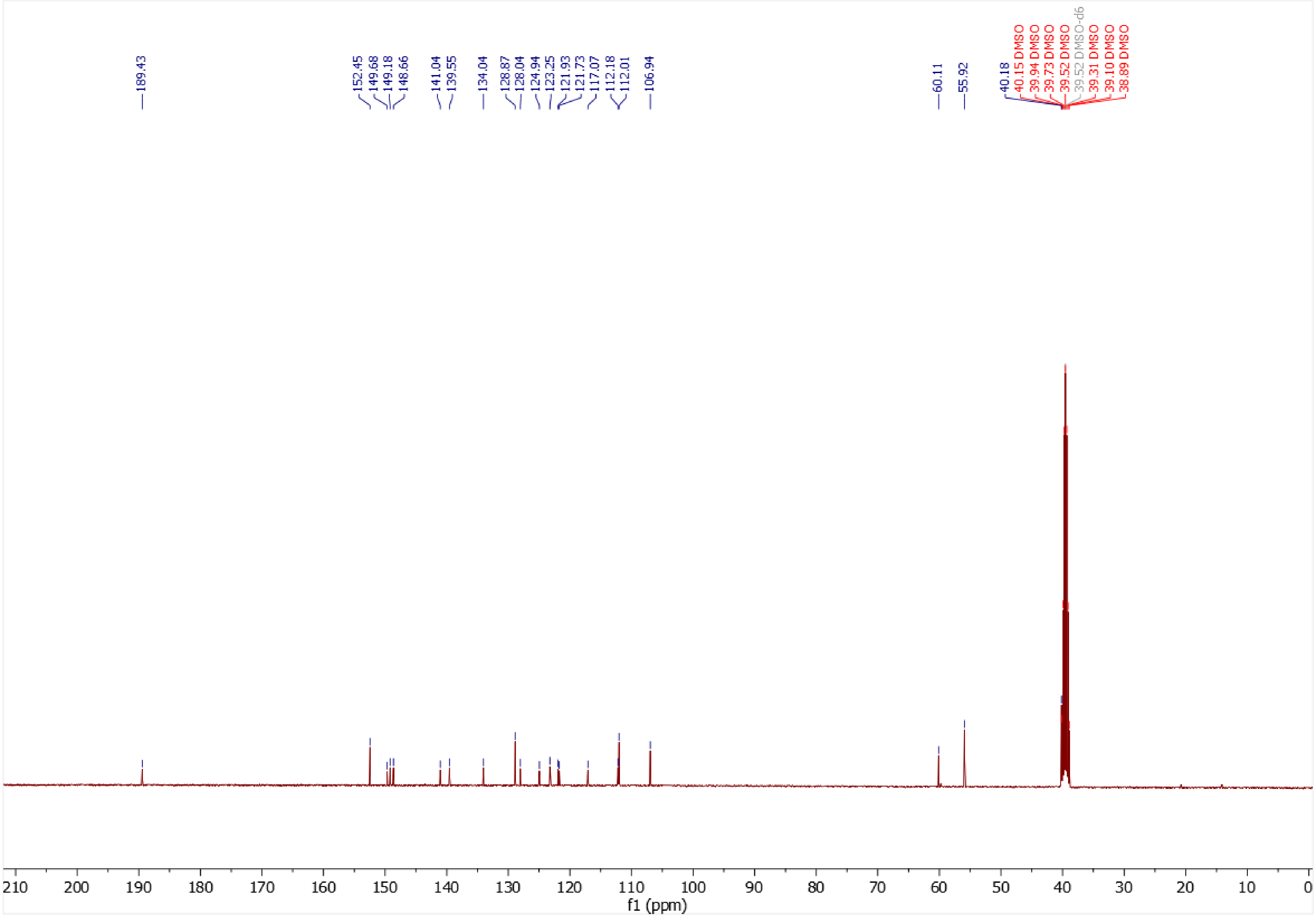
189.43
152.45
149.68
149.18
148.66
141.04
139.55
134.04
128.87
128.04
124.94
123.25
121.93
121.73
117.07
112.18
112.01
106.94
60.11
55.92
40.18
40.15 DMSO
39.94 DMSO
39.73 DMSO
39.52 DMSO
39.52 DMSO-d6
39.31 DMSO
39.10 DMSO
38.89 DMSO
210 200 190 180 170 160 150 140 130 120 110 100 90 80 70 60 50 40 30 20 10 0
f1 (ppm)

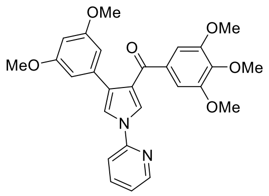

8

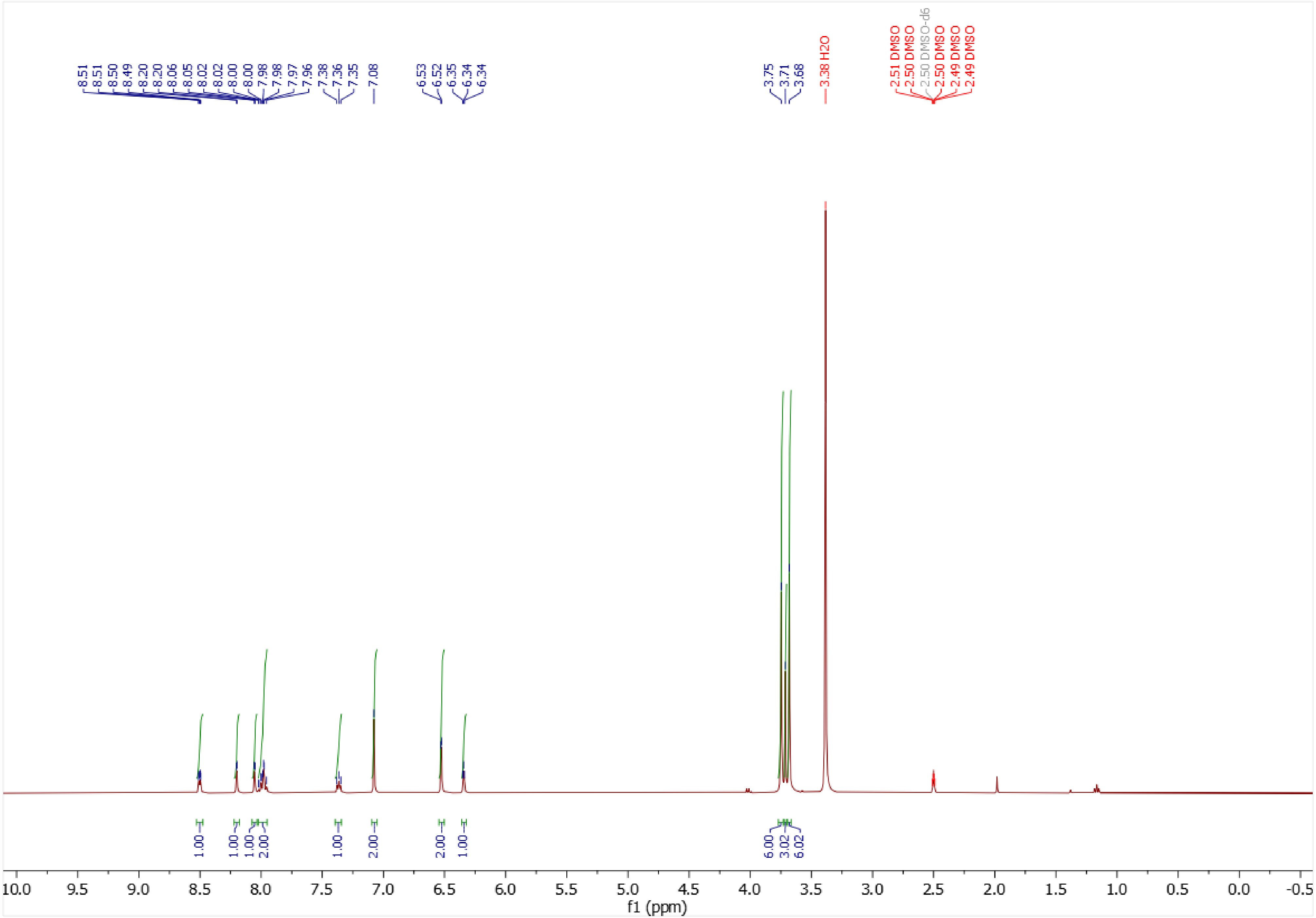

3.38 H2O
2.51 DMSO
2.50 DMSO
2.50 DMSO-d6
2.50 DMSO
2.49 DMSO
2.49 DMSO
f1 (ppm)

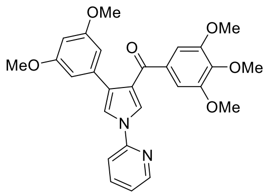
OMe
MeO
O
OMe
OMe
OMe
N
N
8

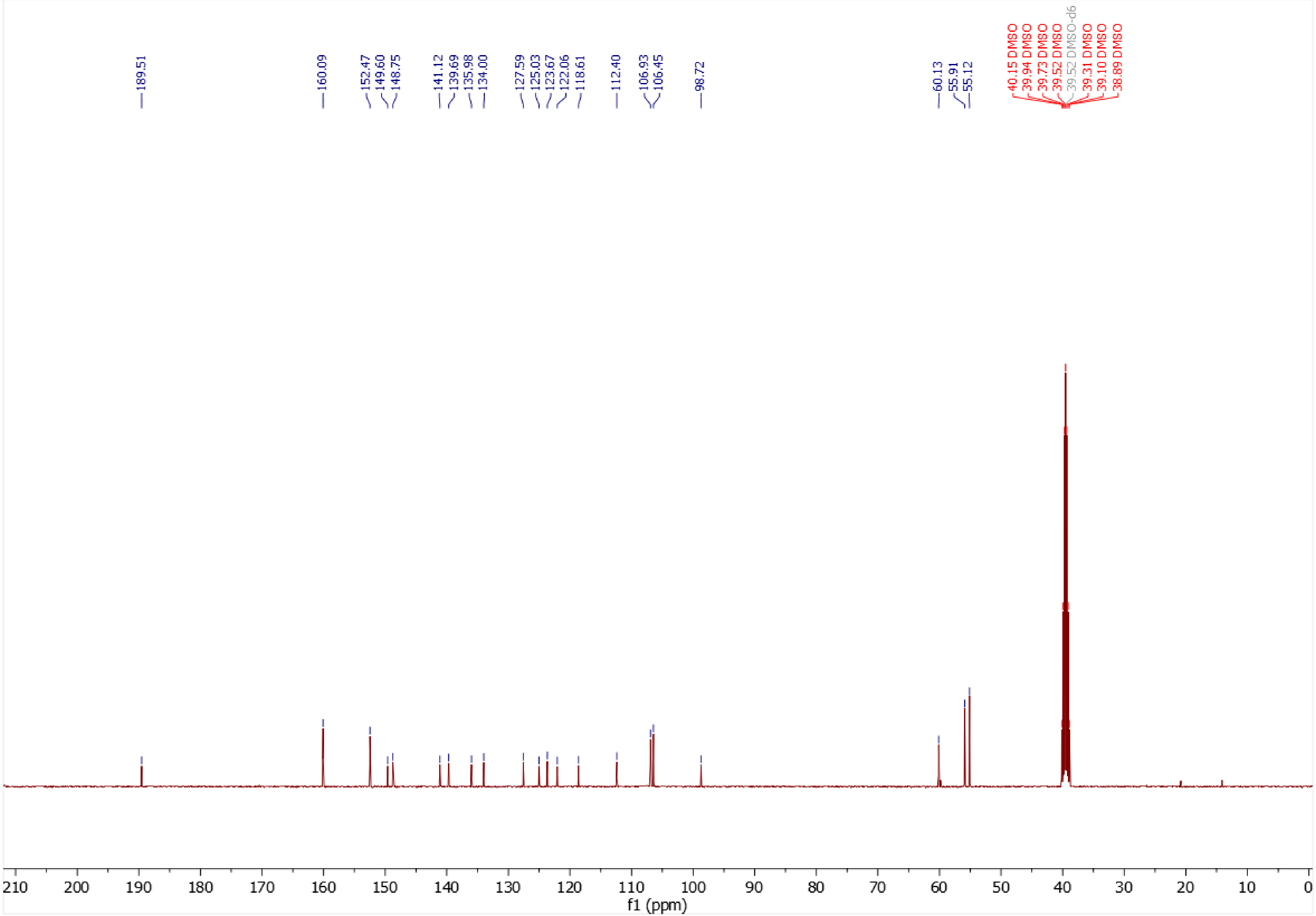
189.51
160.09
152.47
149.60
148.75
141.12
139.69
135.98
134.00
127.59
125.03
123.67
122.06
118.61
112.40
106.93
106.45
98.72
60.13
55.91
55.12
40.15 DMSO
39.94 DMSO
39.73 DMSO
39.52 DMSO
39.52 DMSO-d6
39.31 DMSO
39.10 DMSO
38.89 DMSO
210 200 190 180 170 160 150 140 130 120 110 100 90 80 70 60 50 40 30 20 10 0
f1 (ppm)

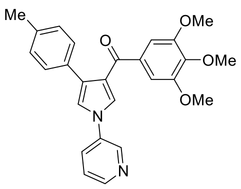

9

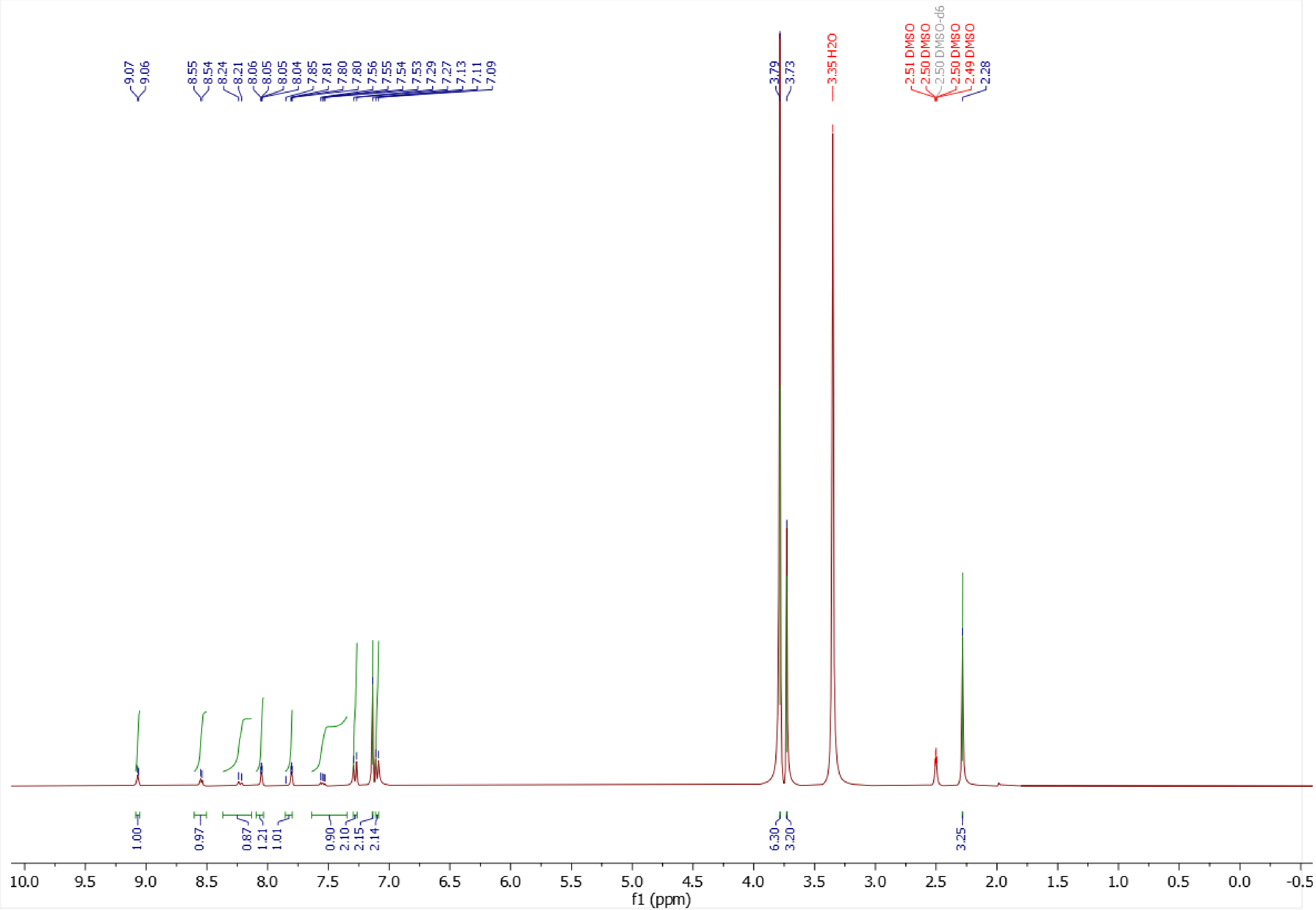

9.07
9.06
8.55
8.54
8.24
8.21
8.06
8.05
8.05
8.04
7.85
7.81
7.80
7.80
7.56
7.55
7.54
7.53
7.29
7.27
7.13
7.11
7.09
3.79
3.73
3.35 H2O
2.51 DMSO
2.50 DMSO
2.50 DMSO-d6
2.50 DMSO
2.49 DMSO
2.28
1.00
0.97
0.87
1.21
1.01
0.90
2.10
2.15
2.14
6.30
3.20
3.25
10.0
9.5
9.0
8.5
8.0
7.5
7.0
6.5
6.0
5.5
5.0
4.5
4.0
3.5
3.0
2.5
2.0
1.5
1.0
0.5
0.0
-0.5
f1 (ppm)

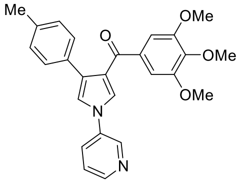
Me
O
OMe
OMe
OMe
N
N

9

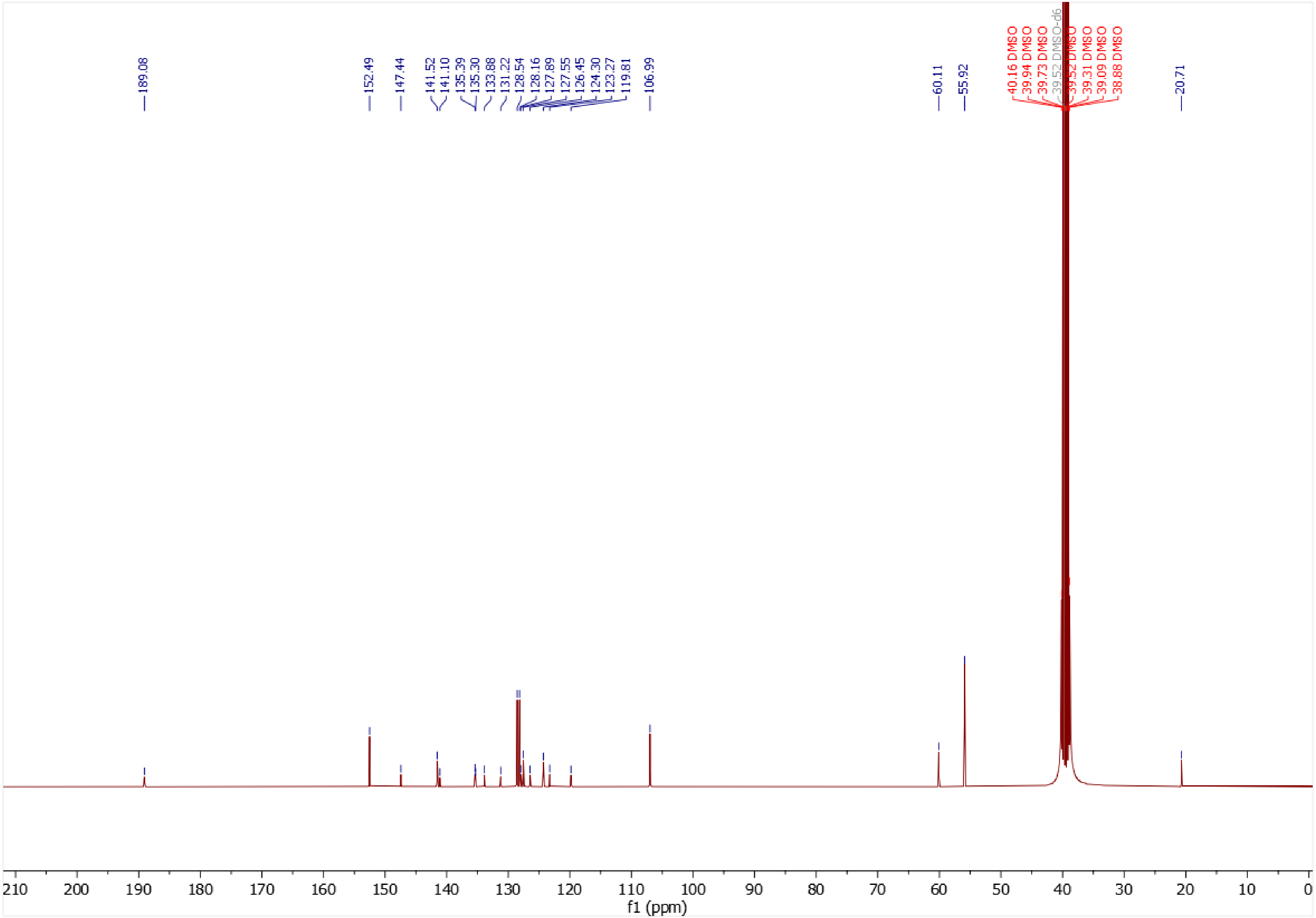
189.08
152.49
147.44
141.52
141.10
135.39
135.30
133.88
131.22
128.54
128.16
127.89
127.55
126.45
124.30
123.27
119.81
106.99
60.11
55.92
40.16 DMSO
39.94 DMSO
39.73 DMSO
39.52 DMSO-d6
39.52 DMSO
39.31 DMSO
39.09 DMSO
38.88 DMSO
20.71
210
200
190
180
170
160
150
140
130
120
110
100
90
80
70
60
50
40
30
20
10
0
f1 (ppm)

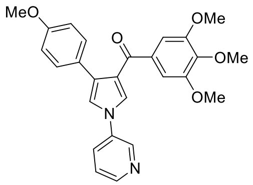
MeO
O
OMe
OMe
OMe
N
N

10

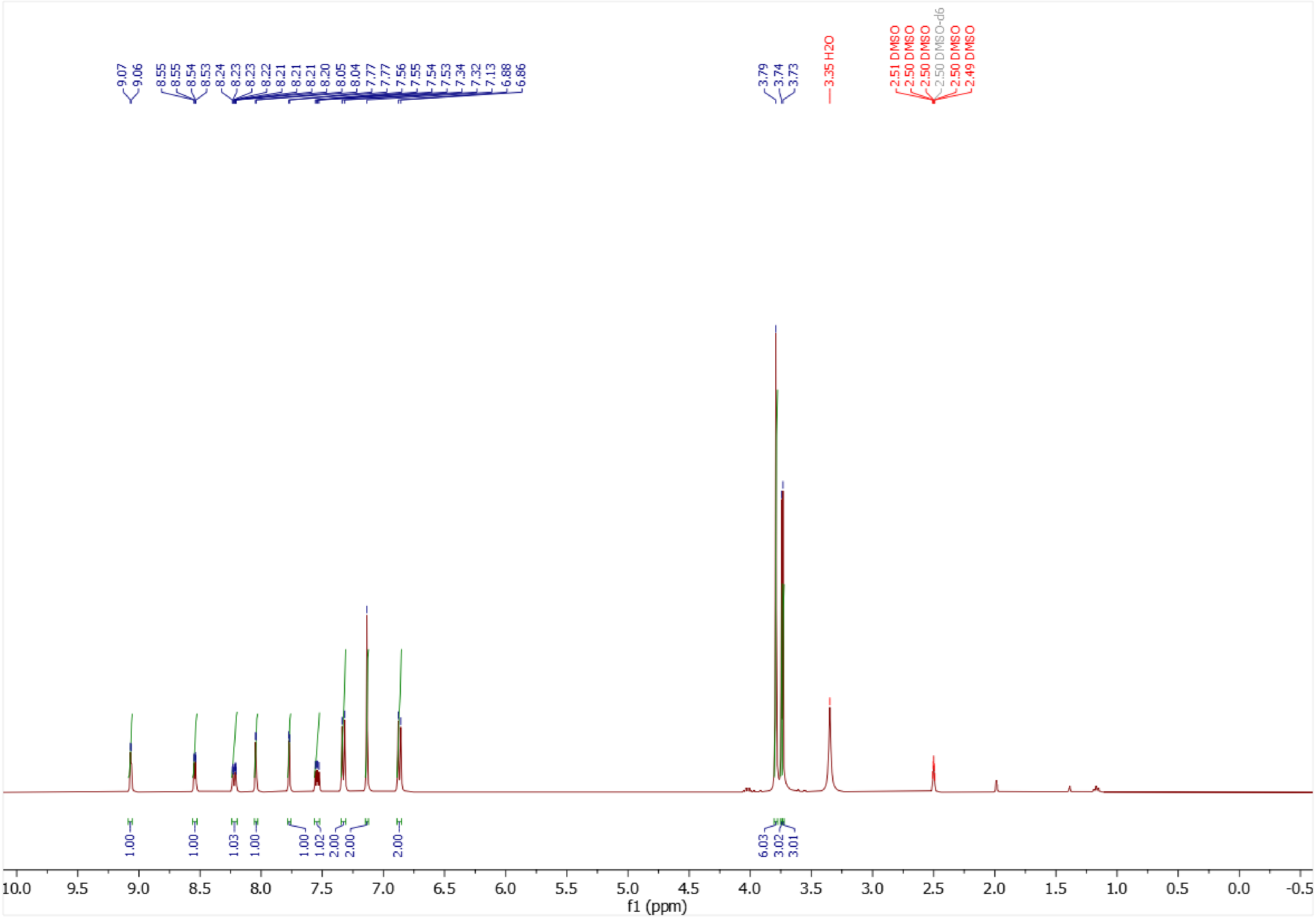
9.07
9.06
8.55
8.55
8.54
8.53
8.24
8.23
8.23
8.22
8.21
8.21
8.21
8.20
8.05
8.04
7.77
7.77
7.56
7.55
7.54
7.53
7.34
7.32
7.13
6.88
6.86
3.79
3.74
3.73
3.35 H2O
2.51 DMSO
2.50 DMSO
2.50 DMSO
2.50 DMSO-d6
2.50 DMSO
2.49 DMSO
1.00
1.00
1.03
1.00
1.00
1.02
2.00
2.00
2.00
6.03
3.02
3.01
10.0
9.5
9.0
8.5
8.0
7.5
7.0
6.5
6.0
5.5
5.0
4.5
4.0
3.5
3.0
2.5
2.0
1.5
1.0
0.5
0.0
-0.5
f1 (ppm)

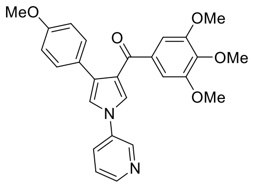
MeO
O
OMe
OMe
OMe
N
N

10

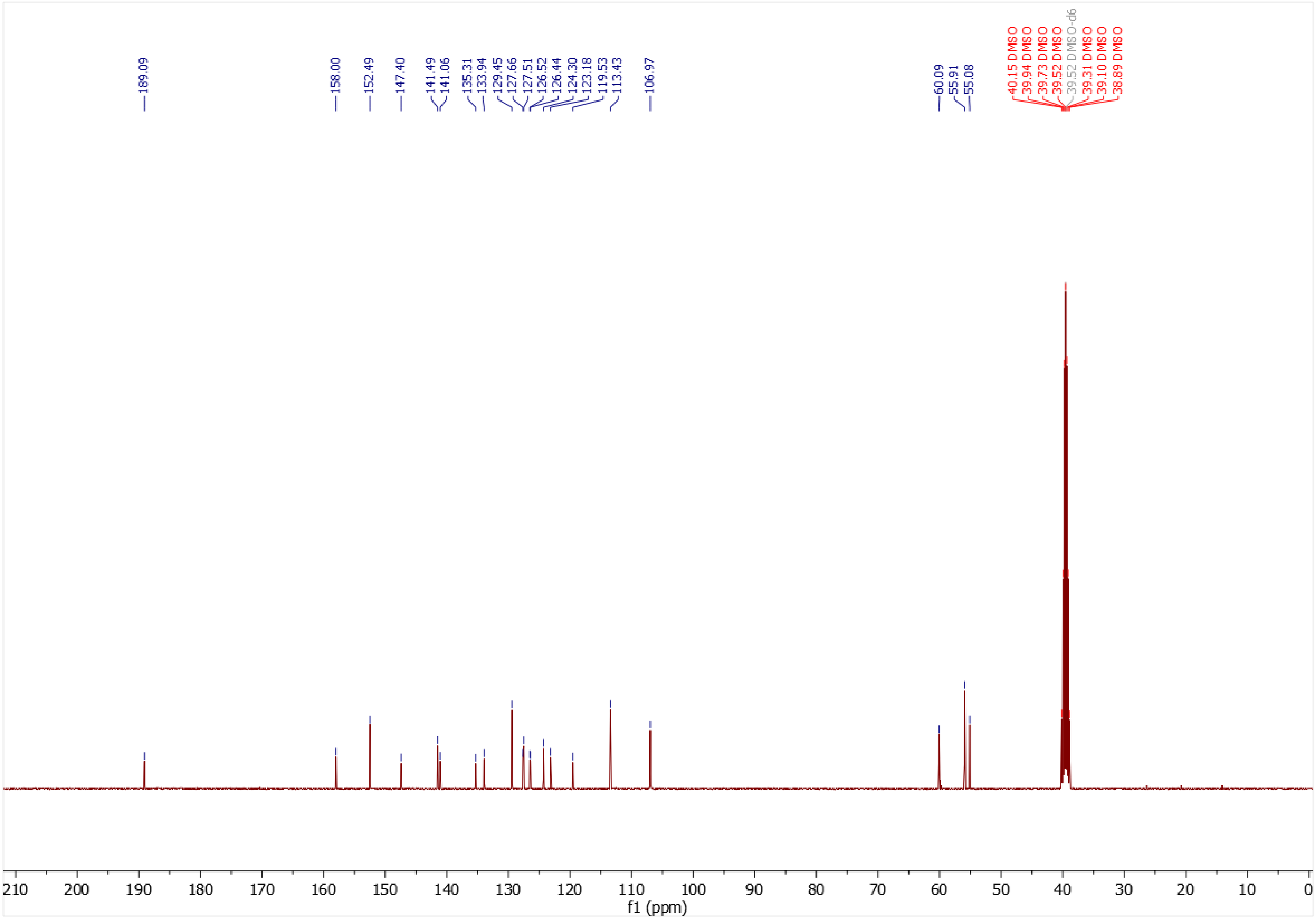
189.09
158.00
152.49
147.40
141.49
141.06
135.31
133.94
129.45
127.66
127.51
126.52
126.44
124.30
123.18
119.53
113.43
106.97
60.09
55.91
55.08
40.15 DMSO
39.94 DMSO
39.73 DMSO
39.52 DMSO
39.52 DMSO-d6
39.31 DMSO
39.10 DMSO
38.89 DMSO
210
200
190
180
170
160
150
140
130
120
110
100
90
80
70
60
50
40
30
20
10
0
f1 (ppm)

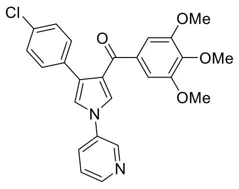

11

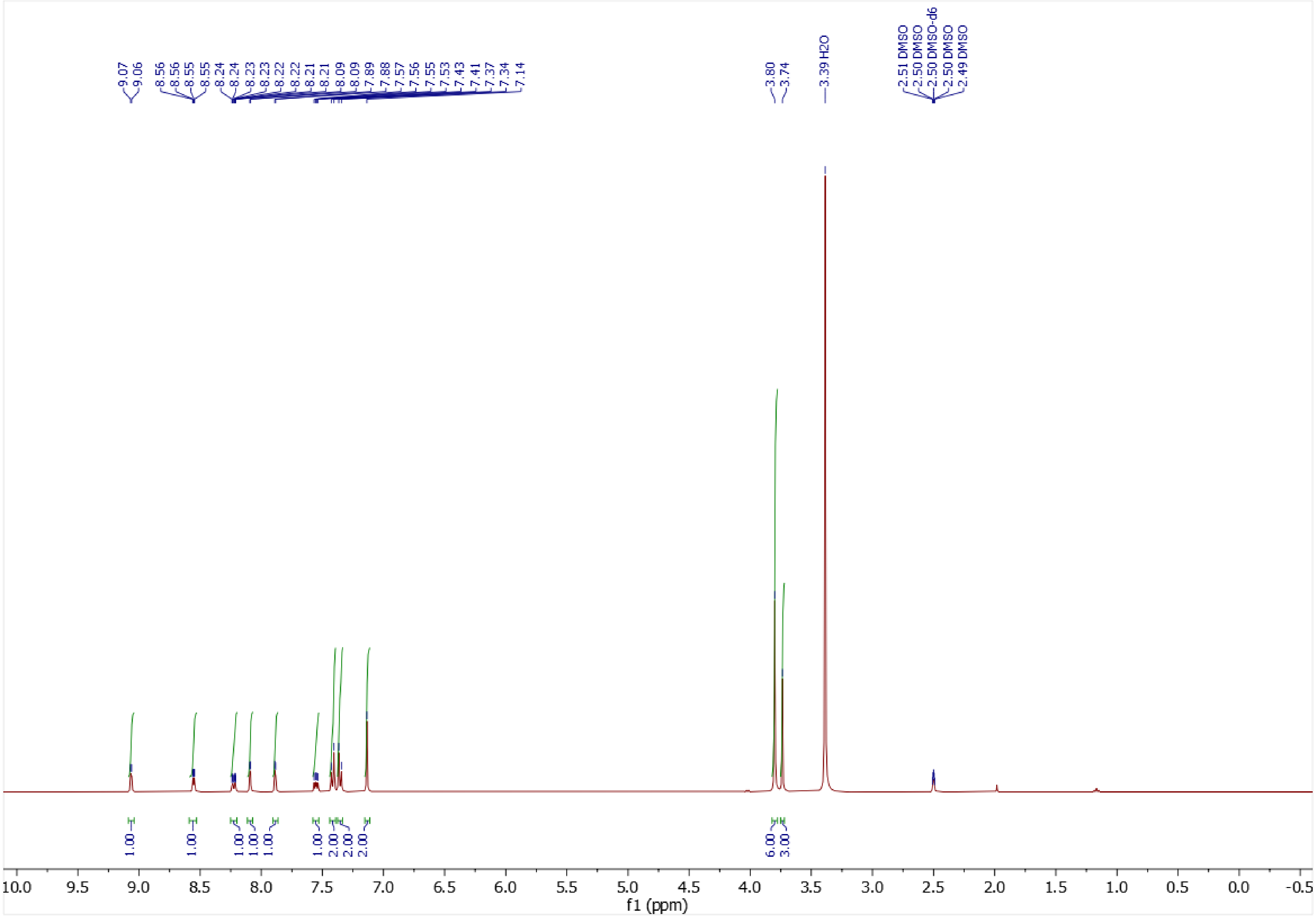

9.07
9.06
8.56
8.56
8.55
8.55
8.24
8.24
8.23
8.23
8.22
8.22
8.21
8.21
8.09
8.09
7.89
7.88
7.57
7.56
7.55
7.53
7.43
7.41
7.37
7.34
7.14
3.80
3.74
3.39 H2O
2.51 DMSO
2.50 DMSO
2.50 DMSO-d6
2.50 DMSO
2.49 DMSO
1.00
1.00
1.00
1.00
1.00
1.00
2.00
2.00
2.00
6.00
3.00
10.0
9.5
9.0
8.5
8.0
7.5
7.0
6.5
6.0
5.5
5.0
4.5
4.0
3.5
3.0
2.5
2.0
1.5
1.0
0.5
0.0
-0.5
f1 (ppm)

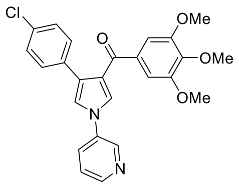
Cl
O
OMe
OMe
OMe
N
N

11

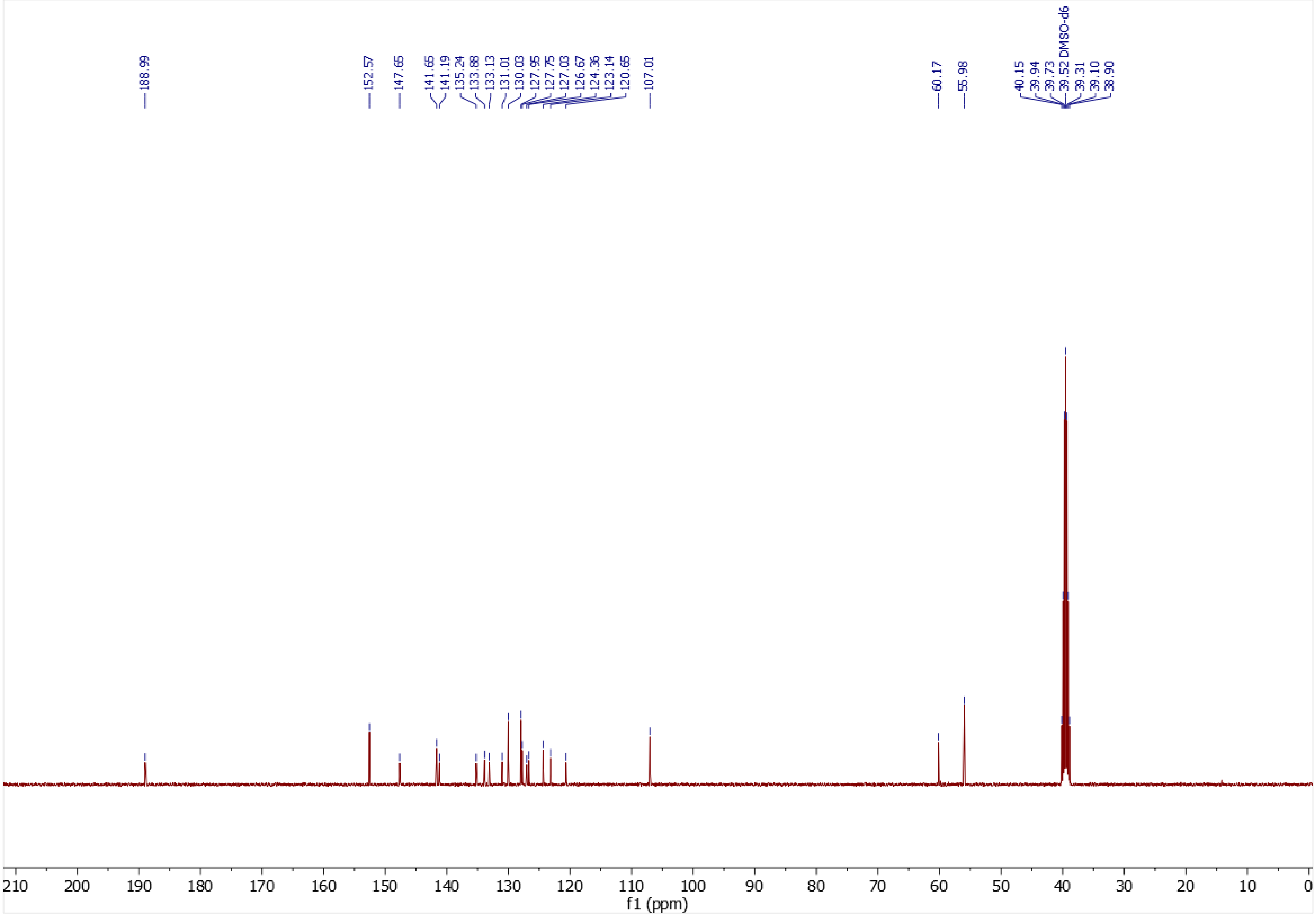
188.99
152.57
147.65
141.65
141.19
135.24
133.88
133.13
131.01
130.03
127.95
127.75
127.03
126.67
124.36
123.14
120.65
107.01
60.17
55.98
40.15
39.94
39.73
39.52 DMSO-d6
39.31
39.10
38.90
210 200 190 180 170 160 150 140 130 120 110 100 90 80 70 60 50 40 30 20 10 0
f1 (ppm)

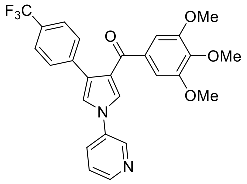
F3C
O
OMe
OMe
OMe
N
N

12

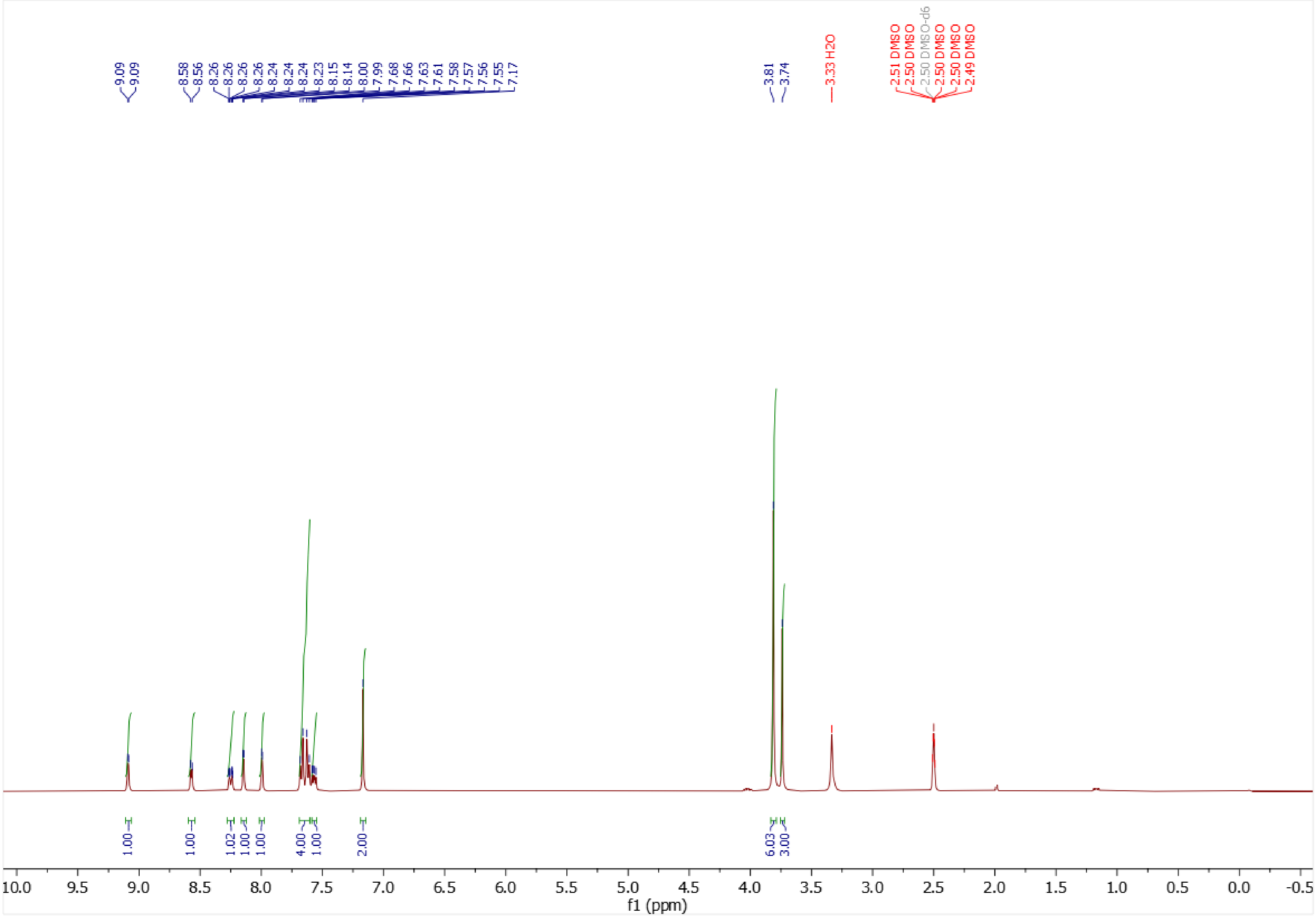
9.09
9.09
8.58
8.56
8.26
8.26
8.26
8.26
8.24
8.24
8.24
8.23
8.15
8.14
8.00
7.99
7.68
7.66
7.63
7.61
7.58
7.57
7.56
7.55
7.17
3.81
3.74
3.33 H2O
2.51 DMSO
2.50 DMSO
2.50 DMSO-d6
2.50 DMSO
2.50 DMSO
2.49 DMSO
1.00
1.00
1.02
1.00
1.00
4.00
1.00
2.00
6.03
3.00
10.0
9.5
9.0
8.5
8.0
7.5
7.0
6.5
6.0
5.5
5.0
4.5
4.0
3.5
3.0
2.5
2.0
1.5
1.0
0.5
0.0
-0.5
f1 (ppm)

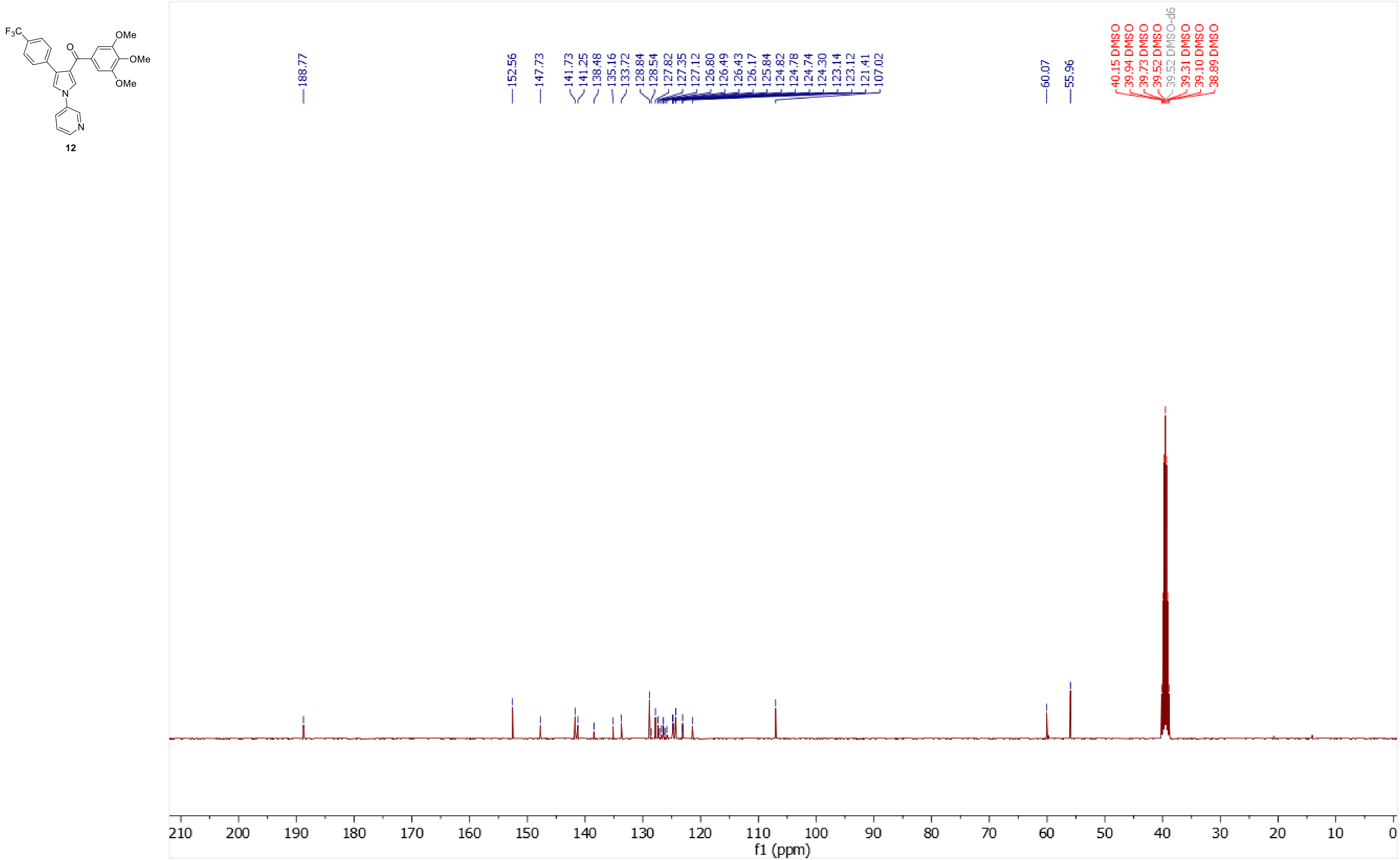
F3C
OMe
O
OMe
OMe
N
N
12
188.77
152.56
147.73
141.73
141.25
138.48
135.16
133.72
128.84
128.54
127.82
127.35
127.12
126.80
126.49
126.43
126.17
125.84
124.82
124.78
124.74
124.30
123.14
123.12
121.41
107.02
60.07
55.96
40.15 DMSO
39.94 DMSO
39.73 DMSO
39.52 DMSO
39.52 DMSO-d6
39.31 DMSO
39.10 DMSO
38.89 DMSO
210 200 190 180 170 160 150 140 130 120 110 100 90 80 70 60 50 40 30 20 10 0
f1 (ppm)

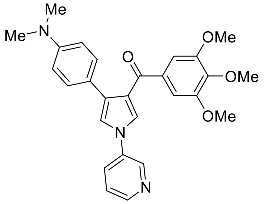

13

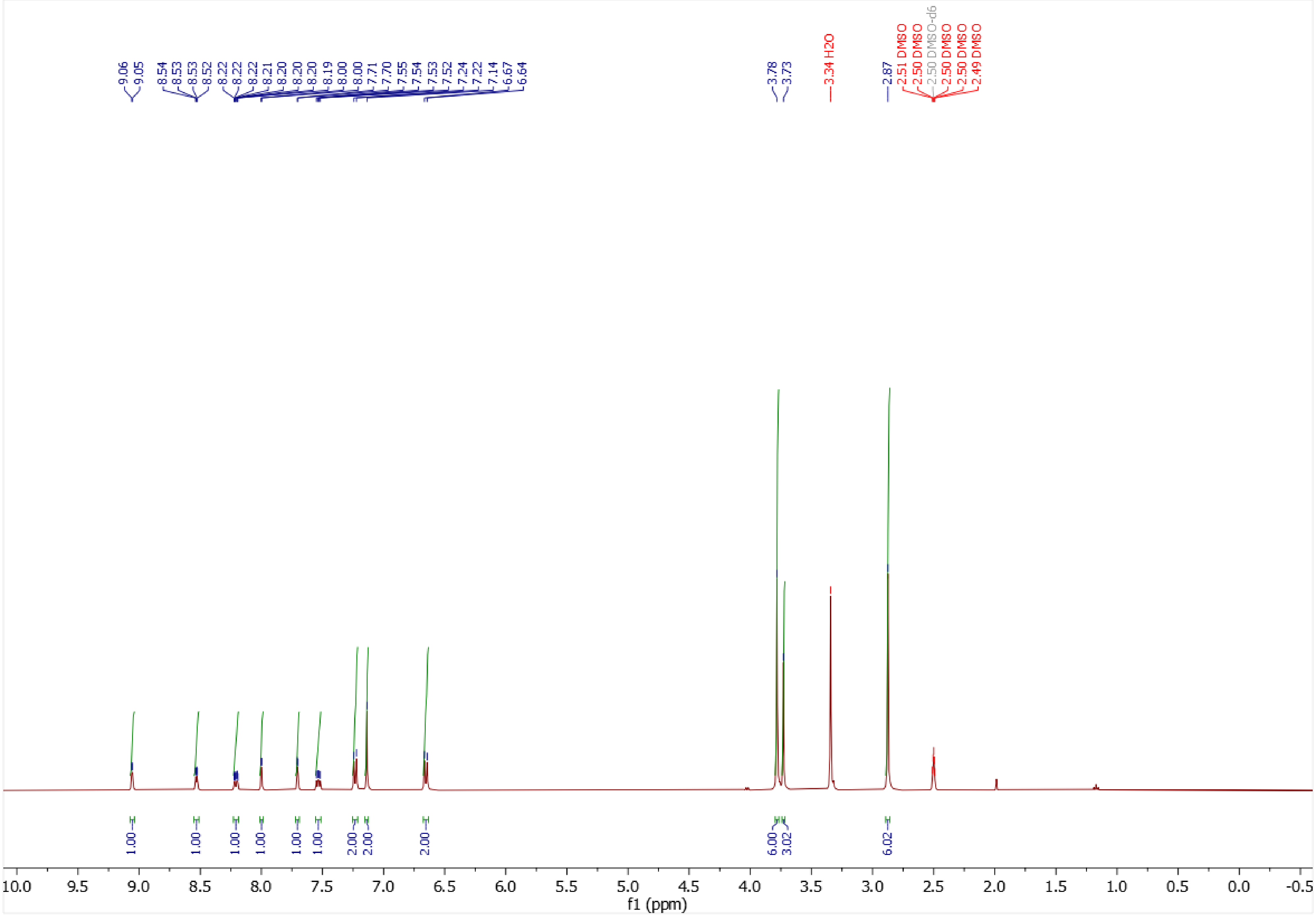
9.06
9.05
8.54
8.53
8.53
8.52
8.22
8.22
8.22
8.21
8.20
8.20
8.20
8.19
8.00
8.00
7.71
7.70
7.55
7.54
7.53
7.52
7.24
7.22
7.14
6.67
6.64
3.78
3.73
3.34 H2O
2.87
2.51 DMSO
2.50 DMSO
2.50 DMSO-d6
2.50 DMSO
2.50 DMSO
2.49 DMSO
1.00
1.00
1.00
1.00
1.00
1.00
2.00
2.00
2.00
6.00
3.02
6.02
10.0 9.5 9.0 8.5 8.0 7.5 7.0 6.5 6.0 5.5 5.0 4.5 4.0 3.5 3.0 2.5 2.0 1.5 1.0 0.5 0.0 -0.5
f1 (ppm)

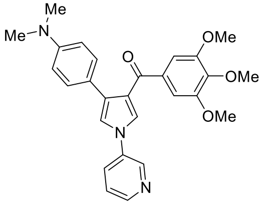

Me
Me
N
O
OMe
OMe
OMe
N
N

13

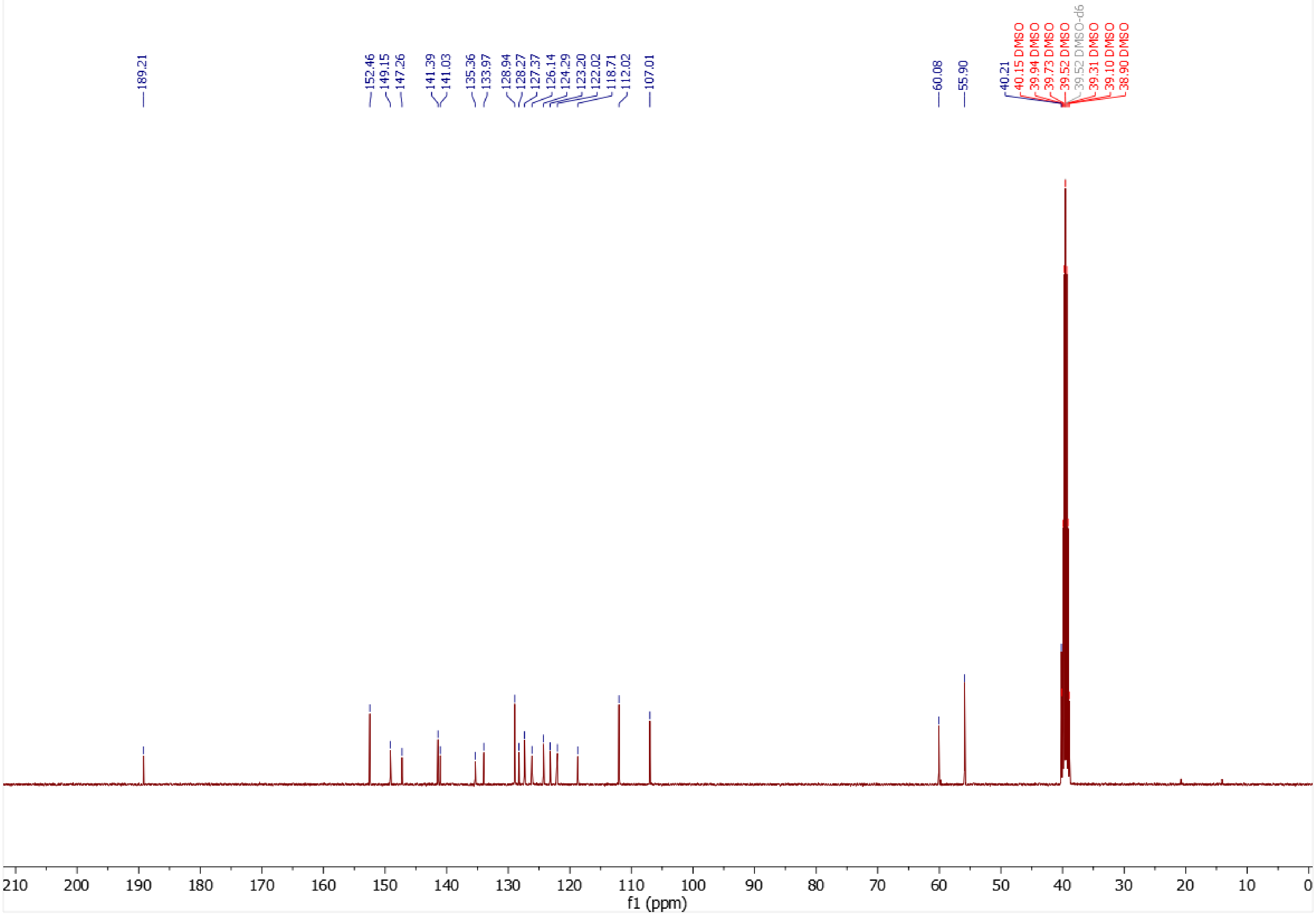

189.21
152.46
149.15
147.26
141.39
141.03
135.36
133.97
128.94
128.27
127.37
126.14
124.29
123.20
122.02
118.71
112.02
107.01
60.08
55.90
40.21
40.15 DMSO
39.94 DMSO
39.73 DMSO
39.52 DMSO
39.52 DMSO-d6
39.31 DMSO
39.10 DMSO
38.90 DMSO
210 200 190 180 170 160 150 140 130 120 110 100 90 80 70 60 50 40 30 20 10 0
f1 (ppm)

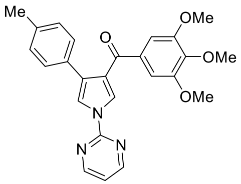

14

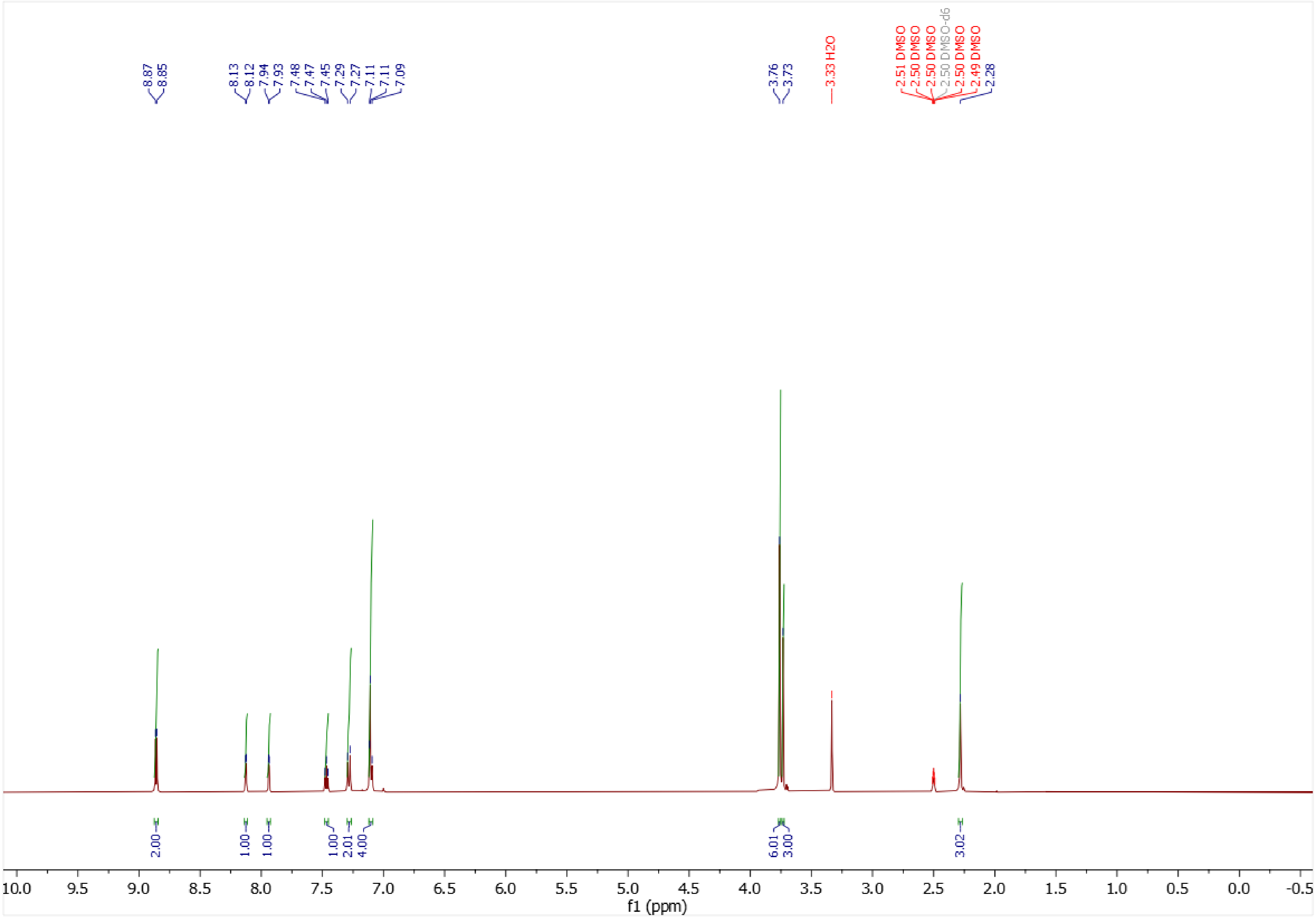

8.87
8.85
8.13
8.12
7.94
7.93
7.48
7.47
7.45
7.29
7.27
7.11
7.11
7.09
3.76
3.73
3.33 H2O
2.51 DMSO
2.50 DMSO
2.50 DMSO
2.50 DMSO-d6
2.50 DMSO
2.49 DMSO
2.28
2.00
1.00
1.00
1.00
2.01
4.00
6.01
3.00
3.02
f1 (ppm)

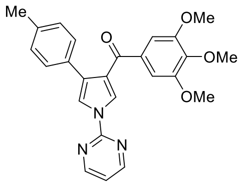
Me
O
OMe
OMe
OMe
N
N
N

14

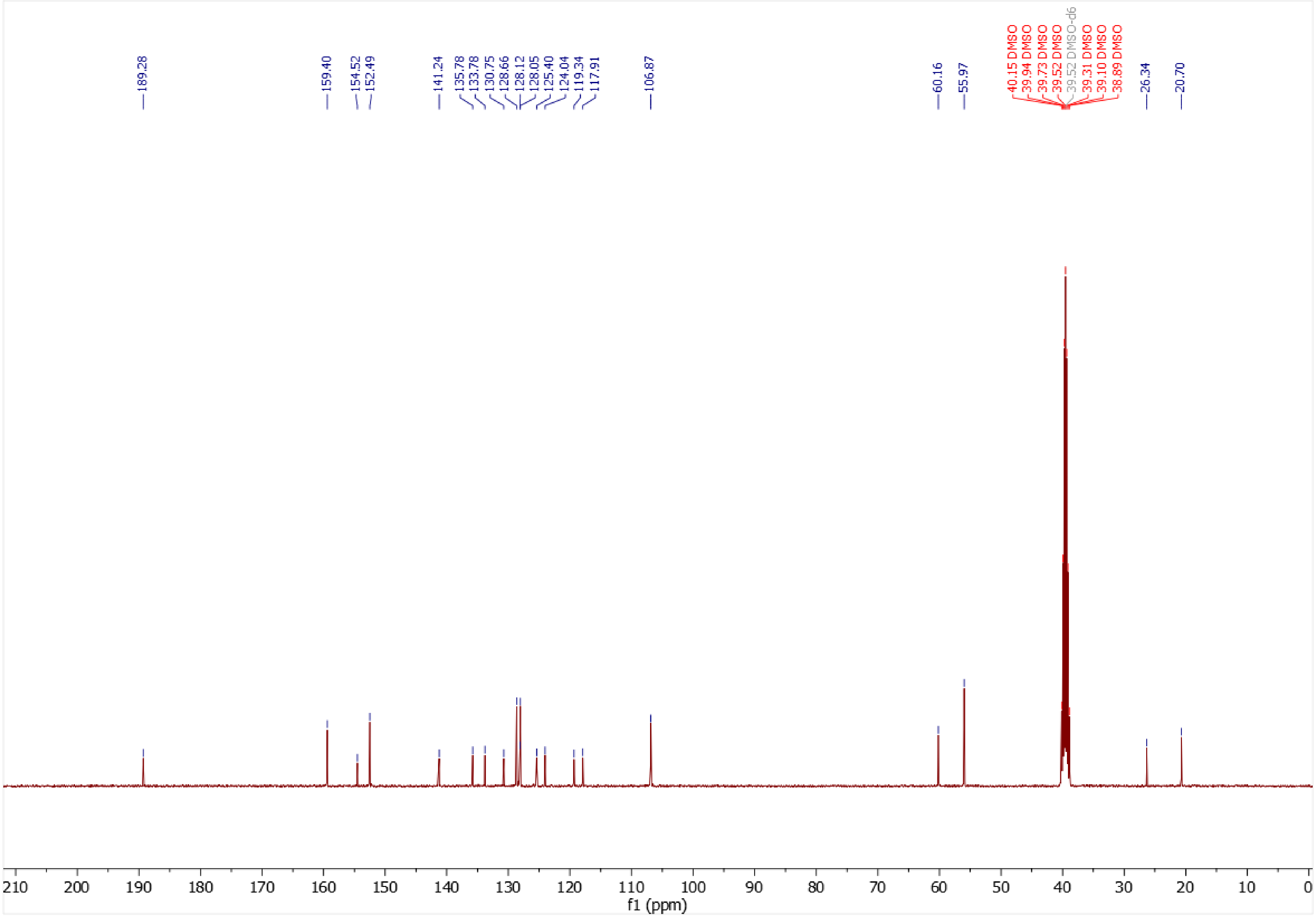
189.28
159.40
154.52
152.49
141.24
135.78
133.78
130.75
128.66
128.12
128.05
125.40
124.04
119.34
117.91
106.87
60.16
55.97
40.15 DMSO
39.94 DMSO
39.73 DMSO
39.52 DMSO
39.52 DMSO-d6
39.31 DMSO
39.10 DMSO
38.89 DMSO
26.34
20.70
210 200 190 180 170 160 150 140 130 120 110 100 90 80 70 60 50 40 30 20 10 0
f1 (ppm)

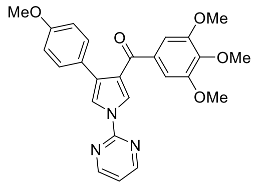
MeO
O
OMe
OMe
OMe
N
N
N

15

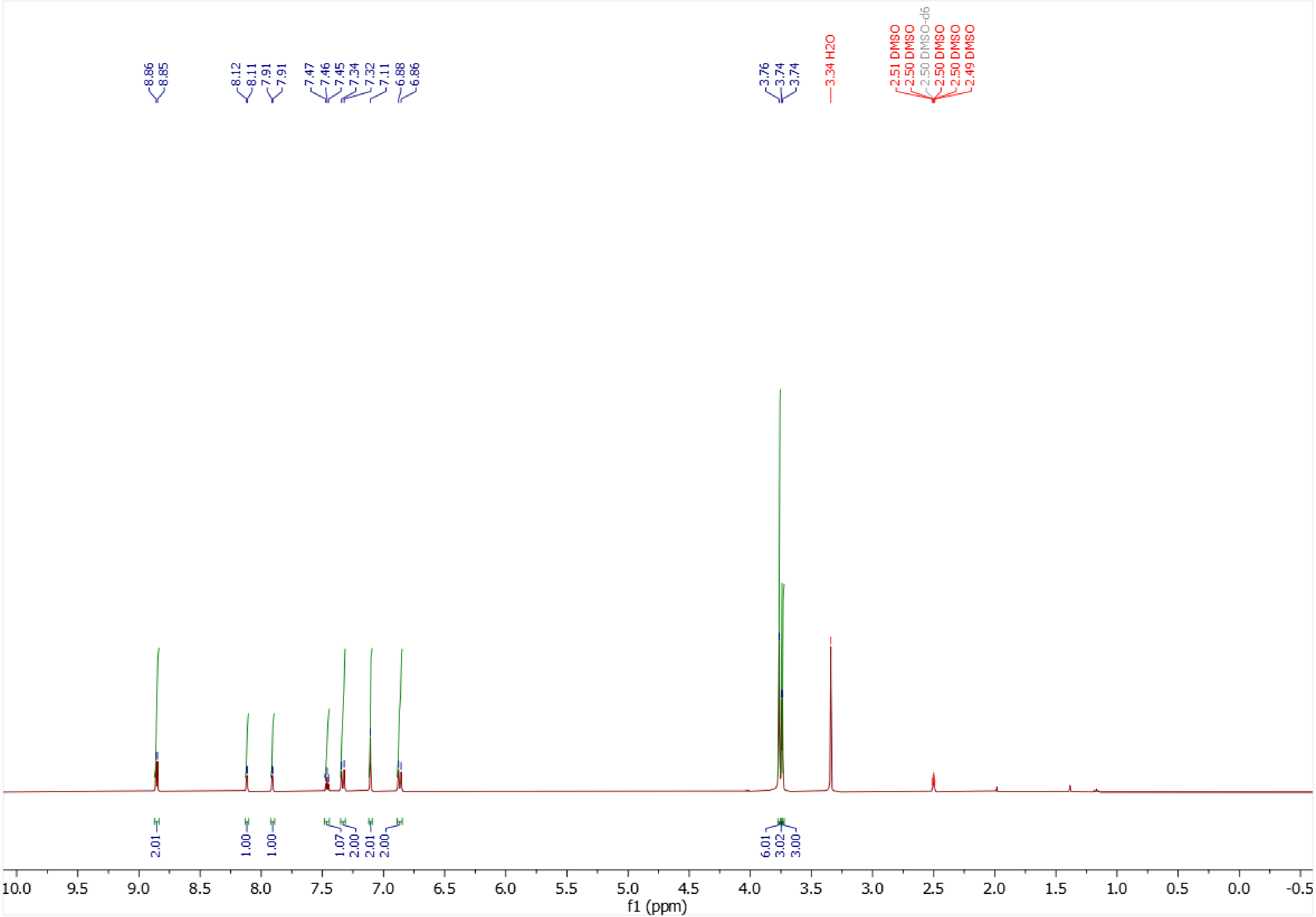
8.86
8.85
8.12
8.11
7.91
7.91
7.47
7.46
7.45
7.34
7.32
7.11
6.88
6.86
3.76
3.74
3.74
3.34 H2O
2.51 DMSO
2.50 DMSO
2.50 DMSO-d6
2.50 DMSO
2.50 DMSO
2.49 DMSO
2.01
1.00
1.00
1.07
2.00
2.01
2.00
6.01
3.02
3.00
10.0 9.5 9.0 8.5 8.0 7.5 7.0 6.5 6.0 5.5 5.0 4.5 4.0 3.5 3.0 2.5 2.0 1.5 1.0 0.5 0.0 -0.5
f1 (ppm)

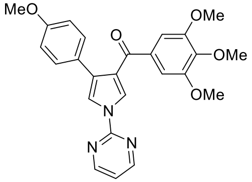
MeO
O
OMe
OMe
OMe
N
N
N

15

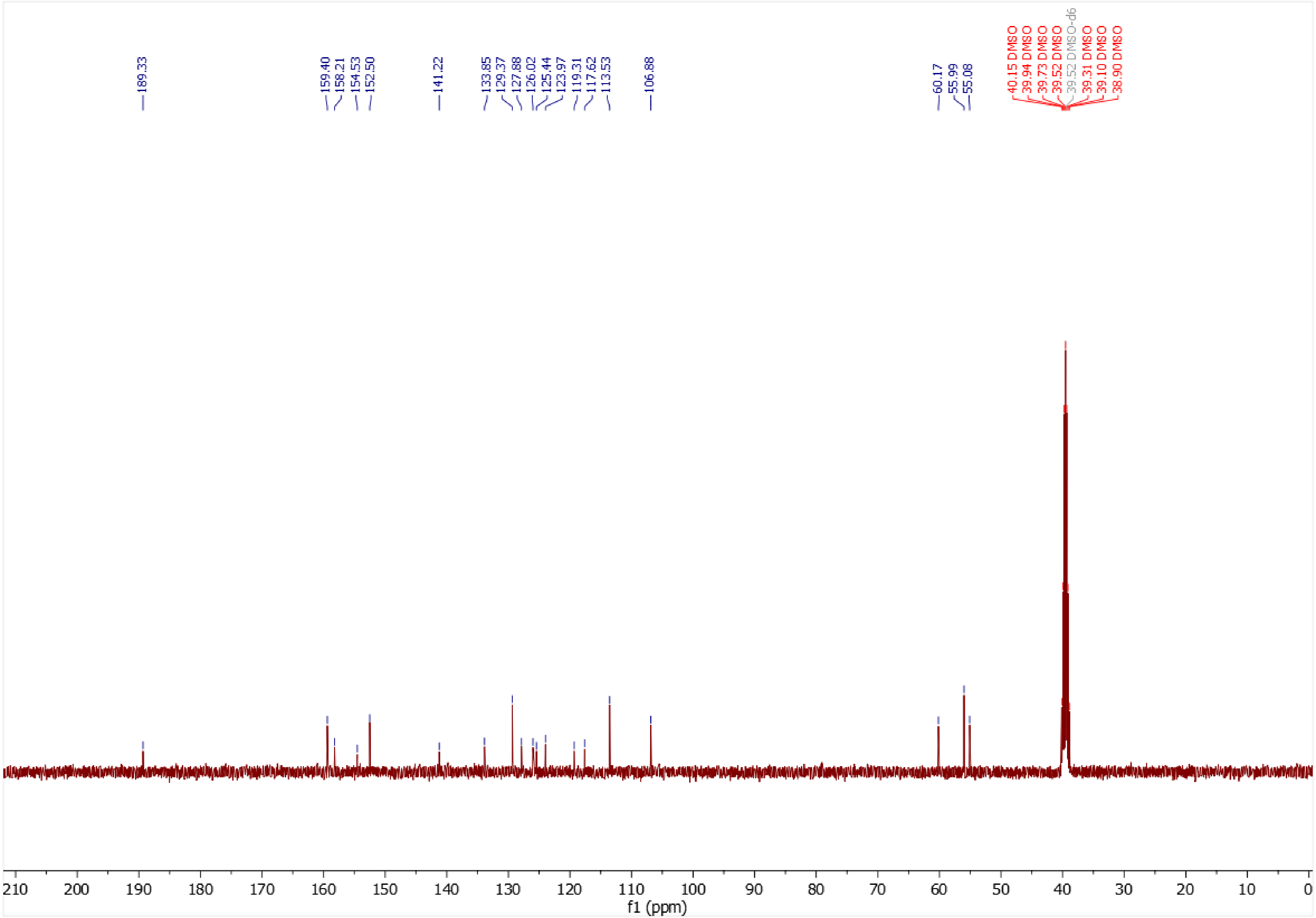
189.33
159.40
158.21
154.53
152.50
141.22
133.85
129.37
127.88
126.02
125.44
123.97
119.31
117.62
113.53
106.88
60.17
55.99
55.08
40.15 DMSO
39.94 DMSO
39.73 DMSO
39.52 DMSO
39.52 DMSO-d6
39.31 DMSO
39.10 DMSO
38.90 DMSO
210 200 190 180 170 160 150 140 130 120 110 100 90 80 70 60 50 40 30 20 10 0
f1 (ppm)

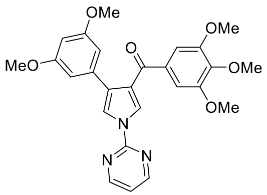
OMe
MeO
O
OMe
OMe
OMe
N
N
N

16

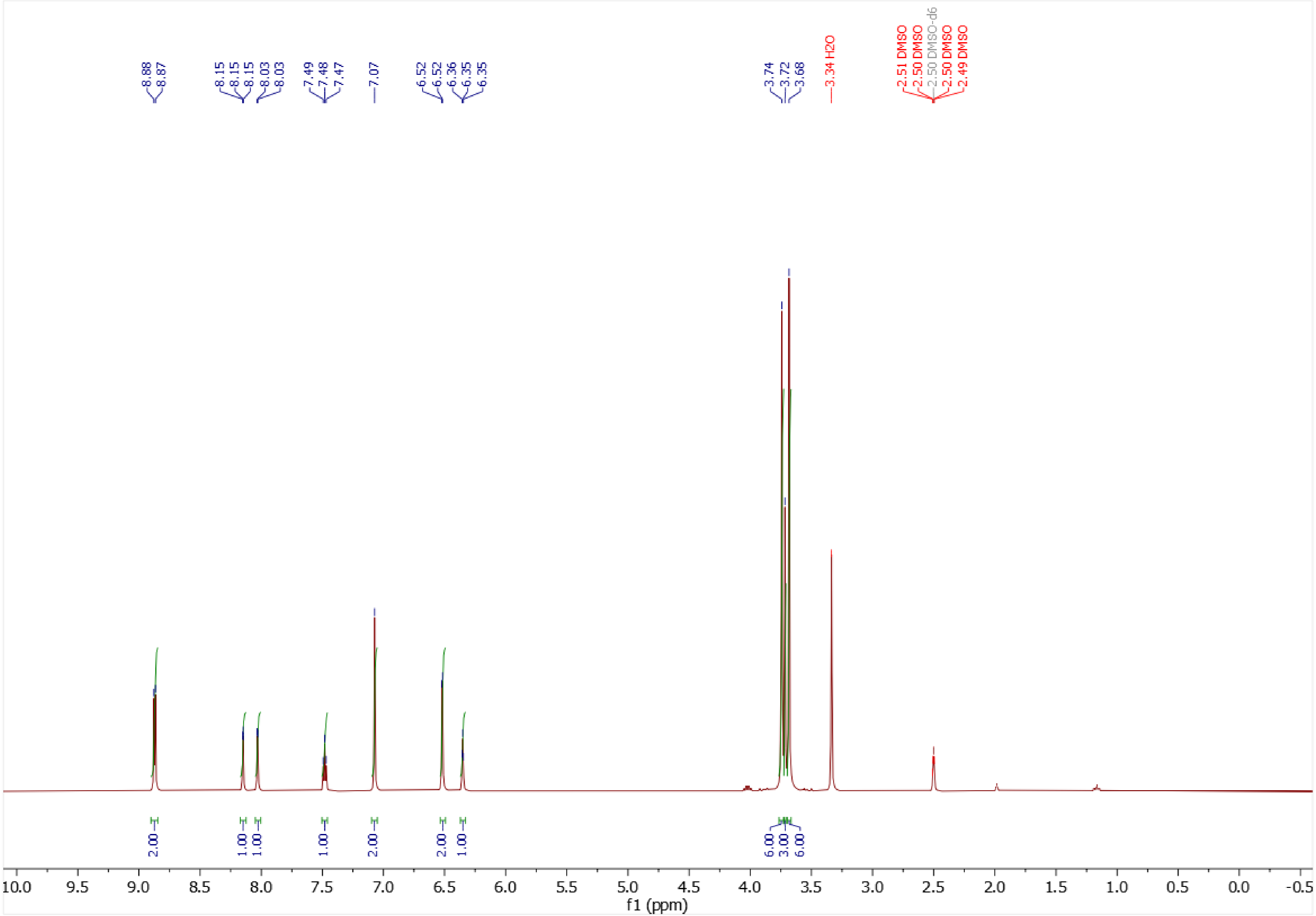
8.88
8.87
8.15
8.15
8.15
8.03
8.03
7.49
7.48
7.47
7.07
6.52
6.52
6.36
6.35
6.35
3.74
3.72
3.68
3.34 H2O
2.51 DMSO
2.50 DMSO
2.50 DMSO-d6
2.50 DMSO
2.49 DMSO
2.00
1.00
1.00
1.00
2.00
2.00
1.00
6.00
3.00
6.00
10.0
9.5
9.0
8.5
8.0
7.5
7.0
6.5
6.0
5.5
5.0
4.5
4.0
3.5
3.0
2.5
2.0
1.5
1.0
0.5
0.0
-0.5
f1 (ppm)

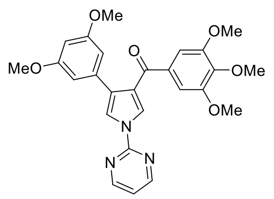

16

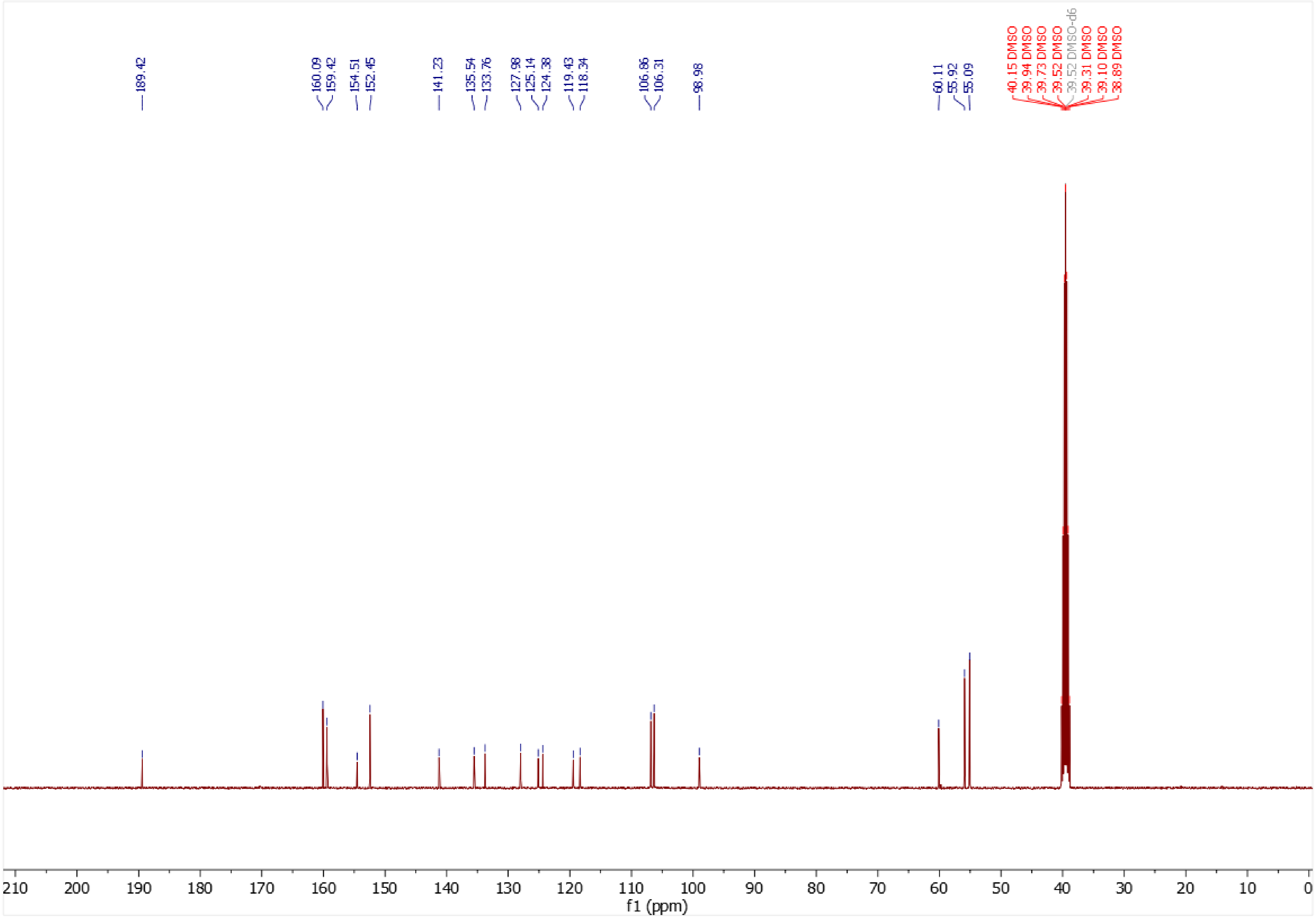

189.42
160.09
159.42
154.51
152.45
141.23
135.54
133.76
127.98
125.14
124.38
119.43
118.34
106.86
106.31
98.98
60.11
55.92
55.09
40.15 DMSO
39.94 DMSO
39.73 DMSO
39.52 DMSO-d6
39.31 DMSO
39.10 DMSO
38.89 DMSO
210 200 190 180 170 160 150 140 130 120 110 100 90 80 70 60 50 40 30 20 10 0
f1 (ppm)

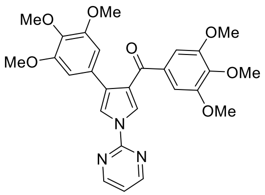
MeO
OMe
MeO
O
OMe
OMe
OMe
N
N
N

17

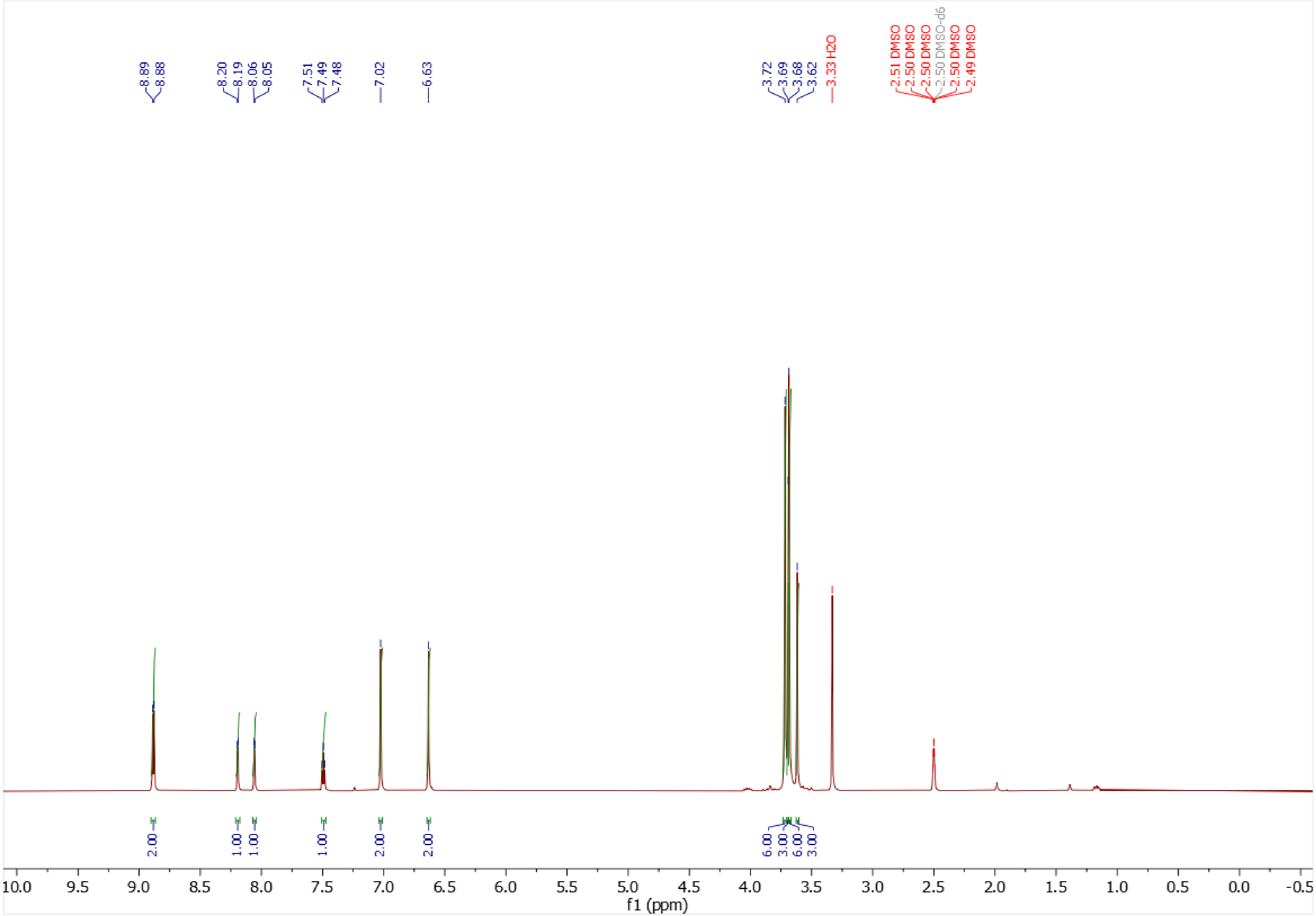
8.89
8.88
8.20
8.19
8.06
8.05
7.51
7.49
7.48
7.02
6.63
3.72
3.69
3.68
3.62
3.33 H2O
2.51 DMSO
2.50 DMSO
2.50 DMSO
2.50 DMSO-d6
2.50 DMSO
2.49 DMSO
2.00
1.00
1.00
1.00
2.00
2.00
6.00
3.00
6.00
3.00
10.0 9.5 9.0 8.5 8.0 7.5 7.0 6.5 6.0 5.5 5.0 4.5 4.0 3.5 3.0 2.5 2.0 1.5 1.0 0.5 0.0 -0.5
f1 (ppm)

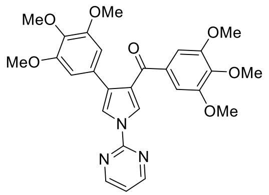
MeO
OMe
MeO
O
OMe
OMe
OMe
N
N
N

17

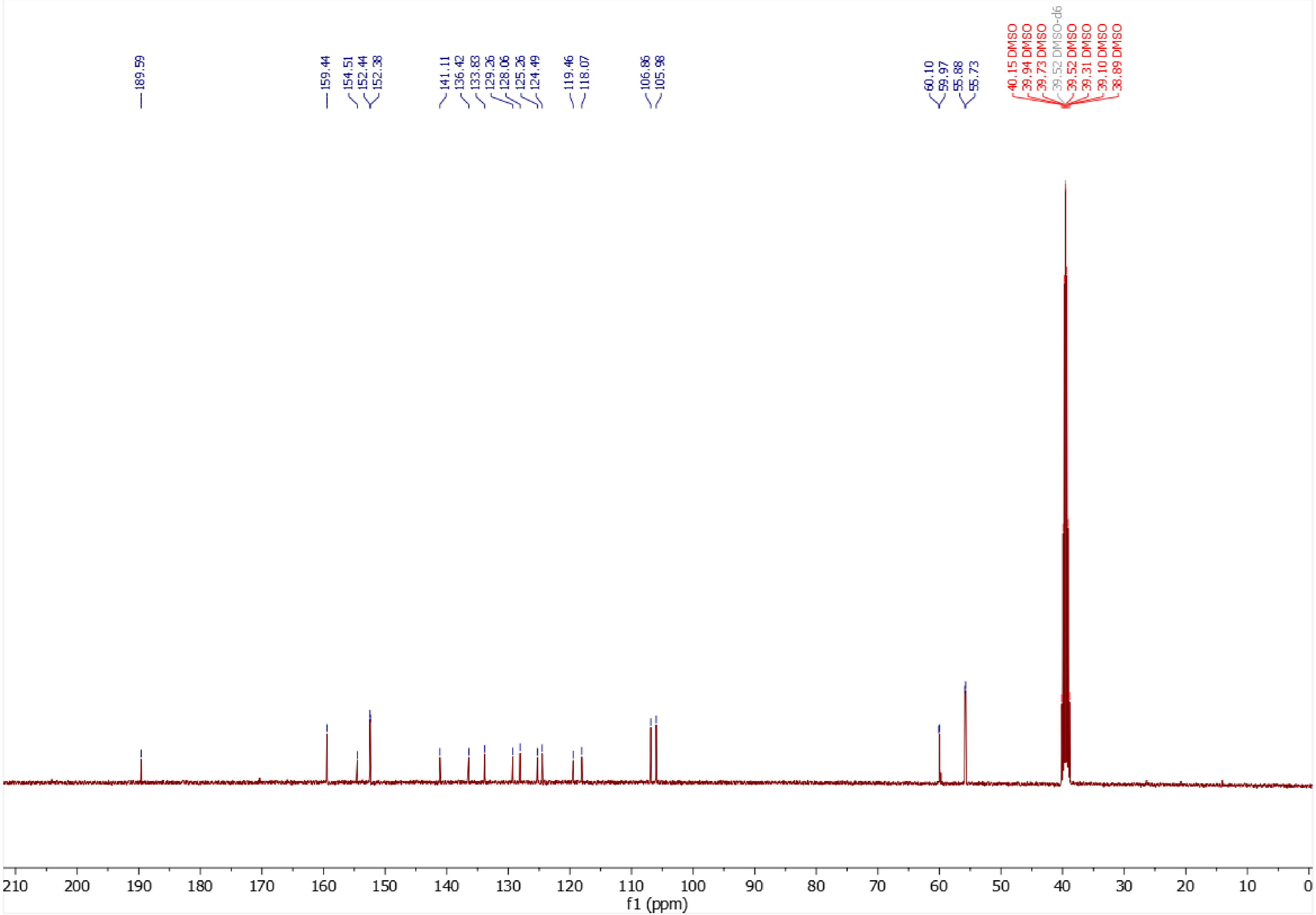
189.59
159.44
154.51
152.44
152.38
141.11
136.42
133.83
129.26
128.06
125.26
124.49
119.46
118.07
106.86
105.98
60.10
59.97
55.88
55.73
40.15 DMSO
39.94 DMSO
39.73 DMSO
39.52 DMSO-d6
39.52 DMSO
39.31 DMSO
39.10 DMSO
38.89 DMSO
210
200
190
180
170
160
150
140
130
120
110
100
90
80
70
60
50
40
30
20
10
0
f1 (ppm)

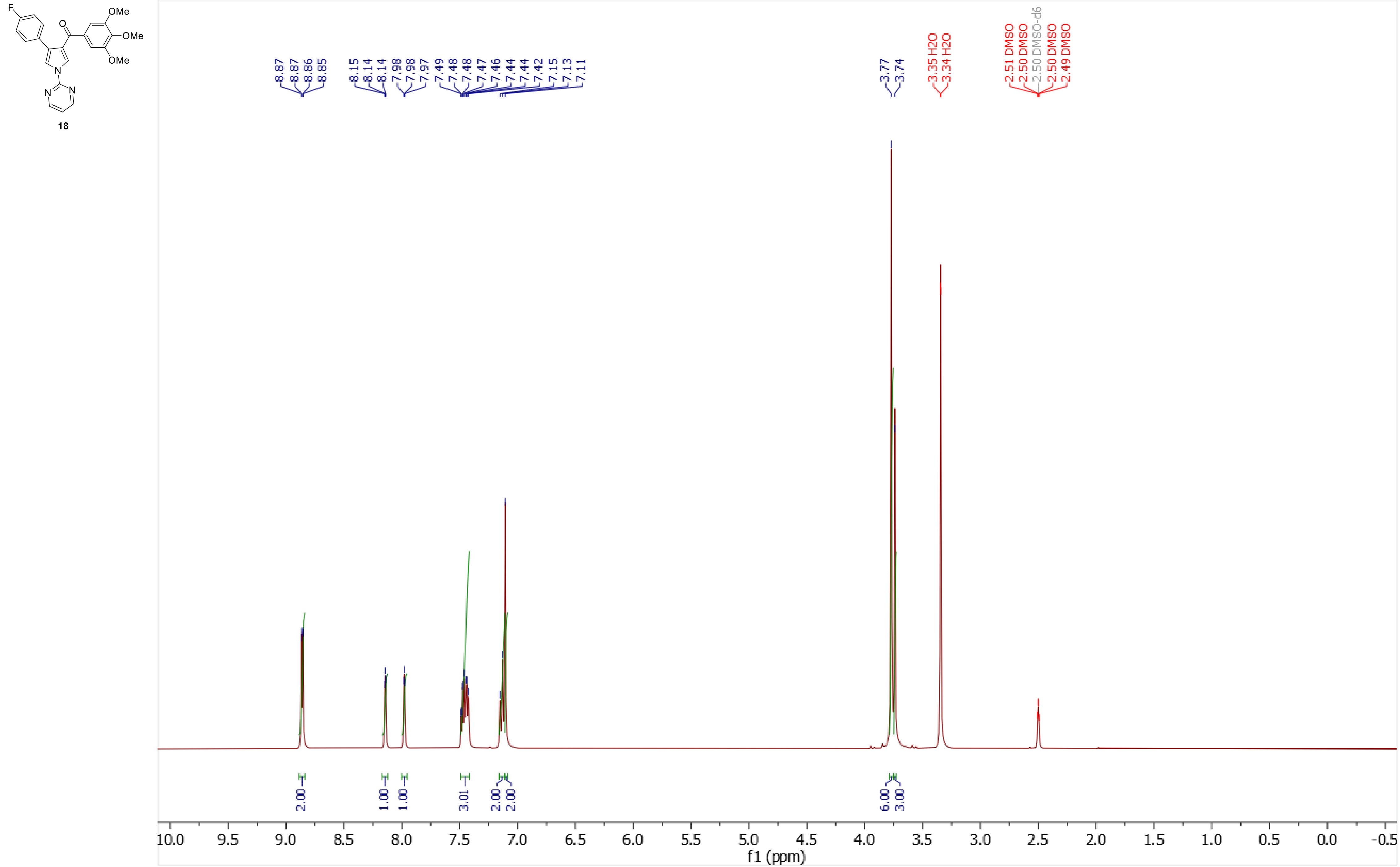
F
O
OMe
OMe
OMe
N
N
N
18
8.87
8.87
8.86
8.85
8.15
8.14
8.14
7.98
7.98
7.97
7.49
7.48
7.48
7.47
7.46
7.44
7.44
7.42
7.15
7.13
7.11
3.77
3.74
3.35 H2O
3.34 H2O
2.51 DMSO
2.50 DMSO
2.50 DMSO-d6
2.50 DMSO
2.49 DMSO
2.00
1.00
1.00
3.01
2.00
2.00
6.00
3.00
f1 (ppm)

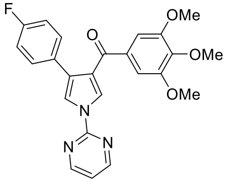
F
O
OMe
OMe
OMe
N
N
N

18

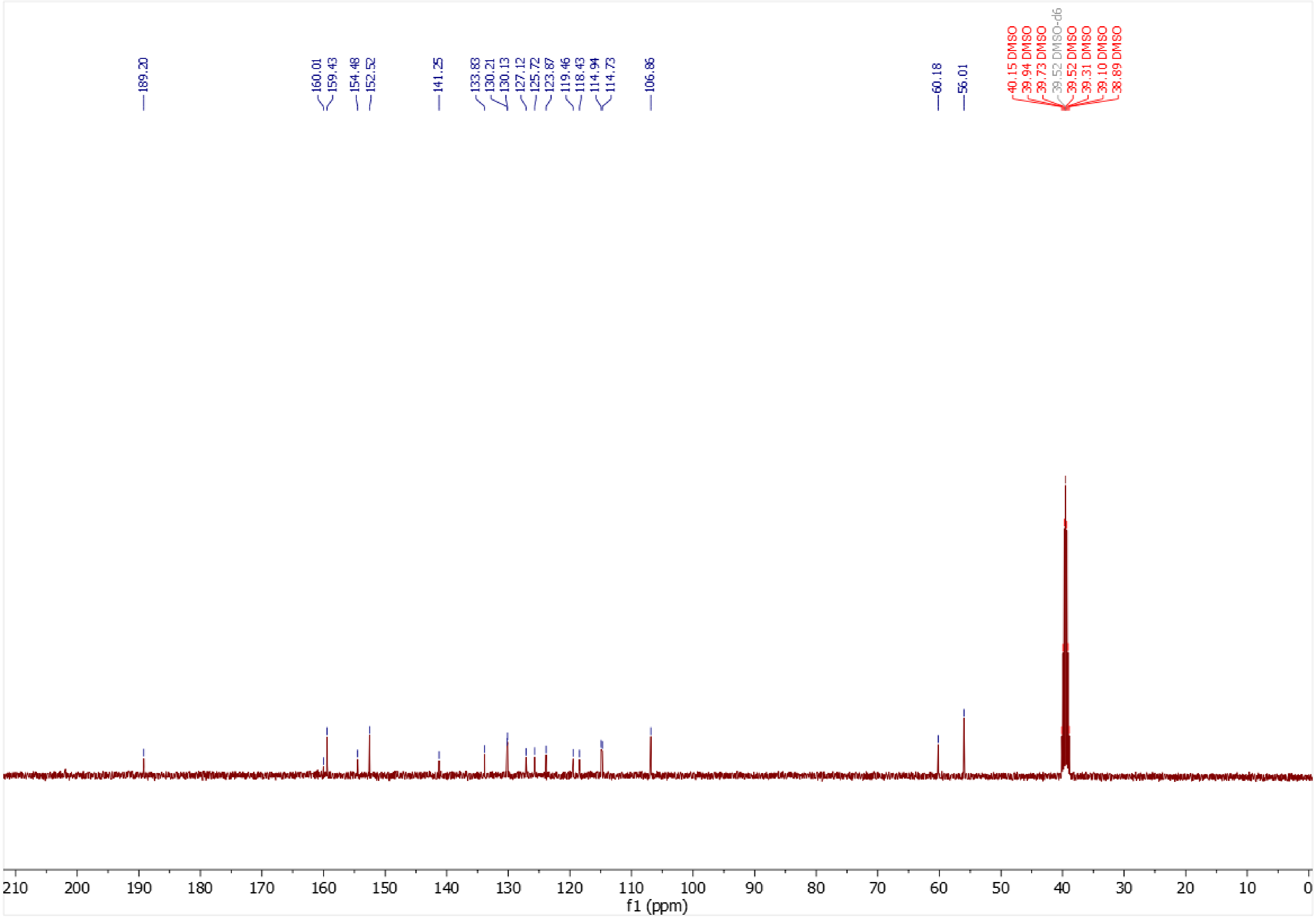
189.20
160.01
159.43
154.48
152.52
141.25
133.83
130.21
130.13
127.12
125.72
123.87
119.46
118.43
114.94
114.73
106.86
60.18
56.01
40.15 DMSO
39.94 DMSO
39.73 DMSO
39.52 DMSO-d6
39.52 DMSO
39.31 DMSO
39.10 DMSO
38.89 DMSO
210 200 190 180 170 160 150 140 130 120 110 100 90 80 70 60 50 40 30 20 10 0
f1 (ppm)

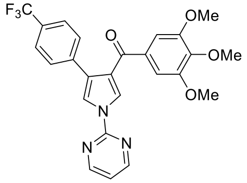

19

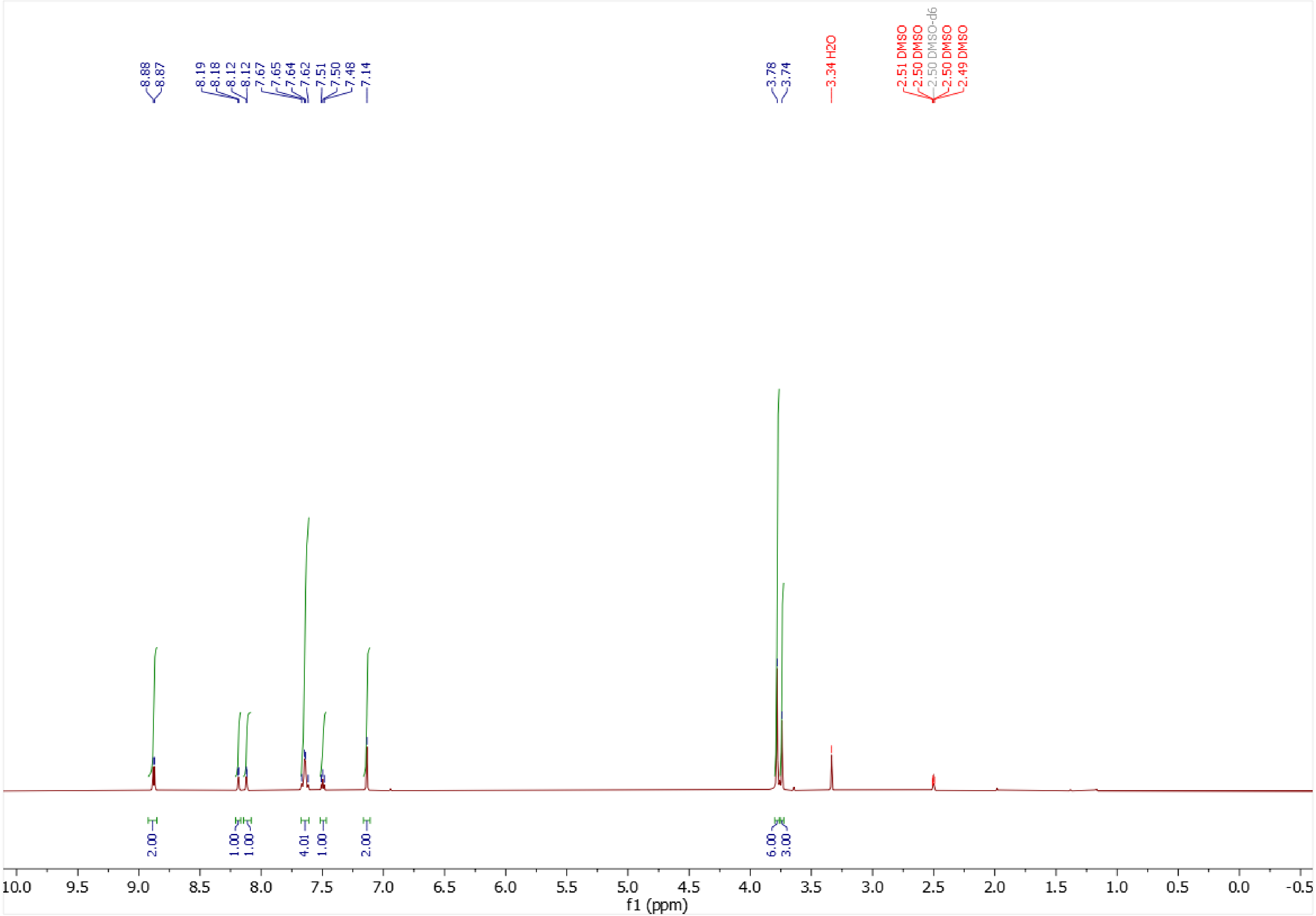

8.88
8.87
8.19
8.18
8.12
8.12
7.67
7.65
7.64
7.62
7.51
7.50
7.48
7.14
3.78
3.74
3.34 H2O
2.51 DMSO
2.50 DMSO
2.50 DMSO-d6
2.50 DMSO
2.49 DMSO
2.00
1.00
1.00
4.01
1.00
2.00
6.00
3.00
10.0 9.5 9.0 8.5 8.0 7.5 7.0 6.5 6.0 5.5 5.0 4.5 4.0 3.5 3.0 2.5 2.0 1.5 1.0 0.5 0.0 -0.5
f1 (ppm)

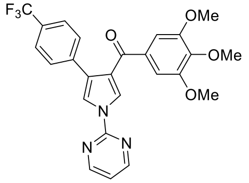
F3C
O
OMe
OMe
OMe
N
N
N

19

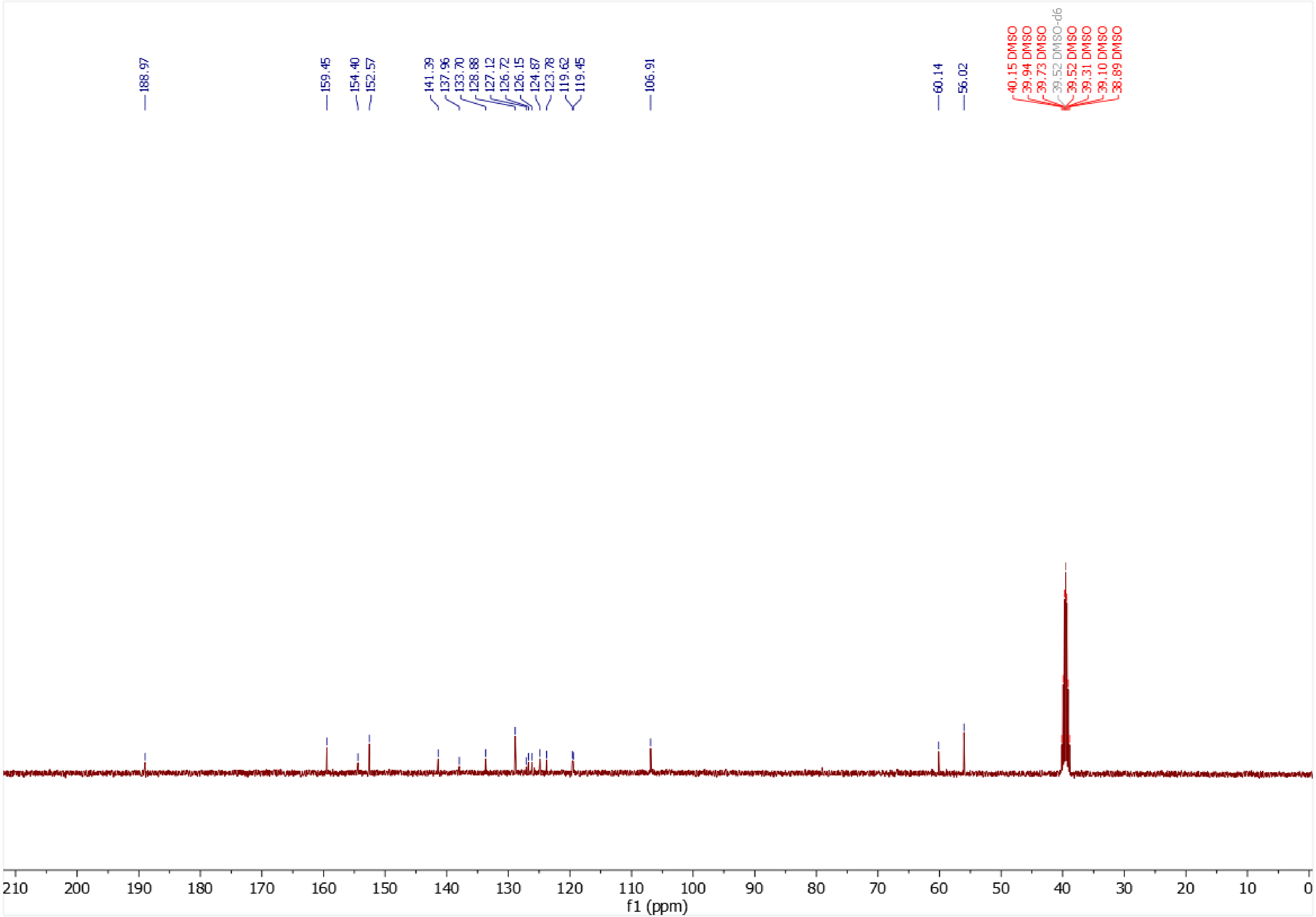
188.97
159.45
154.40
152.57
141.39
137.96
133.70
128.88
127.12
126.72
126.15
124.87
123.78
119.62
119.45
106.91
60.14
56.02
40.15 DMSO
39.94 DMSO
39.73 DMSO
39.52 DMSO-d6
39.52 DMSO
39.31 DMSO
39.10 DMSO
38.89 DMSO
210 200 190 180 170 160 150 140 130 120 110 100 90 80 70 60 50 40 30 20 10 0
f1 (ppm)

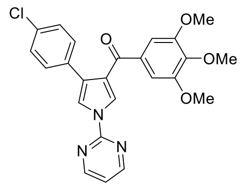

20

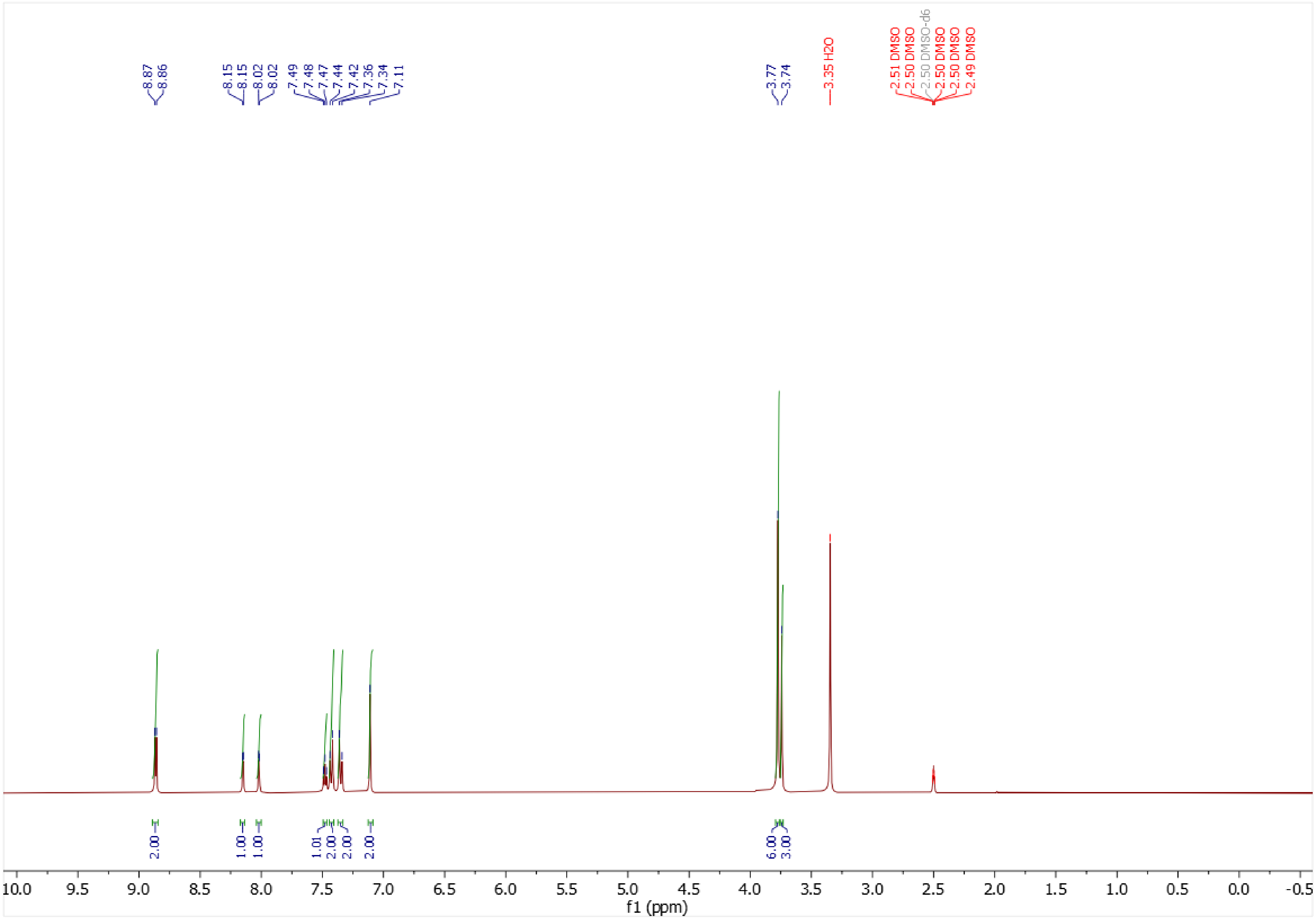

8.87
8.86
8.15
8.15
8.02
8.02
7.49
7.48
7.47
7.44
7.42
7.36
7.34
7.11
3.77
3.74
3.35 H2O
2.51 DMSO
2.50 DMSO
2.50 DMSO-d6
2.50 DMSO
2.50 DMSO
2.49 DMSO
2.00
1.00
1.00
1.01
2.00
2.00
2.00
6.00
3.00
10.0 9.5 9.0 8.5 8.0 7.5 7.0 6.5 6.0 5.5 5.0 4.5 4.0 3.5 3.0 2.5 2.0 1.5 1.0 0.5 0.0 -0.5
f1 (ppm)

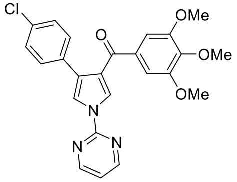
Cl
O
OMe
OMe
OMe
N
N
N

20

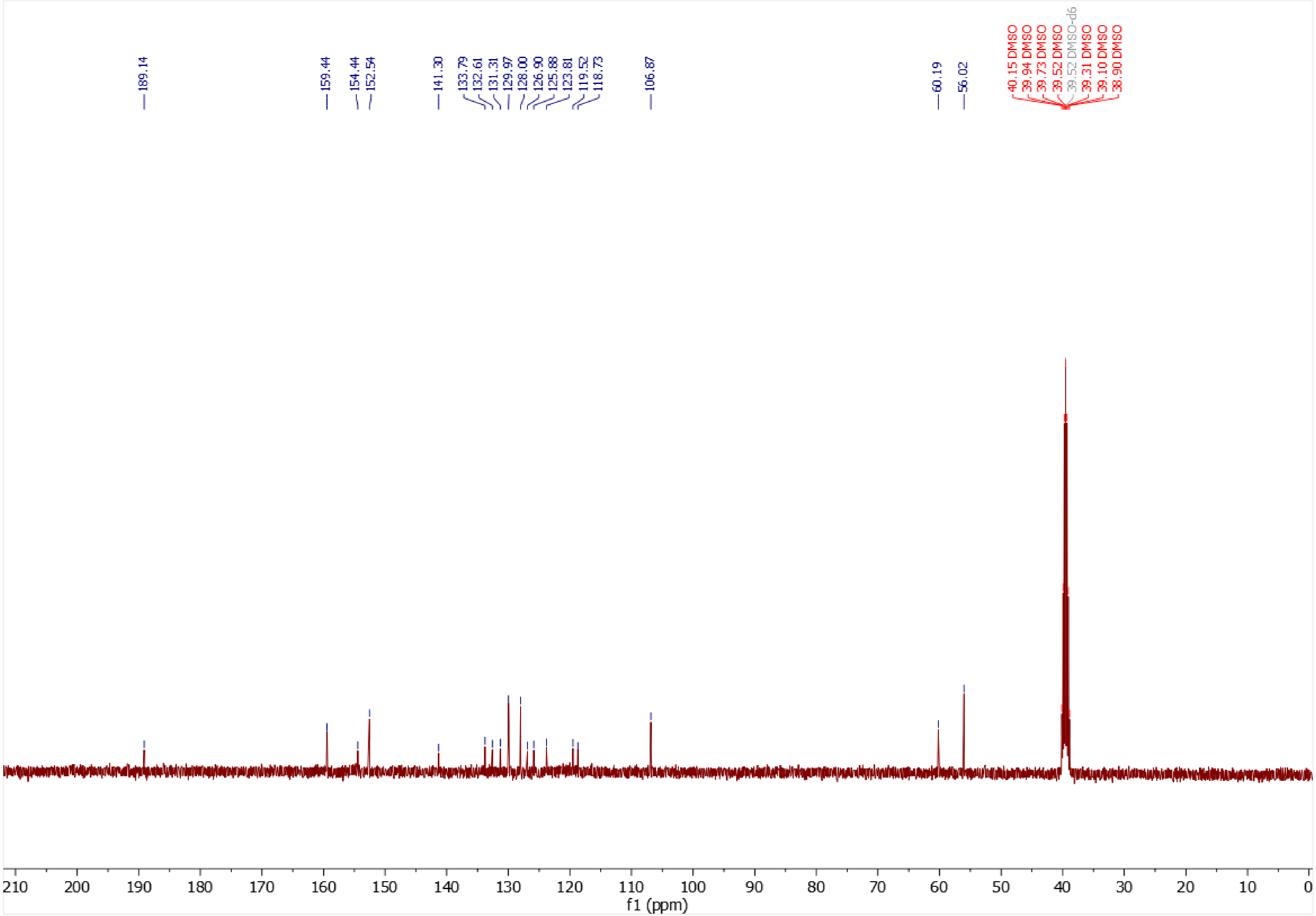
189.14
159.44
154.44
152.54
141.30
133.79
132.61
131.31
129.97
128.00
126.90
125.88
123.81
119.52
118.73
106.87
60.19
56.02
40.15 DMSO
39.94 DMSO
39.73 DMSO
39.52 DMSO
39.52 DMSO-d6
39.31 DMSO
39.10 DMSO
38.90 DMSO
210
200
190
180
170
160
150
140
130
120
110
100
90
80
70
60
50
40
30
20
10
0
f1 (ppm)

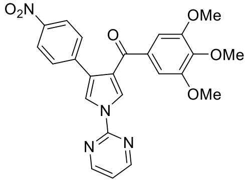

21

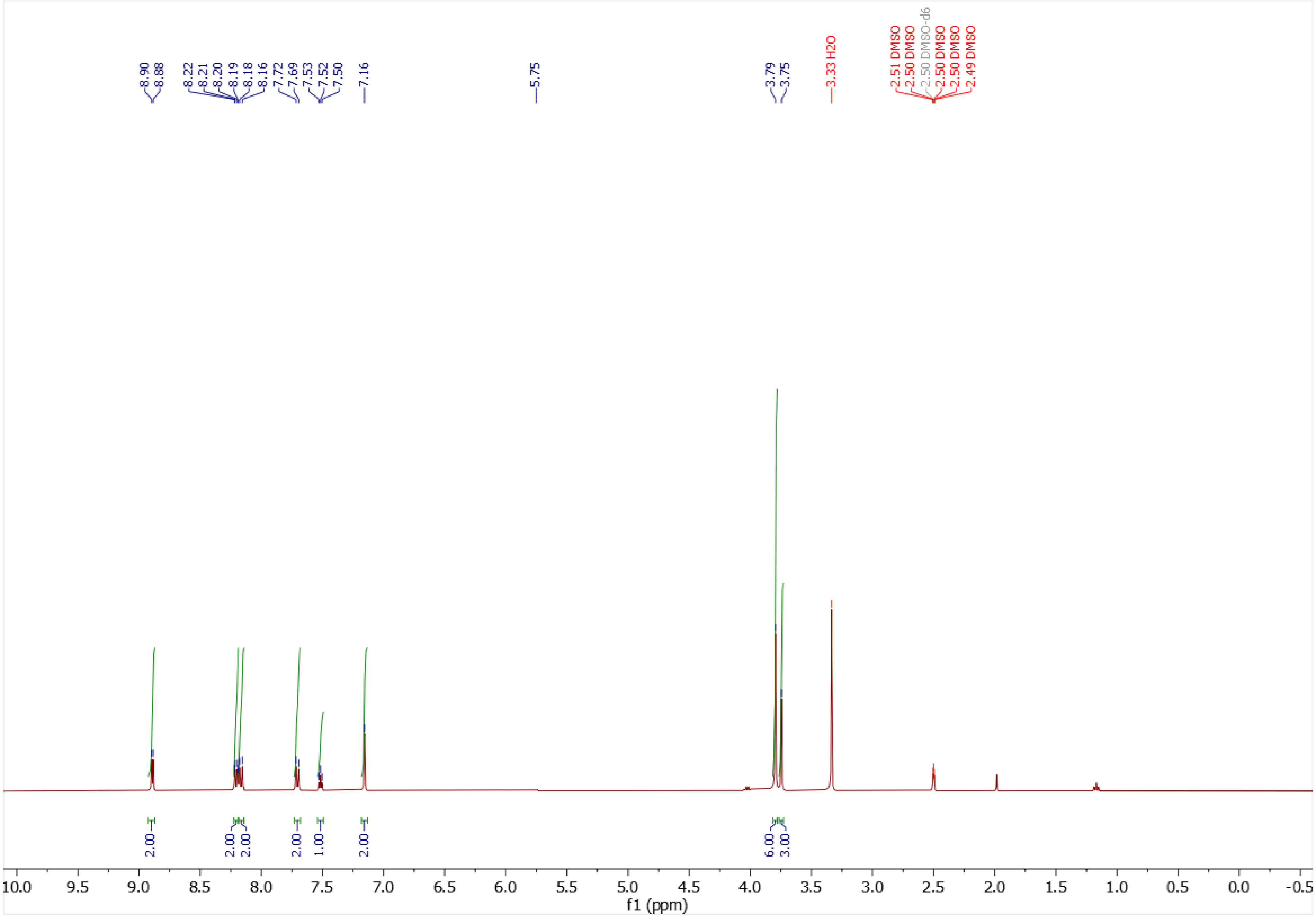
8.90
8.88
8.22
8.21
8.20
8.19
8.18
8.16
7.72
7.69
7.53
7.52
7.50
7.16
5.75
3.79
3.75
3.33 H2O
2.51 DMSO
2.50 DMSO
2.50 DMSO-d6
2.50 DMSO
2.50 DMSO
2.49 DMSO
2.00
2.00
2.00
2.00
1.00
2.00
6.00
3.00
10.0 9.5 9.0 8.5 8.0 7.5 7.0 6.5 6.0 5.5 5.0 4.5 4.0 3.5 3.0 2.5 2.0 1.5 1.0 0.5 0.0 -0.5
f1 (ppm)

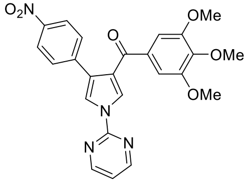

O2N
OMe
OMe
OMe
O
N
N
N

21

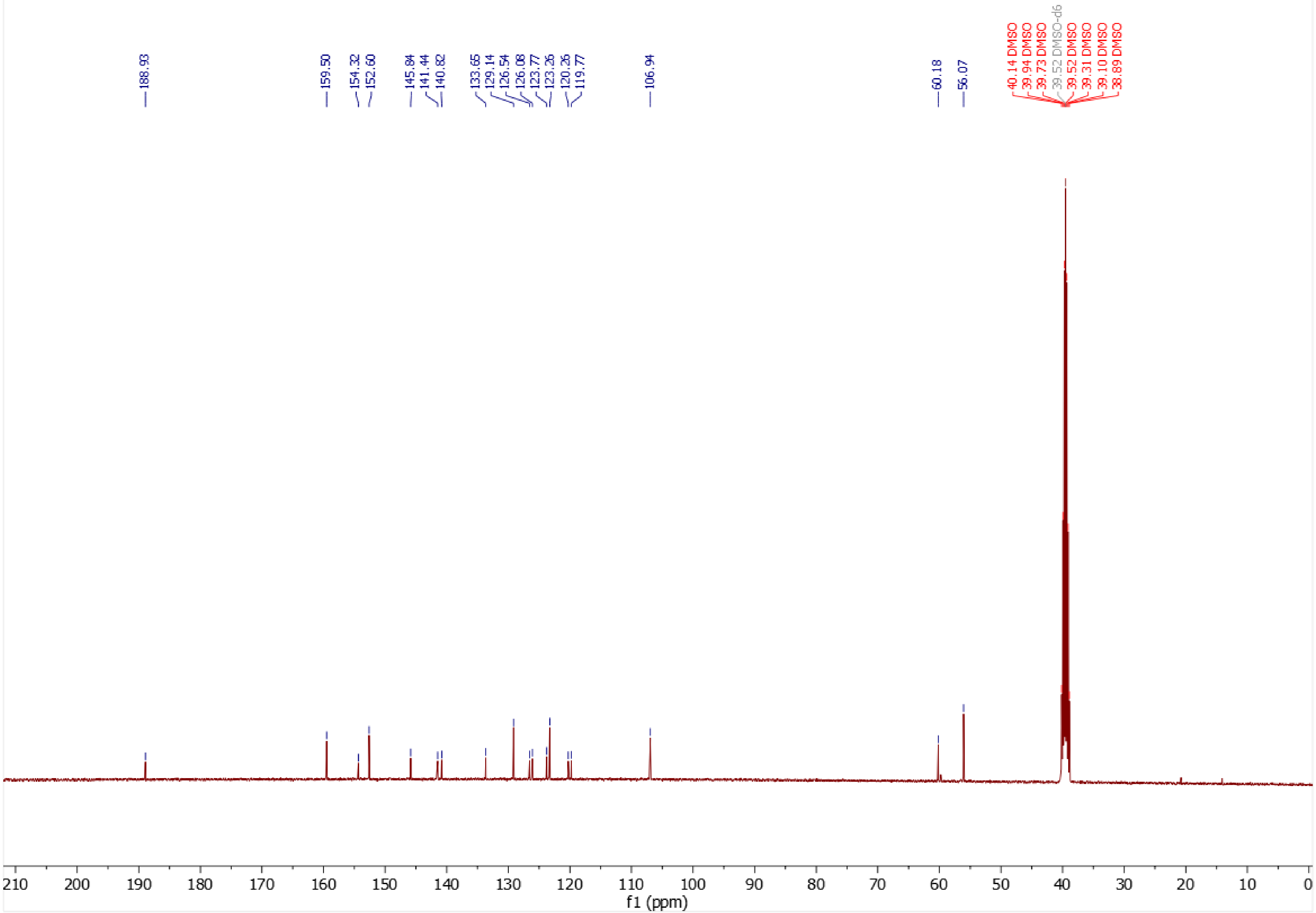

188.93
159.50
154.32
152.60
145.84
141.44
140.82
133.65
129.14
126.54
126.08
123.77
123.26
120.26
119.77
106.94
60.18
56.07
40.14 DMSO
39.94 DMSO
39.73 DMSO
39.52 DMSO-d6
39.52 DMSO
39.31 DMSO
39.10 DMSO
38.89 DMSO
210 200 190 180 170 160 150 140 130 120 110 100 90 80 70 60 50 40 30 20 10 0
f1 (ppm)

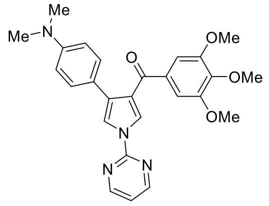

Me
Me
N
O
OMe
OMe
OMe
N
N
N

22

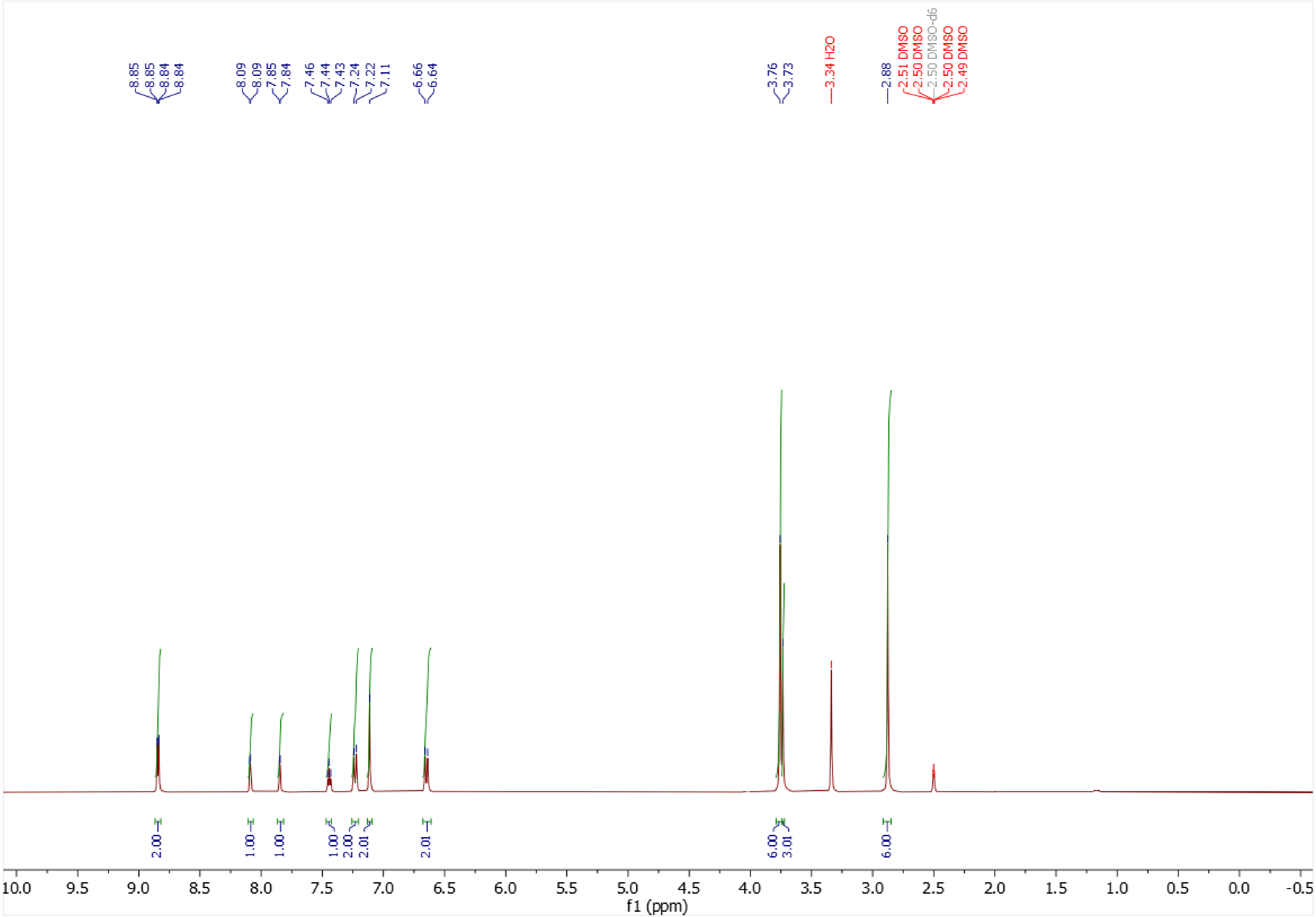

8.85
8.85
8.84
8.84
8.09
8.09
7.85
7.84
7.46
7.44
7.43
7.24
7.22
7.11
6.66
6.64
3.76
3.73
3.34 H2O
2.88
2.51 DMSO
2.50 DMSO
2.50 DMSO-d6
2.50 DMSO
2.49 DMSO
2.00
1.00
1.00
1.00
2.00
2.01
2.01
6.00
3.01
6.00
10.0
9.5
9.0
8.5
8.0
7.5
7.0
6.5
6.0
5.5
5.0
4.5
4.0
3.5
3.0
2.5
2.0
1.5
1.0
0.5
0.0
-0.5
f1 (ppm)

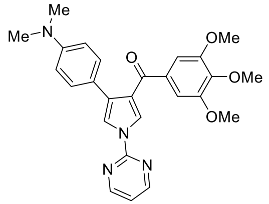

22

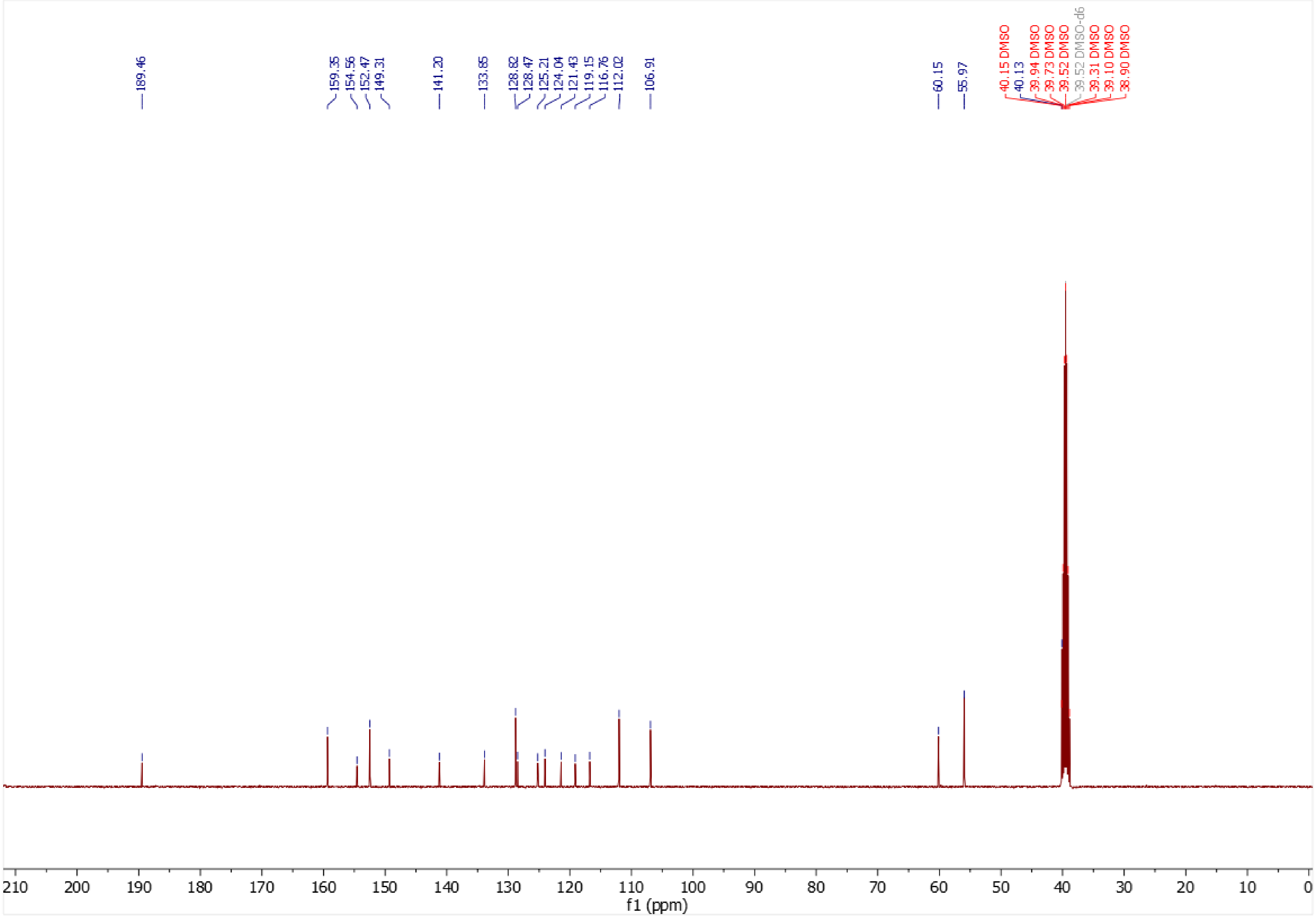

189.46
159.35
154.56
152.47
149.31
141.20
133.85
128.82
128.47
125.21
124.04
121.43
119.15
116.76
112.02
106.91
60.15
55.97
40.15 DMSO
40.13
39.94 DMSO
39.73 DMSO
39.52 DMSO
39.52 DMSO-d6
39.31 DMSO
39.10 DMSO
38.90 DMSO
210 200 190 180 170 160 150 140 130 120 110 100 90 80 70 60 50 40 30 20 10 0
f1 (ppm)

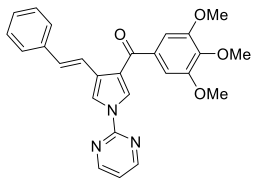
OMe
O
OMe
OMe
N
N
N

23

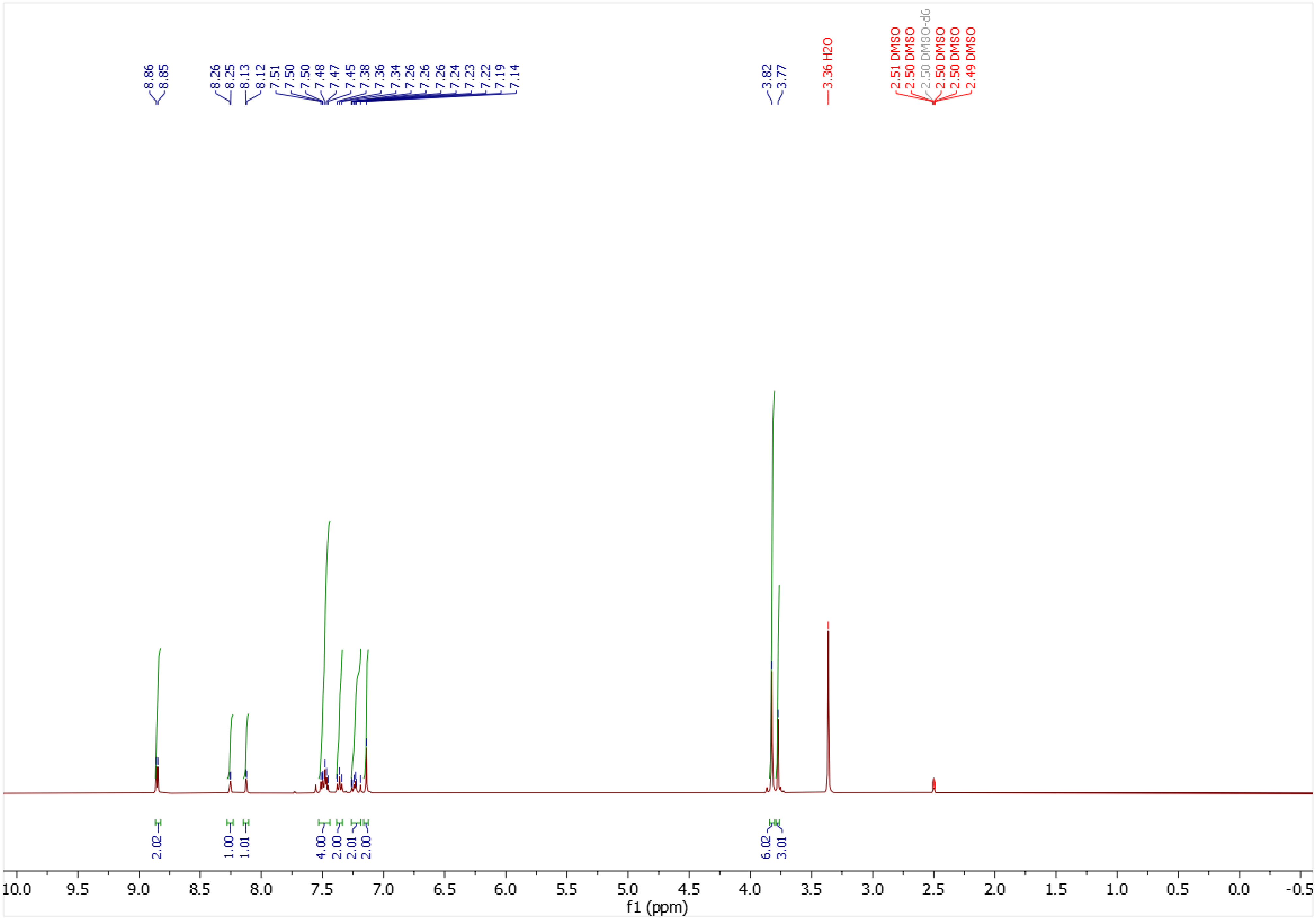
8.86
8.85
8.26
8.25
8.13
8.12
7.51
7.50
7.50
7.48
7.47
7.45
7.38
7.36
7.34
7.26
7.26
7.26
7.24
7.23
7.22
7.19
7.14
3.82
3.77
3.36 H2O
2.51 DMSO
2.50 DMSO
2.50 DMSO-d6
2.50 DMSO
2.50 DMSO
2.49 DMSO
2.02
1.00
1.01
4.00
2.00
2.01
2.00
6.02
3.01
10.0
9.5
9.0
8.5
8.0
7.5
7.0
6.5
6.0
5.5
5.0
4.5
4.0
3.5
3.0
2.5
2.0
1.5
1.0
0.5
0.0
-0.5
f1 (ppm)

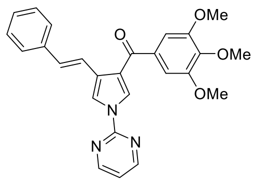

23

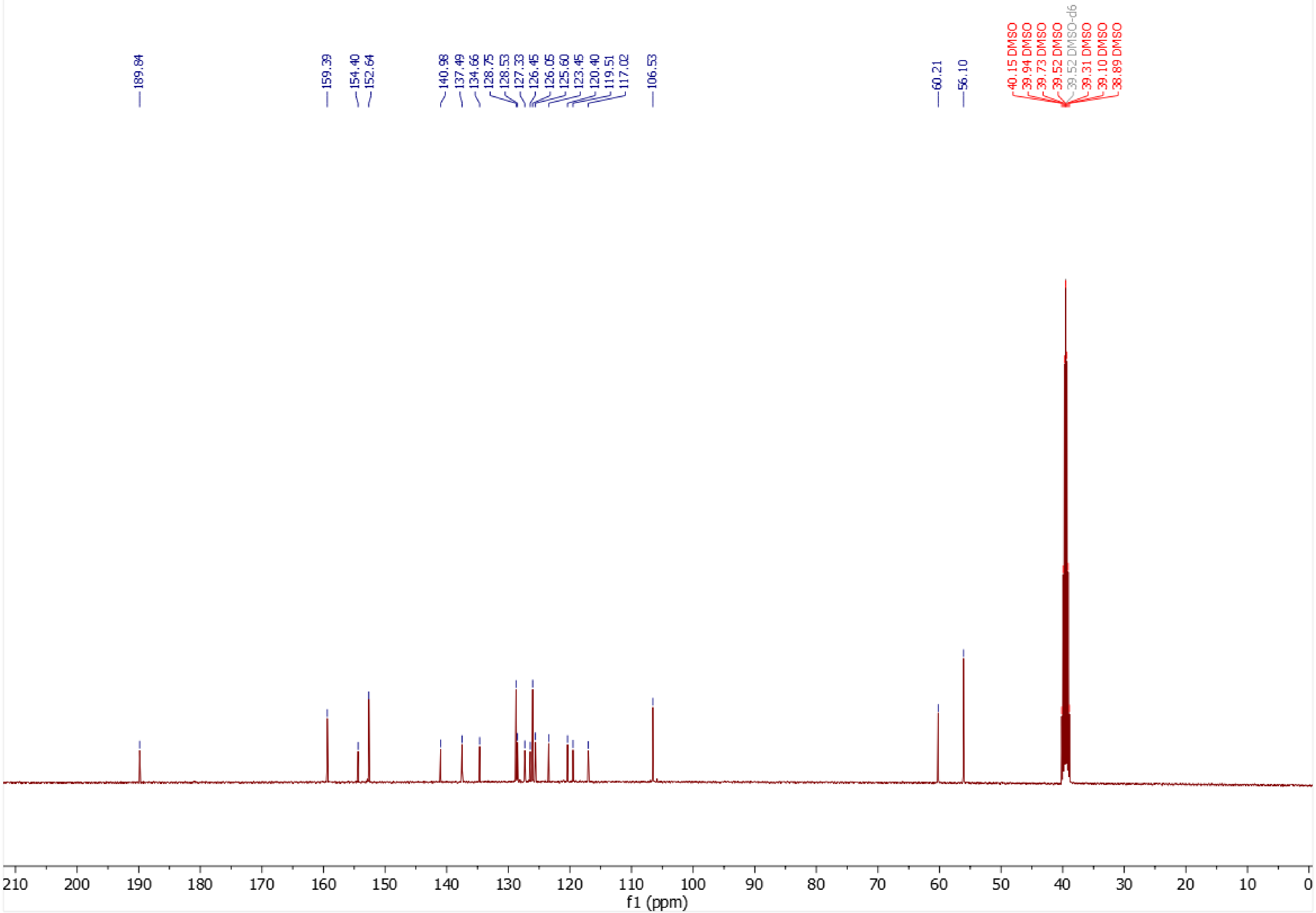

189.84
159.39
154.40
152.64
140.98
137.49
134.66
128.75
128.53
127.33
126.45
126.05
125.60
123.45
120.40
119.51
117.02
106.53
60.21
56.10
40.15 DMSO
39.94 DMSO
39.73 DMSO
39.52 DMSO
39.52 DMSO-d6
39.31 DMSO
39.10 DMSO
38.89 DMSO
210 200 190 180 170 160 150 140 130 120 110 100 90 80 70 60 50 40 30 20 10 0
f1 (ppm)

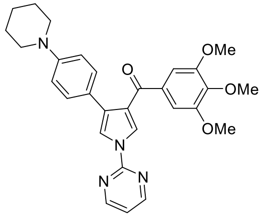
OMe
O
OMe
OMe
N
N
N
N

24

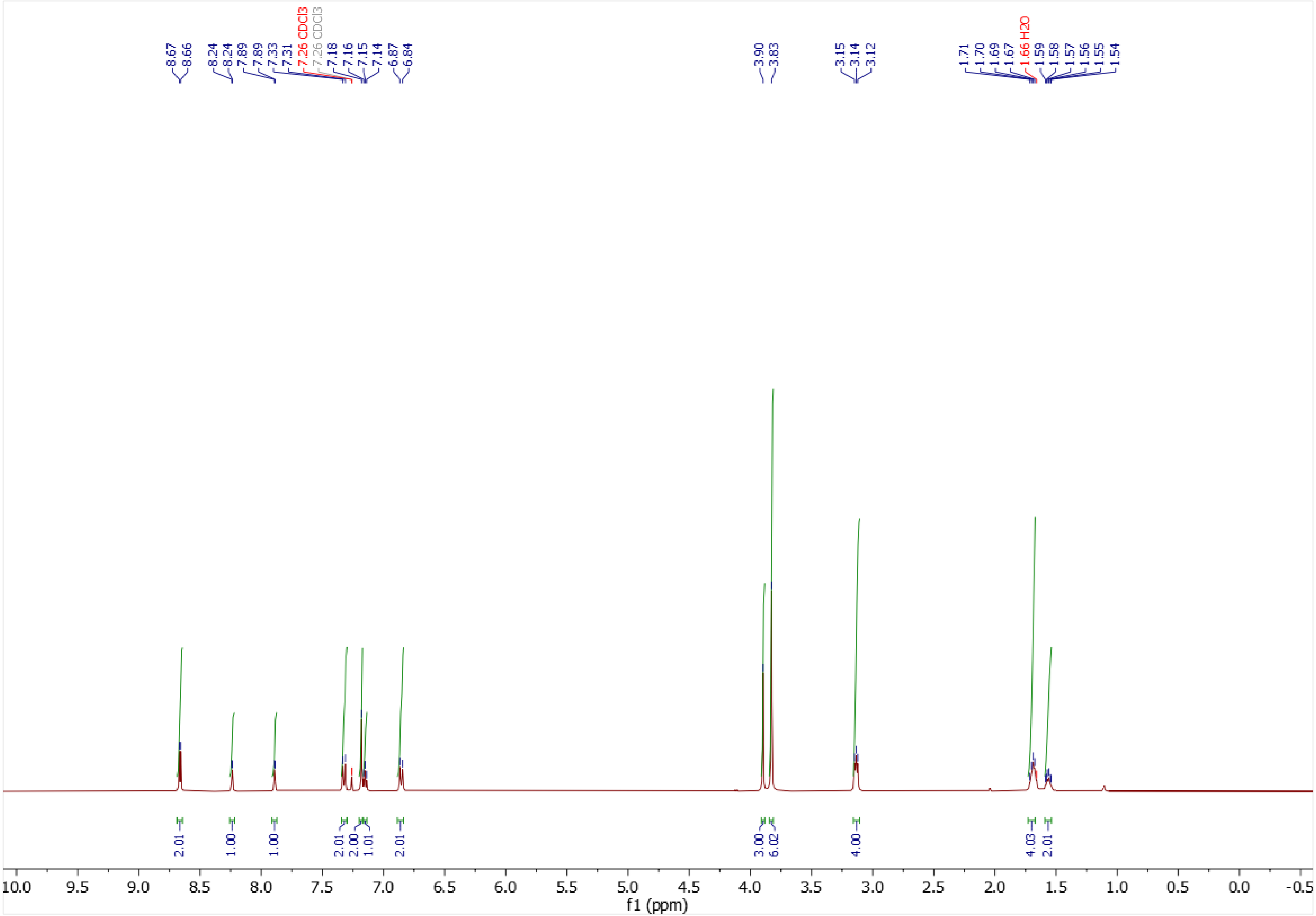
8.67
8.66
8.24
8.24
7.89
7.89
7.33
7.31
7.26 CDCl3
7.26 CDCl3
7.18
7.16
7.15
7.14
6.87
6.84
3.90
3.83
3.15
3.14
3.12
1.71
1.70
1.69
1.67
1.66 H2O
1.59
1.58
1.57
1.56
1.55
1.54
2.01
1.00
1.00
2.01
2.00
1.01
2.01
3.00
6.02
4.00
4.03
2.01
10.0
9.5
9.0
8.5
8.0
7.5
7.0
6.5
6.0
5.5
5.0
4.5
4.0
3.5
3.0
2.5
2.0
1.5
1.0
0.5
0.0
-0.5
f1 (ppm)

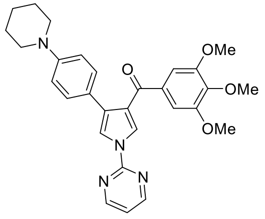

24

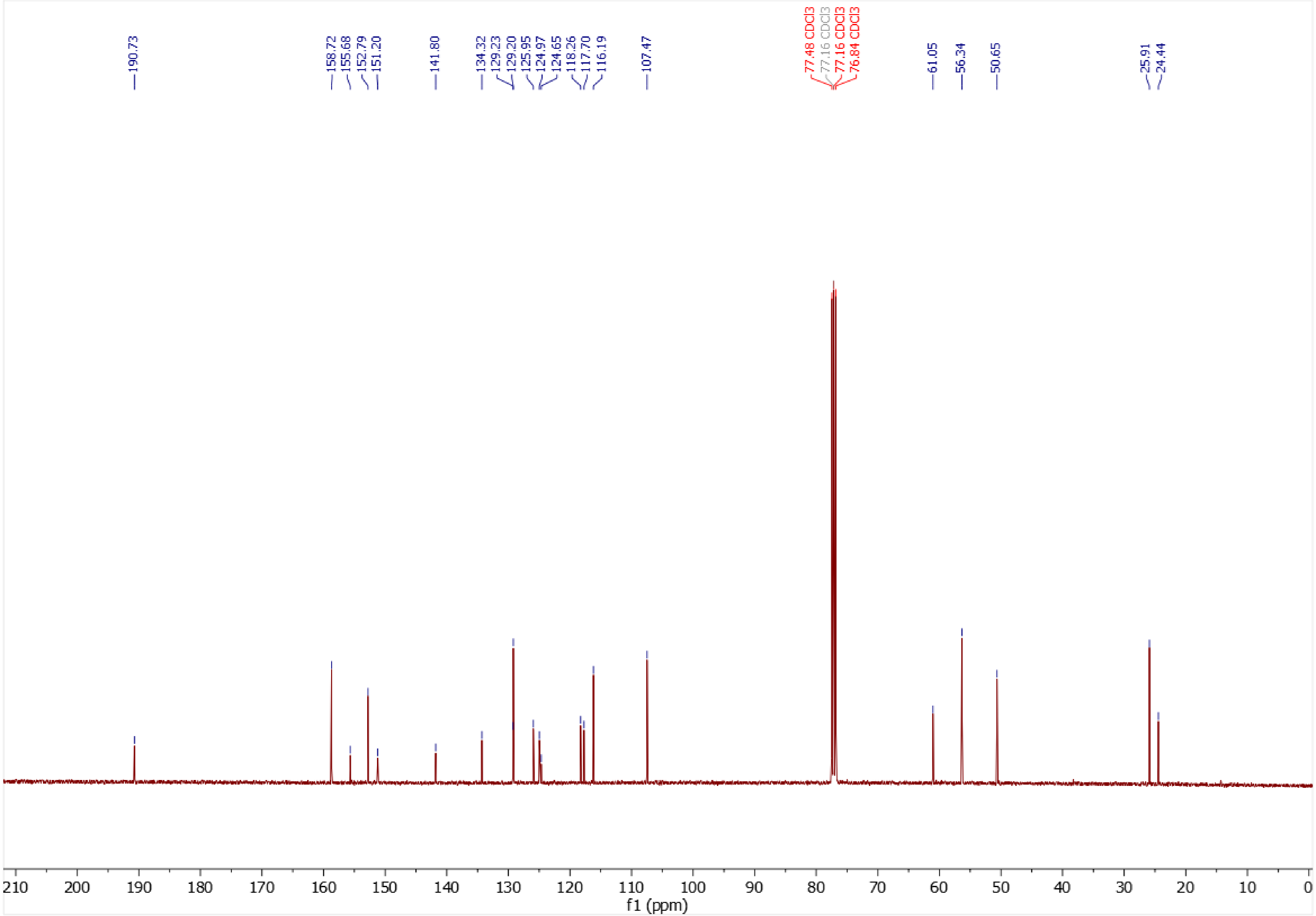

190.73
158.72
155.68
152.79
151.20
141.80
134.32
129.23
129.20
125.95
124.97
124.65
118.26
117.70
116.19
107.47
77.48 CDCl3
77.16 CDCl3
77.16 CDCl3
76.84 CDCl3
61.05
56.34
50.65
25.91
24.44
210
200
190
180
170
160
150
140
130
120
110
100
90
80
70
60
50
40
30
20
10
0
f1 (ppm)

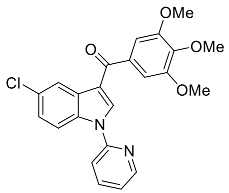
OMe
O
OMe
Cl
OMe
N
N

25

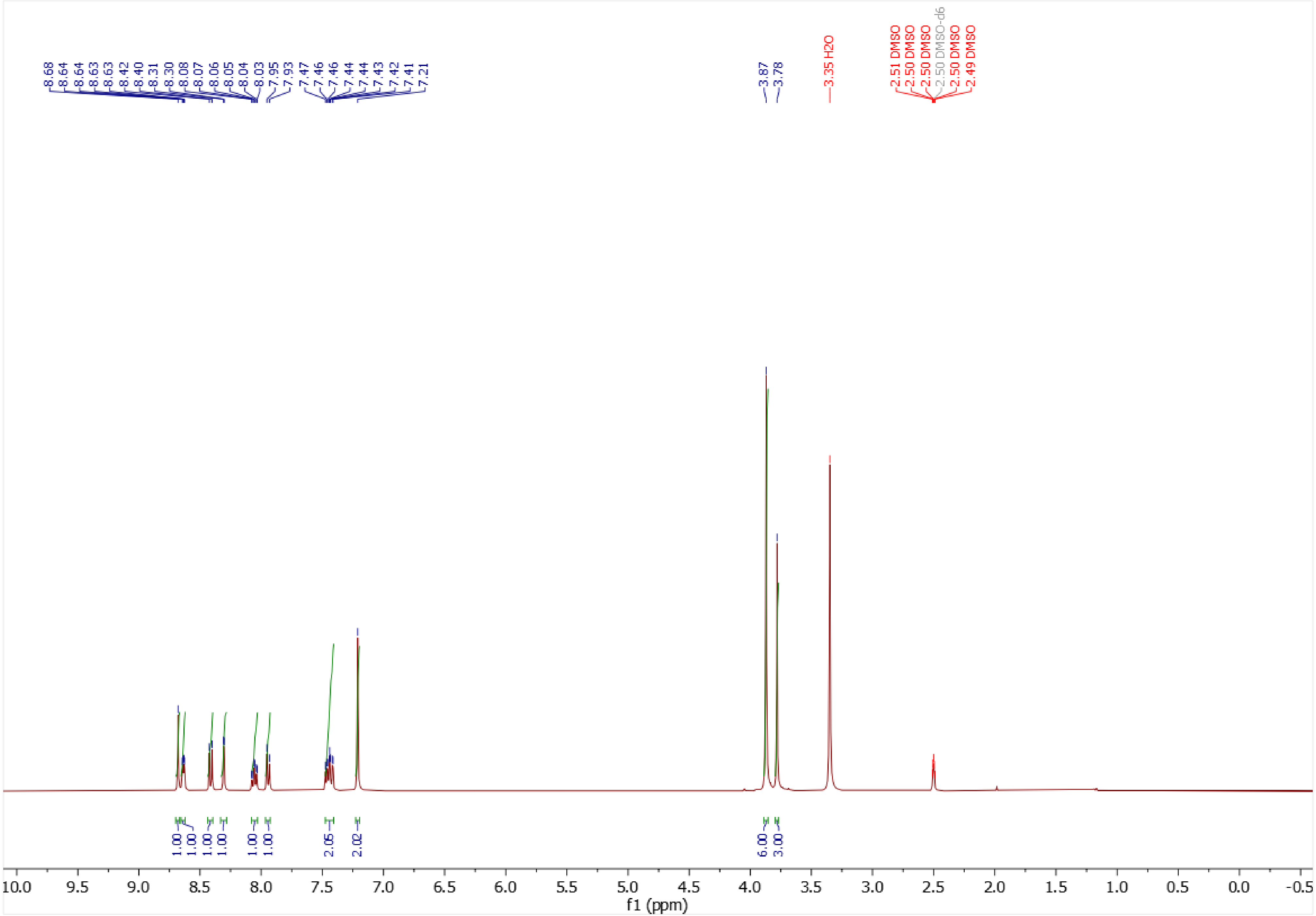
8.68 8.64 8.64 8.63 8.63 8.42 8.40 8.31 8.30 8.08 8.07 8.06 8.05 8.04 8.03 7.95 7.93 7.47 7.46 7.46 7.44 7.44 7.43 7.42 7.41 7.21
3.87 3.78
3.35 H2O
2.51 DMSO 2.50 DMSO 2.50 DMSO 2.50 DMSO-d6 2.50 DMSO 2.49 DMSO
1.00 1.00 1.00 1.00 1.00 1.00 2.05 2.02 6.00 3.00
10.0 9.5 9.0 8.5 8.0 7.5 7.0 6.5 6.0 5.5 5.0 4.5 4.0 3.5 3.0 2.5 2.0 1.5 1.0 0.5 0.0 -0.5
f1 (ppm)

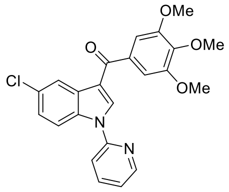

OMe
O
OMe
Cl
OMe
N
N

25

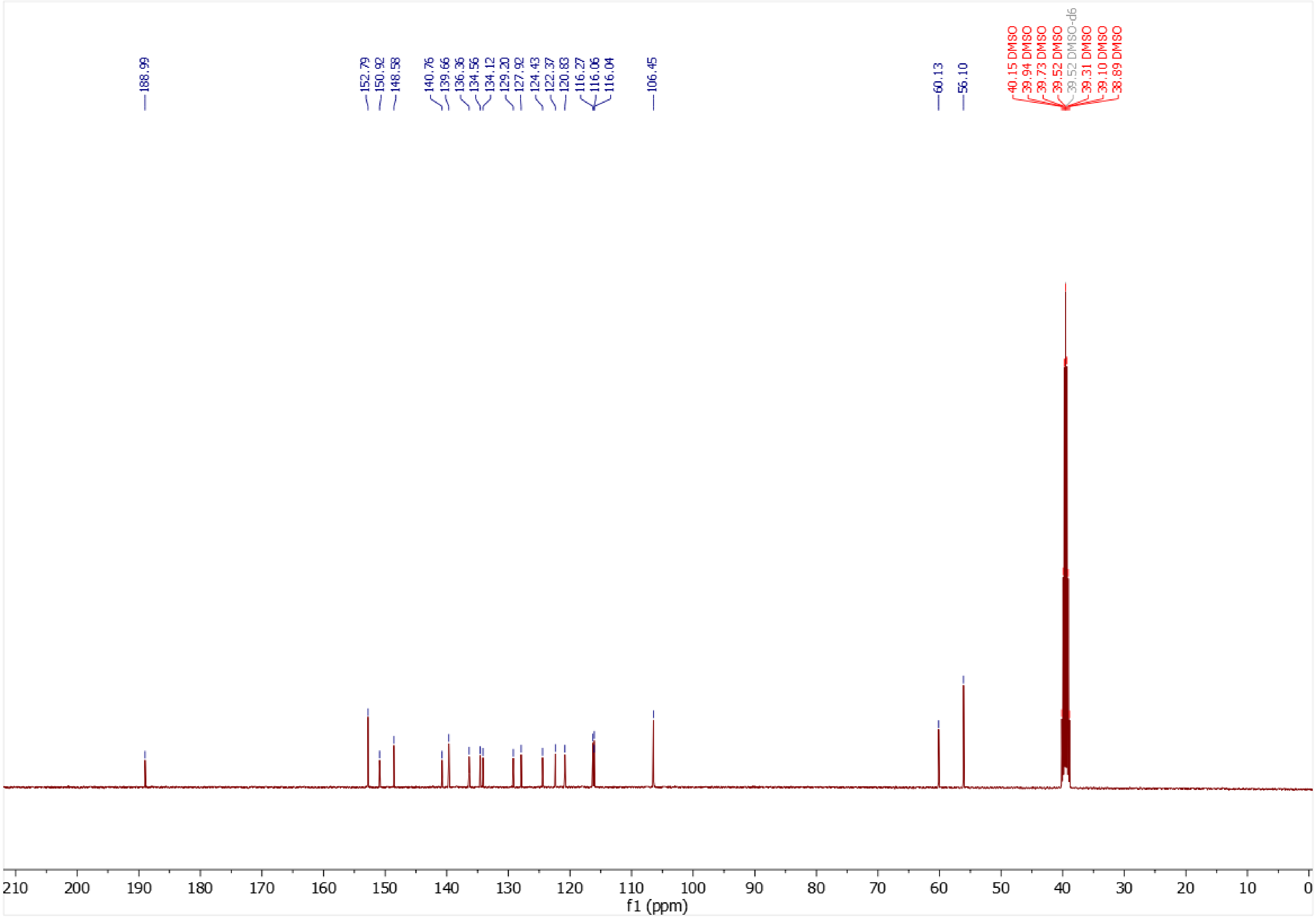

188.99
152.79
150.92
148.58
140.76
139.66
136.36
134.56
134.12
129.20
127.92
124.43
122.37
120.83
116.27
116.06
116.04
106.45
60.13
56.10
40.15 DMSO
39.94 DMSO
39.73 DMSO
39.52 DMSO
39.52 DMSO-d6
39.31 DMSO
39.10 DMSO
38.89 DMSO
210 200 190 180 170 160 150 140 130 120 110 100 90 80 70 60 50 40 30 20 10 0
f1 (ppm)

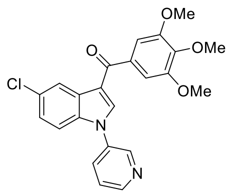
OMe
OMe
OMe
O
Cl
N
N

26

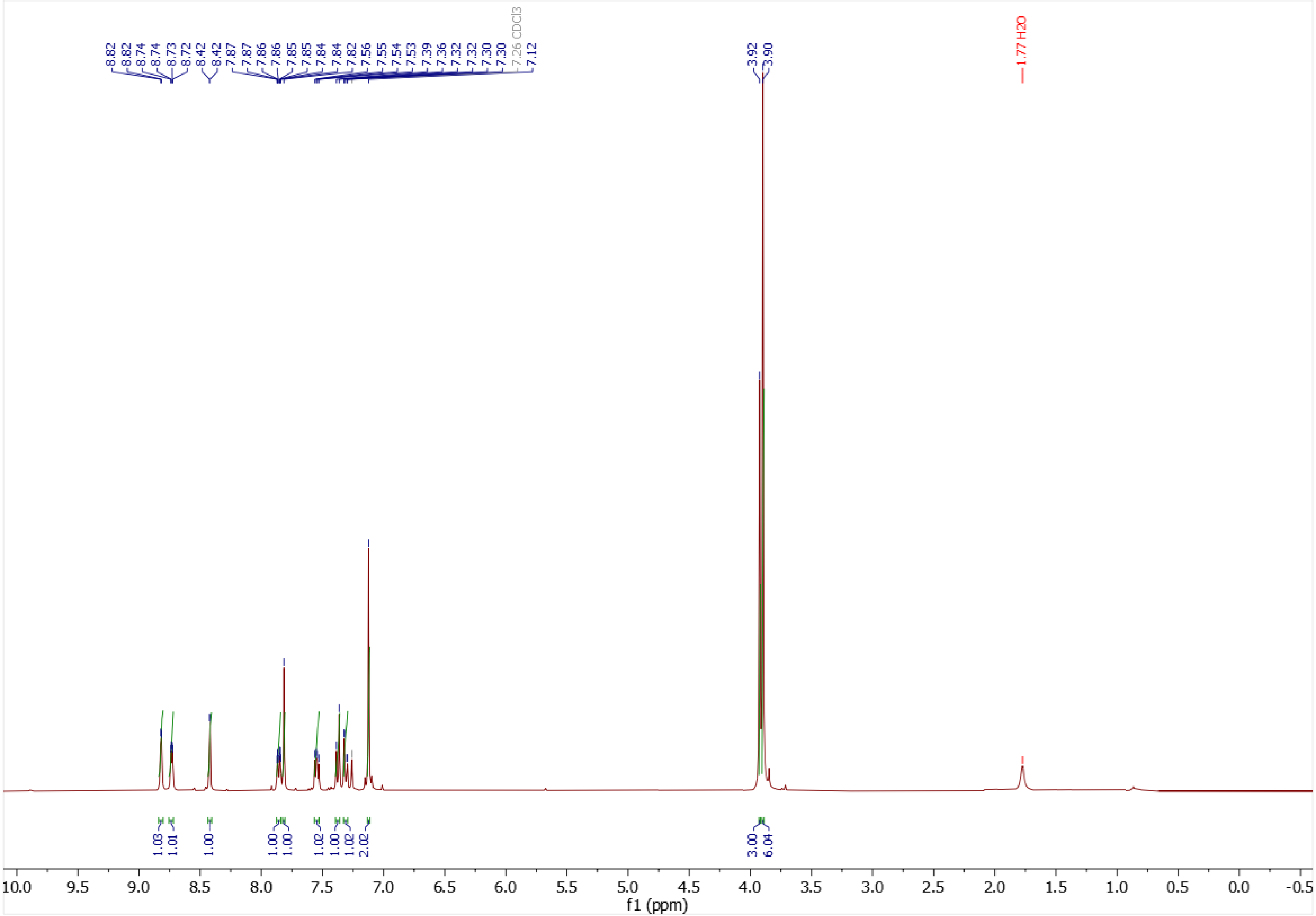
8.82
8.82
8.74
8.74
8.73
8.72
8.42
8.42
7.87
7.87
7.86
7.85
7.85
7.84
7.84
7.82
7.56
7.55
7.54
7.53
7.39
7.36
7.32
7.32
7.30
7.30
7.26 CDCl3
7.12
3.92
3.90
1.77 H2O
1.03
1.01
1.00
1.00
1.00
1.02
1.00
1.02
2.02
3.00
6.04
10.0
9.5
9.0
8.5
8.0
7.5
7.0
6.5
6.0
5.5
5.0
4.5
4.0
3.5
3.0
2.5
2.0
1.5
1.0
0.5
0.0
-0.5
f1 (ppm)

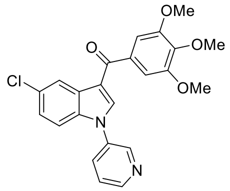

OMe
OMe
OMe
O
Cl
N
N

26

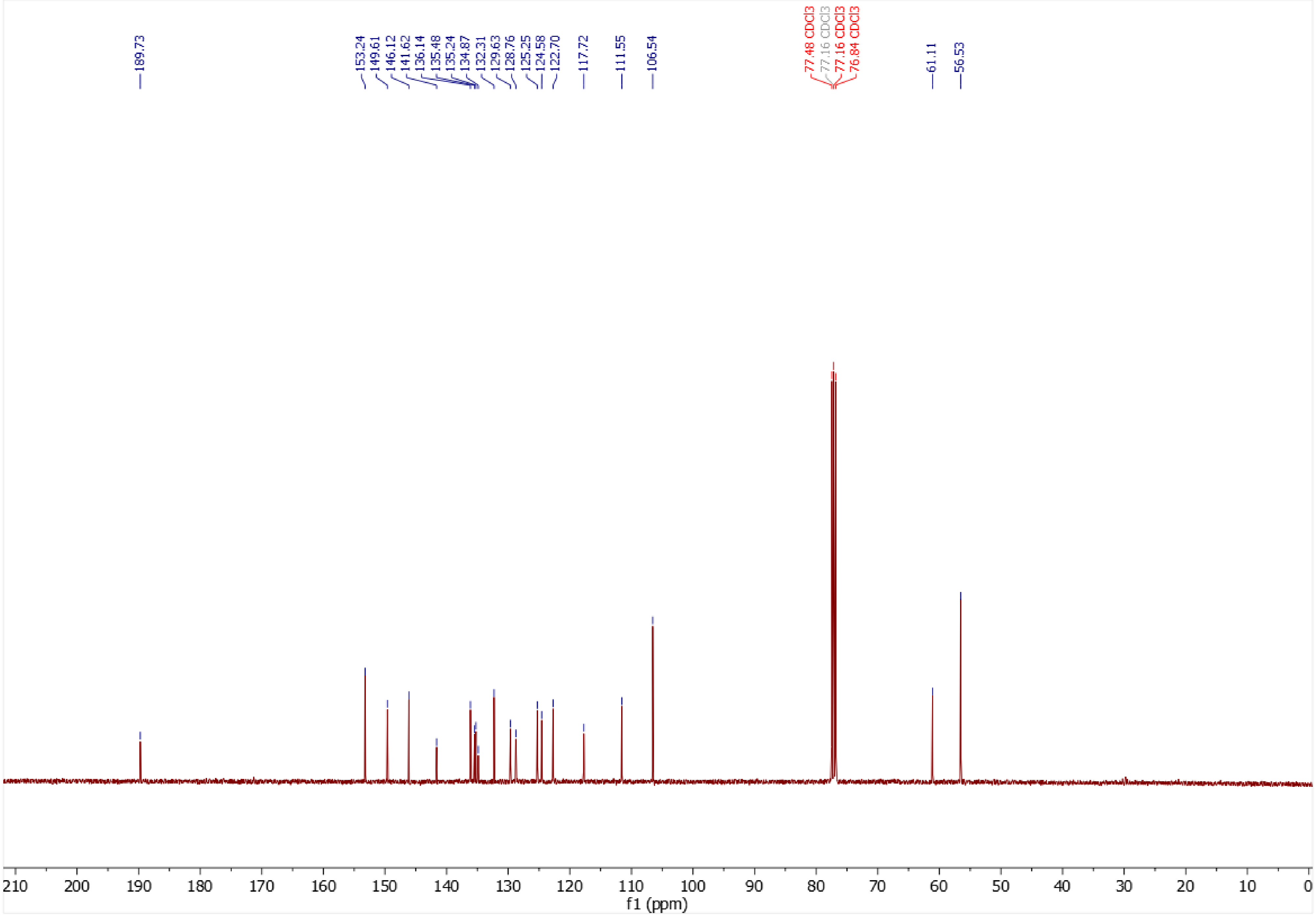

189.73
153.24
149.61
146.12
141.62
136.14
135.48
135.24
134.87
132.31
129.63
128.76
125.25
124.58
122.70
117.72
111.55
106.54
77.48 CDCl3
77.16 CDCl3
77.16 CDCl3
76.84 CDCl3
61.11
56.53
210 200 190 180 170 160 150 140 130 120 110 100 90 80 70 60 50 40 30 20 10 0
f1 (ppm)

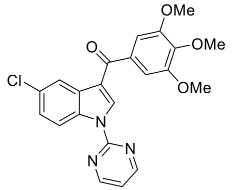

27

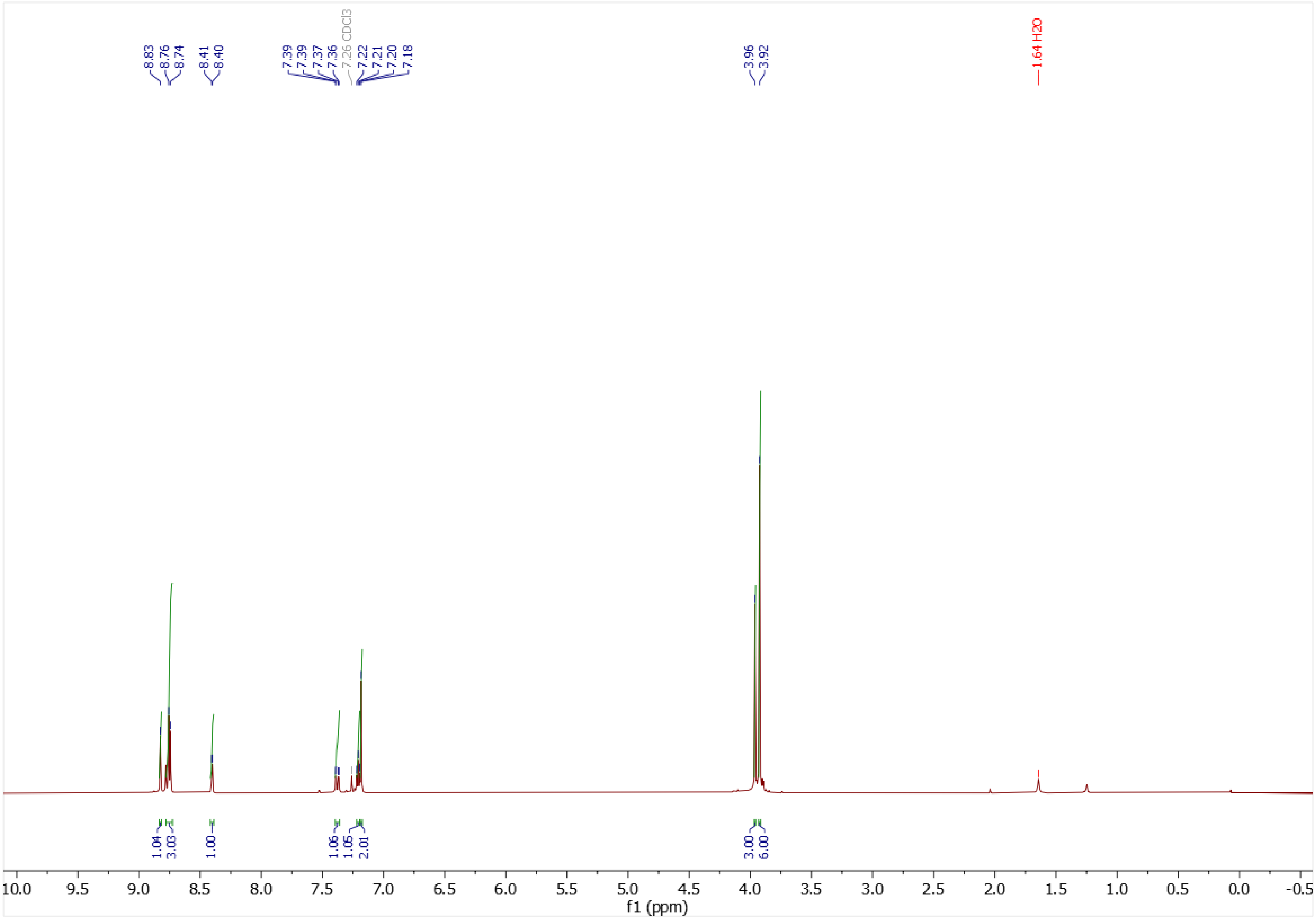

8.83
8.76
8.74
8.41
8.40
7.39
7.39
7.37
7.36
7.26 CDCl3
7.22
7.21
7.20
7.18
3.96
3.92
1.64 H2O
1.04
3.03
1.00
1.06
1.05
2.01
3.00
6.00
10.0 9.5 9.0 8.5 8.0 7.5 7.0 6.5 6.0 5.5 5.0 4.5 4.0 3.5 3.0 2.5 2.0 1.5 1.0 0.5 0.0 -0.5
f1 (ppm)

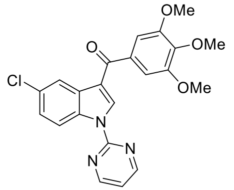
OMe
OMe
OMe
O
Cl
N
N
N

27

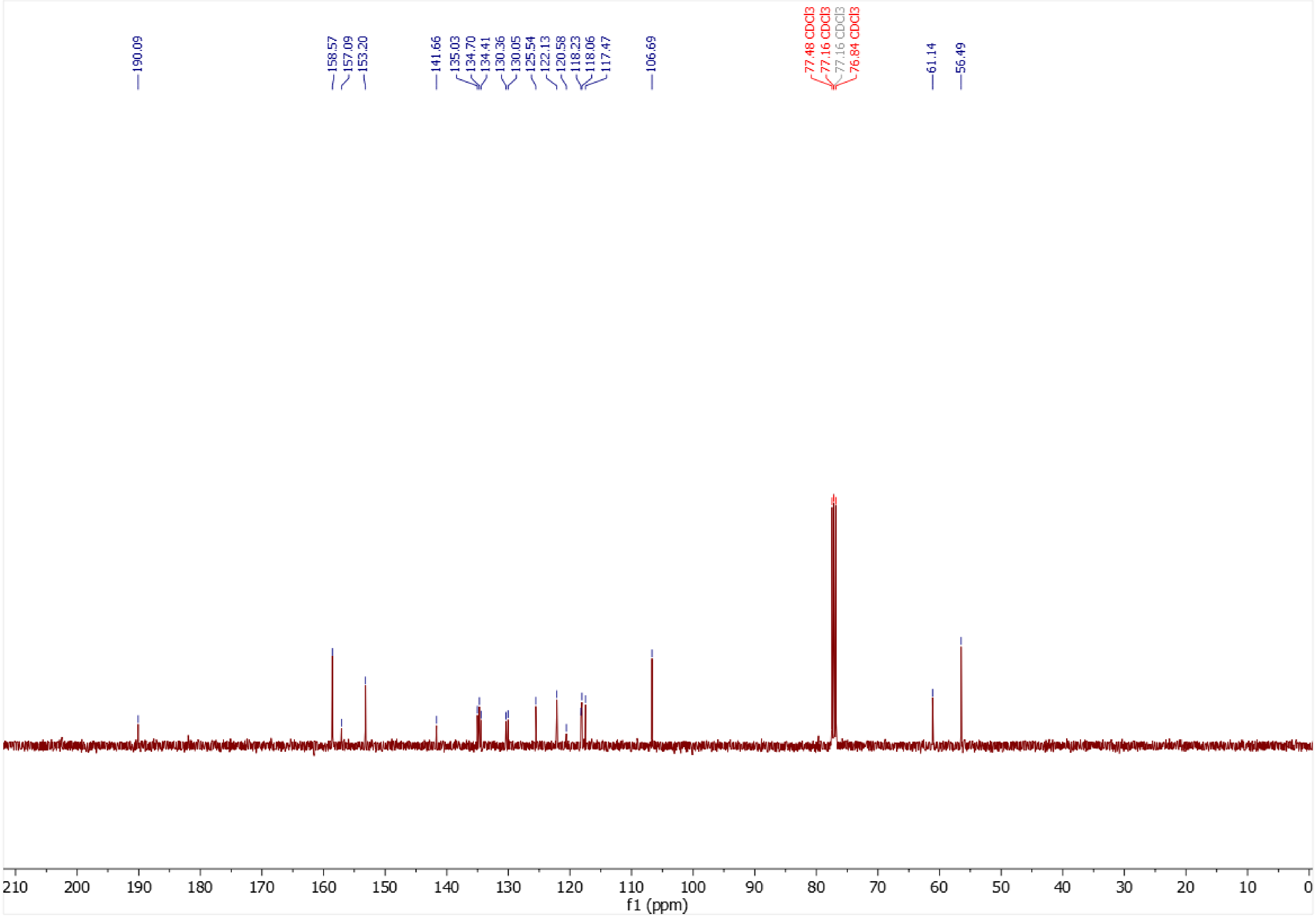
190.09
158.57
157.09
153.20
141.66
135.03
134.70
134.41
130.36
130.05
125.54
122.13
120.58
118.23
118.06
117.47
106.69
77.48 CDCl3
77.16 CDCl3
77.16 CDCl3
76.84 CDCl3
61.14
56.49
210
200
190
180
170
160
150
140
130
120
110
100
90
80
70
60
50
40
30
20
10
0
f1 (ppm)

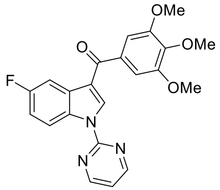
OMe
OMe
OMe
O
F
N
N
N

28

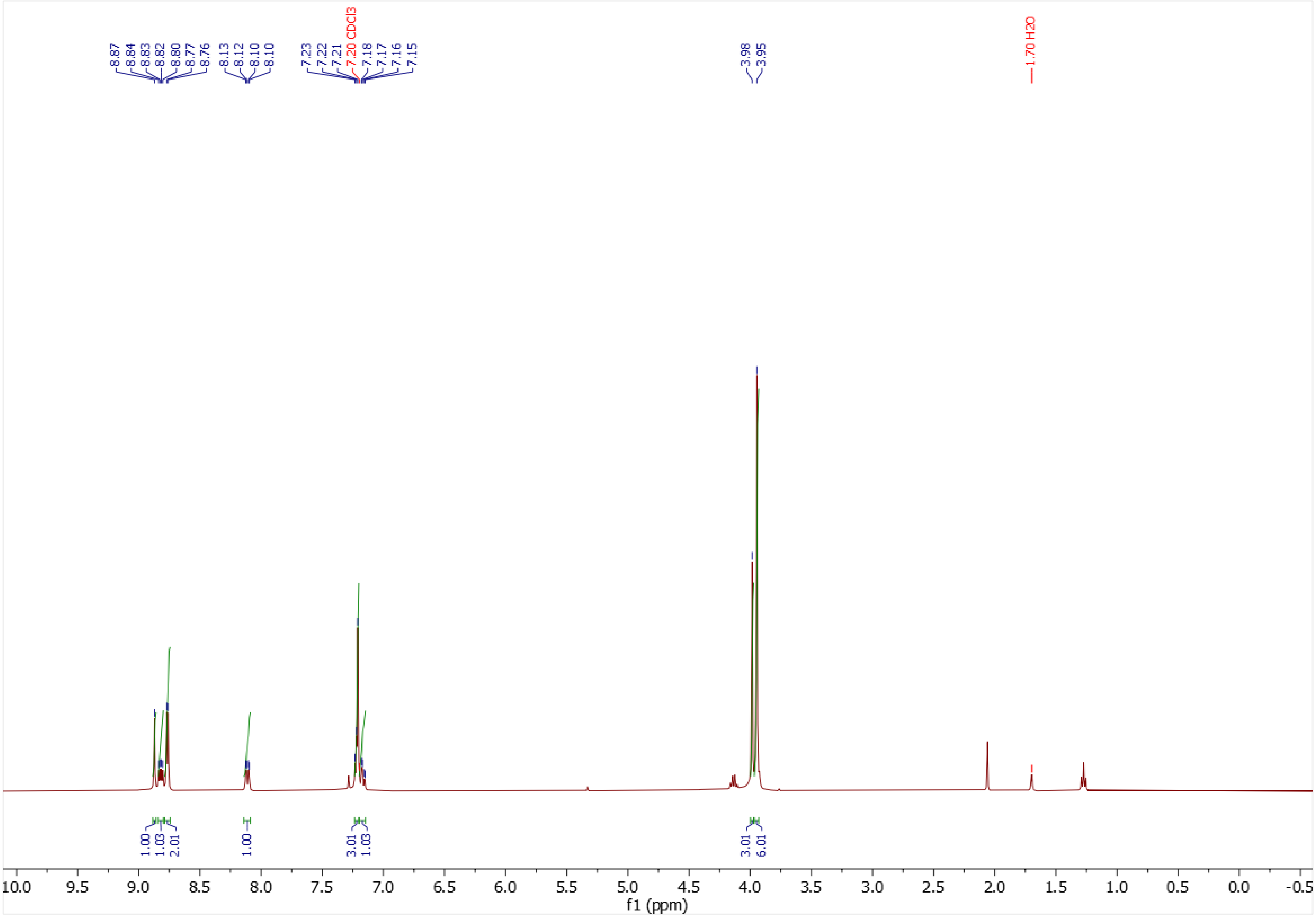
8.87
8.84
8.83
8.82
8.80
8.77
8.76
8.13
8.12
8.10
8.10
7.23
7.22
7.21
7.20 CDCl3
7.18
7.17
7.16
7.15
3.98
3.95
1.70 H2O
1.00
1.03
2.01
1.00
3.01
1.03
3.01
6.01
10.0 9.5 9.0 8.5 8.0 7.5 7.0 6.5 6.0 5.5 5.0 4.5 4.0 3.5 3.0 2.5 2.0 1.5 1.0 0.5 0.0 -0.5
f1 (ppm)

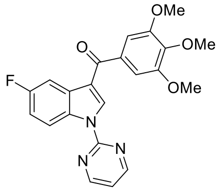
OMe
OMe
OMe
O
F
N
N
N
28

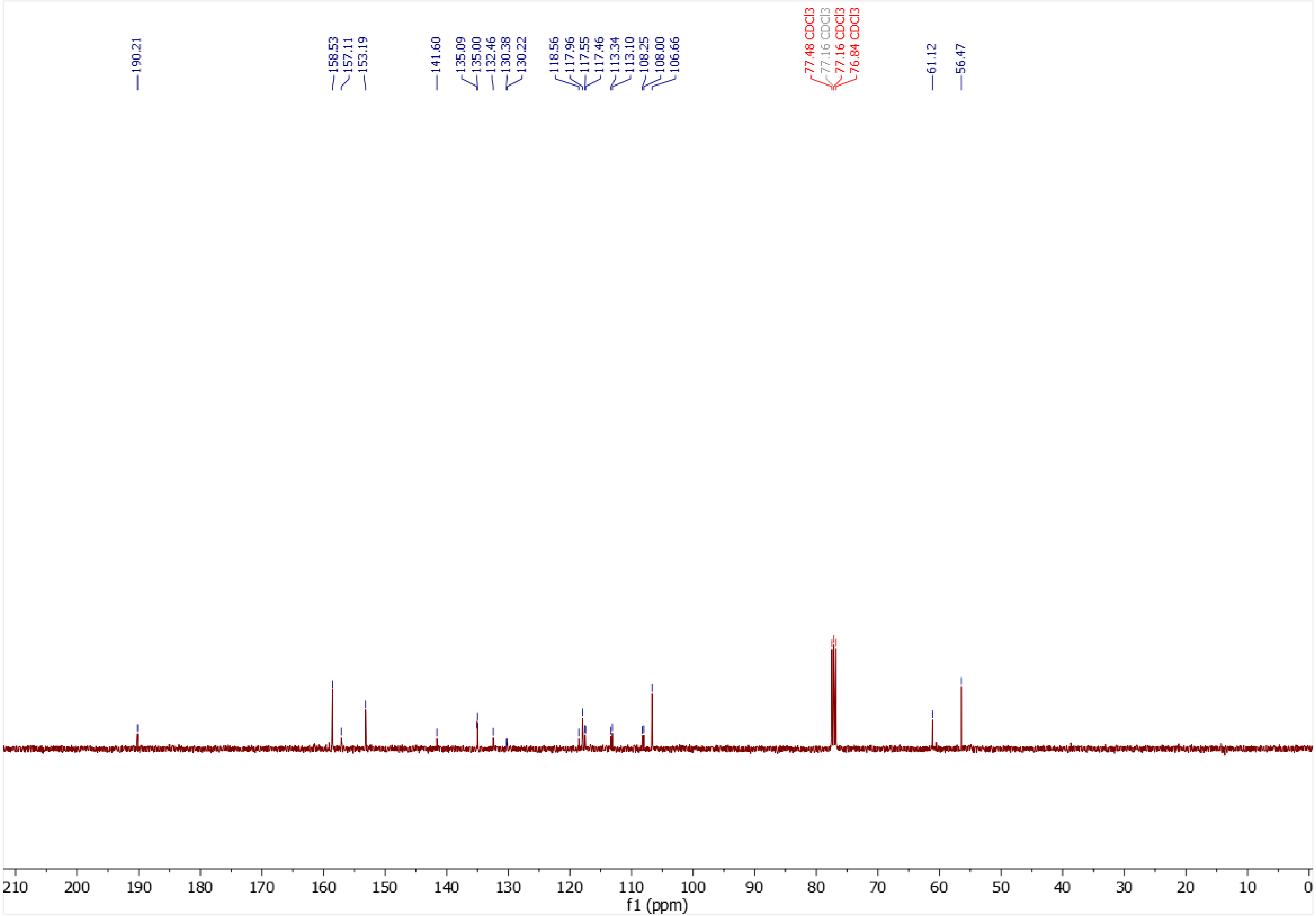
190.21
158.53
157.11
153.19
141.60
135.09
135.00
132.46
130.38
130.22
118.56
117.96
117.55
117.46
113.34
113.10
108.25
108.00
106.66
77.48 CDCl3
77.16 CDCl3
77.16 CDCl3
76.84 CDCl3
61.12
56.47
210
200
190
180
170
160
150
140
130
120
110
100
90
80
70
60
50
40
30
20
10
0
f1 (ppm)

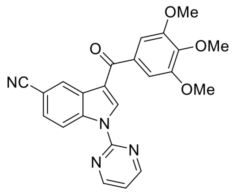
OMe
OMe
OMe
O
NC
N
N
N

29

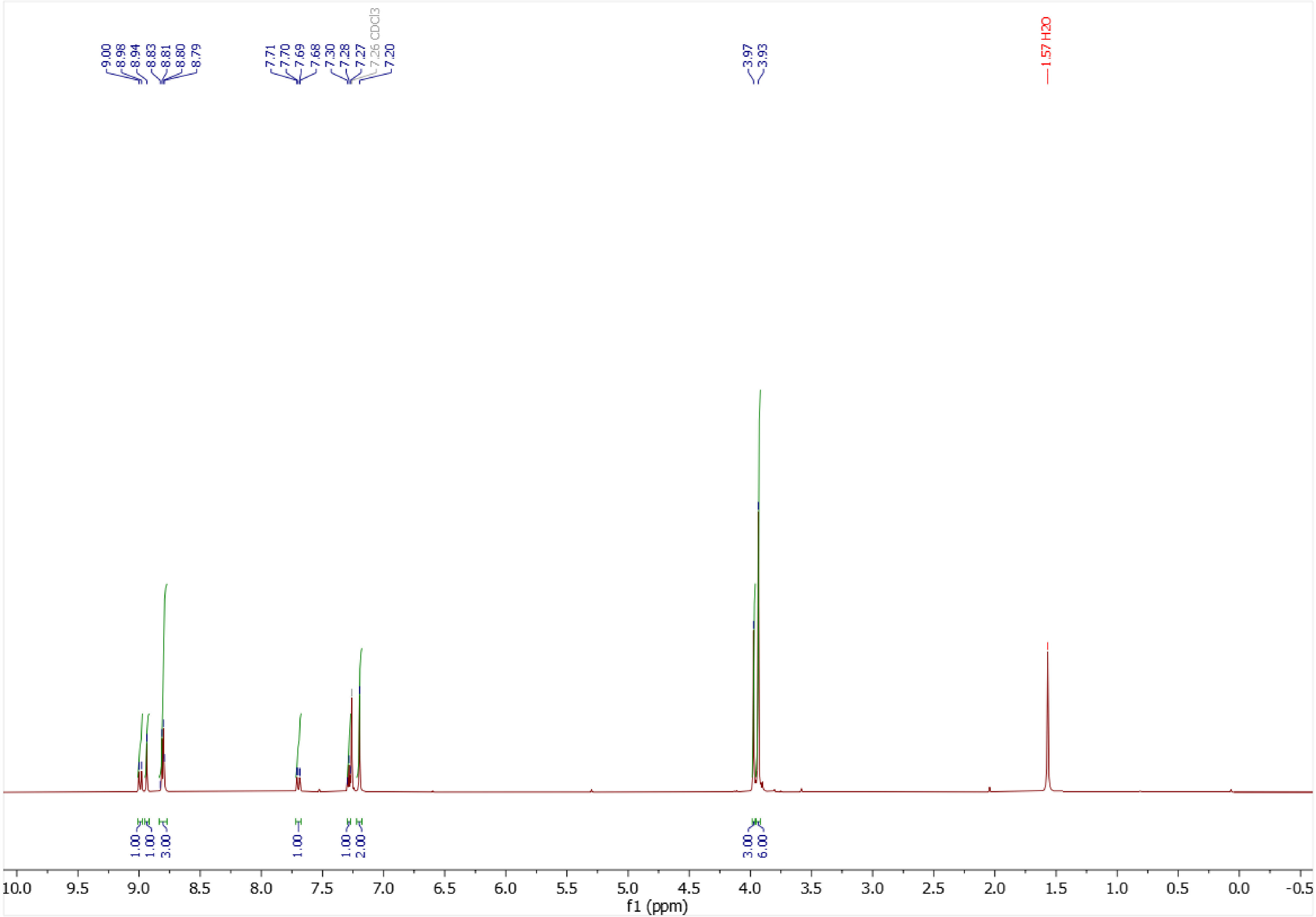
9.00
8.98
8.94
8.83
8.81
8.80
8.79
7.71
7.70
7.69
7.68
7.30
7.28
7.27
7.26 CDCl3
7.20
3.97
3.93
1.57 H2O
1.00
1.00
3.00
1.00
1.00
2.00
3.00
6.00
10.0 9.5 9.0 8.5 8.0 7.5 7.0 6.5 6.0 5.5 5.0 4.5 4.0 3.5 3.0 2.5 2.0 1.5 1.0 0.5 0.0 -0.5
f1 (ppm)

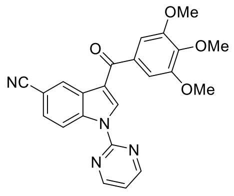
OMe
O
OMe
NC
OMe
N
N
N

29

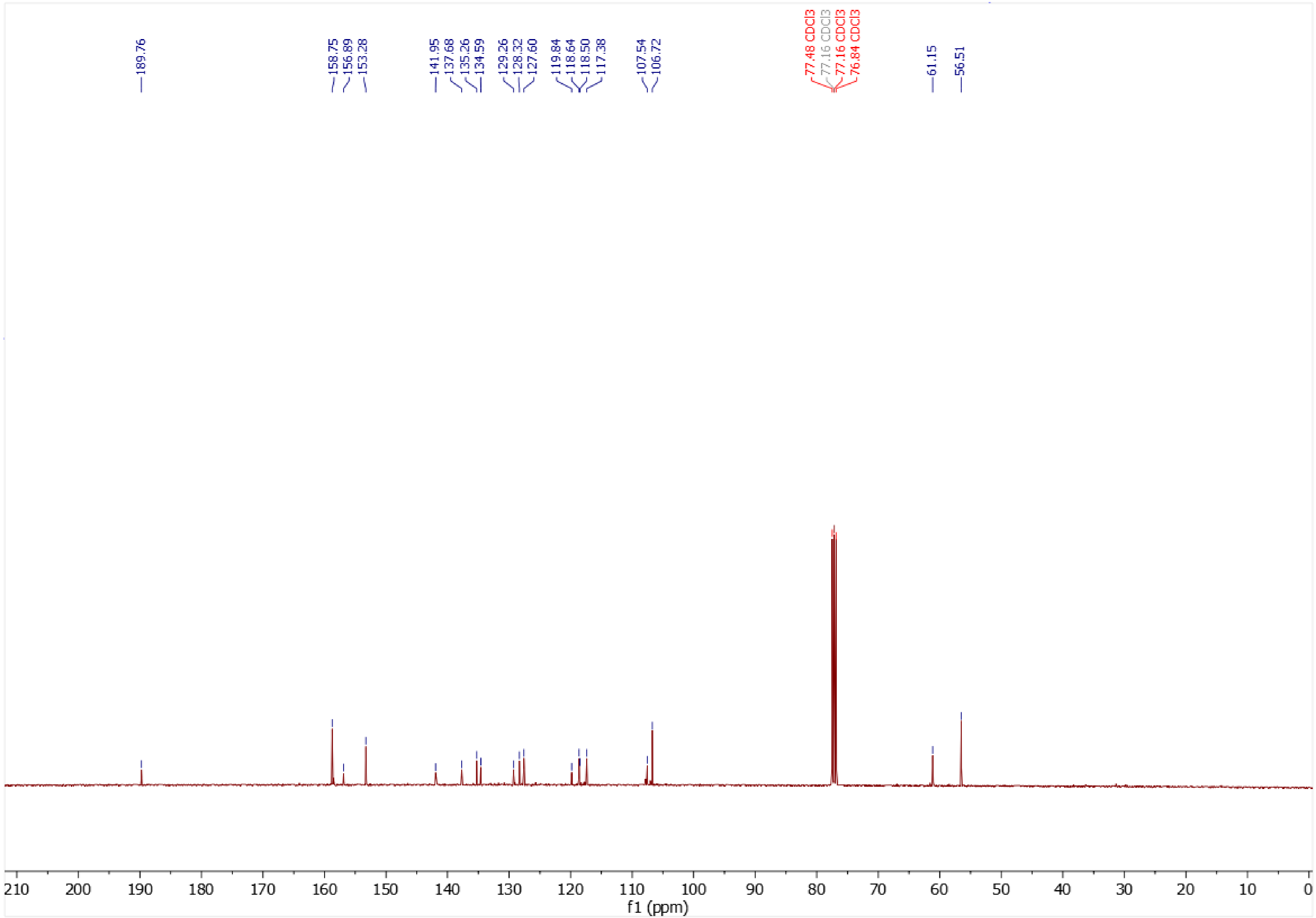
189.76
158.75
156.89
153.28
141.95
137.68
135.26
134.59
129.26
128.32
127.60
119.84
118.64
118.50
117.38
107.54
106.72
77.48 CDCl3
77.16 CDCl3
77.16 CDCl3
76.84 CDCl3
61.15
56.51
210
200
190
180
170
160
150
140
130
120
110
100
90
80
70
60
50
40
30
20
10
0
f1 (ppm)

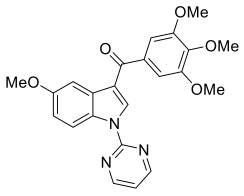
OMe
OMe
MeO
OMe
O
N
N
N

30

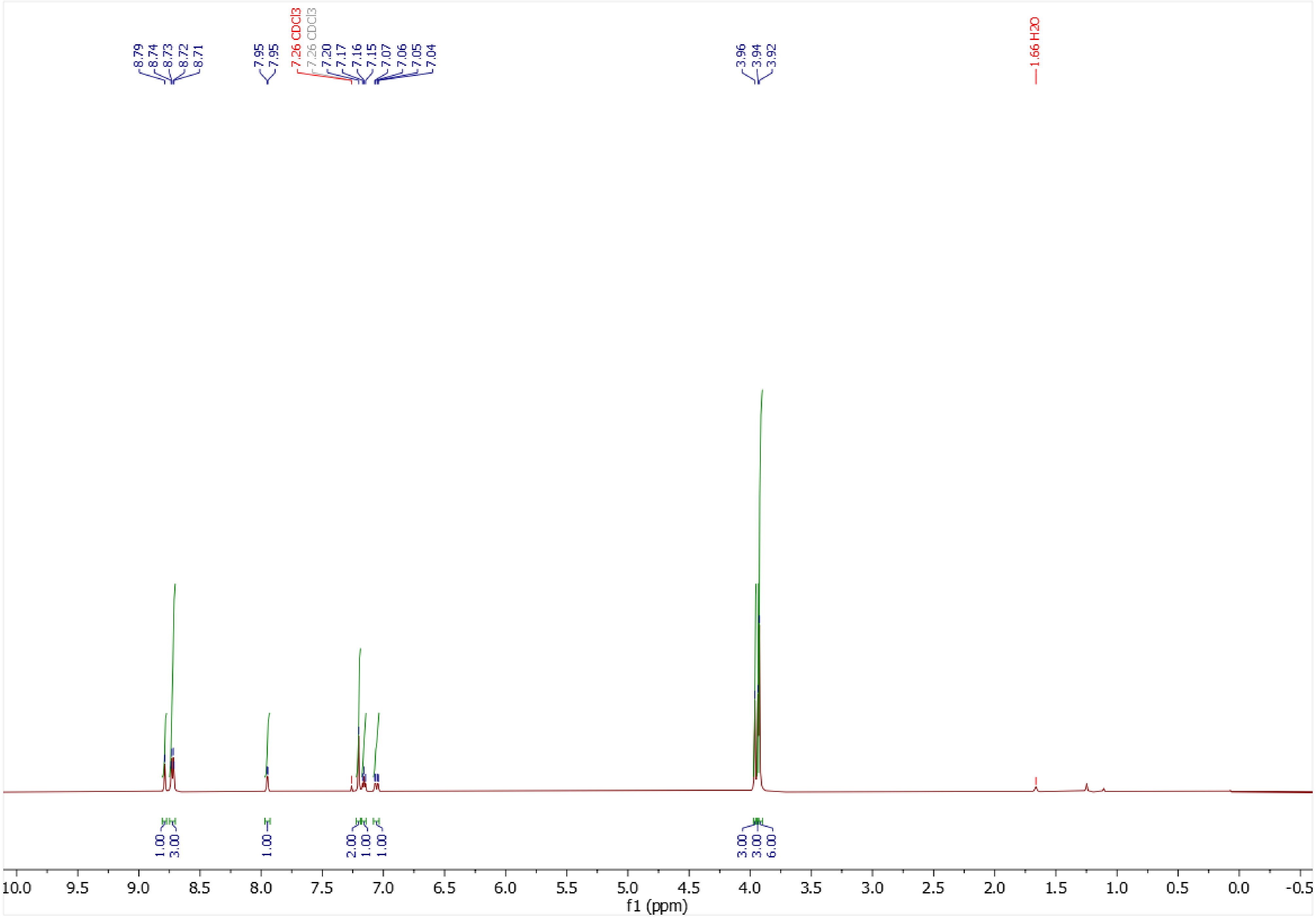
8.79
8.74
8.73
8.72
8.71
7.95
7.95
7.26 CDCl3
7.26 CDCl3
7.20
7.17
7.16
7.15
7.07
7.06
7.05
7.04
3.96
3.94
3.92
1.66 H2O
1.00
3.00
1.00
2.00
1.00
1.00
3.00
3.00
6.00
10.0 9.5 9.0 8.5 8.0 7.5 7.0 6.5 6.0 5.5 5.0 4.5 4.0 3.5 3.0 2.5 2.0 1.5 1.0 0.5 0.0 -0.5
f1 (ppm)

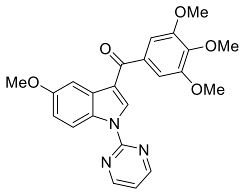
OMe
OMe
OMe
MeO
O
N
N
N

30

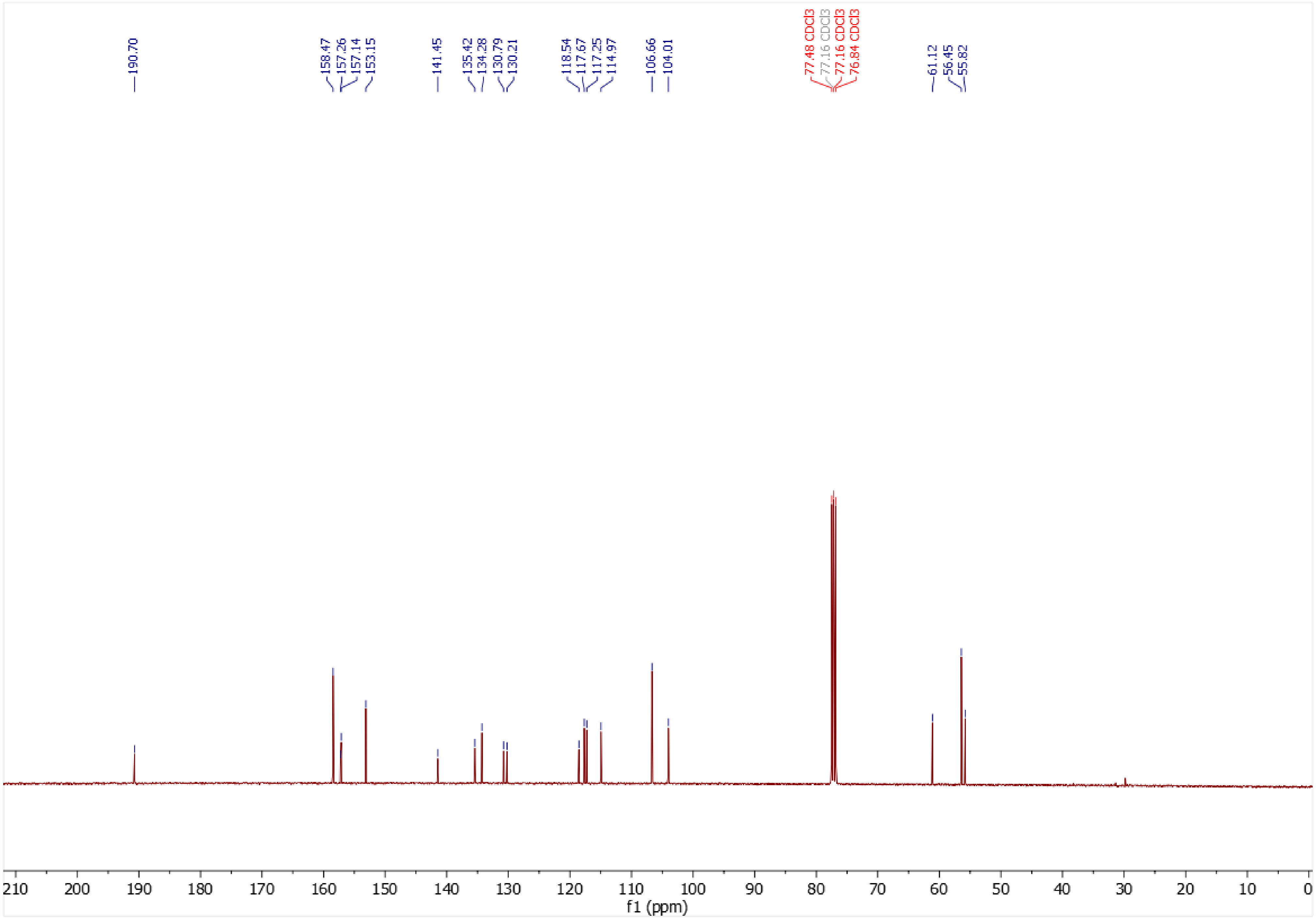
190.70
158.47
157.26
157.14
153.15
141.45
135.42
134.28
130.79
130.21
118.54
117.67
117.25
114.97
106.66
104.01
77.48 CDCl3
77.16 CDCl3
77.16 CDCl3
76.84 CDCl3
61.12
56.45
55.82
210 200 190 180 170 160 150 140 130 120 110 100 90 80 70 60 50 40 30 20 10 0
f1 (ppm)

IR Spectra of Pyrroles **3-24** and Indoles **25-30**

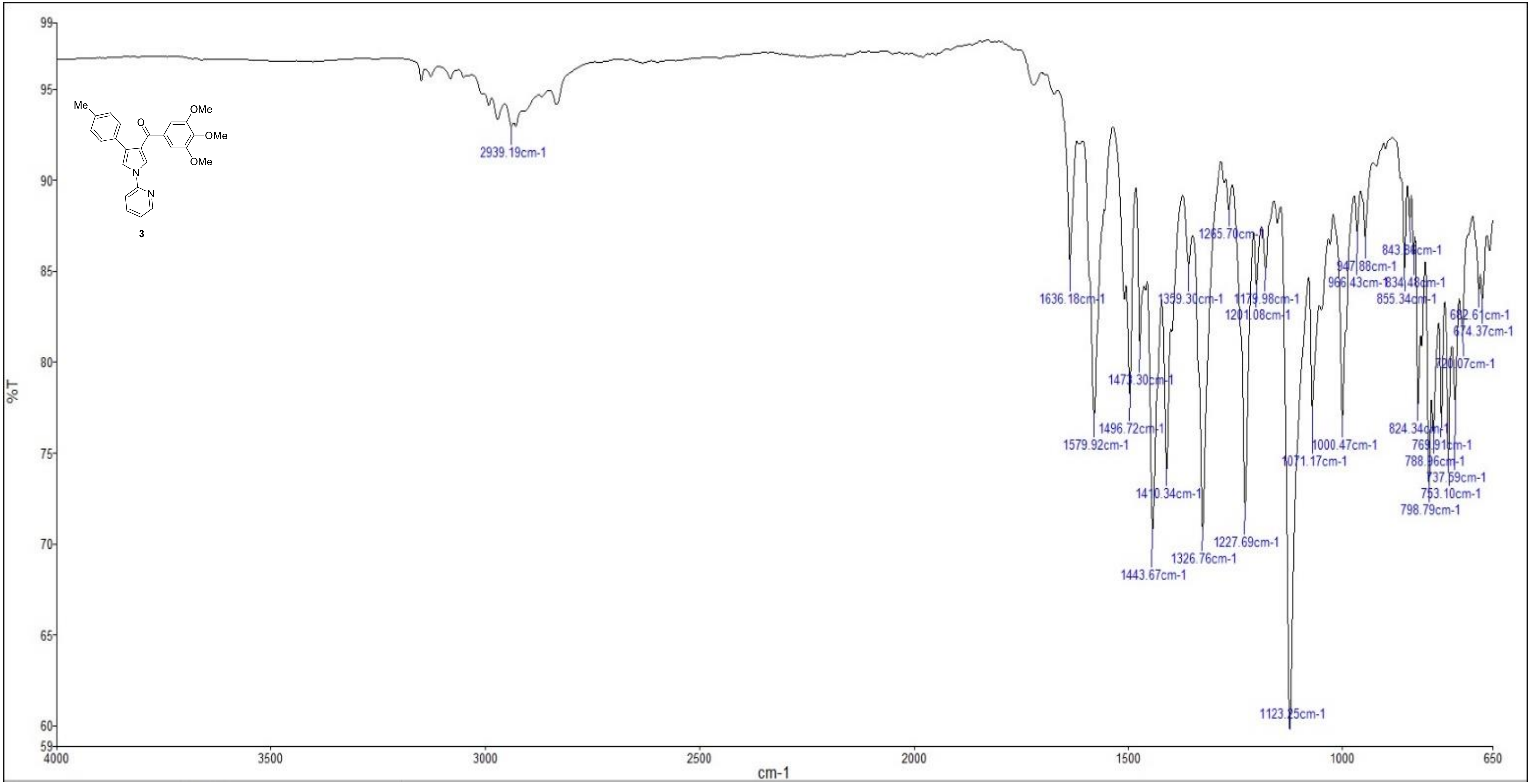

%T
cm-1
2939.19cm-1
1636.18cm-1
1579.92cm-1
1496.72cm-1
1473.30cm-1
1443.67cm-1
1410.34cm-1
1359.30cm-1
1326.76cm-1
1265.70cm-1
1227.69cm-1
1201.08cm-1
1179.98cm-1
1123.25cm-1
1071.17cm-1
1000.47cm-1
966.43cm-1
947.88cm-1
855.34cm-1
843.86cm-1
834.48cm-1
824.34cm-1
798.79cm-1
788.96cm-1
769.91cm-1
753.10cm-1
737.59cm-1
720.07cm-1
682.61cm-1
674.37cm-1
Me
OMe
OMe
OMe
O
N
N
3

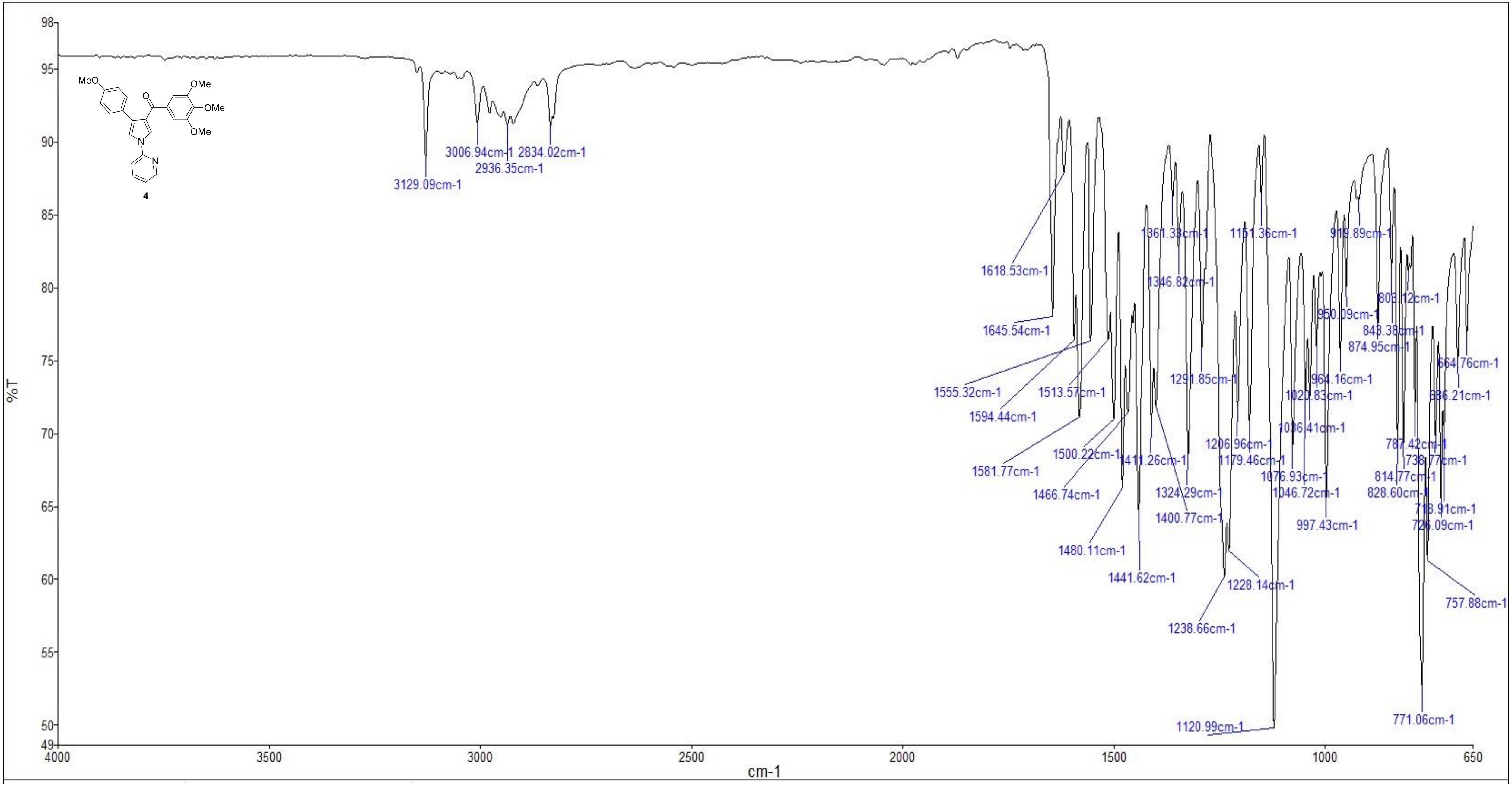

MeO
OMe
O
OMe
OMe
N
N
4
98
95
90
85
80
75
70
65
60
55
50
49
%T
4000
3500
3000
2500
2000
1500
1000
650
cm-1
3129.09cm-1
3006.94cm-1
2936.35cm-1
2834.02cm-1
1645.54cm-1
1618.53cm-1
1594.44cm-1
1581.77cm-1
1555.32cm-1
1513.57cm-1
1500.22cm-1
1480.11cm-1
1466.74cm-1
1441.62cm-1
1411.26cm-1
1400.77cm-1
1361.33cm-1
1346.82cm-1
1324.29cm-1
1291.85cm-1
1238.66cm-1
1228.14cm-1
1206.96cm-1
1179.46cm-1
1151.36cm-1
1120.99cm-1
1076.93cm-1
1046.72cm-1
1036.41cm-1
1020.83cm-1
997.43cm-1
964.16cm-1
950.09cm-1
919.89cm-1
874.95cm-1
843.38cm-1
828.60cm-1
814.77cm-1
803.12cm-1
787.42cm-1
771.06cm-1
757.88cm-1
738.77cm-1
726.09cm-1
718.91cm-1
686.21cm-1
664.76cm-1

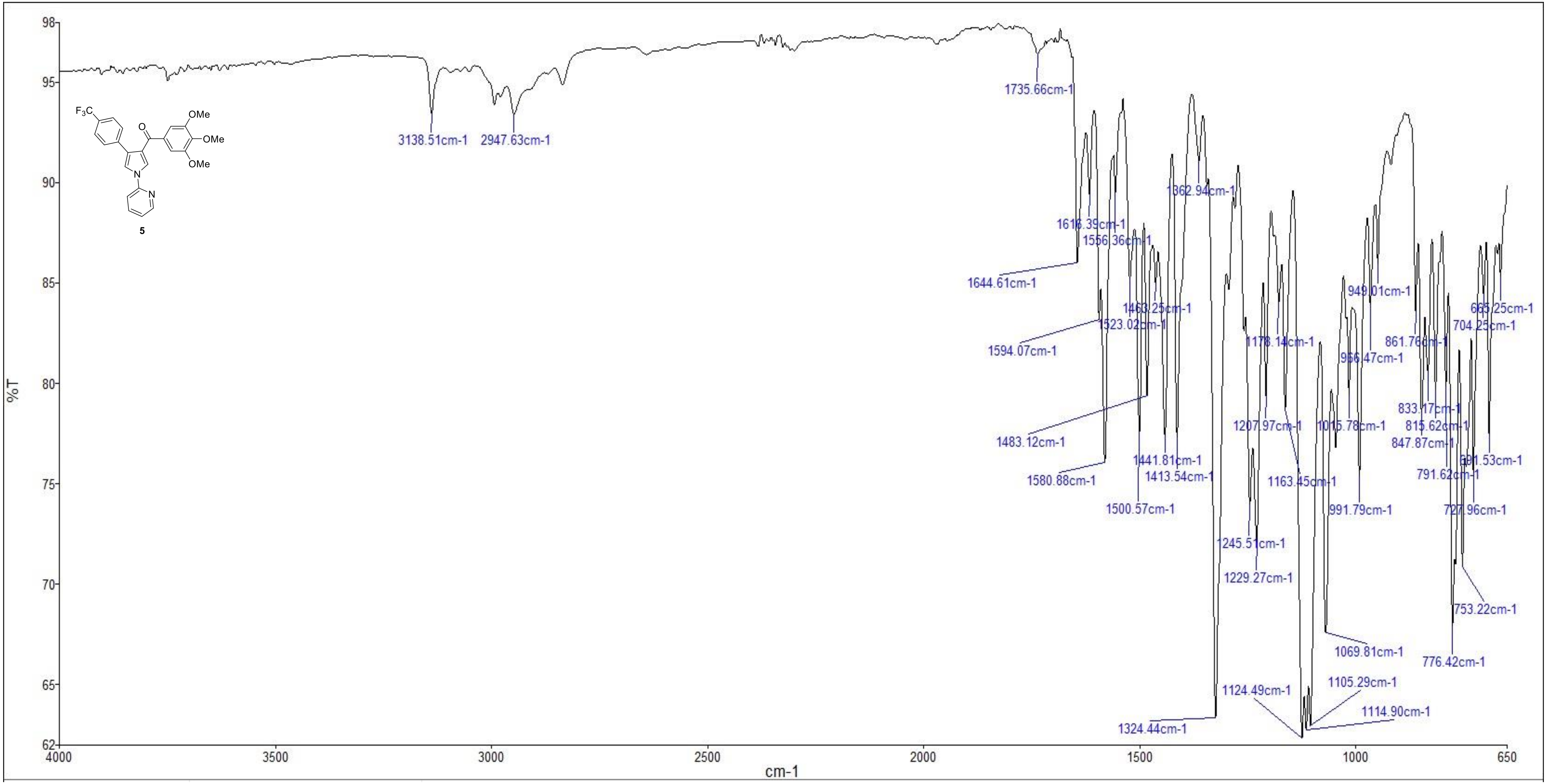

%T
cm-1
98
95
90
85
80
75
70
65
62
4000
3500
3000
2500
2000
1500
1000
650
F3C
OMe
OMe
OMe
O
N
N
5
3138.51cm-1
2947.63cm-1
1735.66cm-1
1644.61cm-1
1556.36cm-1
1594.07cm-1
1523.02cm-1
1483.12cm-1
1580.88cm-1
1500.57cm-1
1441.81cm-1
1413.54cm-1
1362.94cm-1
1324.44cm-1
1245.51cm-1
1229.27cm-1
1207.97cm-1
1178.14cm-1
1163.45cm-1
1124.49cm-1
1114.90cm-1
1105.29cm-1
1069.81cm-1
1015.78cm-1
991.79cm-1
966.47cm-1
949.01cm-1
861.76cm-1
847.87cm-1
833.17cm-1
815.62cm-1
791.62cm-1
776.42cm-1
753.22cm-1
727.96cm-1
704.25cm-1
665.25cm-1

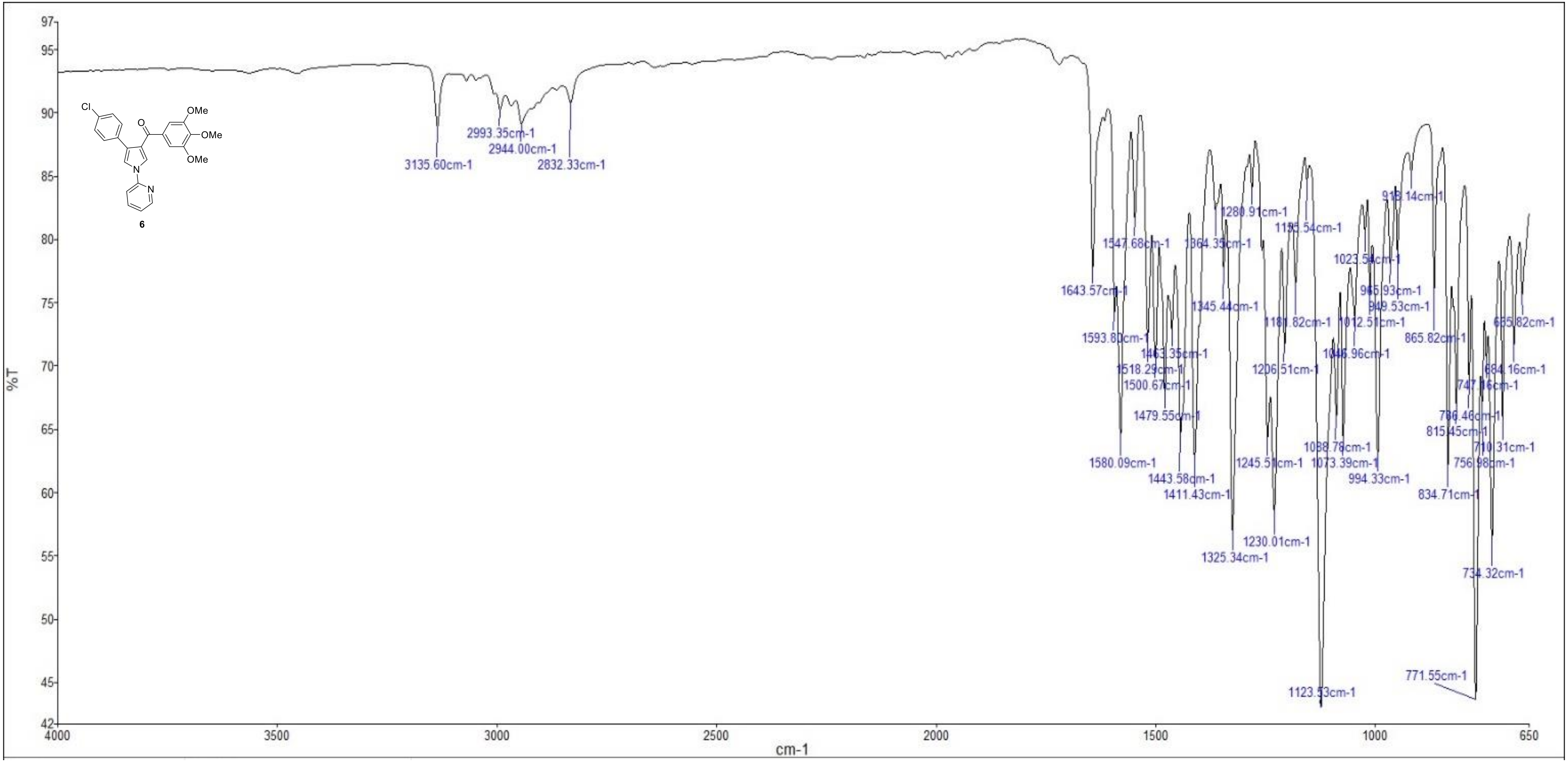
97
95
90
85
80
75
70
65
60
55
50
45
42
%T
4000
3500
3000
2500
2000
1500
1000
650
cm-1
3135.60cm-1
2993.35cm-1
2944.00cm-1
2832.33cm-1
1643.57cm-1
1593.80cm-1
1580.09cm-1
1547.68cm-1
1518.29cm-1
1500.67cm-1
1479.55cm-1
1463.35cm-1
1443.58cm-1
1411.43cm-1
1364.35cm-1
1345.44cm-1
1325.34cm-1
1280.91cm-1
1245.51cm-1
1230.01cm-1
1206.51cm-1
1181.82cm-1
1155.54cm-1
1123.53cm-1
1088.78cm-1
1073.39cm-1
1046.96cm-1
1023.54cm-1
1012.51cm-1
994.33cm-1
965.93cm-1
949.53cm-1
918.14cm-1
865.82cm-1
834.71cm-1
815.45cm-1
786.46cm-1
771.55cm-1
756.98cm-1
747.36cm-1
734.32cm-1
710.31cm-1
684.16cm-1
665.82cm-1
Cl
O
OMe
OMe
OMe
N
N
6

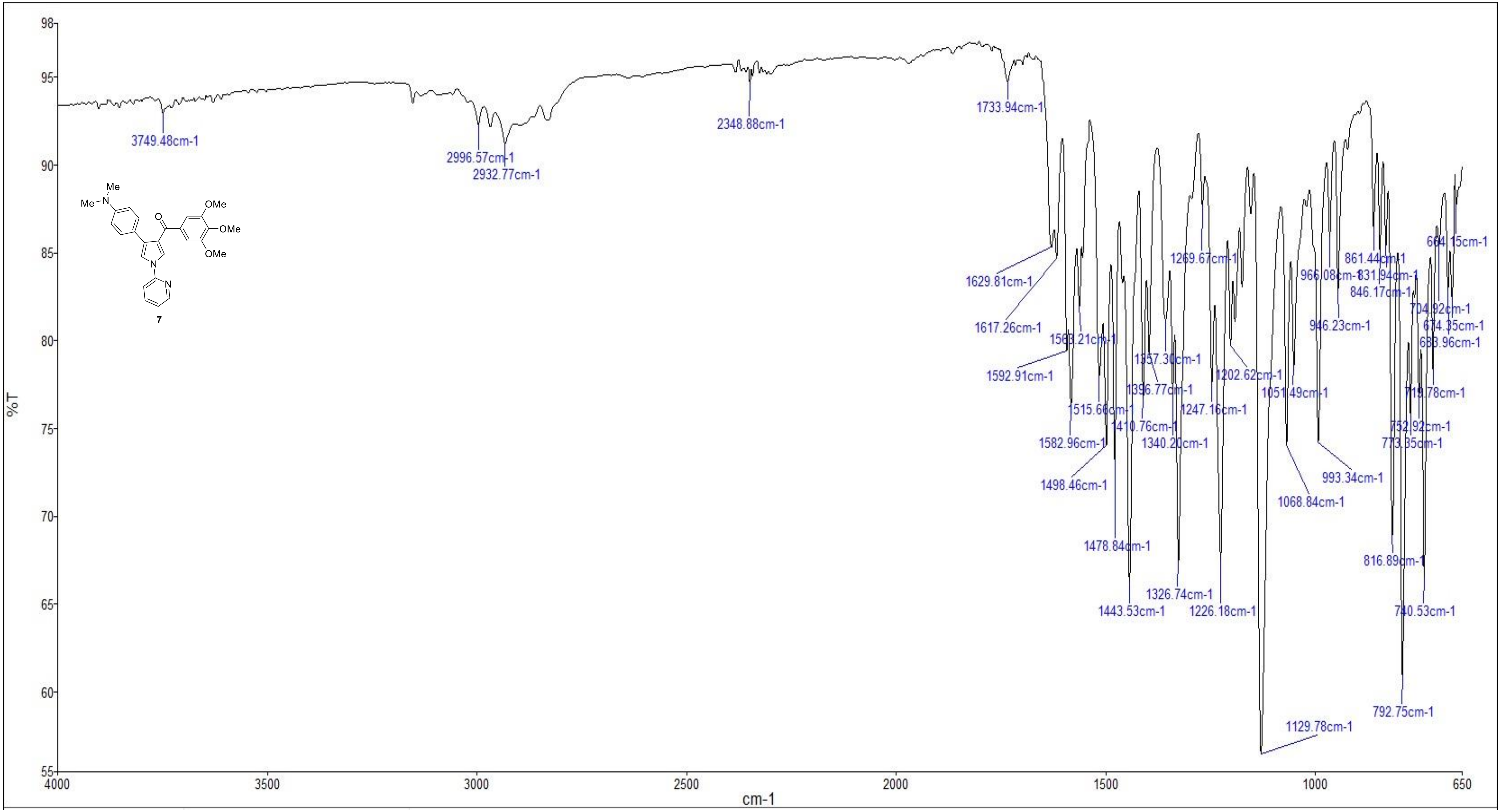

Me
Me
N
O
OMe
OMe
OMe
N
N
7
%T
cm-1
3749.48cm-1
2996.57cm-1
2932.77cm-1
2348.88cm-1
1733.94cm-1
1629.81cm-1
1617.26cm-1
1592.91cm-1
1582.96cm-1
1515.66cm-1
1498.46cm-1
1478.84cm-1
1443.53cm-1
1410.76cm-1
1396.77cm-1
1340.20cm-1
1326.74cm-1
1269.67cm-1
1247.16cm-1
1226.18cm-1
1202.62cm-1
1129.78cm-1
1068.84cm-1
1051.49cm-1
993.34cm-1
966.08cm-1
946.23cm-1
861.44cm-1
846.17cm-1
831.94cm-1
816.89cm-1
792.75cm-1
773.35cm-1
752.92cm-1
740.53cm-1
719.78cm-1
704.92cm-1
674.35cm-1
664.15cm-1

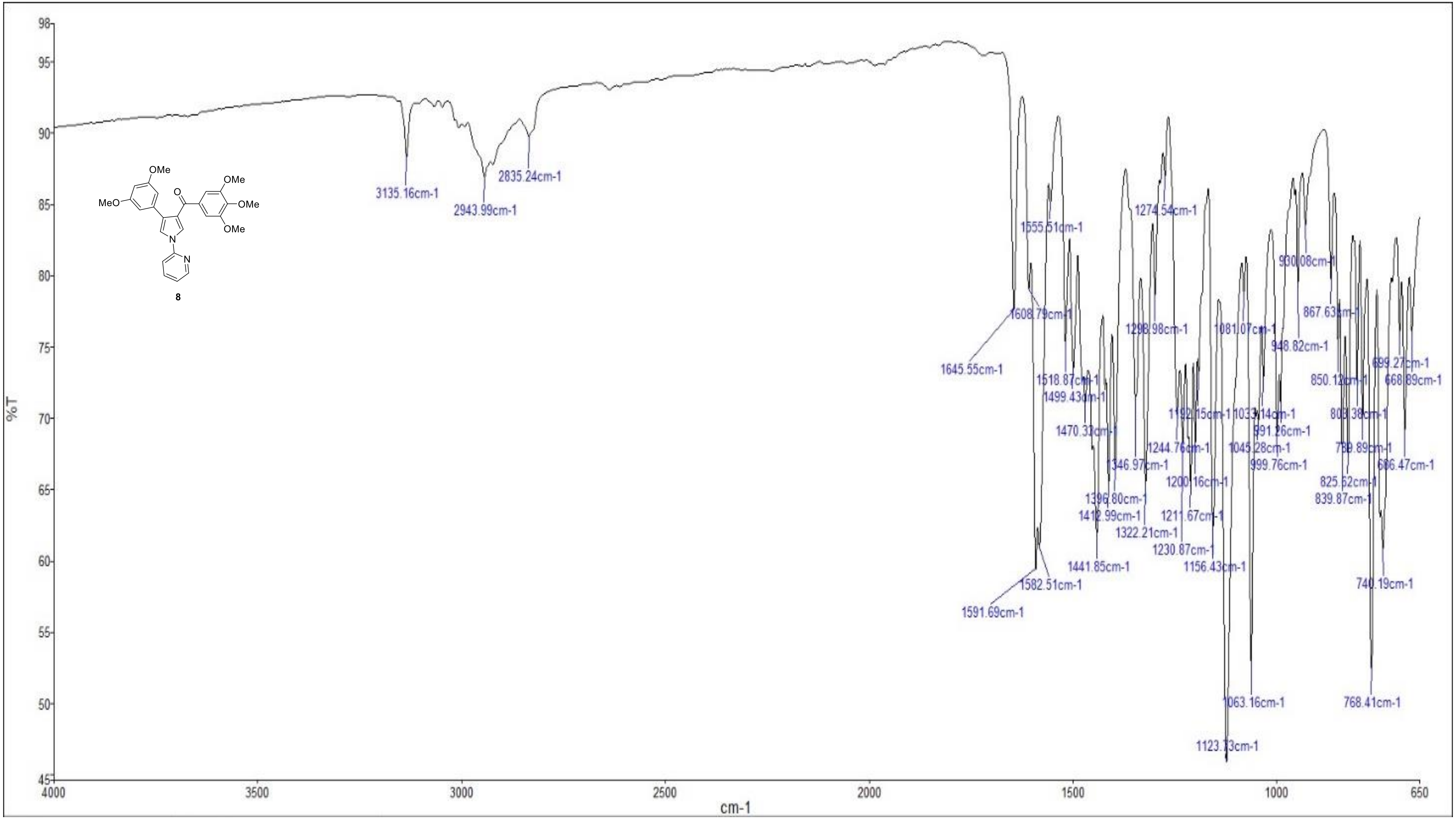
OMe
MeO
O
OMe
OMe
OMe
N
N
8
%T
cm-1
3135.16cm-1
2943.99cm-1
2835.24cm-1
1645.55cm-1
1608.79cm-1
1591.69cm-1
1582.51cm-1
1555.51cm-1
1518.87cm-1
1499.43cm-1
1470.33cm-1
1441.85cm-1
1412.99cm-1
1396.80cm-1
1346.97cm-1
1322.21cm-1
1298.98cm-1
1274.54cm-1
1244.76cm-1
1230.87cm-1
1211.67cm-1
1200.16cm-1
1192.15cm-1
1156.43cm-1
1123.73cm-1
1081.07cm-1
1063.16cm-1
1045.28cm-1
1033.14cm-1
999.76cm-1
991.26cm-1
948.82cm-1
930.08cm-1
867.63cm-1
850.12cm-1
839.87cm-1
825.52cm-1
803.38cm-1
789.89cm-1
768.41cm-1
740.19cm-1
699.27cm-1
686.47cm-1
668.89cm-1

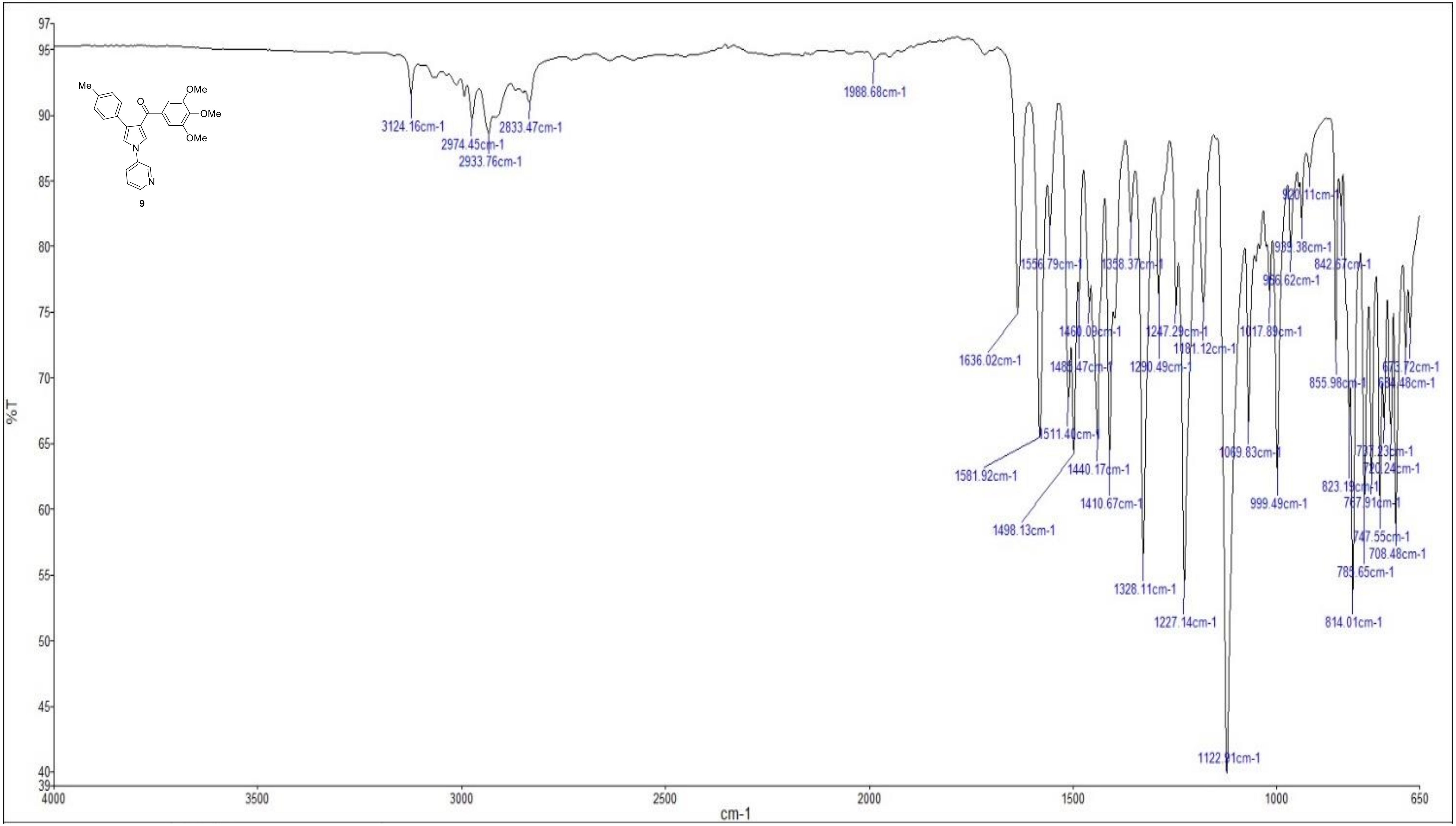

Me
OMe
O
OMe
OMe
N
N
9
%T
cm-1
3124.16cm-1
2974.45cm-1
2933.76cm-1
2833.47cm-1
1988.68cm-1
1636.02cm-1
1581.92cm-1
1556.79cm-1
1511.40cm-1
1498.13cm-1
1440.17cm-1
1410.67cm-1
1358.37cm-1
1328.11cm-1
1290.49cm-1
1247.29cm-1
1227.14cm-1
1181.12cm-1
1122.91cm-1
1069.83cm-1
1017.89cm-1
999.49cm-1
855.98cm-1
842.67cm-1
823.19cm-1
814.01cm-1
720.24cm-1
708.48cm-1

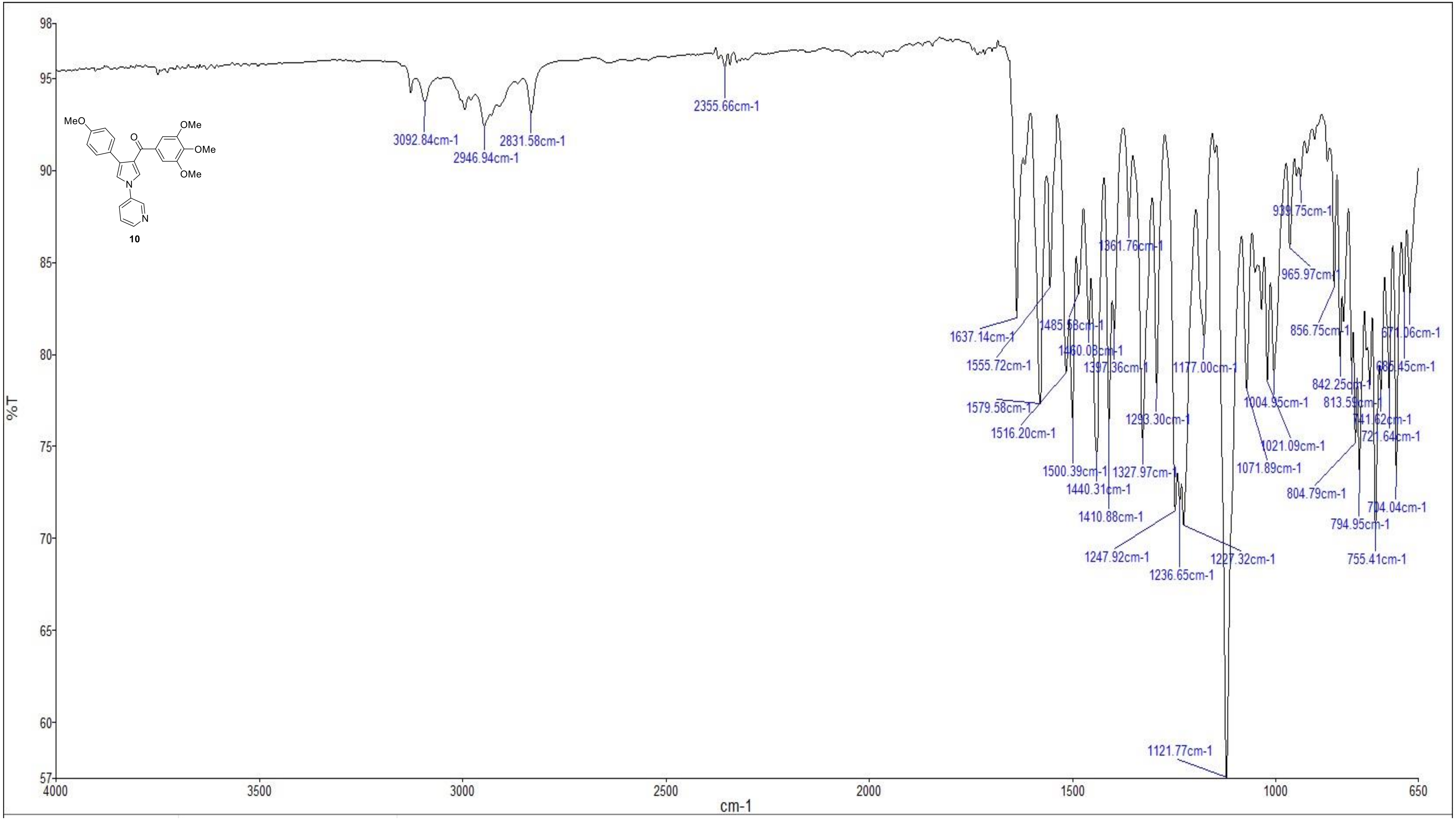
MeO
OMe
OMe
OMe
O
N
N
10
%T
cm-1
3092.84cm-1
2946.94cm-1
2831.58cm-1
2355.66cm-1
1637.14cm-1
1555.72cm-1
1579.58cm-1
1516.20cm-1
1500.39cm-1
1485.58cm-1
1460.08cm-1
1440.31cm-1
1410.88cm-1
1397.36cm-1
1361.76cm-1
1327.97cm-1
1293.30cm-1
1247.92cm-1
1236.65cm-1
1227.32cm-1
1177.00cm-1
1121.77cm-1
1071.89cm-1
1021.09cm-1
1004.95cm-1
965.97cm-1
939.75cm-1
856.75cm-1
842.25cm-1
813.59cm-1
804.79cm-1
794.95cm-1
755.41cm-1
741.62cm-1
721.64cm-1
704.04cm-1
686.45cm-1
671.06cm-1

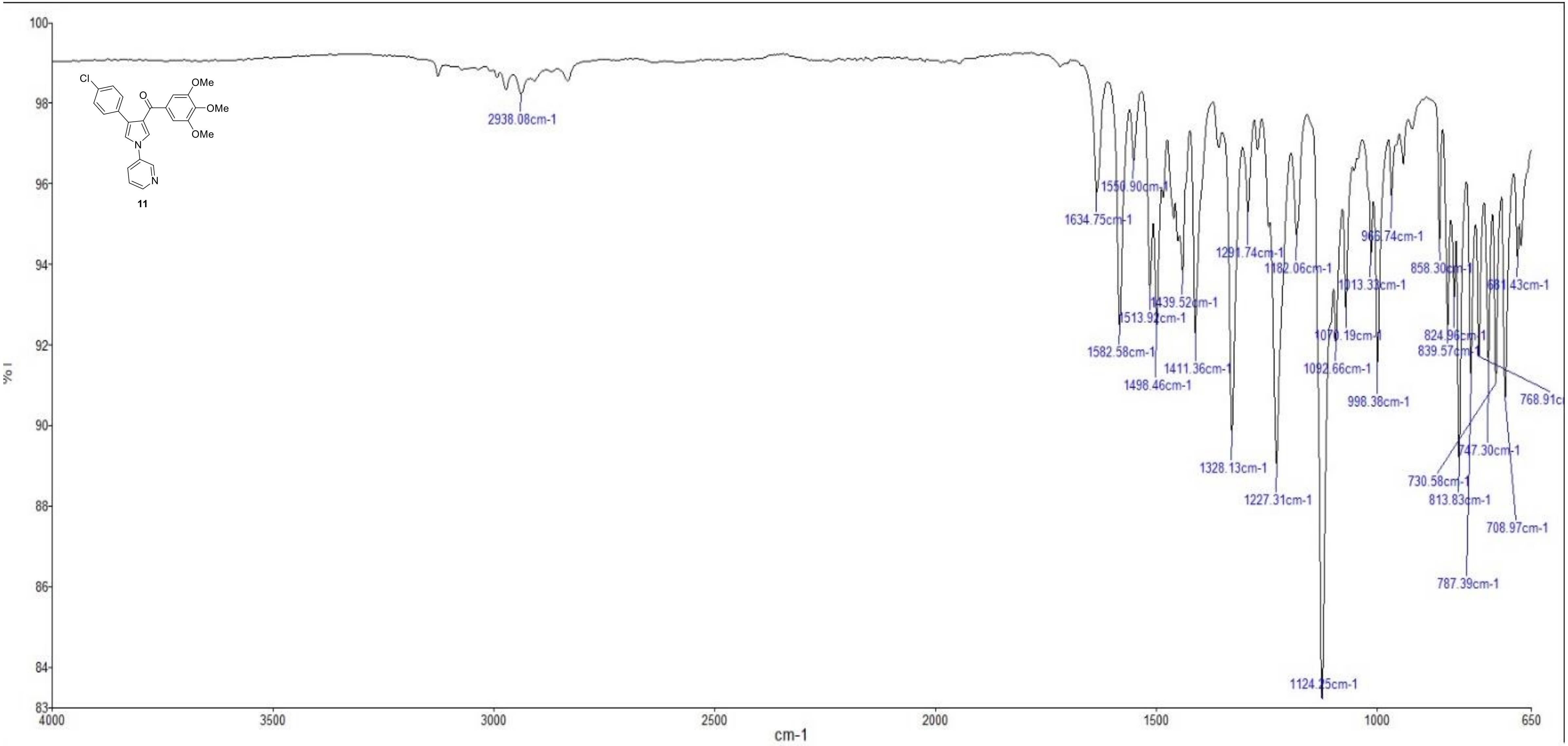

Cl
O
OMe
OMe
OMe
N
N
11
%T
cm-1
2938.08cm-1
1634.75cm-1
1550.90cm-1
1582.58cm-1
1513.92cm-1
1498.46cm-1
1439.52cm-1
1411.36cm-1
1328.13cm-1
1291.74cm-1
1227.31cm-1
1182.06cm-1
1124.25cm-1
1092.66cm-1
1070.19cm-1
1013.33cm-1
998.38cm-1
966.74cm-1
858.30cm-1
839.57cm-1
824.96cm-1
813.83cm-1
787.39cm-1
768.91c
747.30cm-1
730.58cm-1
708.97cm-1
681.43cm-1

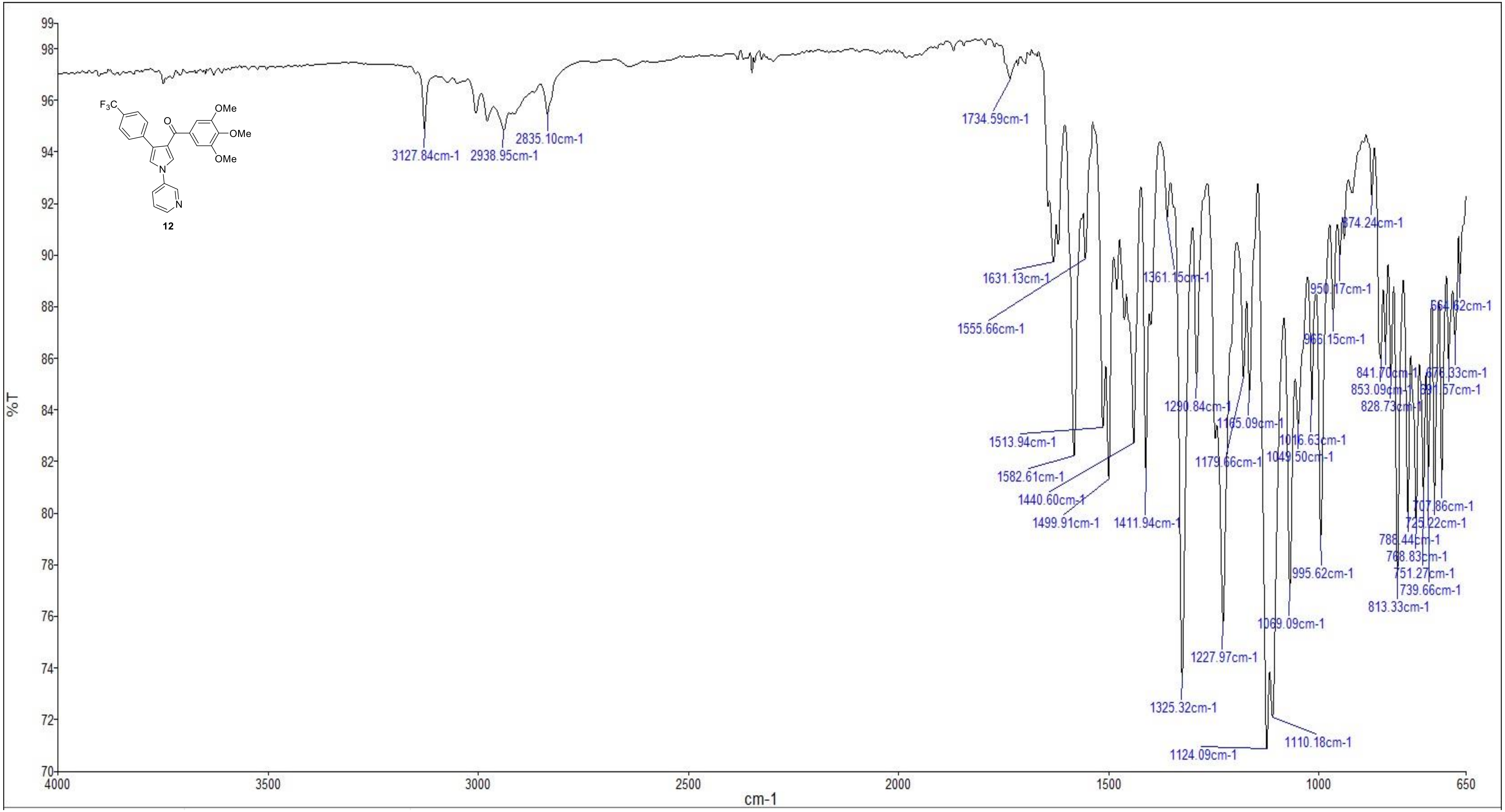
12
%T
cm-1
3127.84cm-1
2938.95cm-1
2835.10cm-1
1734.59cm-1
1631.13cm-1
1555.66cm-1
1513.94cm-1
1582.61cm-1
1440.60cm-1
1499.91cm-1
1411.94cm-1
1361.15cm-1
1290.84cm-1
1165.09cm-1
1179.66cm-1
1325.32cm-1
1227.97cm-1
1124.09cm-1
1110.18cm-1
1069.09cm-1
1049.50cm-1
1016.63cm-1
995.62cm-1
966.15cm-1
950.17cm-1
874.24cm-1
841.70cm-1
853.09cm-1
828.73cm-1
813.33cm-1
788.44cm-1
768.83cm-1
751.27cm-1
739.66cm-1
725.22cm-1
707.86cm-1
691.57cm-1
676.33cm-1
664.62cm-1

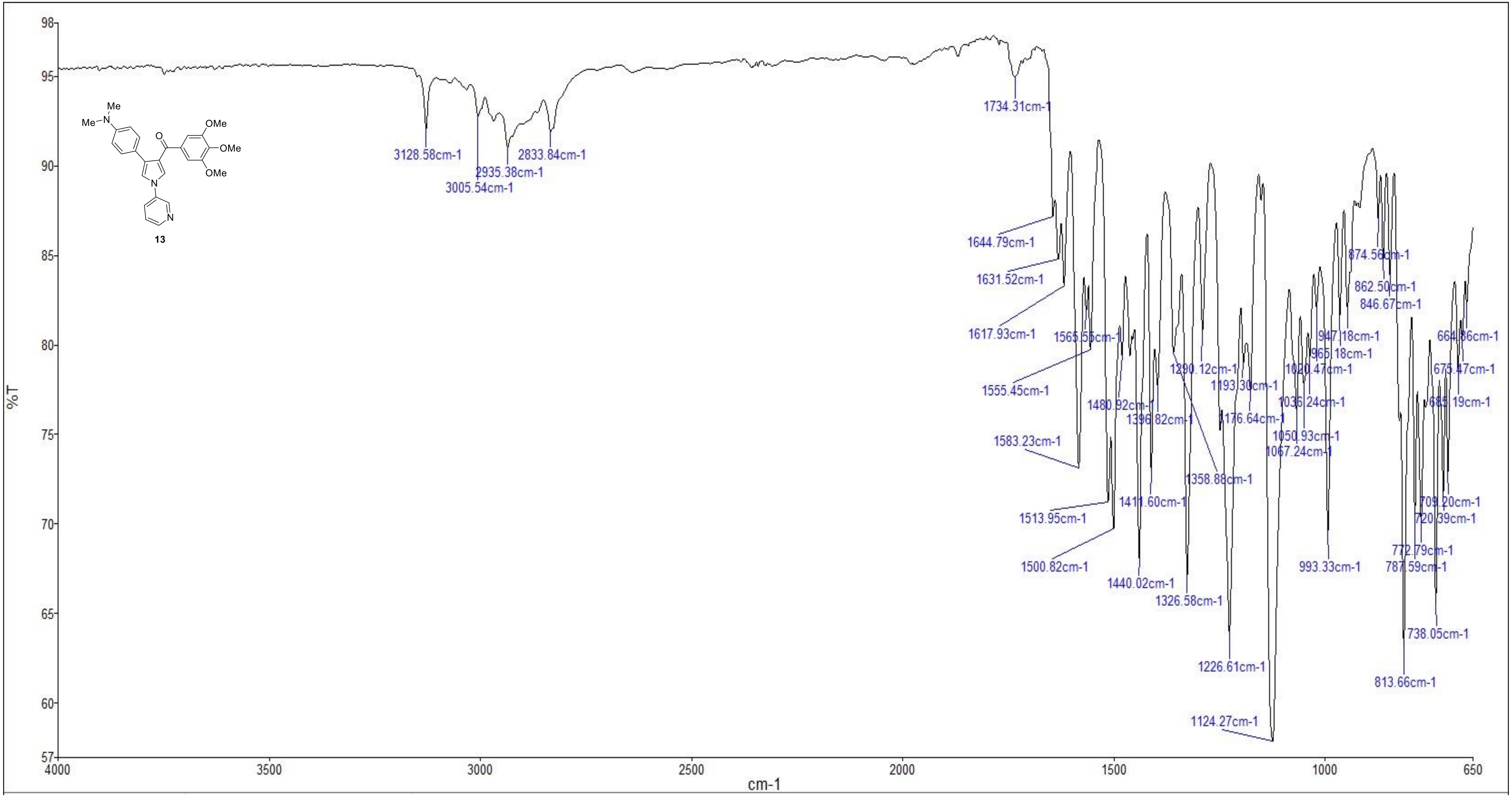

Me
Me
N
O
OMe
OMe
OMe
N
N
13
3128.58cm-1
3005.54cm-1
2935.38cm-1
2833.84cm-1
1734.31cm-1
1644.79cm-1
1631.52cm-1
1617.93cm-1
1565.55cm-1
1555.45cm-1
1583.23cm-1
1513.95cm-1
1500.82cm-1
1480.92cm-1
1440.02cm-1
1411.60cm-1
1396.82cm-1
1358.88cm-1
1326.58cm-1
1290.12cm-1
1226.61cm-1
1193.30cm-1
1176.64cm-1
1124.27cm-1
1067.24cm-1
1050.93cm-1
1036.24cm-1
1020.47cm-1
993.33cm-1
965.18cm-1
947.18cm-1
874.56cm-1
862.50cm-1
846.67cm-1
813.66cm-1
787.59cm-1
772.79cm-1
738.05cm-1
720.39cm-1
709.20cm-1
685.19cm-1
675.47cm-1
664.86cm-1
%T
cm-1

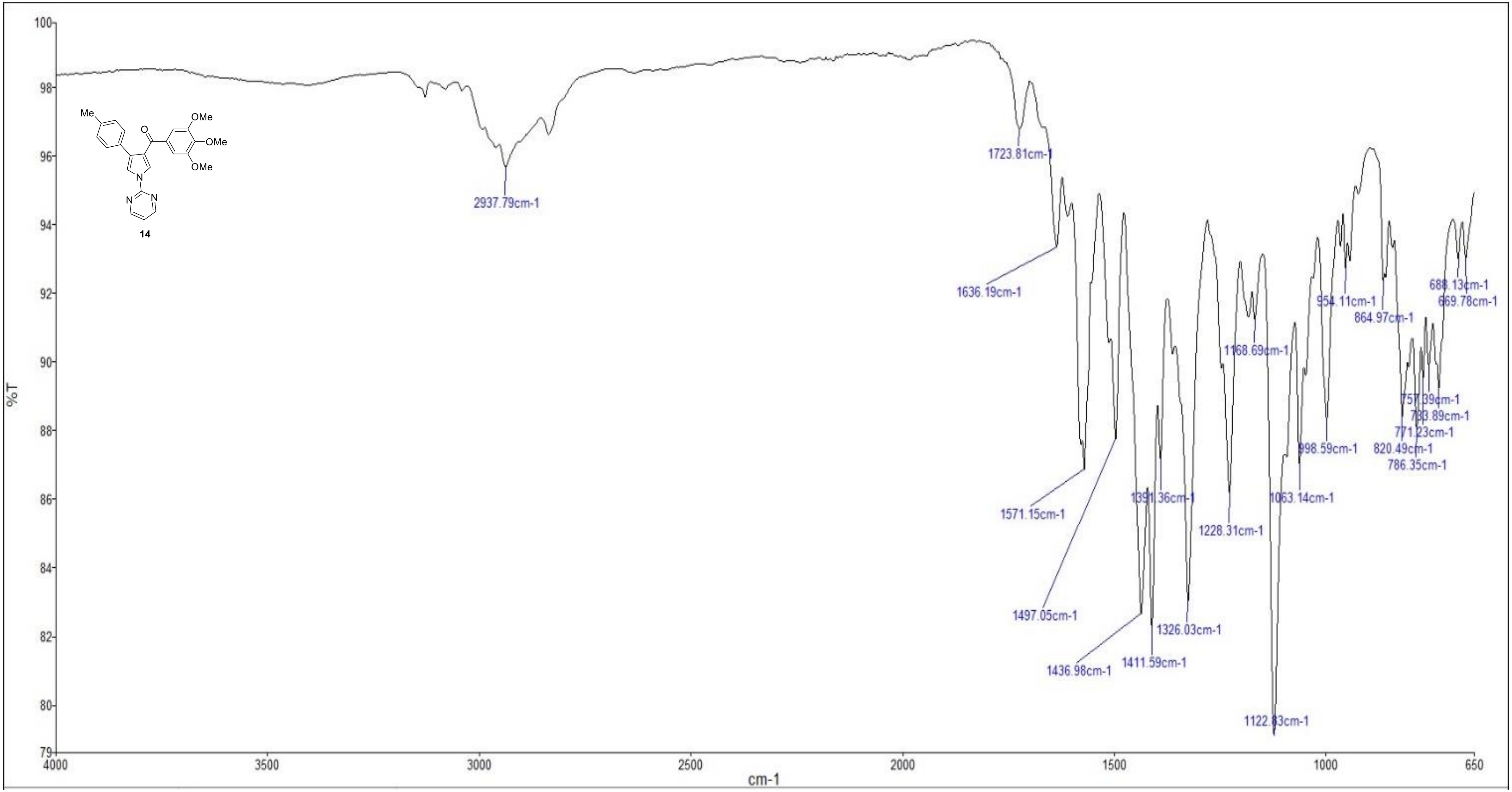
Me
OMe
O
OMe
OMe
N
N
N
14
2937.79cm-1
1723.81cm-1
1636.19cm-1
1571.15cm-1
1497.05cm-1
1436.98cm-1
1411.59cm-1
1391.36cm-1
1326.03cm-1
1228.31cm-1
1168.69cm-1
1122.83cm-1
1063.14cm-1
998.59cm-1
954.11cm-1
864.97cm-1
820.49cm-1
786.35cm-1
771.23cm-1
757.39cm-1
733.89cm-1
688.13cm-1
669.78cm-1
%T
cm-1
100
98
96
94
92
90
88
86
84
82
80
79
4000
3500
3000
2500
2000
1500
1000
650

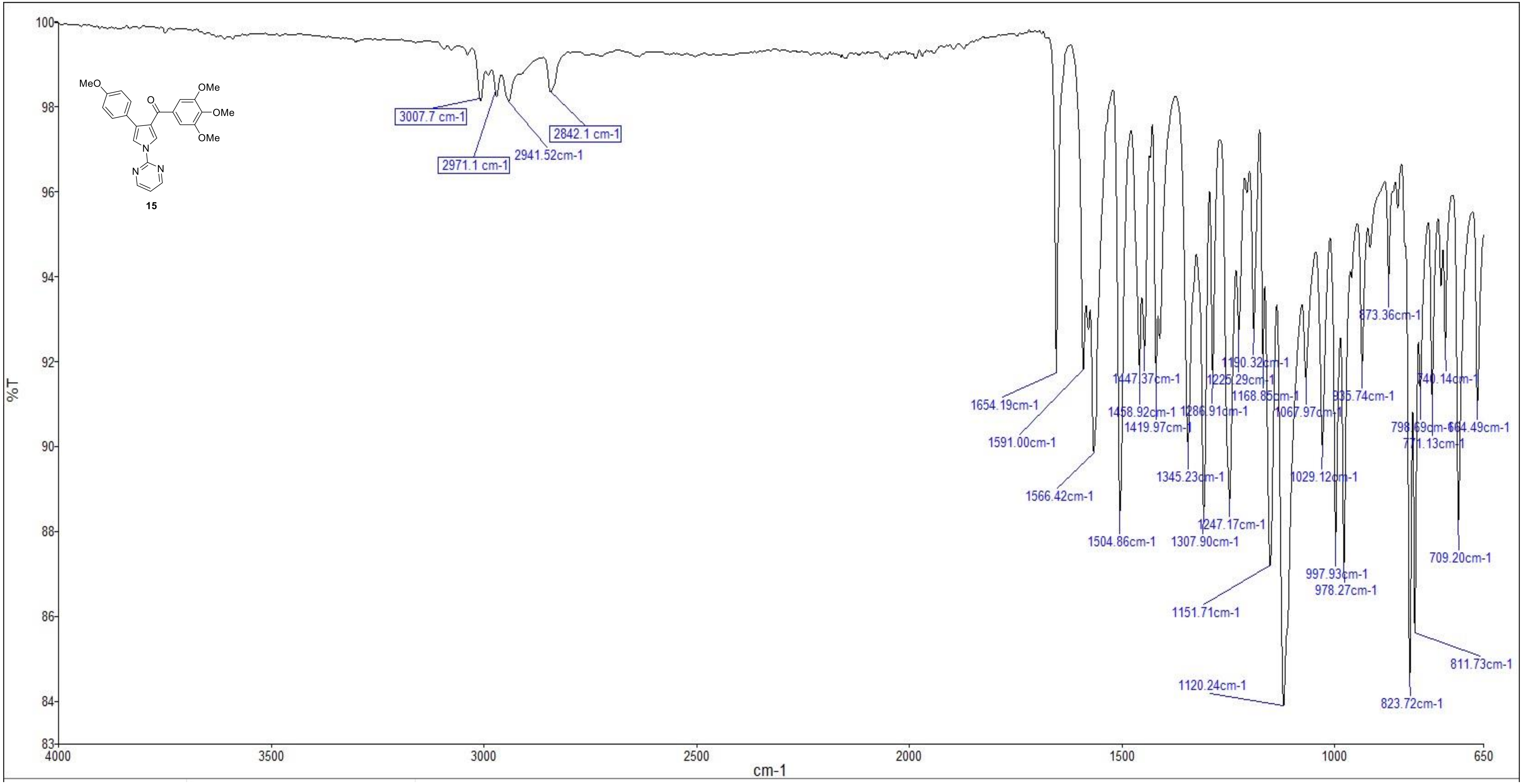

100
98
96
94
92
90
88
86
84
83
%T
4000
3500
3000
2500
2000
1500
1000
650
cm-1
MeO
OMe
O
OMe
OMe
N
N
N
15
3007.7 cm-1
2971.1 cm-1
2941.52cm-1
2842.1 cm-1
1654.19cm-1
1591.00cm-1
1566.42cm-1
1504.86cm-1
1458.92cm-1
1447.37cm-1
1419.97cm-1
1345.23cm-1
1307.90cm-1
1286.91cm-1
1247.17cm-1
1225.29cm-1
1190.32cm-1
1168.85cm-1
1151.71cm-1
1120.24cm-1
1067.97cm-1
1029.12cm-1
997.93cm-1
978.27cm-1
935.74cm-1
873.36cm-1
823.72cm-1
811.73cm-1
771.13cm-1
740.14cm-1
709.20cm-1
664.49cm-1

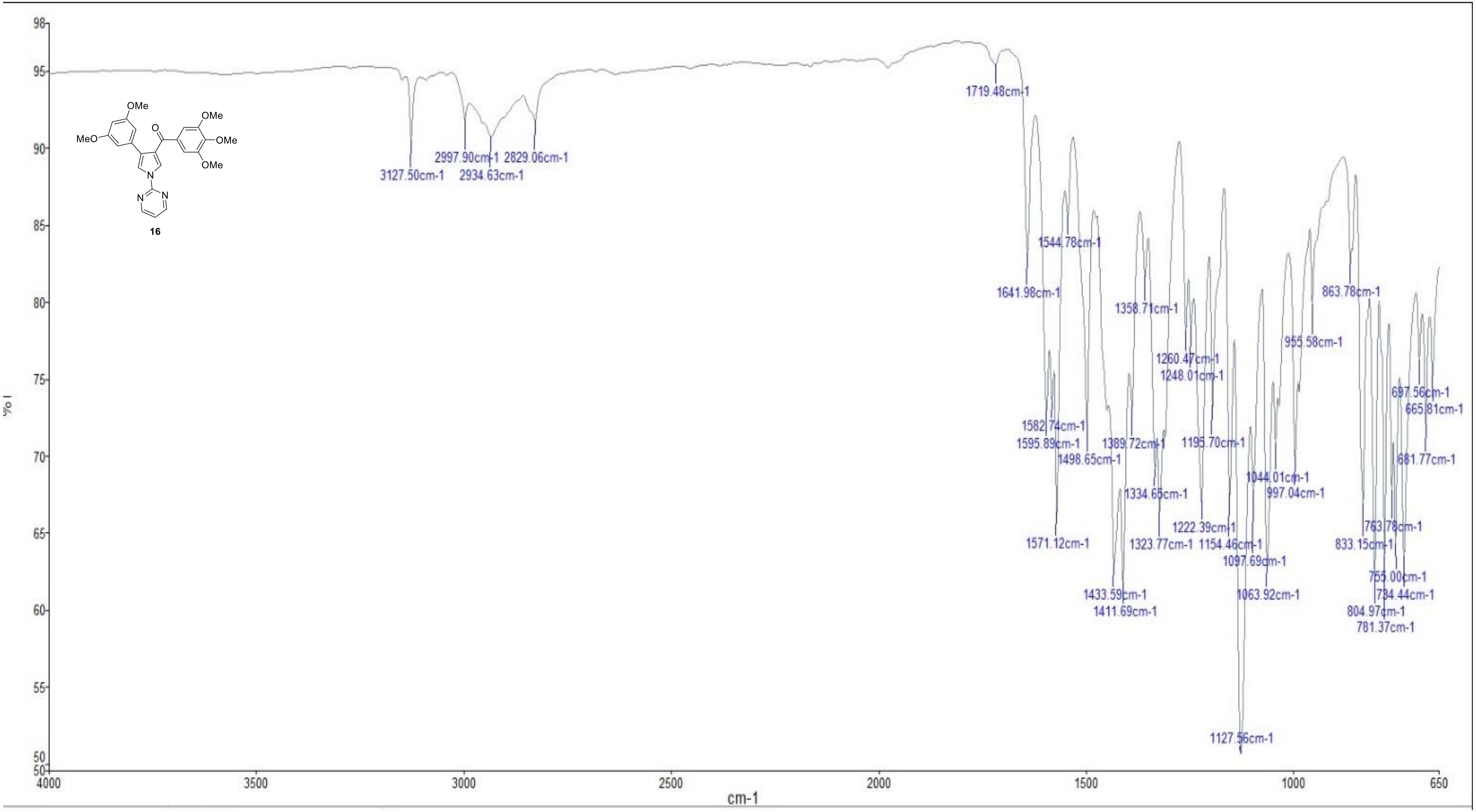

OMe
MeO
O
OMe
OMe
OMe
N
N
N
16
3127.50cm-1
2997.90cm-1
2934.63cm-1
2829.06cm-1
1719.48cm-1
1641.98cm-1
1544.78cm-1
1582.74cm-1
1595.89cm-1
1571.12cm-1
1498.65cm-1
1433.59cm-1
1411.69cm-1
1389.72cm-1
1358.71cm-1
1334.65cm-1
1323.77cm-1
1260.47cm-1
1248.01cm-1
1222.39cm-1
1195.70cm-1
1154.46cm-1
1127.56cm-1
1097.69cm-1
1063.92cm-1
1044.01cm-1
997.04cm-1
955.58cm-1
863.78cm-1
833.15cm-1
804.97cm-1
781.37cm-1
763.78cm-1
755.00cm-1
734.44cm-1
697.56cm-1
681.77cm-1
665.81cm-1
%T
cm-1

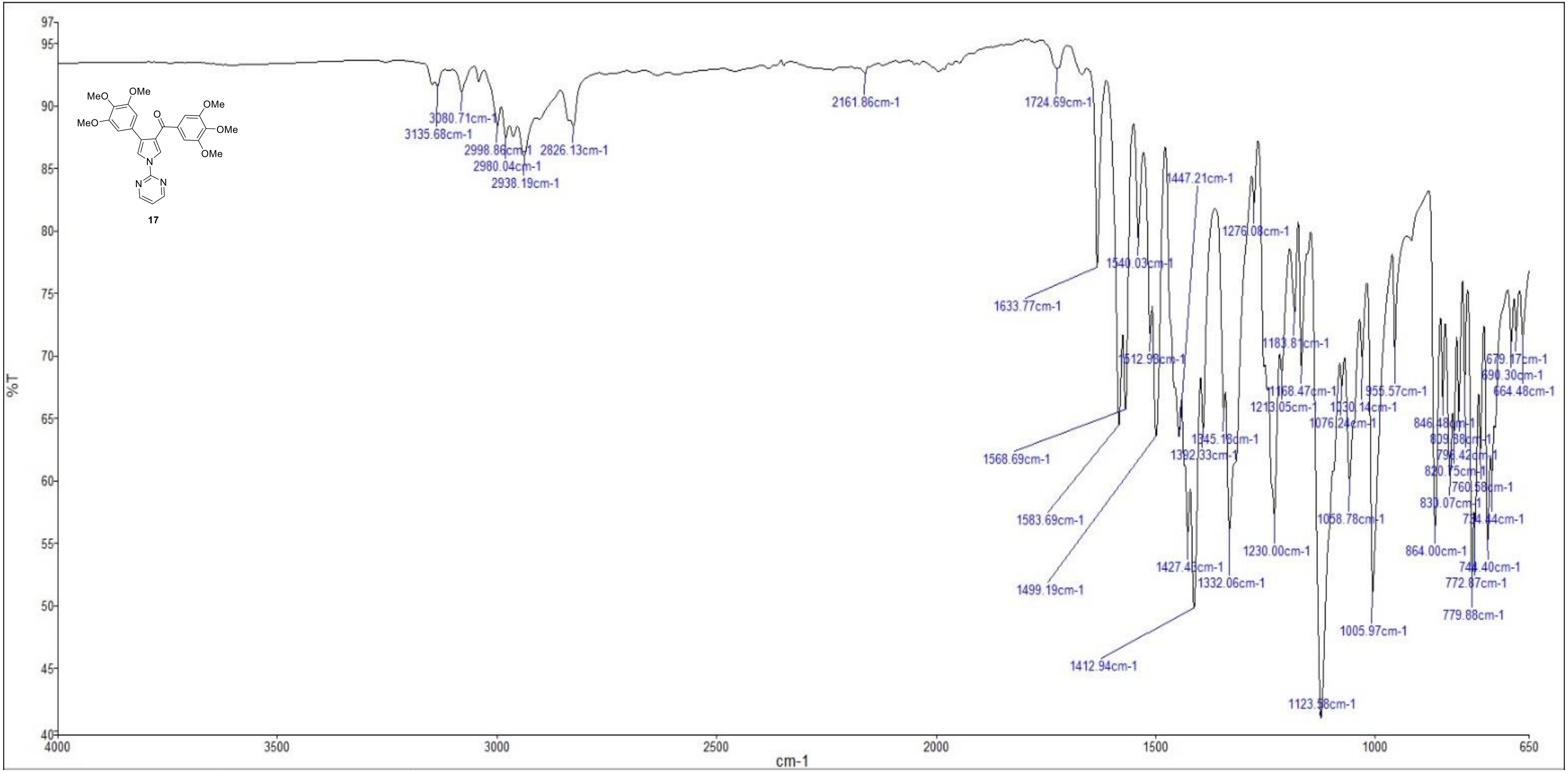
MeO
OMe
MeO
O
OMe
OMe
OMe
N
N
N
17
%T
cm-1
97
95
90
85
80
75
70
65
60
55
50
45
40
4000
3500
3000
2500
2000
1500
1000
650
3135.68cm-1
3080.71cm-1
2998.86cm-1
2980.04cm-1
2938.19cm-1
2826.13cm-1
2161.86cm-1
1724.69cm-1
1633.77cm-1
1568.69cm-1
1583.69cm-1
1540.03cm-1
1512.98cm-1
1499.19cm-1
1447.21cm-1
1427.43cm-1
1412.94cm-1
1392.33cm-1
1345.18cm-1
1332.06cm-1
1276.08cm-1
1230.00cm-1
1213.05cm-1
1183.81cm-1
1168.47cm-1
1123.58cm-1
1076.24cm-1
1058.78cm-1
1030.14cm-1
1005.97cm-1
955.57cm-1
864.00cm-1
846.48cm-1
830.07cm-1
820.75cm-1
809.88cm-1
796.42cm-1
779.88cm-1
772.87cm-1
760.58cm-1
754.44cm-1
744.40cm-1
690.30cm-1
679.17cm-1
664.48cm-1

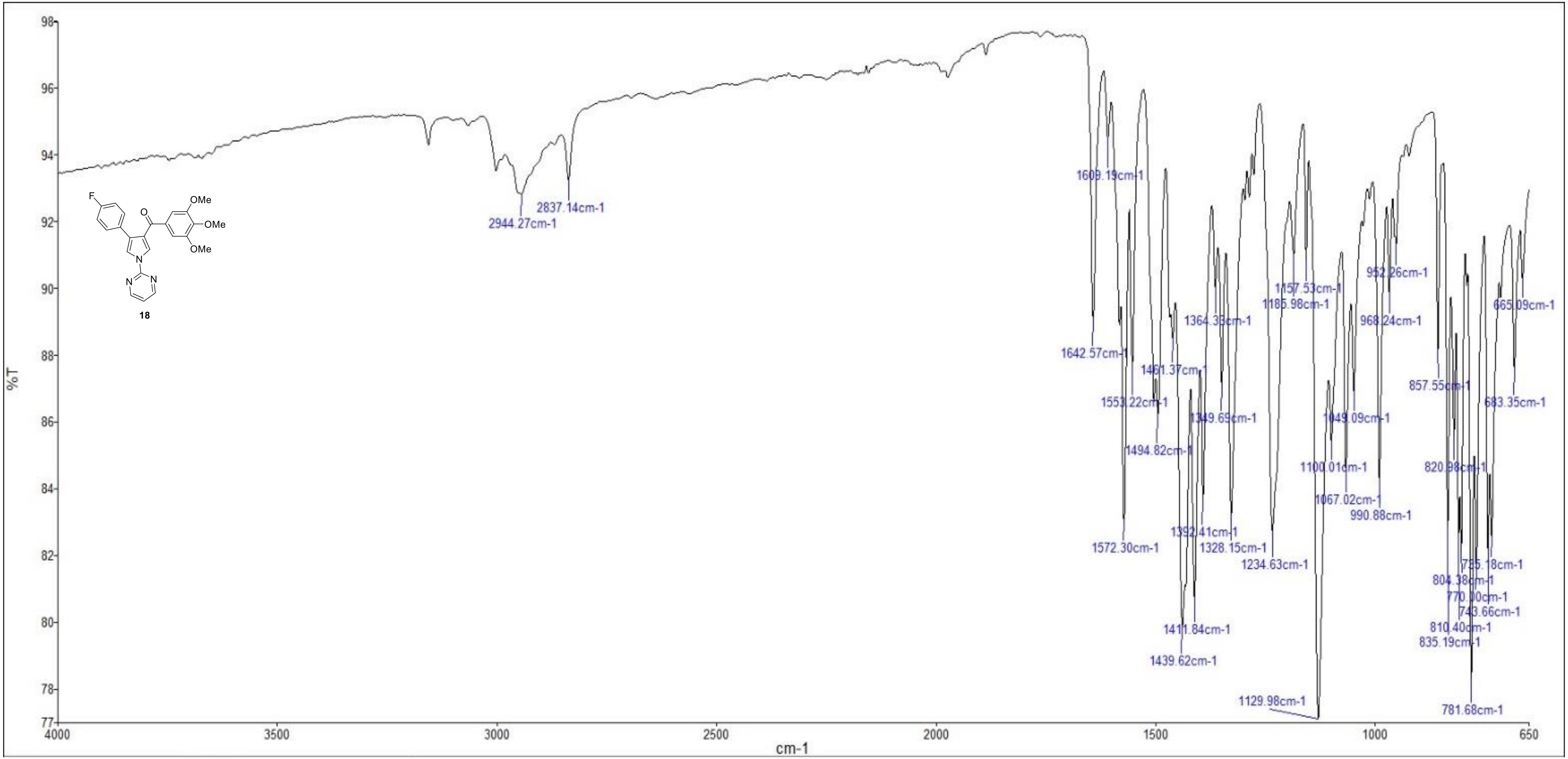
%T
cm-1
18
OMe
OMe
OMe
F
N
2944.27cm-1
2837.14cm-1
1609.19cm-1
1642.57cm-1
1572.30cm-1
1553.22cm-1
1494.82cm-1
1461.37cm-1
1439.62cm-1
1411.84cm-1
1392.41cm-1
1364.33cm-1
1349.69cm-1
1328.15cm-1
1234.63cm-1
1185.98cm-1
1157.53cm-1
1129.98cm-1
1100.01cm-1
1067.02cm-1
1049.09cm-1
990.88cm-1
968.24cm-1
952.26cm-1
857.55cm-1
835.19cm-1
820.98cm-1
810.40cm-1
804.38cm-1
781.68cm-1
770.00cm-1
743.66cm-1
735.18cm-1
683.35cm-1
665.09cm-1

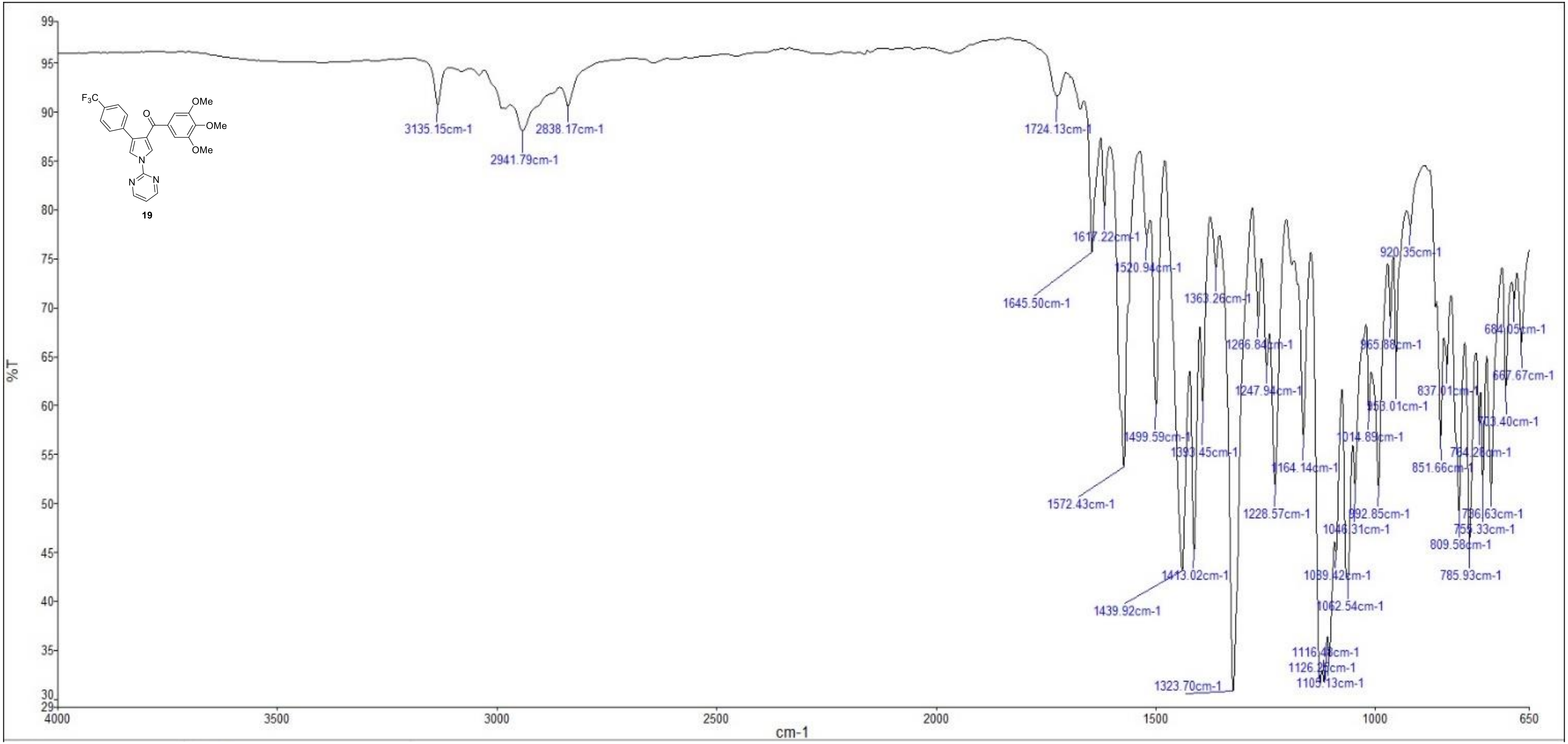

19
%T
cm-1
3135.15cm-1
2941.79cm-1
2838.17cm-1
1724.13cm-1
1617.22cm-1
1520.94cm-1
1645.50cm-1
1363.26cm-1
1266.84cm-1
1247.94cm-1
1499.59cm-1
1393.45cm-1
1164.14cm-1
1572.43cm-1
1228.57cm-1
1413.02cm-1
1439.92cm-1
1323.70cm-1
920.35cm-1
965.88cm-1
953.01cm-1
1014.89cm-1
992.85cm-1
1046.31cm-1
1089.42cm-1
1062.54cm-1
1116.48cm-1
1126.26cm-1
1105.13cm-1
684.05cm-1
667.67cm-1
837.01cm-1
703.40cm-1
764.26cm-1
851.66cm-1
736.63cm-1
755.33cm-1
809.58cm-1
785.93cm-1

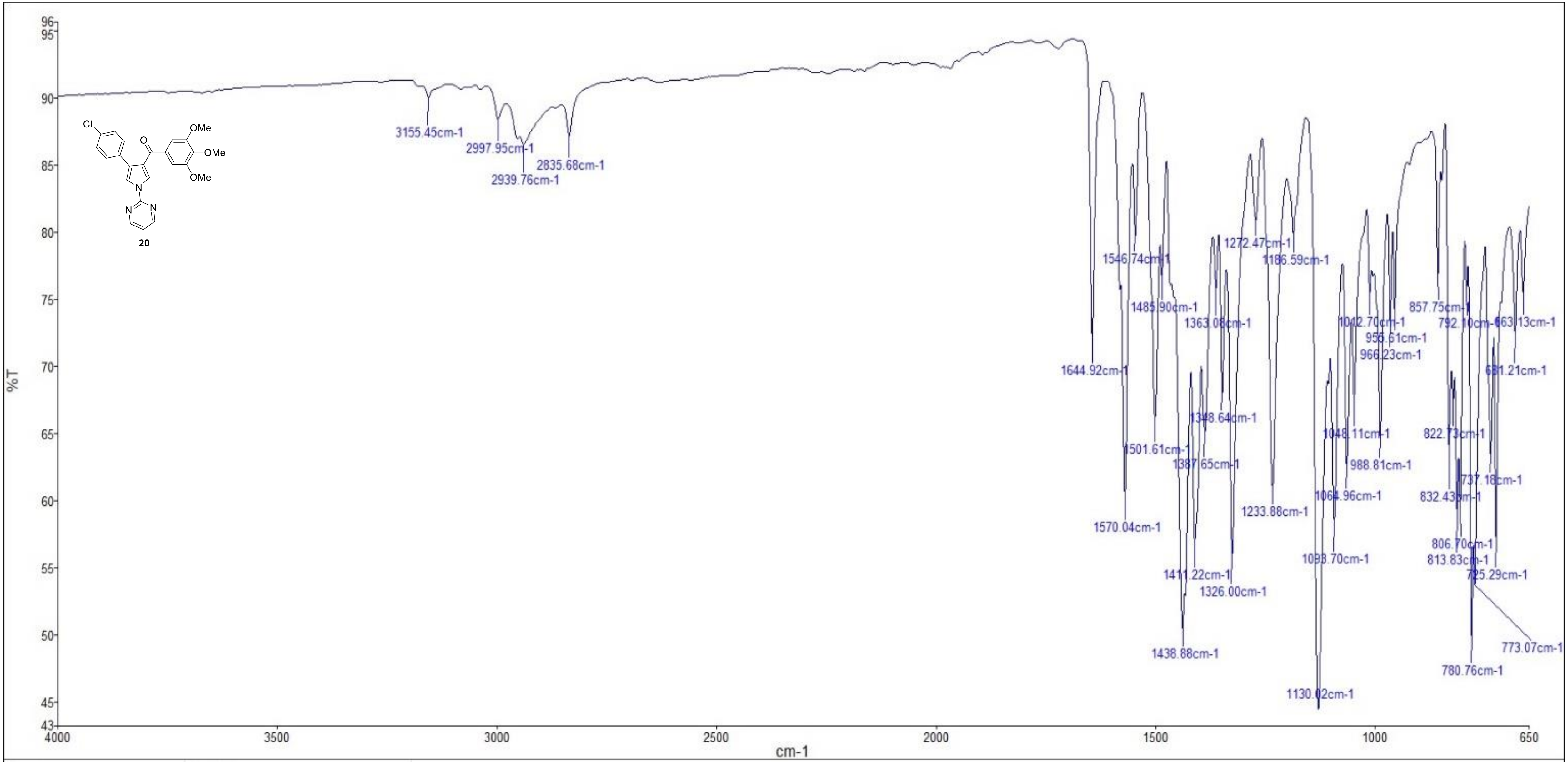

%T
cm-1
20
Cl
O
OMe
OMe
OMe
N
N
N
3155.45cm-1
2997.95cm-1
2939.76cm-1
2835.68cm-1
1644.92cm-1
1570.04cm-1
1546.74cm-1
1501.61cm-1
1485.90cm-1
1438.88cm-1
1411.22cm-1
1387.65cm-1
1363.08cm-1
1348.64cm-1
1326.00cm-1
1272.47cm-1
1233.88cm-1
1186.59cm-1
1130.02cm-1
1098.70cm-1
1064.96cm-1
1048.11cm-1
988.81cm-1
966.23cm-1
955.61cm-1
857.75cm-1
832.43cm-1
822.73cm-1
813.83cm-1
806.70cm-1
780.76cm-1
773.07cm-1
737.18cm-1
725.29cm-1
663.13cm-1

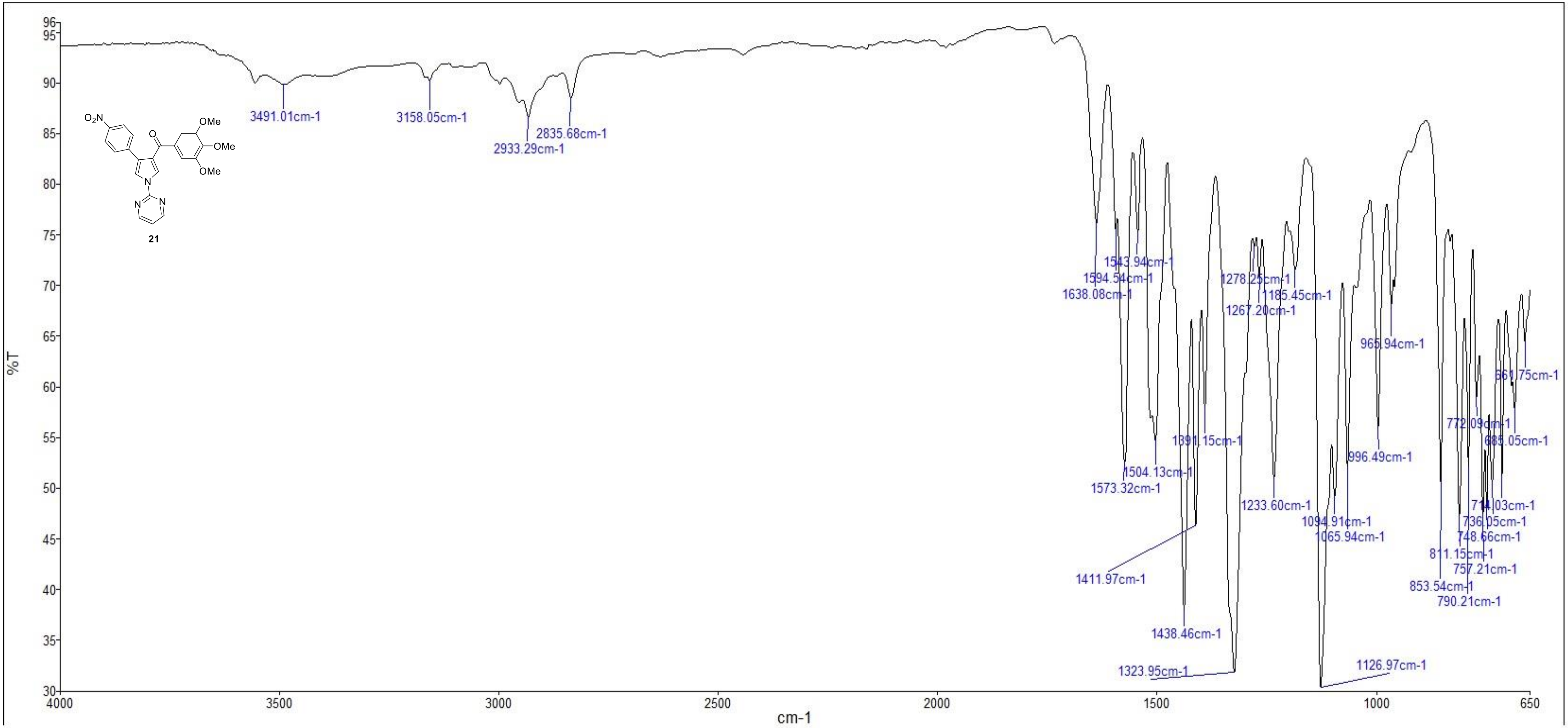

%T
cm-1
21
3491.01cm-1
3158.05cm-1
2933.29cm-1
2835.68cm-1
1638.08cm-1
1594.54cm-1
1543.94cm-1
1573.32cm-1
1504.13cm-1
1438.46cm-1
1411.97cm-1
1391.15cm-1
1323.95cm-1
1278.25cm-1
1267.20cm-1
1233.60cm-1
1185.45cm-1
1126.97cm-1
1094.91cm-1
1065.94cm-1
996.49cm-1
965.94cm-1
853.54cm-1
811.15cm-1
790.21cm-1
772.09cm-1
757.21cm-1
748.66cm-1
736.05cm-1
714.03cm-1
685.05cm-1
661.75cm-1

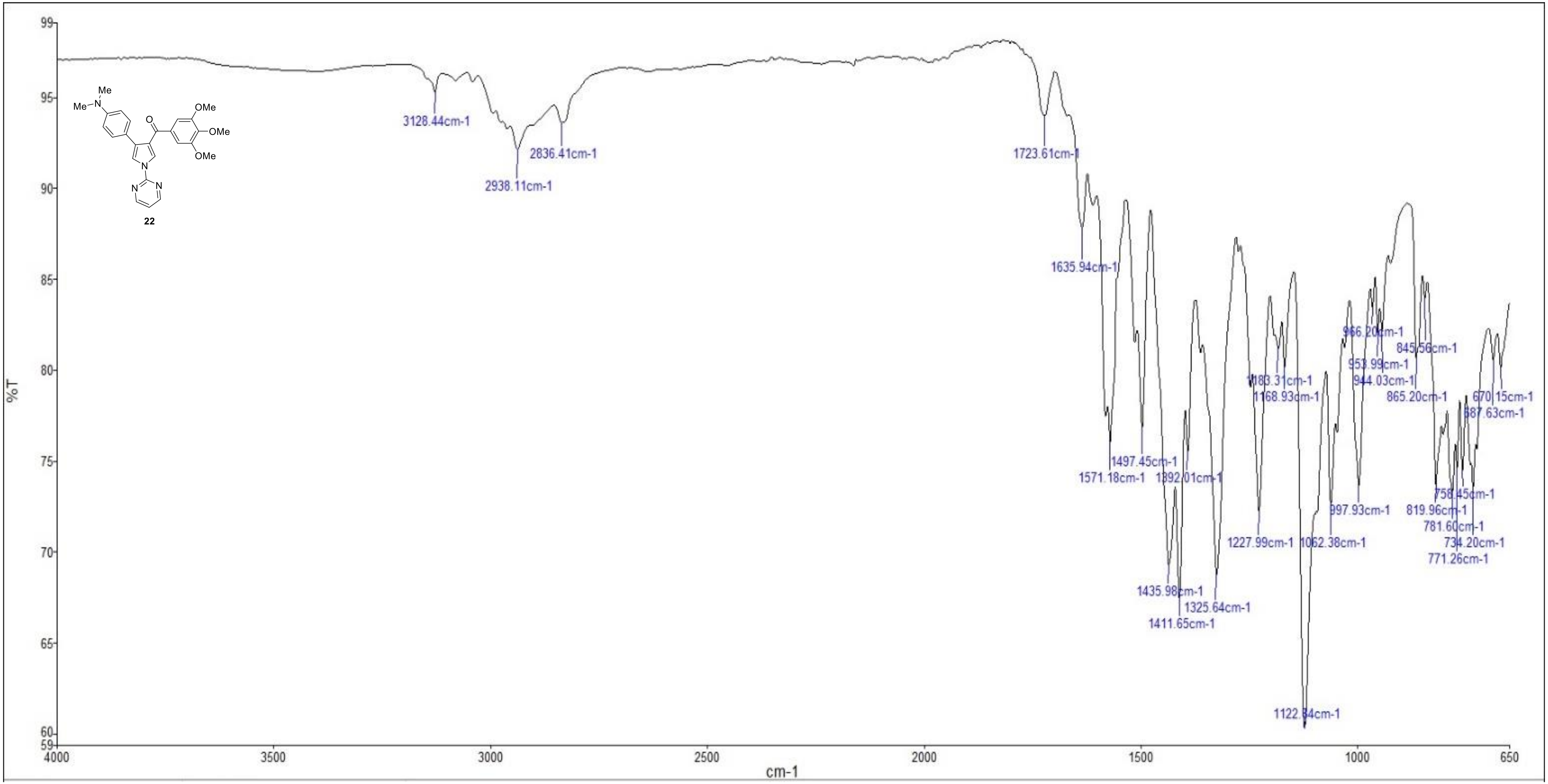

Me
Me–N
O
OMe
OMe
OMe
N
N
N
22
%T
cm-1
3128.44cm-1
2938.11cm-1
2836.41cm-1
1723.61cm-1
1635.94cm-1
1571.18cm-1
1497.45cm-1
1435.98cm-1
1411.65cm-1
1325.64cm-1
1227.99cm-1
1168.93cm-1
1122.84cm-1
1062.38cm-1
997.93cm-1
944.03cm-1
865.20cm-1
819.96cm-1
781.60cm-1
771.26cm-1
758.45cm-1
734.20cm-1
670.15cm-1

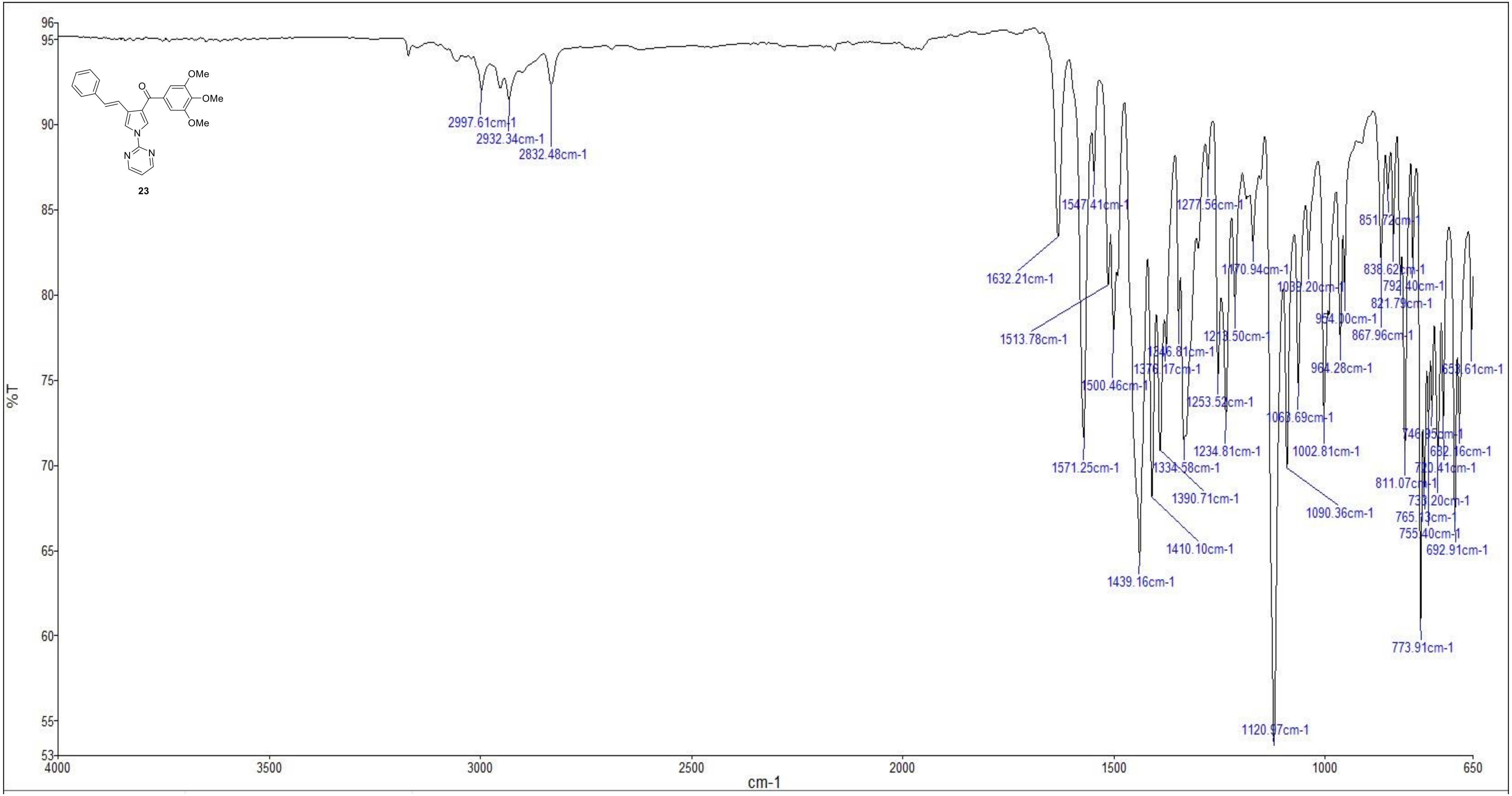
OMe
OMe
OMe
O
N
N
N
23
%T
cm-1
2997.61cm-1
2932.34cm-1
2832.48cm-1
1632.21cm-1
1547.41cm-1
1513.78cm-1
1500.46cm-1
1571.25cm-1
1439.16cm-1
1410.10cm-1
1390.71cm-1
1376.17cm-1
1346.81cm-1
1334.58cm-1
1277.56cm-1
1253.52cm-1
1234.81cm-1
1213.50cm-1
1170.94cm-1
1120.97cm-1
1090.36cm-1
1063.69cm-1
1039.20cm-1
1002.81cm-1
964.28cm-1
954.00cm-1
867.96cm-1
851.72cm-1
838.62cm-1
821.79cm-1
811.07cm-1
792.40cm-1
773.91cm-1
765.13cm-1
755.40cm-1
746.95cm-1
733.20cm-1
720.41cm-1
692.91cm-1
682.16cm-1
653.61cm-1

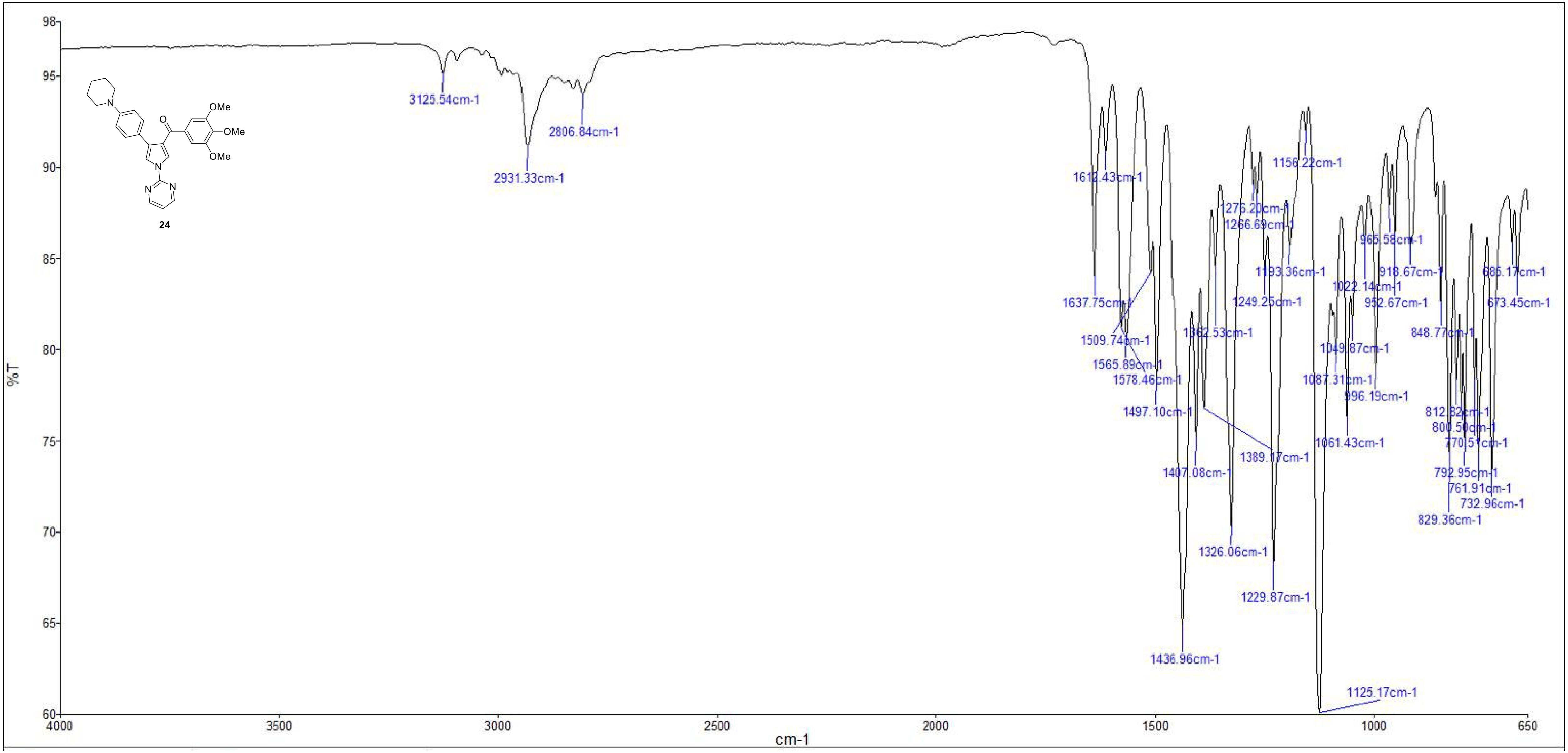
%T
cm-1
98
95
90
85
80
75
70
65
60
4000
3500
3000
2500
2000
1500
1000
650
OMe
OMe
OMe
O
N
N
N
N
24
3125.54cm-1
2931.33cm-1
2806.84cm-1
1612.43cm-1
1637.75cm-1
1509.74cm-1
1565.89cm-1
1578.46cm-1
1497.10cm-1
1436.96cm-1
1407.08cm-1
1362.53cm-1
1389.17cm-1
1326.06cm-1
1276.20cm-1
1266.69cm-1
1249.25cm-1
1229.87cm-1
1193.36cm-1
1156.22cm-1
1125.17cm-1
1087.31cm-1
1061.43cm-1
1049.87cm-1
1022.14cm-1
996.19cm-1
965.58cm-1
952.67cm-1
918.67cm-1
848.77cm-1
829.36cm-1
812.82cm-1
800.50cm-1
792.95cm-1
770.51cm-1
761.91cm-1
732.96cm-1
686.17cm-1
673.45cm-1

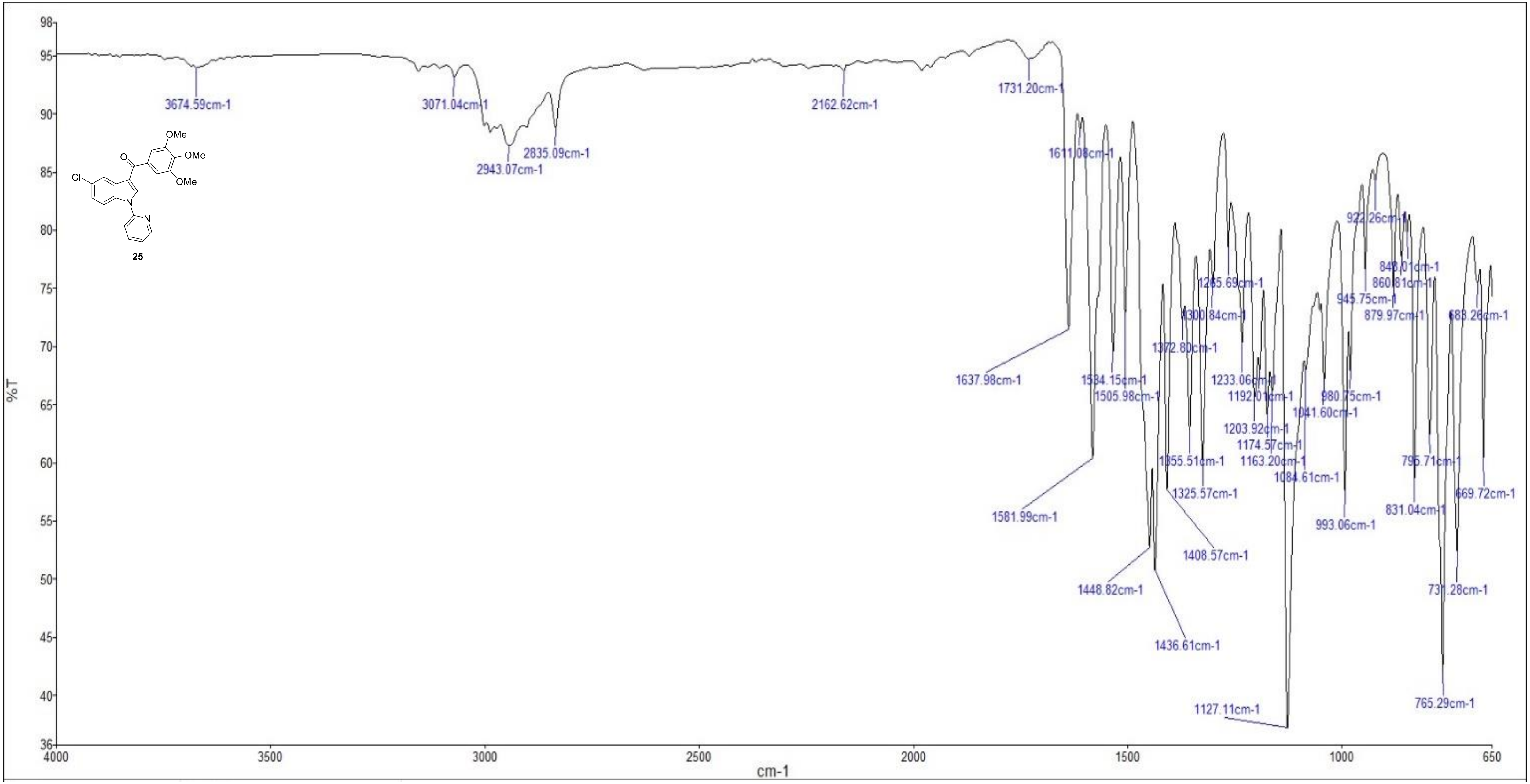

3674.59cm-1
3071.04cm-1
2943.07cm-1
2835.09cm-1
2162.62cm-1
1731.20cm-1
1611.08cm-1
1637.98cm-1
1581.99cm-1
1534.15cm-1
1505.98cm-1
1448.82cm-1
1436.61cm-1
1408.57cm-1
1372.80cm-1
1355.51cm-1
1325.57cm-1
1300.84cm-1
1265.69cm-1
1233.06cm-1
1203.92cm-1
1192.01cm-1
1174.57cm-1
1163.20cm-1
1127.11cm-1
1084.61cm-1
1041.60cm-1
993.06cm-1
980.75cm-1
945.75cm-1
922.26cm-1
879.97cm-1
860.81cm-1
848.01cm-1
831.04cm-1
796.71cm-1
765.29cm-1
731.28cm-1
683.26cm-1
669.72cm-1
%T
cm-1
OMe
OMe
OMe
O
Cl
N
N
25

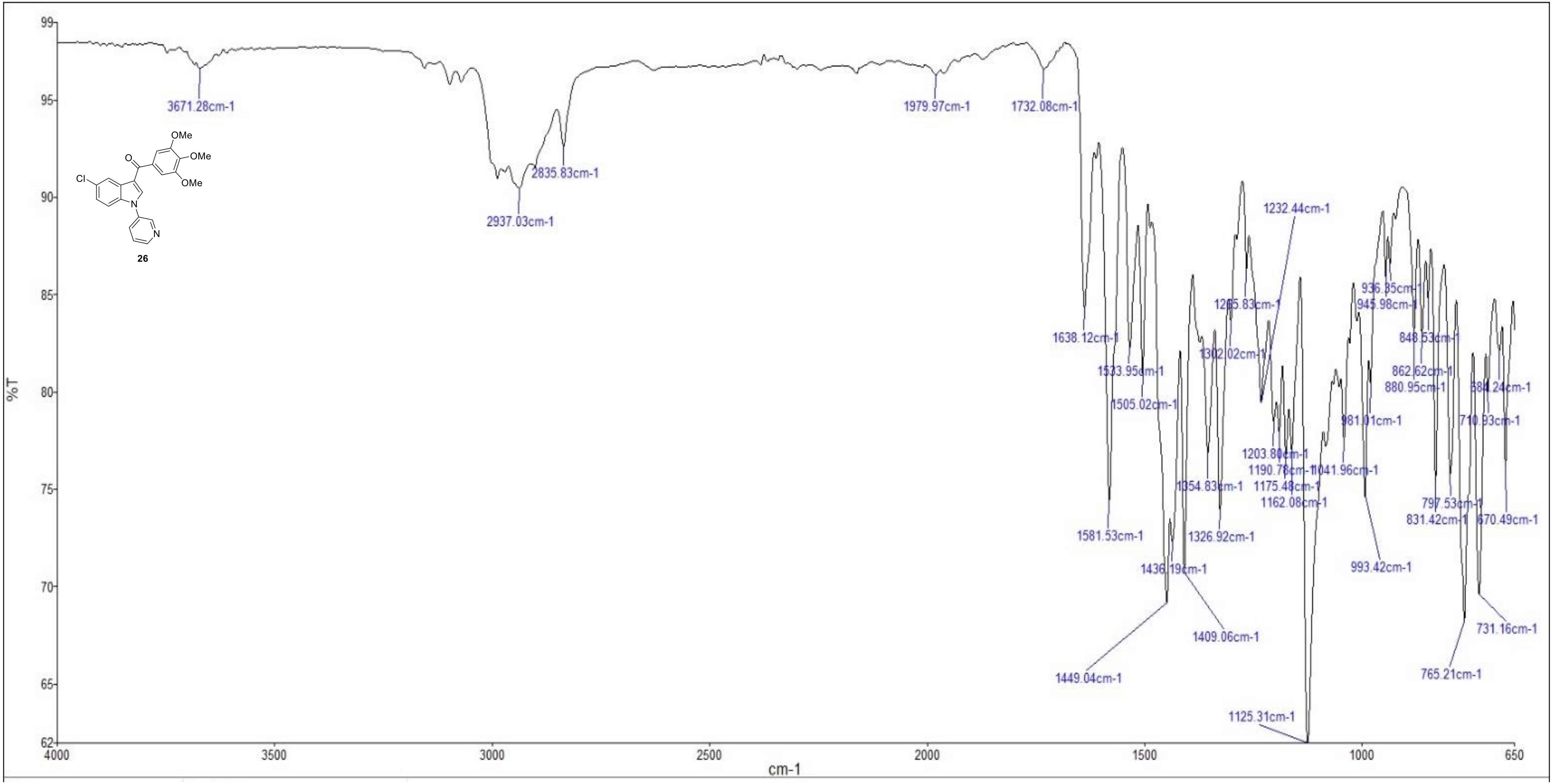
OMe
OMe
OMe
Cl
O
N
N
26
%T
cm-1
3671.28cm-1
2937.03cm-1
2835.83cm-1
1979.97cm-1
1732.08cm-1
1638.12cm-1
1581.53cm-1
1533.95cm-1
1505.02cm-1
1449.04cm-1
1436.19cm-1
1409.06cm-1
1354.83cm-1
1326.92cm-1
1302.02cm-1
1265.83cm-1
1232.44cm-1
1203.80cm-1
1190.78cm-1
1175.48cm-1
1162.08cm-1
1125.31cm-1
1041.96cm-1
993.42cm-1
981.01cm-1
945.98cm-1
936.85cm-1
880.95cm-1
862.62cm-1
848.53cm-1
831.42cm-1
797.53cm-1
765.21cm-1
731.16cm-1
710.93cm-1
684.24cm-1
670.49cm-1

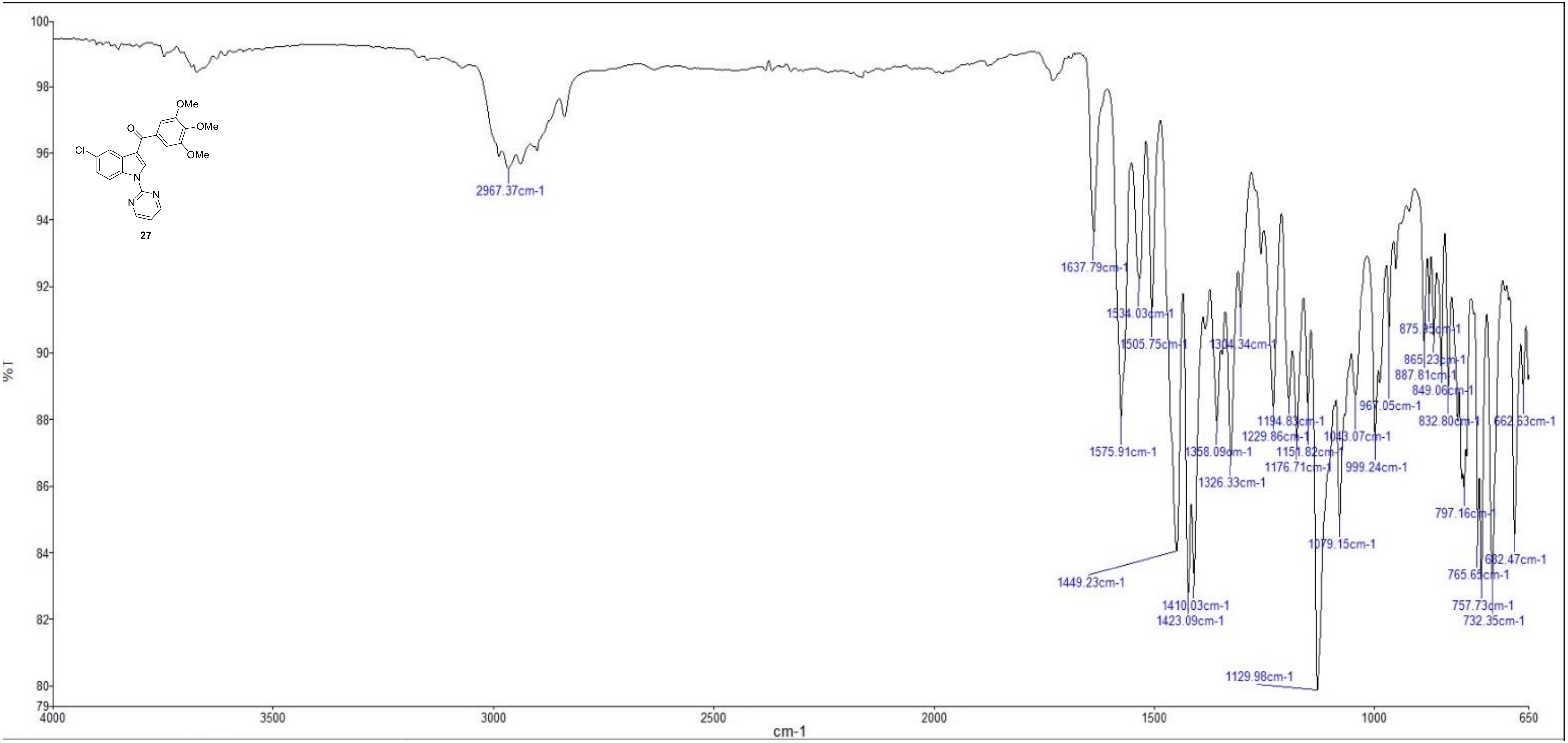
OMe
OMe
OMe
O
Cl
N
N
N
27
%T
cm-1
2967.37cm-1
1637.79cm-1
1534.03cm-1
1505.75cm-1
1575.91cm-1
1449.23cm-1
1410.03cm-1
1423.09cm-1
1358.09cm-1
1326.33cm-1
1304.34cm-1
1194.83cm-1
1229.86cm-1
1151.82cm-1
1176.71cm-1
1129.98cm-1
1079.15cm-1
1043.07cm-1
999.24cm-1
967.05cm-1
875.95cm-1
865.23cm-1
887.81cm-1
849.06cm-1
832.80cm-1
797.16cm-1
765.65cm-1
757.73cm-1
732.35cm-1
682.47cm-1
662.63cm-1

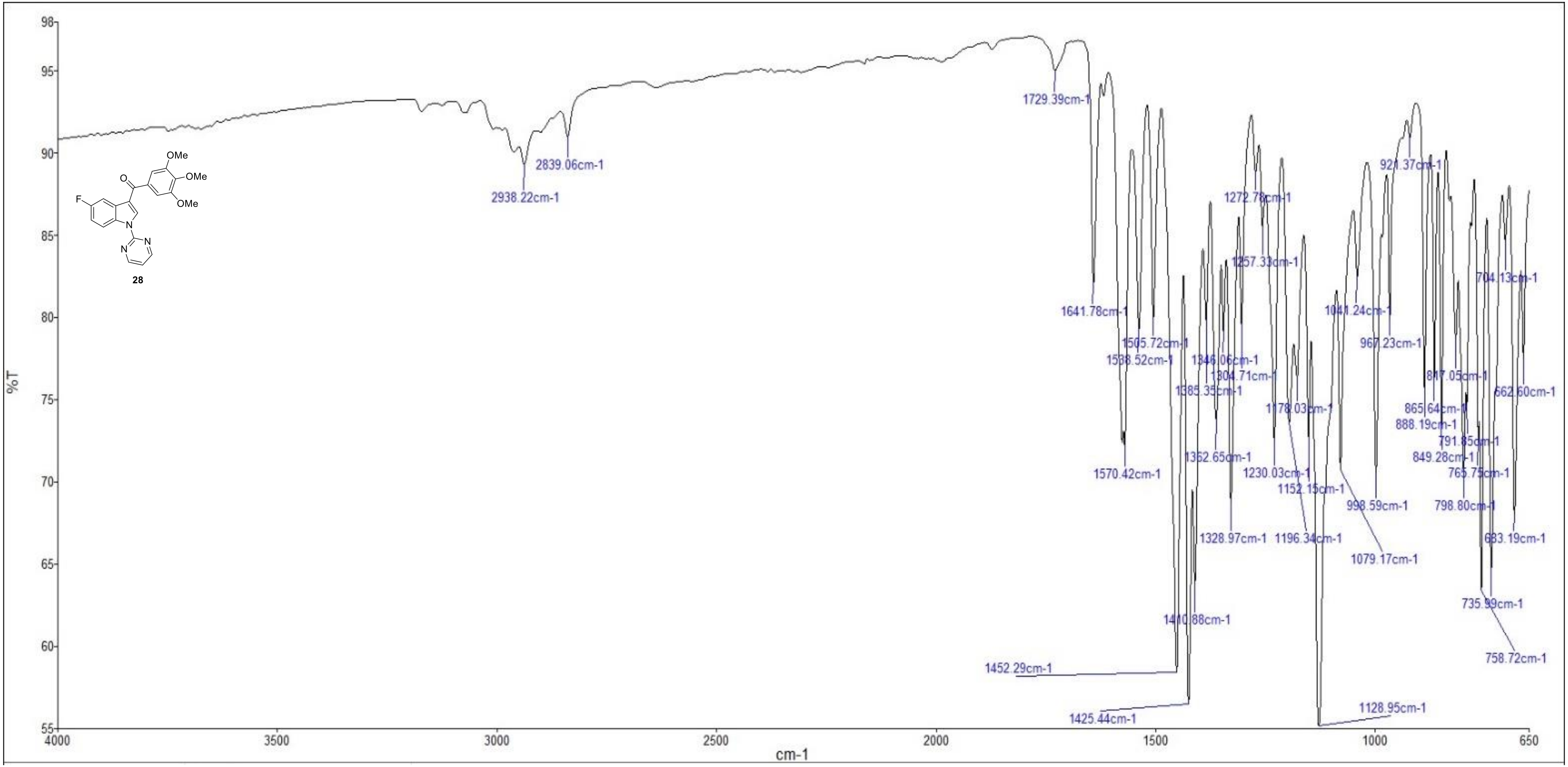

%T
cm-1
98
95
90
85
80
75
70
65
60
55
4000
3500
3000
2500
2000
1500
1000
650
OMe
OMe
OMe
O
F
N
N
N
28
2938.22cm-1
2839.06cm-1
1729.39cm-1
1641.78cm-1
1570.42cm-1
1538.52cm-1
1505.72cm-1
1452.29cm-1
1425.44cm-1
1410.88cm-1
1385.35cm-1
1362.65cm-1
1346.06cm-1
1328.97cm-1
1304.71cm-1
1272.78cm-1
1257.33cm-1
1230.03cm-1
1196.34cm-1
1178.03cm-1
1152.15cm-1
1128.95cm-1
1079.17cm-1
1041.24cm-1
998.59cm-1
967.23cm-1
921.37cm-1
888.19cm-1
865.64cm-1
849.28cm-1
817.05cm-1
798.80cm-1
791.85cm-1
765.75cm-1
758.72cm-1
735.99cm-1
704.13cm-1
683.19cm-1
662.60cm-1

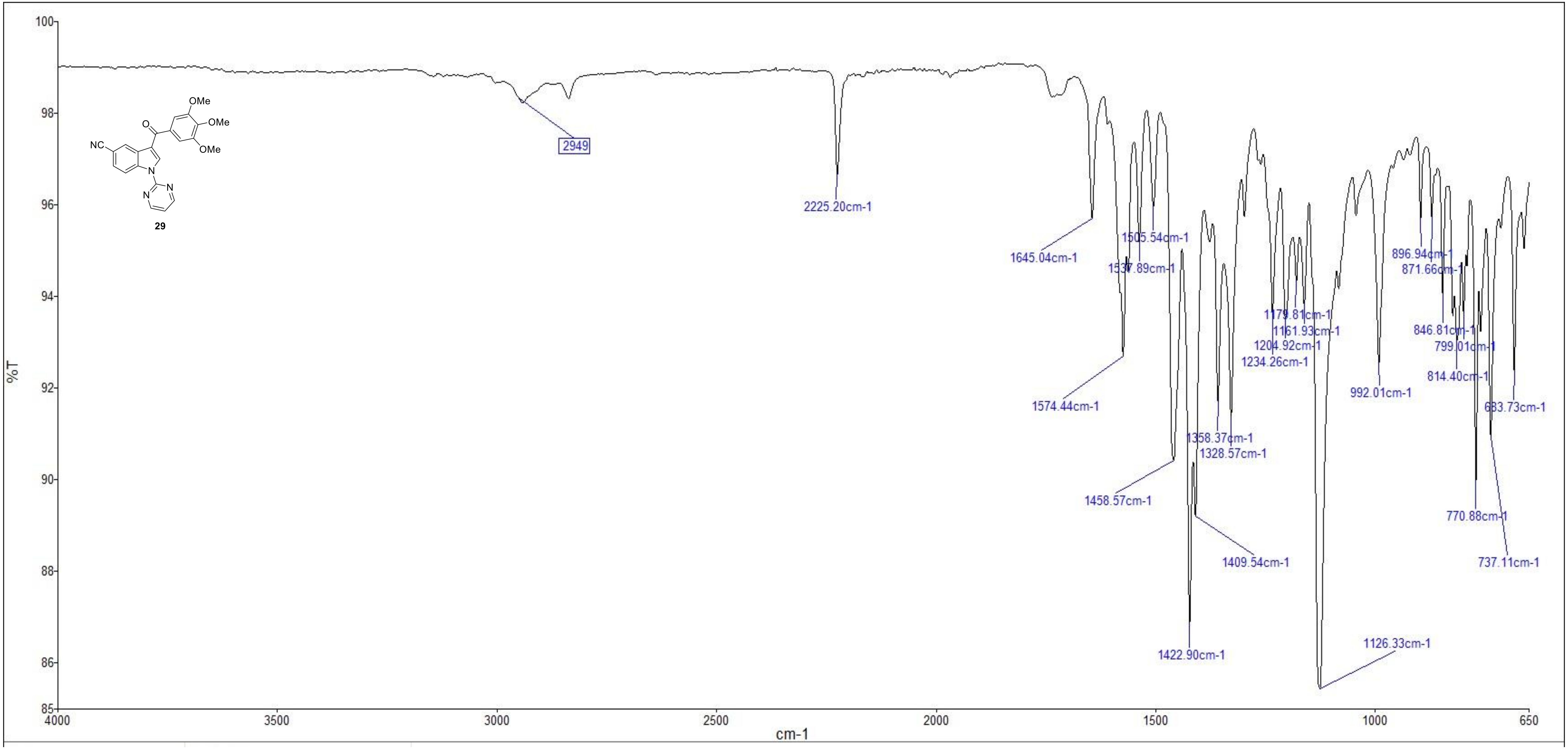
OMe
OMe
OMe
O
NC
N
N
N
29
2949
2225.20cm-1
1645.04cm-1
1505.54cm-1
1537.89cm-1
1574.44cm-1
1458.57cm-1
1422.90cm-1
1409.54cm-1
1358.37cm-1
1328.57cm-1
1234.26cm-1
1204.92cm-1
1179.81cm-1
1161.93cm-1
1126.33cm-1
992.01cm-1
896.94cm-1
871.66cm-1
846.81cm-1
814.40cm-1
799.01cm-1
770.88cm-1
737.11cm-1
683.73cm-1
%T
cm-1
100
98
96
94
92
90
88
86
85
4000
3500
3000
2500
2000
1500
1000
650

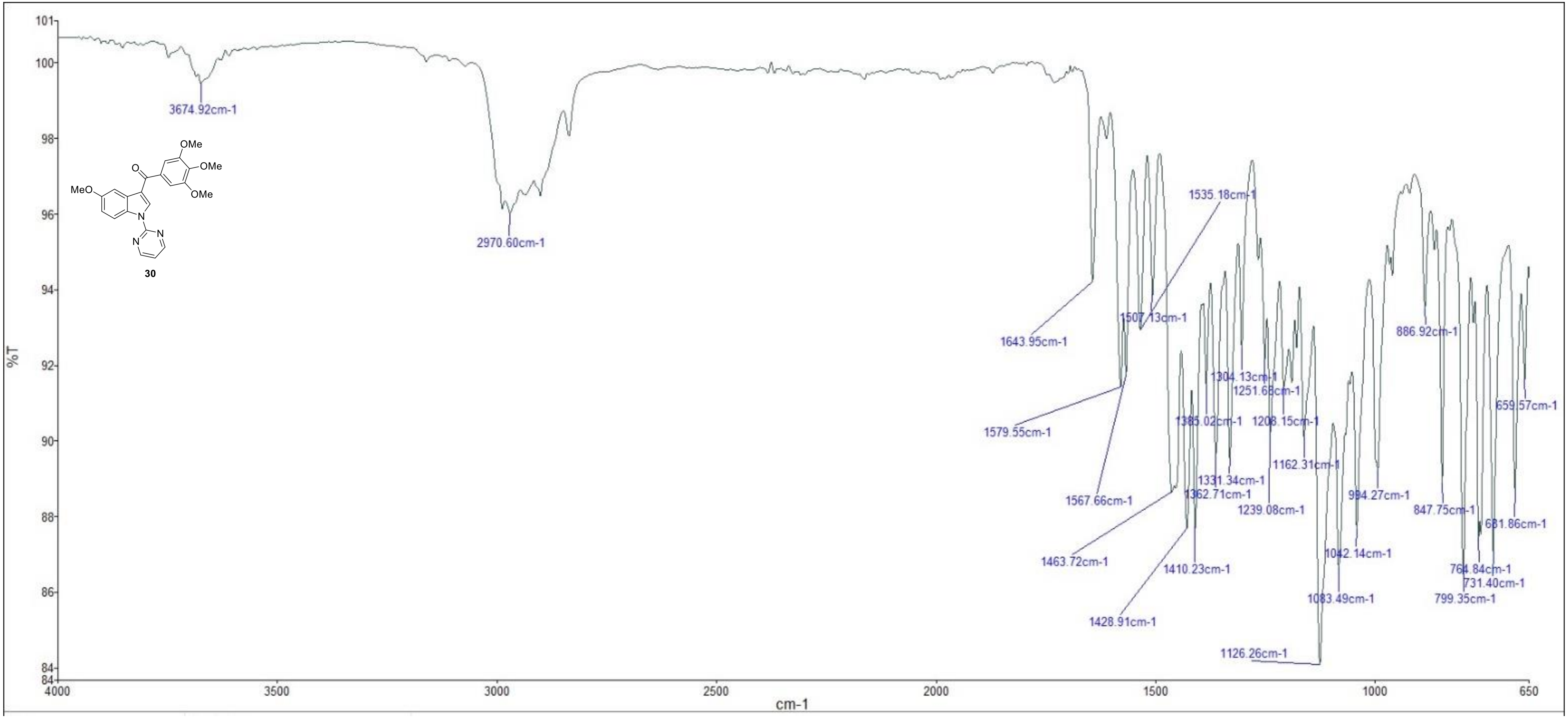

101
100
98
96
94
92
90
88
86
84
%T
4000
3500
3000
2500
2000
1500
1000
650
cm-1
3674.92cm-1
2970.60cm-1
1643.95cm-1
1579.55cm-1
1567.66cm-1
1535.18cm-1
1507.13cm-1
1463.72cm-1
1428.91cm-1
1410.23cm-1
1385.02cm-1
1362.71cm-1
1331.34cm-1
1304.13cm-1
1251.68cm-1
1239.08cm-1
1208.15cm-1
1162.31cm-1
1126.26cm-1
1083.49cm-1
1042.14cm-1
994.27cm-1
886.92cm-1
847.75cm-1
799.35cm-1
764.84cm-1
731.40cm-1
681.86cm-1
659.57cm-1
OMe
OMe
OMe
MeO
O
N
N
N
30

HPLC Chromatograms of Compounds **4**, **14**, **15**, **18**, **20**, **22** and **24**

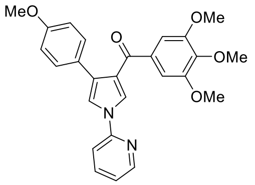

4

Cpd 4

Sample Name:	**Cpd 4**	*Injection Volume:*	**20,0**
Vial Number:	**22**	*Channel:*	**UV_VIS_3**
Sample Type:	**unknown**	*Wavelength:*	**254.0**
Control Program:		*Bandwidth:*	**4**
Quantif. Method:	**default_0_05**	*Dilution Factor:*	**1,0000**
Run Time (min):	**34,00**	*Sample Weight:*	**1,0000**
		Sample Amount:	**1,0000**

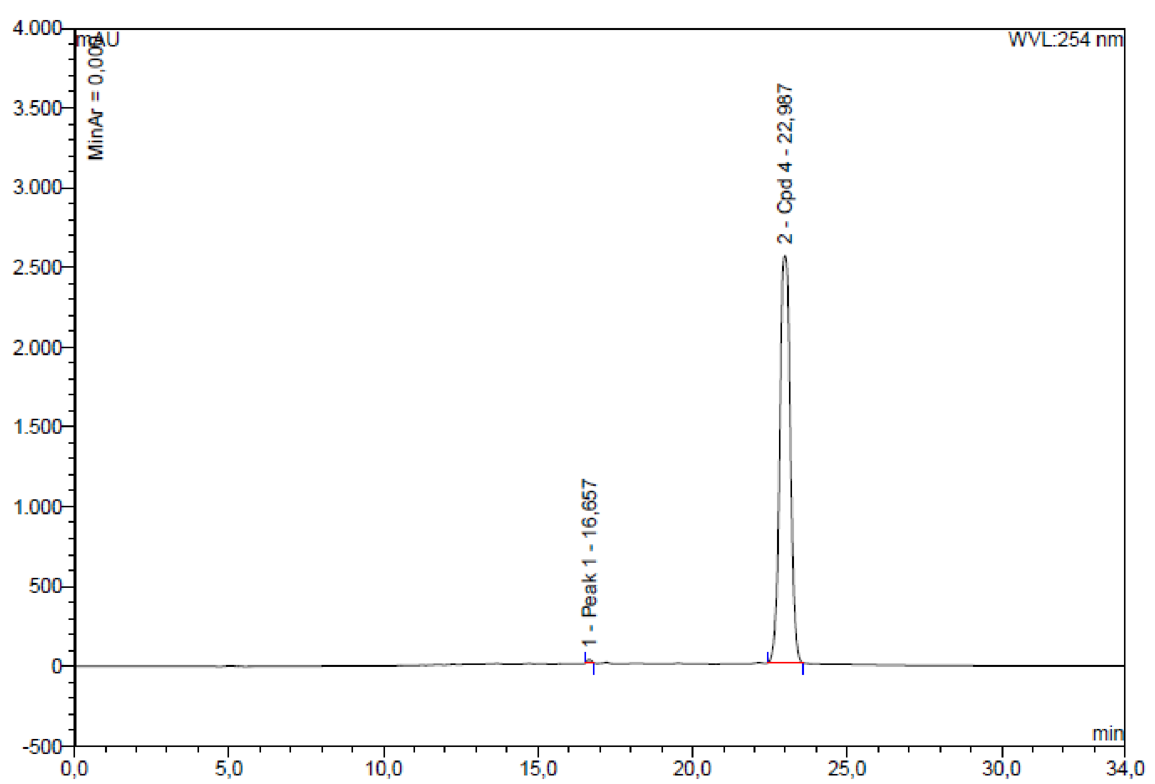

No.	Ret.Time min	Peak Name	Height mAU	Area mAU*min	Rel.Area %	Amount	Type
1	16,66	Peak 1	24,048	3,637	0,36	n.a.	BM *^
2	22,99	Cpd 4	2554,339	1018,404	99,64	n.a.	BMB*
Total:			2578,388	1022,040	100,00	0,000	

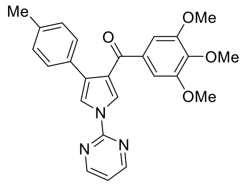

14

Cpd 14

Sample Name:	**Cpd 14**	*Injection Volume:*	**20,0**
Vial Number:	**53**	*Channel:*	**UV_VIS_3**
Sample Type:	**unknown**	*Wavelength:*	**254.0**
Control Program:		*Bandwidth:*	**4**
Quantif. Method:	**default_0_05**	*Dilution Factor:*	**1,0000**
Run Time (min):	**34,00**	*Sample Weight:*	**1,0000**
		Sample Amount:	**1,0000**

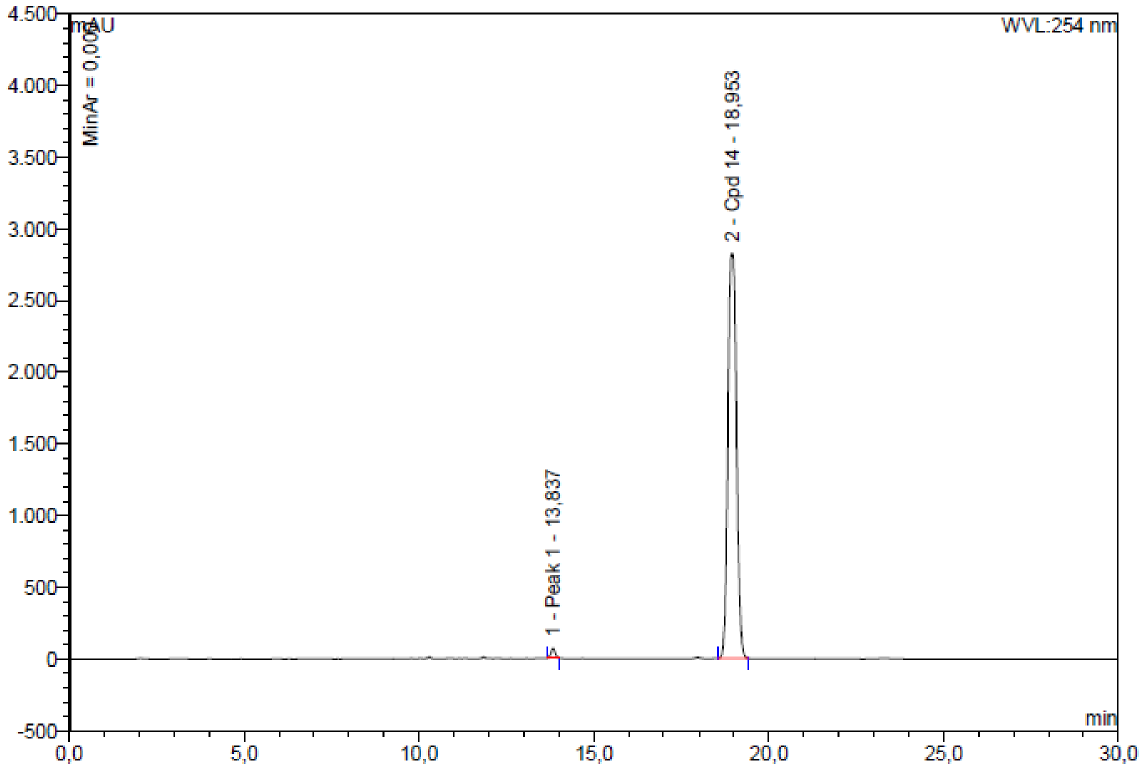

No.	Ret.Time min	Peak Name	Height mAU	Area mAU*min	Rel.Area %	Amount	Type
1	13,84	Peak 1	66,801	9,145	1,14	n.a.	BMB*^
2	18,95	Cpd 14	2827,333	793,189	98,86	n.a.	BMB*^
**Total:**			2894,135	802,334	100,00	0,000	

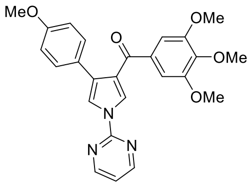

15

Cpd 15

Sample Name:	Cpd 15	*Injection Volume:*	20,0
Vial Number:	29	*Channel:*	UV_VIS_3
Sample Type:	unknown	*Wavelength:*	254.0
Control Program:		*Bandwidth:*	4
Quantif. Method:	default_0_05	*Dilution Factor:*	1,0000
Run Time (min):	34,00	*Sample Weight:*	1,0000
		Sample Amount:	1,0000

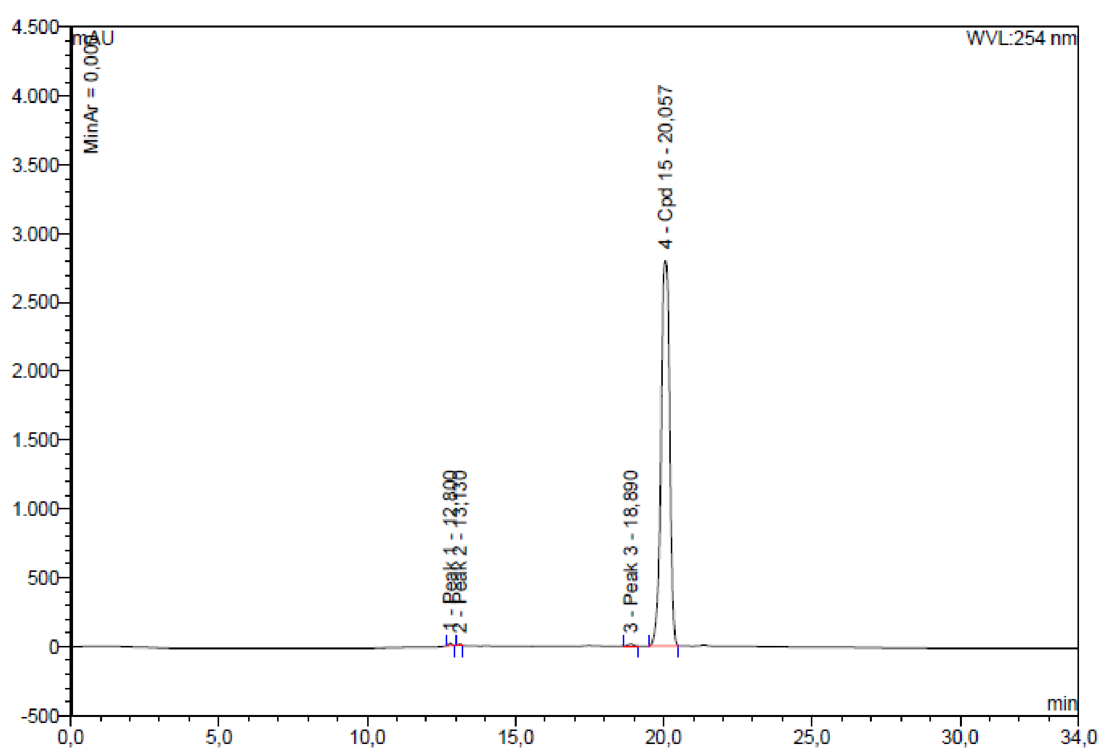

No.	Ret.Time min	Peak Name	Height mAU	Area mAU*min	Rel.Area %	Amount	Type
1	12,80	Peak 1	19,435	2,288	0,24	n.a.	BMB*^
2	13,13	Peak 2	11,621	1,247	0,13	n.a.	BMB*^
3	18,89	Peak 3	15,285	3,348	0,35	n.a.	BMB*^
4	20,06	Cpd 15	2797,019	938,075	99,27	n.a.	BMB*
Total:			2843,361	944,958	100,00	0,000	

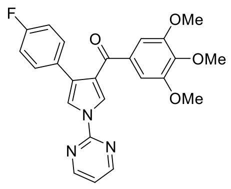

18

Cpd 18

Sample Name:	Cpd 18	*Injection Volume:*	20,0
Vial Number:	53	*Channel:*	UV_VIS_3
Sample Type:	unknown	*Wavelength:*	254.0
Control Program:		*Bandwidth:*	4
Quantif. Method:	default_0_05	*Dilution Factor:*	1,0000
Run Time (min):	30,00	*Sample Weight:*	1,0000
		Sample Amount:	1,0000

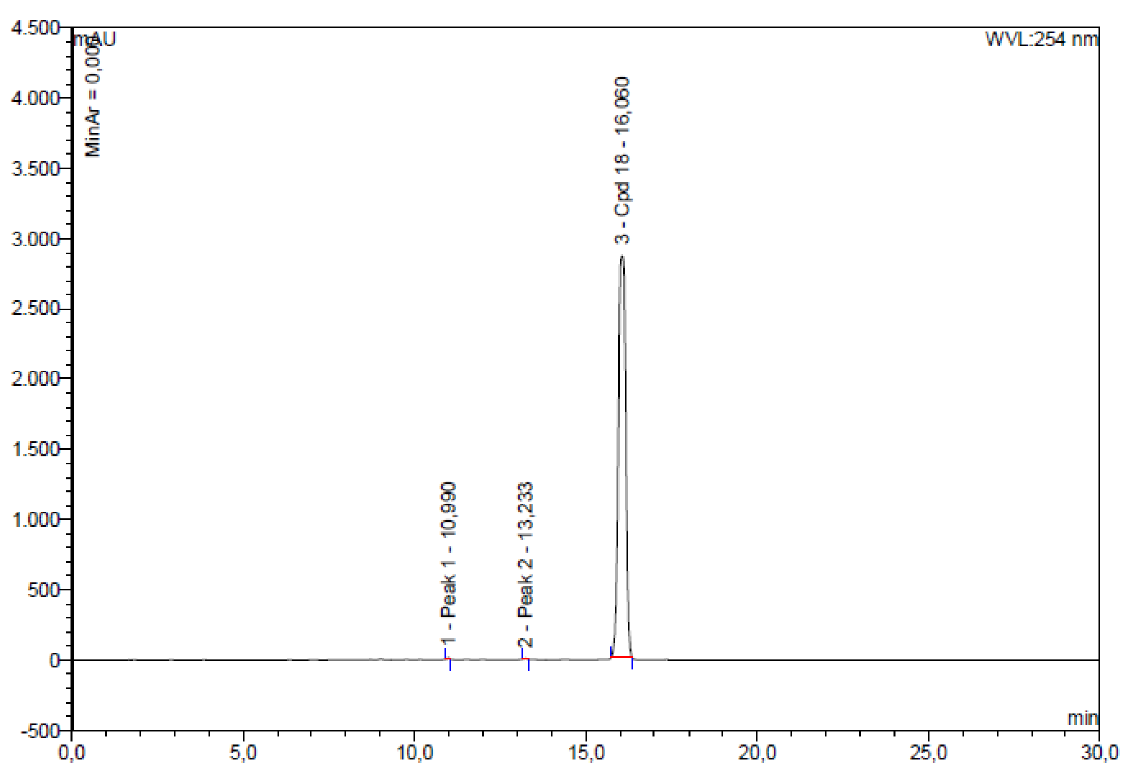

No.	Ret.Time min	Peak Name	Height mAU	Area mAU*min	Rel.Area %	Amount	Type
1	10,99	Peak 1	10,376	0,773	0,11	n.a.	BMB*^
2	13,23	Peak 2	4,346	0,388	0,05	n.a.	BMB*^
3	16,06	Cpd 18	2857,142	708,472	99,84	n.a.	BMB*^
Total:			2871,864	709,633	100,00	0,000	

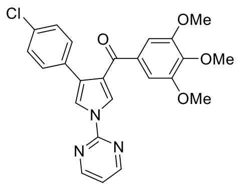

20

Cpd 20

Sample Name:	**Cpd 20**	*Injection Volume:*	**20,0**
Vial Number:	**41**	*Channel:*	**UV_VIS_3**
Sample Type:	**unknown**	*Wavelength:*	**254.0**
Control Program:		*Bandwidth:*	**4**
Quantif. Method:	**default_0_05**	*Dilution Factor:*	**1,0000**
Run Time (min):	**34,00**	*Sample Weight:*	**1,0000**
		Sample Amount:	**1,0000**

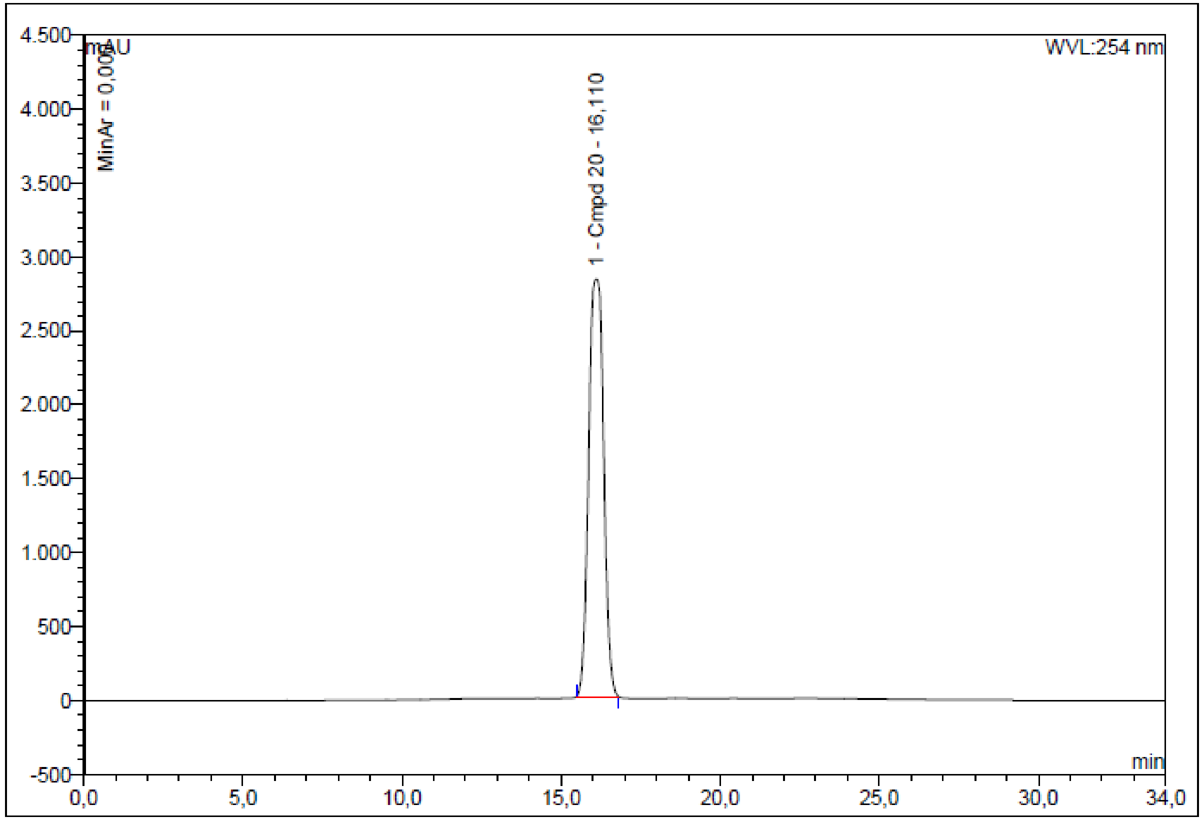

No.	Ret.Time min	Peak Name	Height mAU	Area mAU*min	Rel.Area %	Amount	Type
1	16,11	Cmpd 20	2822,964	1543,254	100,00	n.a.	BMB*
**Total:**			2822,964	1543,254	100,00	0,000	

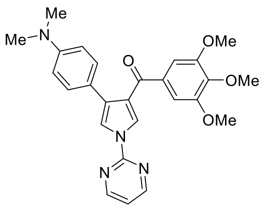

22

Cpd 22

Sample Name:	**Cpd 22**	*Injection Volume:*	**20,0**
Vial Number:	**25**	*Channel:*	**UV_VIS_3**
Sample Type:	**unknown**	*Wavelength:*	**254.0**
Control Program:		*Bandwidth:*	**4**
Quantif. Method:	**default_0_05**	*Dilution Factor:*	**1,0000**
Run Time (min):	**34,00**	*Sample Weight:*	**1,0000**
		Sample Amount:	**1,0000**

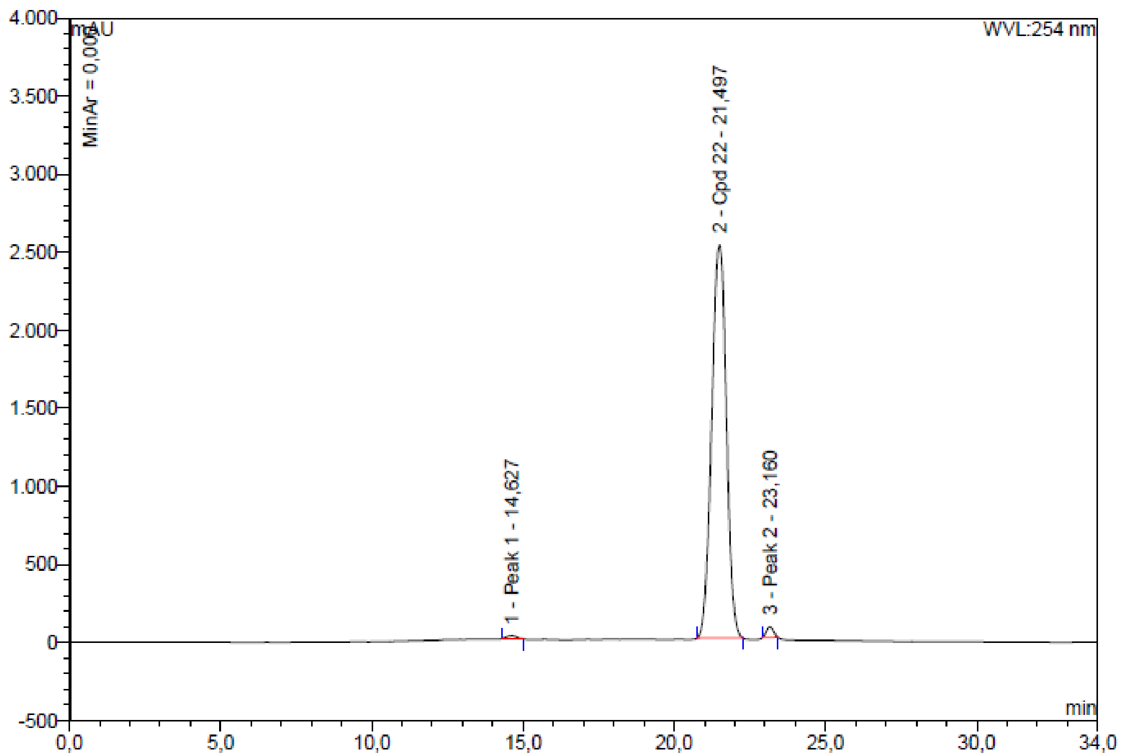

No.	Ret.Time min	Peak Name	Height mAU	Area mAU*min	Rel.Area %	Amount	Type
1	14,63	Peak 1	21,719	8,777	0,61	n.a.	BMB*^
2	21,50	Cpd 22	2516,590	1417,760	98,13	n.a.	BMB*^
3	23,16	Peak 2	66,965	18,311	1,27	n.a.	BMB*^
**Total:**			2605,274	1444,848	100,00	0,000	

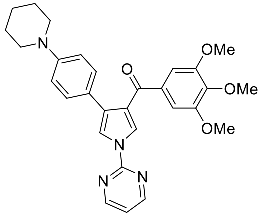

24

Cpd 24

Sample Name:	**Cpd 24**	*Injection Volume:*	**20,0**
Vial Number:	**55**	*Channel:*	**UV_VIS_3**
Sample Type:	**unknown**	*Wavelength:*	**254.0**
Control Program:		*Bandwidth:*	**4**
Quantif. Method:	**default_0_05**	*Dilution Factor:*	**1,0000**
Run Time (min):	**30,00**	*Sample Weight:*	**1,0000**
		Sample Amount:	**1,0000**

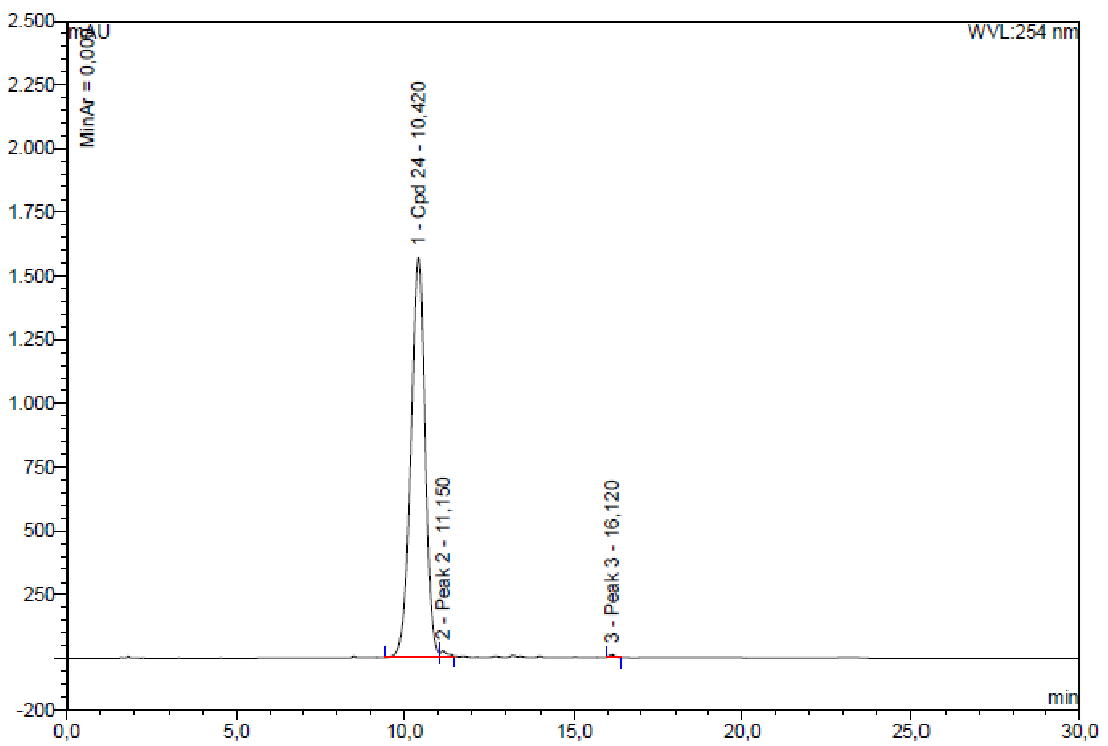

No.	Ret.Time min	Peak Name	Height mAU	Area mAU*min	Rel.Area %	Amount	Type
1	10,42	Cpd 24	1567,201	769,437	98,09	n.a.	BM *
2	11,15	Peak 2	27,614	6,519	0,83	n.a.	M *^
3	16,12	Peak 3	10,508	2,019	0,26	n.a.	BMB*^
**Total:**			1605,323	777,975	100,00	0,000	
